# Continual integration of single-cell multimodal data with MIRACLE

**DOI:** 10.1038/s43588-026-01030-9

**Published:** 2026-07-31

**Authors:** Jiahao Zhou, Jing Wang, Shuofeng Hu, Tongtong Kan, Chao Feng, Xiaohan Qiang, Guohua Dong, Jinhui Shi, Runyan Liu, Xiaochen Bo, Le Ou-Yang, Xiaomin Ying, Zhen He

**Affiliations:** 1 Center for Computational Biology, Beijing Institute of Basic Medical Sciences, Beijing, China; 2 College of Electronics and Information Engineering, Shenzhen University, Shenzhen, China; 3 School of Statistics and Mathematics, Central University of Finance and Economics, Beijing, China; 4 Institute of Health Service and Transfusion Medicine, Beijing, China; 5 Faculty of Engineering, Shenzhen MSU-BIT University, Shenzhen, China

**Keywords:** Machine learning, Bioinformatics, Data integration

## Abstract

Single-cell sequencing has transformed our understanding of cellular heterogeneity, enabling the construction of multi-omics atlases through data integration. However, conventional atlas updates require full reintegration of all datasets, creating scalability challenges that limit the timeliness and adaptability of biomedical research. Here we present multimodal integration with continual learning (MIRACLE), an online learning framework for scalable multimodal integration. Using dynamic architecture adaptation and data rehearsal, MIRACLE continually integrates diverse datasets while preserving biological fidelity. Across evaluations, MIRACLE achieves accurate online integration with substantially improved efficiency, refining and expanding atlases with new cross-modal, cross-tissue and cross-disease data. Applied to respiratory infections, it reveals both shared and pathogen-specific immune mechanisms in coronavirus disease 2019, influenza A and tuberculosis. Overall, MIRACLE provides an efficient and collaborative solution for the continual integration, sharing and exploration of biological knowledge.

## Abstract (web summary)

MIRACLE is an online continual learning framework that efficiently integrates single-cell multimodal data across batches, modalities, tissues, and diseases.

## Main

Single-cell sequencing technologies enable the high-resolution characterization of functional molecules within individual cells, extending biological exploration to the single-cell level. Integrating and analyzing single-cell data across omics and sources consolidates biological knowledge from heterogeneous data, advancing our understanding of cellular functions^1–3^. Large-scale single-cell atlases capturing diverse cell states and types provide a comprehensive reference for studying cell heterogeneity, facilitating insights into molecular mechanisms underlying development, disease and other biological processes^4,5^.

However, single-cell sequencing data are generated asynchronously and continuously, often emphasizing distinct biological questions and containing varied biological knowledge. As new data accumulate, their integration is crucial for refining and expanding existing atlases to enhance biological insights. Addressing this scenario of continual integration necessitates methods that support continuous updates while maintaining consistency and accuracy.

Most existing integration approaches operate in an offline manner, which require all data to be available simultaneously^6–9^. Consequently, each update demands reprocessing the entire dataset, leading to escalating computational costs. To address this, online integration methods have been proposed^10–13^, enabling atlas updates using only new data and improving efficiency.

Despite these advances, existing online methods face critical limitations. Generalization-based approaches^11,13,14^ map new data onto a reference using pretrained models, but their performance depends heavily on the scale and diversity of training data and they struggle to capture previously unseen biological or technical variations. Fine-tuning-based approaches^10,12,15^ adapt to new inputs but tend to focus on the current data, causing catastrophic forgetting—the gradual loss of previously learned knowledge—which hinders the accumulation of biological insights over time. Moreover, existing online methods are modality-specific and cannot handle mosaic data, where new datasets introduce different omics and features^16^. A more flexible and robust framework is therefore needed.

To address these challenges, we introduce multimodal integration with continual learning (MIRACLE), an online learning framework for multimodal single-cell integration via continual learning (CL). CL enables models to incrementally incorporate new data while preserving previously acquired knowledge^17,18^. MIRACLE employs CL strategies including dynamic architecture adaptation and data rehearsal to enhance adaptability and knowledge retention. It leverages MIDAS^19^ as a base model to support the integration of multimodal mosaic data, enabling online updates across diverse omics. MIRACLE also facilitates precise query mapping to reference atlases, improving the accuracy of cell label transfer and the discovery of previously unrecognized cell types.

Systematic evaluation demonstrates that MIRACLE efficiently integrates multimodal data across batches, modalities and cell types with constrained memory usage, achieving accuracy comparable to offline methods while substantially reducing computational costs. By continually incorporating cross-modal, cross-tissue and cross-disease data, MIRACLE enables dynamic atlas refinement and expansion. Applied to respiratory infection datasets, MIRACLE uncovers both shared and pathogen-specific mechanisms in coronavirus disease 2019 (COVID-19), influenza A (flu A) and tuberculosis (TB), showcasing its translational potential in immunological research. Users can update and share MIRACLE for collaboration, enabling scalable single-cell analysis and iterative biological exploration through its extensible, open design.

## Results

### The MIRACLE model

**Fig. 1:**
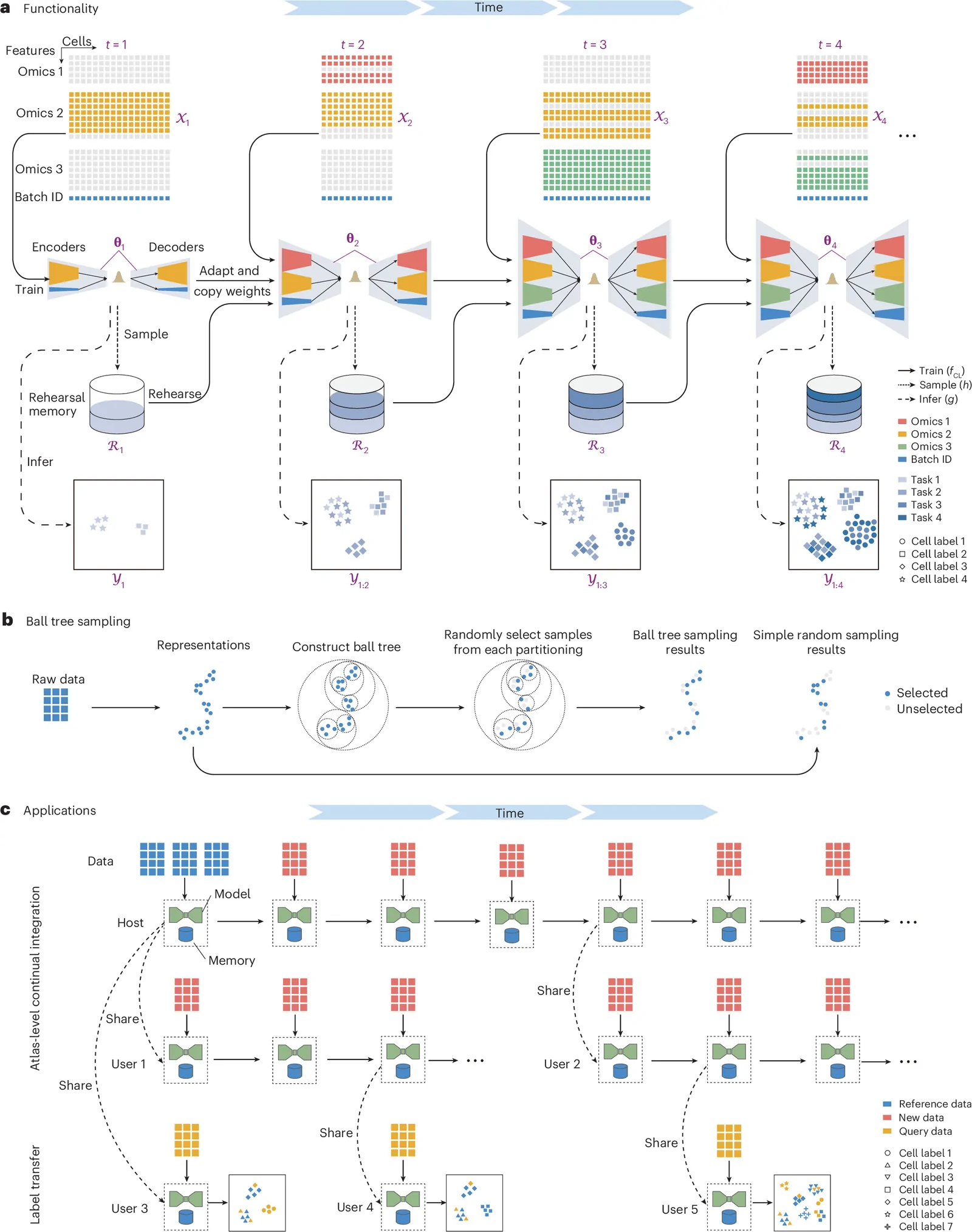
Overview of the MIRACLE framework for continual multimodal integration. **a**, At time *t* = 1, a model is trained on data ${{\mathcal{X}}}_{1}$ using function *f*_CL_, generating integration results ${{\mathcal{Y}}}_{1}$ via inference function *g*. Rehearsal memory ${{\mathcal{R}}}_{1}$ is then created using sampling function *h*. For *t* > 1, new data ${{\mathcal{X}}}_{t}$ is integrated by updating the previous model **θ**_*t*−1_ to obtain the new model **θ**_*t*_ via *f*_CL_. The inference function *g* produces integration results ${{\mathcal{Y}}}_{1:t}$ for all data observed up to time *t*, and rehearsal memory is updated from ${{\mathcal{R}}}_{t-1}$ to ${{\mathcal{R}}}_{t}$. **b**, BTS partitions the embedding space with a ball tree and draws samples from each partition, better preserving the data distribution than simple random sampling. **c**, To achieve atlas-level continual integration, a host can begin by building a model using reference data and then continuously incorporate new data into this model. Users can also continuously update the model with their own data and reshare it, or use the model to perform label transfer on their data.

MIRACLE is a deep generative model^20,21^ combined with CL to incrementally integrate single-cell multimodal mosaic datasets of assays for transposase-accessible chromatin (ATAC), RNA and antibody-derived tags (ADT; Fig. 1a). The input data may be collected at different time points and exhibit variations in biological information, technical noise and modality combinations. Whenever new data arrive, MIRACLE can incorporate them to update the existing integration results, removing batch effects while preserving biological variation.

MIRACLE uses MIDAS, a multimodal variational autoencoder (VAE)^22^, as the base model to better handle mosaic data (Methods). A dynamic architecture approach lets the model adapt to newly added modalities and features, while a data rehearsal strategy mitigates forgetting during continual integration. We further introduced a ball tree sampling (BTS) approach to bound the rehearsal memory while preserving the data distribution, enabling memory-efficient training (Fig. 1b).

With MIRACLE, one can construct large-scale single-cell multimodal atlases and update them by continuously integrating newly added mosaic data (Fig. 1c). A model updated at any time point can be shared with other users, who can update it with their own data and reshare it, or use it to transfer labels to their data—enabling efficient knowledge reuse in practice.

### Continual integration under limited memory

MIRACLE employs a rehearsal-based strategy for continual integration. To ensure computational efficiency, we impose a limit on the memory capacity—the maximum number of single cells retained in the rehearsal memory. When the stored data exceed this capacity, downsampling is performed. Here, we propose the distribution-preserving reservoir sampling (DPRS) approach (Methods), which can dynamically limit the memory capacity while effectively preserving the intrinsic distribution of the data, ultimately obtaining unbiased continual integration results. We validated our approach on a large-scale single-nucleus RNA sequencing (snRNA-seq) dataset of human dilated and hypertrophic cardiomyopathy (DHCM)^23^, containing 523,369 cells across 42 batches.

**Extended Data Fig. 1:**
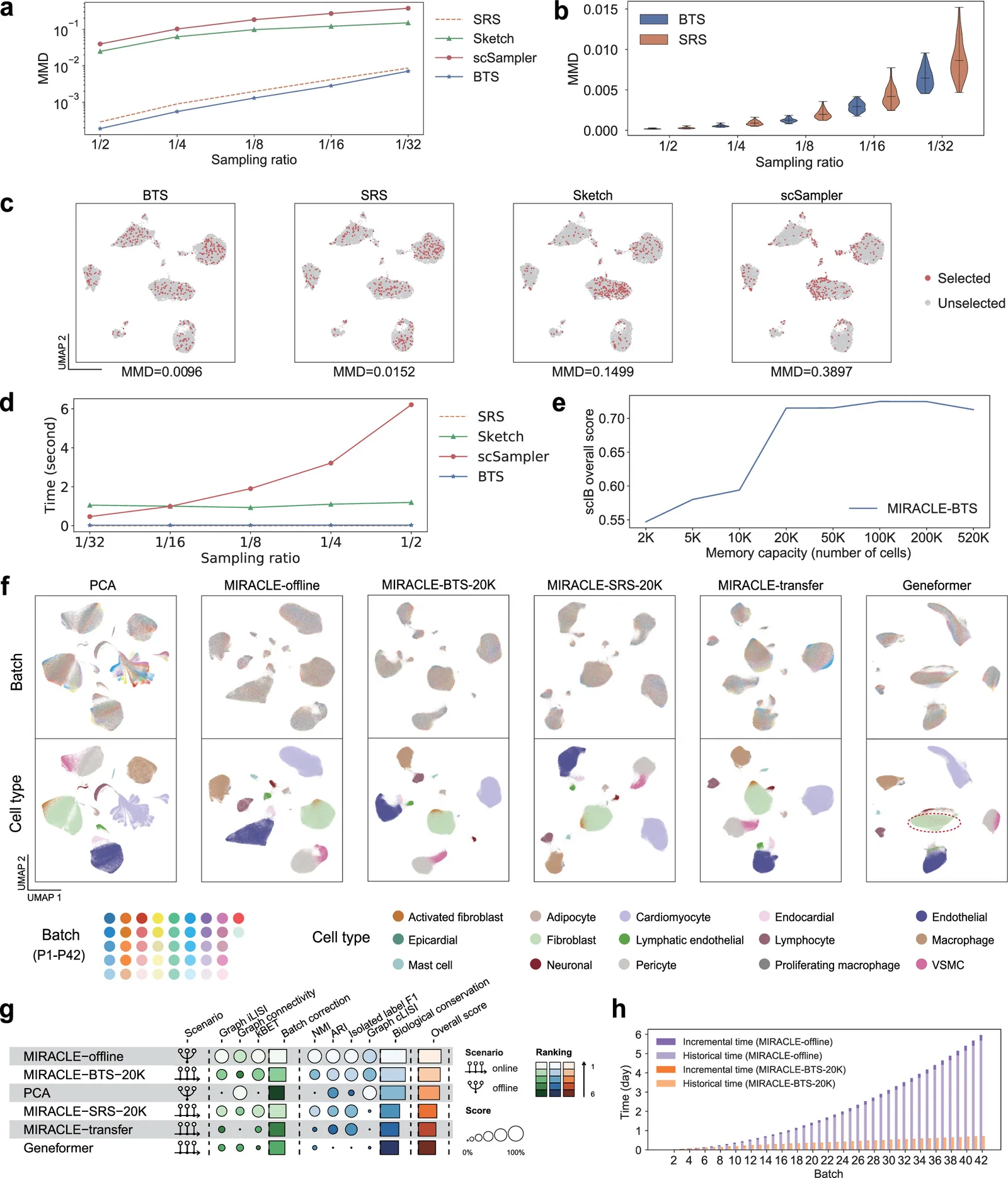
Evaluation of MIRACLE on the DHCM snRNA-seq dataset under memory constraints. **a**, Mean MMD for SRS, Sketch, scSampler, and BTS at different sampling ratios. **b**, Violin plots comparing MMD values of BTS and SRS across sampling ratios. **c**, UMAP visualization of maximum MMD results for each sampling method at a 1/32 sampling ratio. **d**, Runtime comparison of sampling methods across ratios from 1/32 to 1/2. **e**, scIB overall score of MIRACLE-BTS under varying memory constraints. **f**, UMAP visualization of cell embeddings inferred by MIRACLE-BTS-20K and other methods. In the Geneformer result, the Fibroblast and Activated fibroblast region is highlighted (red circle). **g**, scIB benchmarking of MIRACLE under different settings compared with other online and offline methods. **h**, Runtime comparison between MIRACLE-offline and MIRACLE-BTS-20K. Violin plots (b): violin, data distribution; center line, mean; caps, minimum and maximum; *n* = 50 independent subsampling repetitions per method and ratio. Source data

We first assessed the distribution-preserving capability of BTS, a core component of DPRS. Operating in the embedding space, we compared BTS against simple random sampling (SRS), Sketch^24^ and scSampler^25^ across different sampling ratios, quantifying the dissimilarity between the original and sampled distributions with the maximum mean discrepancy (MMD), where lower is better (Methods). Both BTS and SRS achieved much lower MMDs than Sketch and scSampler, with BTS attaining the lowest values and consistently smaller mean and variance than SRS across ratios (Extended Data Fig. 1a,b). Uniform manifold approximation and projection (UMAP) visualization at the lowest ratio of 1/32 confirmed that BTS best preserved the data distribution (Extended Data Fig. 1c). BTS was also as fast as SRS and robust to the sampling ratio, whereas scSampler and Sketch were slower, with scSampler scaling poorly with the ratio (Extended Data Fig. 1d).

Having demonstrated the superiority of BTS, we then explored how different memory capacities impact the continual integration performance of the BTS-based MIRACLE (MIRACLE-BTS; Extended Data Fig. 1e and Supplementary Fig. 1). The integration performance was quantitatively evaluated using single-cell integration benchmarking (scIB^26^; Methods). It was observed that MIRACLE’s performance improved as the memory capacity increased and reached a saturation point at 20K cells, which accounted for only 1/26 of the total sample size.

We then compared MIRACLE-BTS-20K against MIRACLE-SRS-20K (SRS-based, 20K memory), offline principal component analysis (PCA), the pretrained Geneformer^27^ and two key benchmarks (Methods): MIRACLE-offline (trained on all data at once, the upper benchmark free of forgetting) and MIRACLE-transfer (no memory rehearsal, the lower benchmark prone to forgetting). UMAP visualization showed that MIRACLE-offline, MIRACLE-BTS-20K and MIRACLE-SRS-20K removed batch effects while preserving biological differences (Extended Data Fig. 1f), whereas PCA and MIRACLE-transfer failed to mix batches and Geneformer could not distinguish cell types such as ‘Fibroblast’ and ‘Activated fibroblast’. Accordingly, MIRACLE-BTS-20K performed comparably to MIRACLE-offline and outperformed PCA on scIB, while using SRS, omitting rehearsal or relying on a pretrained model alone substantially degraded performance (Extended Data Fig. 1g and Supplementary Table 1).

We also compared the time efficiency during continual integration (Extended Data Fig. 1h and Supplementary Fig. 2). Because MIRACLE-offline reintegrates all batches whenever a new one arrives, its per-integration and cumulative time rose sharply with the number of batches. In contrast, MIRACLE-BTS-20K’s per-integration time was unaffected by batch number, keeping cumulative time substantially lower, and its computational memory footprint remained low and constant, whereas PCA’s scaled linearly with accumulating data (Supplementary Fig. 3). These scalability advantages held on the 2.3 million-cell Human Lung Cell Atlas (HLCA)^4^, a larger and more challenging benchmark with pronounced technical artifacts, where MIRACLE achieved superior accuracy, ran over an order of magnitude faster than its offline counterpart and again maintained a constant, low memory footprint (Supplementary Fig. 4 and Supplementary Table 2).

### Continual integration across batches and cell types

Multimodal single-cell datasets frequently arise from diverse platforms or subjects, requiring batch effect removal while preserving biological variation, including cell-type composition, during continual integration. We therefore used a bimodal dataset of eight CITE-seq batches of human peripheral blood mononuclear cells (PBMCs) from the weighted-nearest neighbor (WNN) study^28^—the WNN dataset—removing specific cell types so that each batch held a unique cell-type combination (Methods and Supplementary Table 3).

**Fig. 2:**
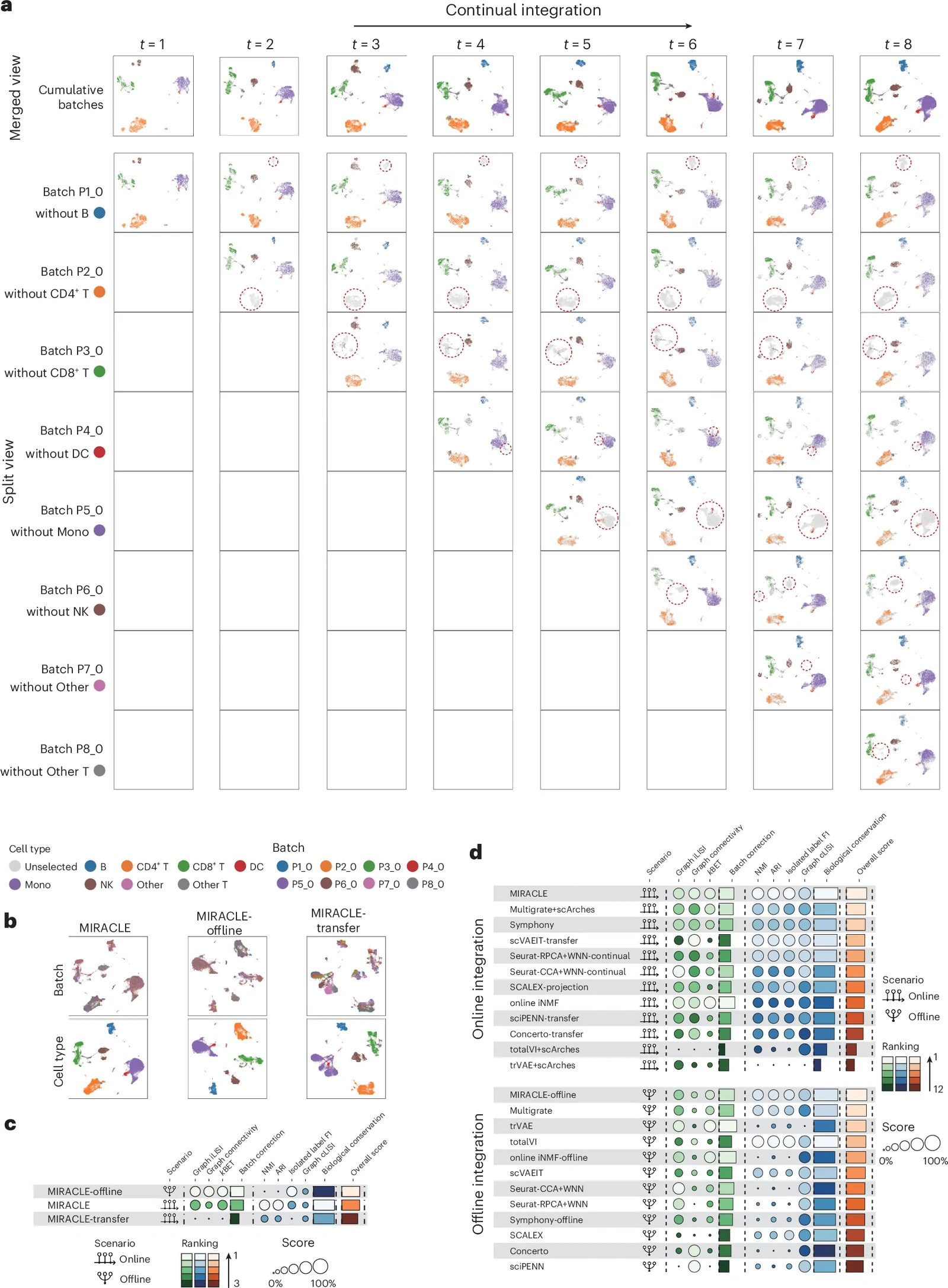
Evaluation of MIRACLE on the WNN bimodal dataset across batches and cell types. **a**, UMAP visualization of MIRACLE-inferred cell embeddings across time points, with each column corresponding to a specific time point. Row 1 shows the merged view of the cumulative integration of all available batches over time. Rows 2–9 show the split view, where each row displays the integration results for a single batch at different time points, with missing cell type regions highlighted (red dashed circles); the blank lower-triangular area reflects the stepwise nature of continual integration, as data from future batches are not yet available. Cells are colored by cell type. **b**, UMAP visualization of cell embeddings inferred by MIRACLE and its variants. **c**, scIB benchmarking comparing MIRACLE with its variants. **d**, scIB benchmarking comparing MIRACLE with 11 state-of-the-art single-cell integration methods in online (top) and offline (bottom) scenarios. DC, dendritic cell; NK, natural killer cell; mono, monocytes. Source data

We first continually integrated the WNN dataset with MIRACLE. The UMAP visualization of the low-dimensional cell embeddings showed that MIRACLE robustly integrated new batches into the existing batches and updated all integration results successively, effectively removing batch effects while preserving differences in cell type composition across different batches (Fig. 2a and Supplementary Fig. 5).

To validate our memory rehearsal strategy, we compared MIRACLE with MIRACLE-offline (gold standard, no forgetting) and MIRACLE-transfer (negative control, no rehearsal). Both MIRACLE and MIRACLE-offline mixed batches well while retaining cell-type information (Fig. 2b), whereas MIRACLE-transfer failed to mix batches, forming multiple internal clusters within the ‘Mono’ cells. Consistently, scIB showed MIRACLE performing comparably to MIRACLE-offline and surpassing MIRACLE-transfer, which was weak in batch correction (Fig. 2c), and CL metrics confirmed MIRACLE’s stable performance over time versus the degradation of MIRACLE-transfer (Methods and Supplementary Fig. 6). These results demonstrate the efficacy of memory rehearsal in tackling catastrophic forgetting.

We next compared MIRACLE against 11 state-of-the-art integration methods on WNN in both online and offline settings, applying fine-tuning-based transfer learning to methods lacking online capability (Methods). Under both settings, MIRACLE consistently mixed batches while clustering shared cell types (Supplementary Fig. 7). Several methods that performed well offline degraded markedly online owing to catastrophic forgetting; for example, totalVI and trVAE removed batch effects well, but their online versions (totalVI-scArches and trVAE-scArches) failed to mix batches. Accordingly, MIRACLE and MIRACLE-offline achieved the highest scIB scores in the online and offline scenarios, respectively, while the online counterparts of the top offline methods (trVAE and totalVI) lagged behind (Fig. 2d and Supplementary Tables 4 and 5). Superior performance on a more demanding dataset with complex cell-type dynamics (Supplementary Figs. 8 and 9 and Supplementary Tables 6–8) reinforced MIRACLE’s advantage in continually integrating multi-omics data across batches and cell types.

### Continual mosaic integration

To evaluate continual integration across modality combinations, we combined two trimodal human PBMC datasets that jointly profile RNA, ADT and ATAC—DOGMA-seq^29^ and TEA-seq^30^—to construct the DOTEA dataset. Selecting four batches from each and removing certain omics and cell types, we obtained a mosaic dataset of eight batches spanning four omics combinations (ATAC+RNA, ATAC+ADT, RNA+ADT and ATAC+RNA+ADT) with diverse cell types (Methods and Supplementary Table 9).

We first visualized the cell embeddings and MIRACLE-inferred technical noise at successive integration stages (Supplementary Fig. 10). As more batches were integrated, newly added batches aligned well while knowledge from prior batches was retained without catastrophic forgetting, even when omics and cell types were missing. After integrating all batches, the distributions of both cell embeddings and technical noise were highly similar across modalities (Supplementary Fig. 11), showing that MIRACLE aligns modalities effectively.

**Fig. 3:**
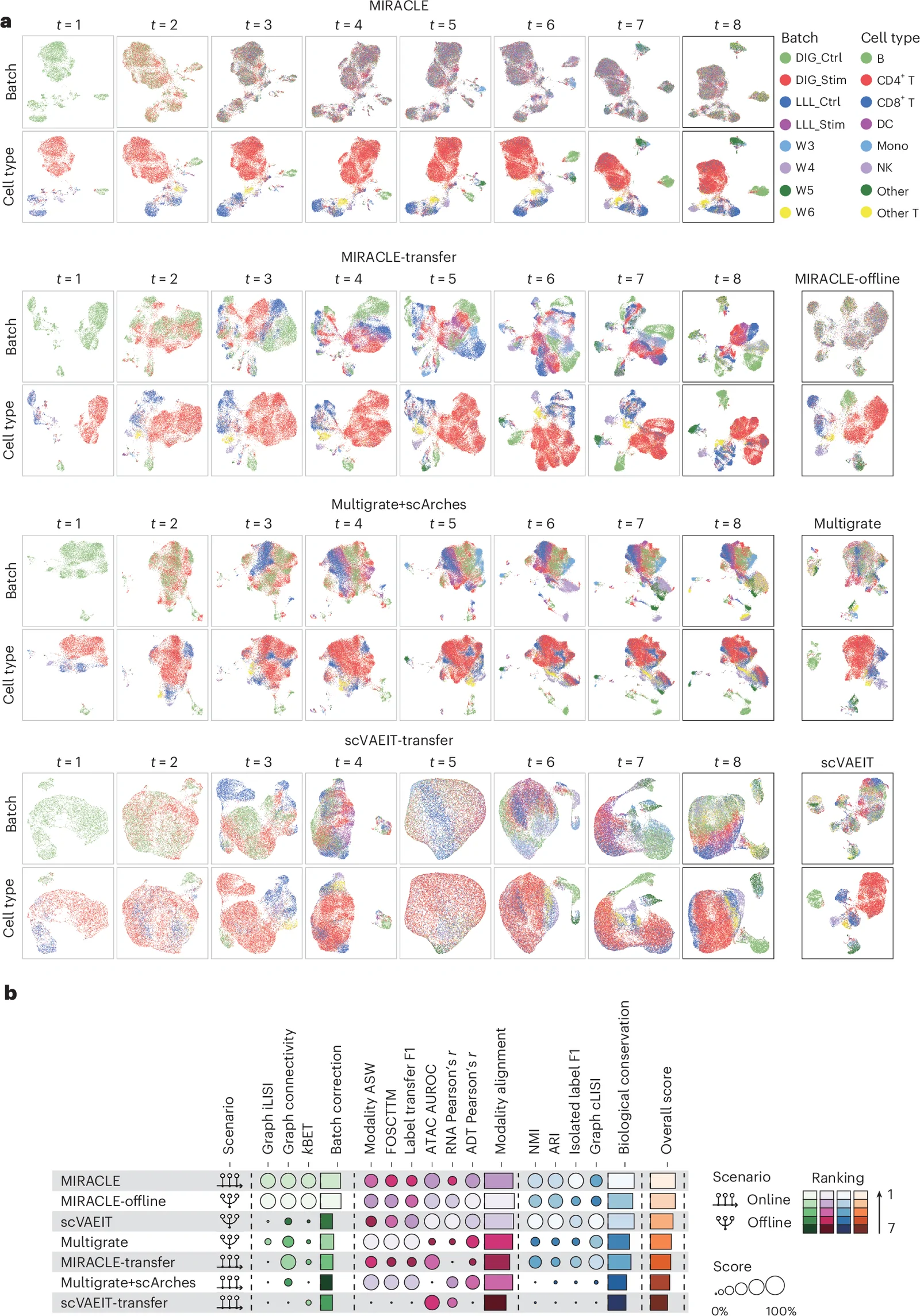
Evaluation of MIRACLE on the DOTEA trimodal dataset in mosaic integration. **a**, UMAP visualization of cell embeddings across time points for online methods and across all cells for offline methods. Cells are colored by batch and cell type. **b**, scMIB benchmarking comparing MIRACLE with Multigrate and scVAEIT in online and offline settings. Source data

We then benchmarked MIRACLE against leading mosaic integration methods online and offline. MIRACLE performed on par with MIRACLE-offline and far better than MIRACLE-transfer, preserving biological information while removing batch effects (Fig. 3a and Supplementary Fig. 12); Multigrate+scArches and scVAEIT-transfer failed to remove batch effects or retain cell-type information as batches accumulated, even offline. Quantitatively, using scMIB^19^ (Methods), MIRACLE achieved the highest overall score, considerably surpassing scVAEIT, Multigrate and especially their online versions, with MIRACLE and MIRACLE-offline excelling in batch correction (Fig. 3b and Supplementary Table 10); notably, MIRACLE’s overall score was slightly higher than MIRACLE-offline’s (Discussion). CL metrics further quantified the severe forgetting in MIRACLE-transfer that MIRACLE avoids (Methods and Supplementary Fig. 6). These findings show that MIRACLE continually integrates data across batches, modalities and cell types.

### Continual construction of a cross-tissue multimodal atlas

Because atlases are often built from large, high-dimensional datasets, reintegrating all data at each update is costly, motivating online integration. We evaluated MIRACLE in atlas-level continual integration under within- and cross-tissue scenarios. For the within-tissue scenario, we collected 10 human PBMC datasets (34 batches), using 28 batches (atlas-no-nac) to build the initial atlas and 6 new batches (nac) for updating, with two batches each from NEAT-seq (ATAC+RNA)^31^, ASAP-seq (ATAC+ADT)^29^ and ASAP-CITE (RNA+ADT; a CITE-seq dataset from the ASAP-seq study). For the cross-tissue scenario, we further added three human-immunity batches from tonsil (RNA)^32^, bone marrow mononuclear cell (BMMC; ATAC+ADT)^29^ and spleen (RNA)^33^ to update the 34-batch PBMC atlas (Methods and Supplementary Table 11).

In each scenario, we integrated the reference batches offline and then the new batches online from the pretrained model, comparing three online strategies: the default CL-based MIRACLE, MIRACLE-generalization (inferring embeddings directly from the pretrained model)^11,13^ and MIRACLE-transfer (fine-tuning on the new batches)^12,15^. MIRACLE-offline integrating all batches offline provided a gold standard for quantitative evaluation (Supplementary Fig. 13).

**Fig. 4:**
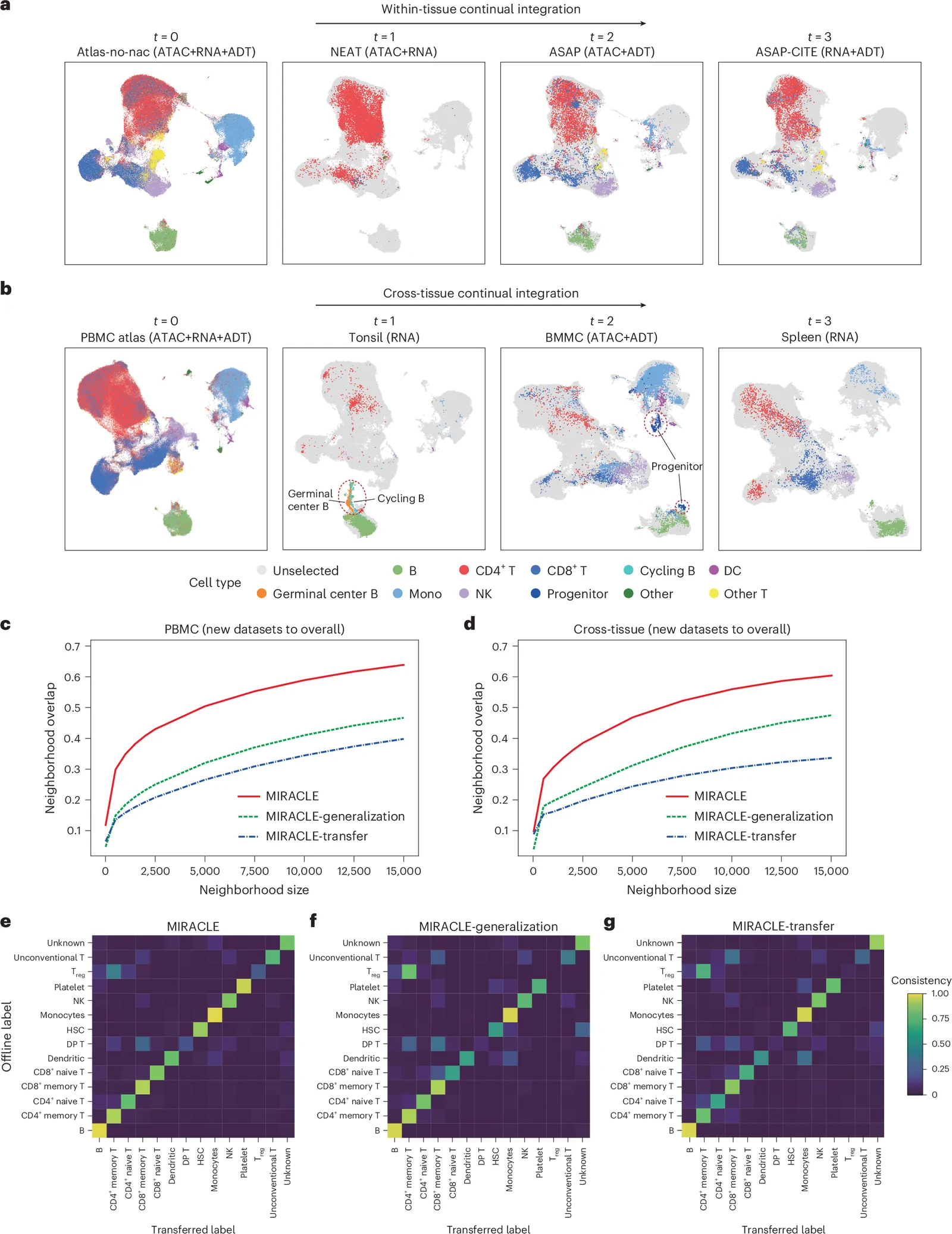
Evaluation of MIRACLE in within- and cross-tissue atlas construction. **a**,**b**, UMAP visualization of MIRACLE-inferred cell embeddings for within-tissue (**a**) and cross-tissue (**b**) continual atlas construction. New cell types in the cross-tissue data (**b**) are highlighted (red circles). **c,d**, Neighborhood overlap between each online strategy and MIRACLE-offline for the newly added data across neighborhood sizes, in within-tissue (**c**) and cross-tissue (**d**) settings. **e**–**g**, Label confusion plots comparing transfer consistency for MIRACLE (**e**), MIRACLE-generalization (**f**) and MIRACLE-transfer (**g**) in cross-tissue continual integration. DP T, double positive T cell; HSC, hematopoietic stem cell. Source data

UMAP visualizations showed that MIRACLE maintained cell-type information while removing batch effects (Fig. 4a,b), with new cell types in cross-tissue data (‘Germinal center B’ and ‘Cycling B’ cells in tonsil, ‘Progenitor’ cells in bone marrow) forming distinct clusters (Fig. 4b). It also preserved finer-grained variation, distinguishing tissue-specific states such as circulating memory T cells in PBMCs from terminally differentiated effector T cells in spleen (Supplementary Fig. 14), and matched MIRACLE-offline in retaining prior knowledge (Supplementary Figs. 15 and 16). By contrast, MIRACLE-generalization preserved cell-type information for the new PBMC batches but could not identify previously unseen cross-tissue cell types (Supplementary Fig. 17a,b), and MIRACLE-transfer failed to distinguish cell types or remove batch effects in either scenario (Supplementary Fig. 17c,d).

We then quantified cell-neighborhood consistency between online and offline results by computing, for each online strategy, the neighborhood overlap with MIRACLE-offline. Both MIRACLE and MIRACLE-generalization showed substantially higher overlap than MIRACLE-transfer (Supplementary Fig. 18a,b); however, for the new data specifically, MIRACLE’s overlap far exceeded that of the other two strategies, indicating more appropriate integration of new data (Fig. 4c,d). Cell-type consistency assessed by label transfer agreed: confusion plots showed that MIRACLE’s transferred labels closely matched MIRACLE-offline and were substantially more accurate than the alternatives (Fig. 4e–g and Supplementary Fig. 18c–e). Thus, MIRACLE supports the continual construction of cross-tissue atlases, yielding results consistent with offline construction and approaching the gold standard.

### Label transfer for cross-tissue mosaic data

Beyond atlas construction, MIRACLE supports label transfer: once the reference atlas is built, labels are transferred to a query set for rapid analysis by matching the query to the reference through continual integration. To avoid cross-contamination, we transferred labels independently for each query set against the initial reference atlas in both within- and cross-tissue scenarios. We compared MIRACLE with MIRACLE-generalization and MIRACLE-transfer, which match via generalization^11,13,14,34,35^ and fine-tuning^12,15^, respectively; for each, labels were transferred by a *k*-nearest neighbor (*k*NN) classifier with novelty detection that flags new query cell types as ‘Unknown’ (Methods).

**Fig. 5:**
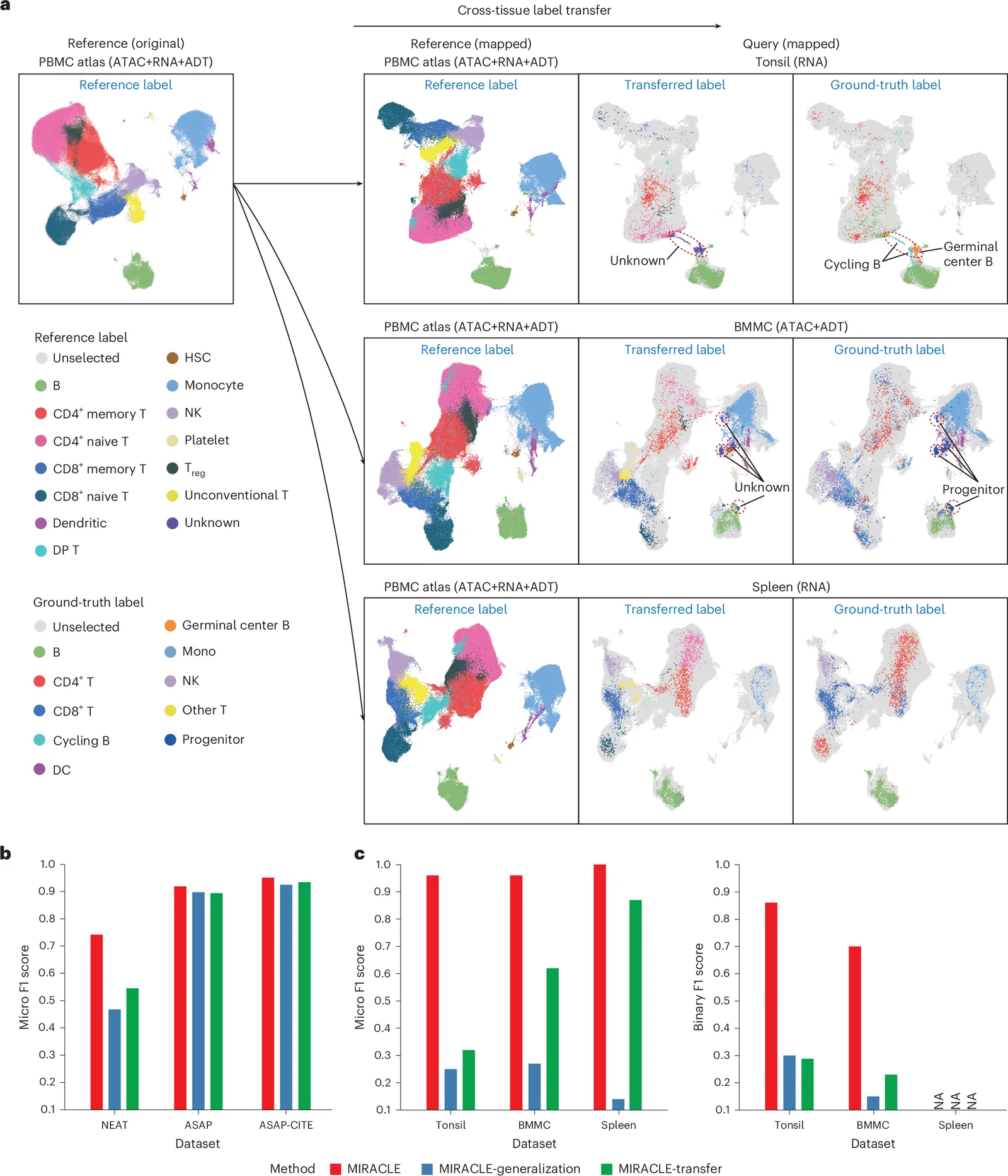
Evaluation of MIRACLE in within- and cross-tissue label transfer. **a**, UMAP visualization of MIRACLE-inferred cell embeddings for cross-tissue label transfer. Column 1: original PBMC reference colored by reference labels. Column 2: mapped PBMC references after separately incorporating different cross-tissue queries, colored by reference labels. Columns 3 and 4: the three mapped cross-tissue queries, colored by transferred labels (column 3) and third-party ground-truth labels (column 4). **b**, Micro F1 scores comparing MIRACLE and two other strategies for within-tissue label transfer. **c**, Micro F1 scores (left) and binary F1 scores (right) comparing MIRACLE and two other strategies for cross-tissue label transfer. NA, not applicable. Source data

MIRACLE’s transferred labels were highly consistent with the query set’s ground-truth labels (Fig. 5a, Supplementary Fig. 19 and Methods), and in the cross-tissue scenario the cells flagged as ‘Unknown’ corresponded precisely to the new query cell types (‘Cycling B’ and ‘Germinal center B’ cells in tonsil, ‘Progenitor’ cells in bone marrow; Fig. 5a). In contrast, MIRACLE-generalization and MIRACLE-transfer showed pronounced batch effects and label inconsistency (Supplementary Figs. 20 and 21) and struggled to recognize new cross-tissue cell types (Supplementary Fig. 21a,b).

Quantitatively, in the within-tissue scenario, MIRACLE’s label-transfer consistency (Supplementary Fig. 22) and micro F1 (Fig. 5b) exceeded both alternatives, notably on the NEAT query set, which contained only CD4^+^ T cells and was harder to match against a multitype reference. In the cross-tissue scenario, MIRACLE’s consistency (Supplementary Fig. 23) and its micro and binary F1 scores for new cell-type identification (Fig. 5c) were substantially higher than the other strategies. MIRACLE thus achieves accurate, robust label transfer and new cell-type identification across batches, modalities and tissues.

### Continual integration of respiratory infection data

The rapid evolution of respiratory pathogens demands continuous data integration for a timely public health response. Using MIRACLE, we incrementally integrated heterogeneous datasets from three major respiratory infections with distinct immune dynamics. Beginning with a healthy PBMC reference atlas (34 batches; ATAC+RNA+ADT), we sequentially added PBMC datasets from COVID-19 (10 batches; RNA+ADT)^36^, flu A (7 batches; RNA)^37^ and TB (20 batches; RNA+ADT)^38^ (Supplementary Table 11), simulating real-world data accumulation while enabling cross-pathogen immune analyses.

**Fig. 6:**
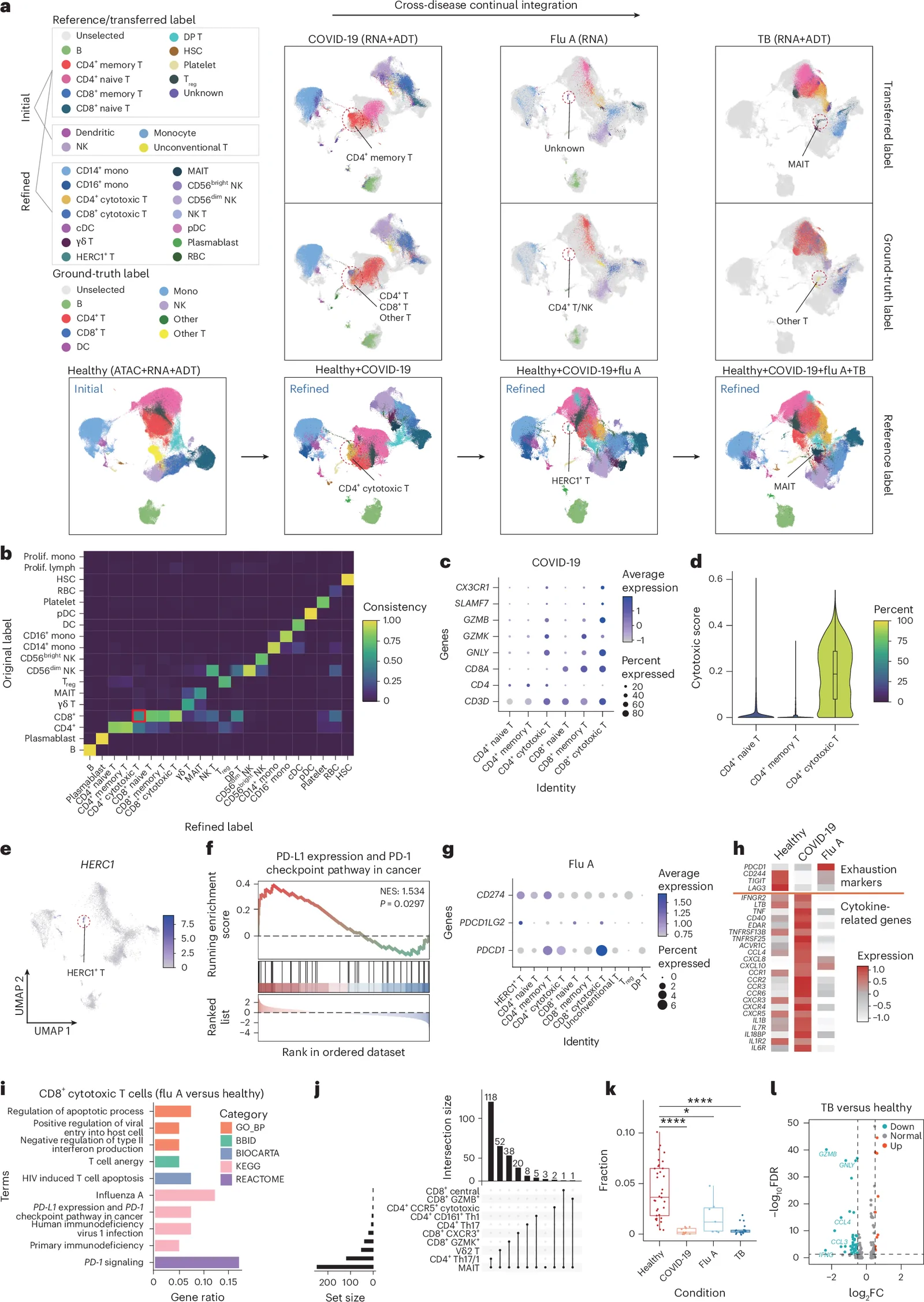
Application of MIRACLE to continual atlas construction across respiratory infections. **a**, UMAP of cross-disease atlas construction. Column 1: healthy PBMC atlas; columns 2–4: per disease, the integrated dataset with transferred and ground-truth labels, and the updated atlas with refined labels (red circles, major cell-type updates). **b**, Confusion plot showing original versus refined labels (COVID-19). **c**, Marker-gene dot plot for COVID-19 T cells. **d**, Cytotoxic scores in CD4^+^ naive, memory and cytotoxic T cells (COVID-19; *n* = 9,822, 2,391, 5,231 cells). **e,** *HERC1* expression (UMAP), integrated flu A dataset. **f**, GSEA for the ‘PD-L1 expression and PD-1 checkpoint pathway in cancer’ gene set in HERC1^+^ T cells (flu A; *P* = 0.0297); NES, normalized enrichment score. **g**, Expression of *PDCD1* (PD-1), *CD274* (PD-L1) and *PDCD1LG2* (PD-L2) in flu A T cells. **h**, Exhaustion markers and cytokines in CD8^+^ cytotoxic T cells across healthy, COVID-19 and flu A. **i**, Exhaustion-related term enrichment for flu A versus healthy CD8^+^ cytotoxic T cells. **j**, UpSet plot of cells reclassified as MAIT from each original label. **k**, MAIT cell fractions within non-naive T cells in healthy individuals and those with COVID-19, flu A and TB (*n* = 34, 10, 7, 20); two-sided Wilcoxon rank-sum test versus healthy (*P* = 5.7 × 10^−6^, 0.015 and 1.3 × 10^−8^ for COVID-19, flu A and TB). **l**, Volcano plot of differentially expressed genes in MAIT cells (TB versus healthy); FC, fold change; FDR, false discovery rate. In the box plots in **d** and **k**, the box shows the 25th to 75th percentiles, the center marks the median and the whiskers show 1.5 × interquartile range; *n* is the number of cells (**d**) or individuals (**k**). RBC, red blood cell; prolif., proliferating. Source data

Following integration, MIRACLE expanded the initial PBMC atlas with effective alignment across batches, modalities and diseases, enabling the manual refinement from 13 to 23 cell types (Fig. 6a).

COVID-19 integration resolved a classification discrepancy: label transfer reclassified a subset of CD8^+^ T cells from the original study^36^ (Supplementary Fig. 24a) as CD4^+^ T cells (Fig. 6a). Multimodal validation identified these as CD4^+^ cytotoxic T lymphocytes (CTLs; Fig. 6a,b), expressing CD4^+^ T cell markers (*CD3D* and *CD4*) and surface proteins together with cytotoxic effectors (*GZMB*, *GZMK* and *GNLY*; Fig. 6c and Supplementary Fig. 24b,c). They showed higher cytotoxic activity than conventional CD4^+^ T cells (Fig. 6d) and were enriched for cytotoxic signatures (Supplementary Fig. 24d), with selective expansion in moderate cases (Supplementary Fig. 24e), consistent with known CD4^+^ CTL involvement in viral pathogenesis^39,40^.

Flu A integration identified a HERC1^+^ T cell subset with high *HERC1* expression (Fig. 6a,e and Supplementary Fig. 25a–d). In patients with flu A, this subset showed programmed cell death protein 1 (PD-1) pathway enrichment (*P* = 0.0297; Fig. 6f and Supplementary Fig. 25e) and elevated *CD274* (PD-L1) and *PDCD1LG2* (PD-L2), while CD8^+^ CTLs showed increased *PDCD1* (PD-1) (Fig. 6g)—a pattern absent in healthy individuals and those with COVID-19 (Supplementary Fig. 25f,g). CD8^+^ CTLs in flu A also displayed stronger exhaustion and anergy/apoptosis signatures than in healthy individuals and those with COVID-19 (Fig. 6h,i and Supplementary Fig. 25h). These findings suggest that HERC1^+^ T cells may contribute to CD8^+^ T cell exhaustion via the PD-1/programmed cell death ligand (PD-L)1/PD-L2 axis, identifying a previously unrecognized subset that warrants further investigation (Supplementary Section 1).

TB integration reclassified a subset of CD4^+^ Th17/1, Vδ2 and CD8^+^ GZMK^+^ T cells from the original study^38^ as mucosal-associated invariant T (MAIT) cells (Fig. 6a,j and Supplementary Fig. 26a), on the basis of *SLC4A10* and TCR Vα7.2 expression (Supplementary Fig. 26b–e). MAIT cells, known for antimicrobial immunity, were significantly depleted in all three infections relative to healthy individuals (Fig. 6k), consistent with reported peripheral MAIT redistribution during infection^41,42^. TB and COVID-19 MAIT cells showed reduced cytotoxic gene expression (*GZMB*, *GNLY* and *IFNG* in TB; *GZMB*, *PRF1* and *NKG7* in COVID-19; Fig. 6l and Supplementary Fig. 26f), suggesting impaired antimicrobial function, whereas flu A MAIT cells were relatively unaffected (Supplementary Fig. 26g).

In summary, MIRACLE’s sequential integration maintains consistent annotations, resolves ambiguous subsets through multimodal validation and reveals both shared and pathogen-specific immune mechanisms, with refined cell types robust to integration order (Supplementary Fig. 27). The expanded CD4^+^ CTLs in COVID-19 and putatively exhaustion-associated HERC1^+^ T cells in flu A—both reproducible in independent analyses (Supplementary Section 2)—together with impaired MAIT functionality across infections underscore the translational value of continual integration for respiratory immunity research.

## Discussion

The continuous generation of single-cell sequencing data across diverse platforms and biological contexts necessitates continual integration methods that can incrementally refine and expand single-cell atlases. Conventional methods require full reintegration of all available data upon each update, imposing substantial computational costs and limiting scalability. MIRACLE addresses this challenge by enabling efficient and memory-constrained continual integration of various multimodal single-cell datasets, leveraging CL strategies and deep generative models to maintain biological consistency while mitigating catastrophic forgetting.

Our results show that, through dynamic architecture adaptation and data rehearsal, MIRACLE’s integration quality generally matches its offline counterpart—typically the upper benchmark—while substantially reducing computational overhead; in some complex scenarios, the online process even performs slightly better (Fig. 3b and Supplementary Fig. 4b), which we attribute to an implicit ‘curriculum learning’ effect that may help the model avoid local optima. MIRACLE incorporates new cross-modal, cross-tissue and cross-disease data while preserving prior knowledge, outperforming existing online methods in accuracy and robustness. It refined a large-scale PBMC trimodal atlas with multi-tissue datasets (tonsil, BMMC and spleen), maintaining annotation consistency and enabling precise cross-tissue label transfer, and on respiratory infection datasets (COVID-19, flu A and TB) it facilitated the discovery of shared and pathogen-specific immune mechanisms.

Beyond its immediate applications, MIRACLE introduces a scalable paradigm for iterative single-cell analysis. Unlike traditional methods that require computationally expensive retraining, MIRACLE enables resource-efficient updates, making continual integration feasible even for laboratories with limited computational infrastructure. This democratization of single-cell data integration fosters broader collaboration, accelerating discoveries in biological mechanisms, disease pathology and medical applications.

Looking forward, MIRACLE’s CL framework and mosaic integration capability provide a foundation for incorporating additional data types, such as methylomics and metabolomics. This is particularly relevant for the rapidly evolving field of spatial omics, where spatial information could be incorporated by treating coordinates as model inputs or, more explicitly, via graph neural networks and spatial regularization to model tissue architecture and correct artifacts. A key future direction is to use MIRACLE as a universal online wrapper for offline integration models, extending continual learning to established multi-omic models^7,9,12,13,15,35^ and to those incorporating spatial context^43–47^. Other directions include automating dynamic architecture adaptation to incoming data complexity^48,49^, adopting order-robust CL techniques to stabilize long, complex integration sequences^50,51^ and integrating federated learning for collaborative updates that preserve data privacy^52,53^, further enhancing MIRACLE’s scalability and applicability.

In summary, MIRACLE provides a robust, efficient and scalable solution for the continual integration of multimodal single-cell data, addressing key challenges in data adaptation and knowledge retention and paving the way for dynamic, shared and collaborative single-cell analysis.

## Methods

### Single-cell continual integration with CL

#### The scenario of single-cell continual integration

Single-cell continual integration aims to continuously incorporate new data into the atlas. Let ${{\mathcal{X}}}_{t}={\{{{\mathbf{x}}}_{t,n}\}}_{n = 1}^{{N}_{t}}$ be the batch of *N*_*t*_ cells arriving at time *t*, with **x**_*t*,*n*_ the observation of cell *n*, and let ${{\mathcal{Y}}}_{t}={\{{{\mathbf{y}}}_{t,n}\}}_{n = 1}^{{N}_{t}}$ be its integration results, where **y**_*t*,*n*_ may include the low-dimensional embedding or batch-corrected data of cell *n*. The goal is to update all results ${{\mathcal{Y}}}_{1:t}=\mathop{\bigcup }\nolimits_{\tau = 1}^{t}{{\mathcal{Y}}}_{\tau }$ whenever new data ${{\mathcal{X}}}_{t}$ arrives.

#### Offline strategy

To achieve continual integration, the common approach is an offline strategy^6–9^

1a $${{\mathbf{\uptheta}}}_{t}={f}_{{\rm{off}}}({{\mathcal{X}}}_{1:t})$$

1b $${{\mathcal{Y}}}_{1:t}=g({{\mathcal{X}}}_{1:t};{{\mathbf{\uptheta}}}_{t})$$

Whenever new data ${{\mathcal{X}}}_{t}$ arrives, all data ${{\mathcal{X}}}_{1:t}=\mathop{\bigcup }\nolimits_{\tau = 1}^{t}{{\mathcal{X}}}_{\tau }$ are used to train parameters **θ**_*t*_ via the offline function *f*_off_, and the inference function *g* predicts the results ${{\mathcal{Y}}}_{1:t}$. Although effective, retraining on all data each time becomes increasingly inefficient.

#### Online strategy 1 using generalization

To avoid repeated retraining, online strategies can be adopted. One efficient approach is generalization^11,13,14^

2a $${{\mathbf{\uptheta}}}_{0}={f}_{{\rm{off}}}({{\mathcal{X}}}_{0})$$

2b $${{\mathcal{Y}}}_{t}=g({{\mathcal{X}}}_{t};{{\mathbf{\uptheta}}}_{0})$$

The model is first pretrained offline on large external datasets ${{\mathcal{X}}}_{0}$ to obtain fixed parameters **θ**_0_, which are then generalized to new data by inferring ${{\mathcal{Y}}}_{t}$ from each ${{\mathcal{X}}}_{t}$ via *g*, so past results need no update. This avoids training during integration but relies on external datasets that often fail to capture unknown biological and technical variations in new data, giving poor adaptability.

#### Online strategy 2 using fine-tuning

To adapt to unknown changes in new data, online strategies based on fine-tuning have emerged^10,12,15^

3a $${{\mathbf{\uptheta}}}_{t}={f}_{{\rm{FT}}}({{\mathcal{X}}}_{t};{{\mathbf{\uptheta}}}_{t-1})$$

3b $${{\mathcal{Y}}}_{1:t}=g({{\mathcal{X}}}_{1:t};{{\mathbf{\uptheta}}}_{t})$$

At step *t*, *f*_FT_ fine-tunes the previous parameters **θ**_*t*−1_ on new data ${{\mathcal{X}}}_{t}$ to obtain **θ**_*t*_, which *g* uses to predict ${{\mathcal{Y}}}_{1:t}$ (**θ**_0_ is pretrained or randomly initialized). However, as *t* grows, **θ**_*t*_ gradually forgets past information, degrading results, especially for earlier data.

#### Online strategy 3 (ours) using CL

To adapt to new data while preventing forgetting, we propose an online integration strategy based on CL^17,18^

4a $${{\mathbf{\uptheta}}}_{t}={f}_{{\rm{CL}}}({{\mathcal{X}}}_{t},{{\mathcal{R}}}_{t-1};{{\mathbf{\uptheta}}}_{t-1})$$

4b $${{\mathcal{R}}}_{t}=h({{\mathcal{X}}}_{t},{{\mathcal{R}}}_{t-1};{{\mathbf{\uptheta}}}_{t})$$

4c $${{\mathcal{Y}}}_{1:t}=g({{\mathcal{X}}}_{1:t};{{\mathbf{\uptheta}}}_{t})$$

Unlike fine-tuning, CL introduces a rehearsal memory ${{\mathcal{R}}}_{t}\subseteq {{\mathcal{X}}}_{1:t}$ that retains key data to prevent forgetting. Combining new data ${{\mathcal{X}}}_{t}$ with the previous memory ${{\mathcal{R}}}_{t-1}$, *f*_CL_ updates **θ**_*t*−1_ to **θ**_*t*_; the memory is then updated by the sampling function *h*, and predictions ${{\mathcal{Y}}}_{1:t}$ are made by *g*. Initially, ${{\mathcal{R}}}_{0}={\emptyset}$ and **θ**_0_ is pretrained or randomly initialized.

### Dynamic architecture adaptation

In continual integration, new data ${{\mathcal{X}}}_{t}$ often originate from various sequencing technologies and may introduce new modalities or features. To seamlessly incorporate these, we propose dynamically expanding the model architecture in the parameter update equation (equation (4a)). Let ${\tilde{{\mathbf{\uptheta}}}}_{t}$ be the model parameters associated with the new modalities or features. We randomly initialize ${\tilde{{\mathbf{\uptheta}}}}_{t}$ and combine it with the previous parameters **θ**_*t*−1_ to get the initial value ${\hat{{\mathbf{\uptheta}}}}_{t}$:

5 $${\hat{{\mathbf{\uptheta}}}}_{t}=\left[{{\mathbf{\uptheta}}}_{t-1},{\tilde{{\mathbf{\uptheta}}}}_{t}\right]$$

On the basis of ${\hat{{\mathbf{\uptheta}}}}_{t}$, we can then obtain **θ**_*t*_ by training on ${{\mathcal{X}}}_{t}$ and ${{\mathcal{R}}}_{t-1}$.

### Distribution-preserving reservoir sampling

In equation (4b), the rehearsal memory ${{\mathcal{R}}}_{t}$ grows with more iterations, harming efficiency and knowledge sharing. To bound it, we propose a batched reservoir sampling algorithm that limits the capacity of ${{\mathcal{R}}}_{t}$ when a new batch ${{\mathcal{X}}}_{t}$ arrives. To keep integration unbiased, we further combine stratified sampling to preserve the interbatch distribution with a ball tree sampling method to preserve the intrabatch distribution. We call this combined scheme DPRS.

#### Batched reservoir sampling for batch data

Classical reservoir sampling gives each cell an equal probability of being retained under a fixed capacity but handles only one new cell at a time. To handle new batches with many cells, we propose batched reservoir sampling.

Assume that the rehearsal memory has a capacity limit of *M* cells. After each integration, we add new data ${{\mathcal{X}}}_{t}$ of size *N*_*t*_ to the previous rehearsal memory ${{\mathcal{R}}}_{t-1}$ of size *M*_*t*−1_, resulting in ${{\mathcal{R}}}_{t}$. However, if *N*_*t*_ + *M*_*t*−1_ > *M*, we first sample ${{\mathcal{R}}}_{t-1}$ and ${{\mathcal{X}}}_{t}$ with sampling ratios *α* and *β*, respectively, where *α* and *β* satisfy

6 $$\left\{\begin{array}{ll}\alpha {M}_{t-1}+\beta {N}_{t}=M\\\alpha {M}_{t-1}:\beta {N}_{t}={S}_{t-1}:{N}_{t}\end{array}\right.$$

where *S*_*τ*_ = *N*_1_ + *N*_2_ + ⋯ + *N*_*τ*_ is the total number of cells in the first *τ* batches. Equation (6) ensures that ${{\mathcal{R}}}_{t}$ has size *M*, with new and old data proportions consistent with their original batches. From equation (6), we get *α* = *S*_*t*−1_*M*/(*S*_*t*_*M*_*t*−1_) and *β* = *M*/*S*_*t*_. Note that, when *N*_*t*_ = 1, batched reservoir sampling reduces to classical reservoir sampling.

#### Stratified sampling for interbatch distribution

To maintain the cell distribution across batches, in addition to batched reservoir sampling, we further introduce batch-based stratified sampling on past data. Specifically, in memory ${{\mathcal{R}}}_{t-1}$, assuming that ${{\mathcal{R}}}_{t-1}^{(\tau )}$ is the data from the *τ*th batch and is of size ${M}_{t-1}^{(\tau )}$, we sample each ${{\mathcal{R}}}_{t-1}^{(\tau )}$ with a ratio of *α* for *τ* = 1, 2, …, *t* − 1. This ensures that the cell proportions in ${{\mathcal{R}}}_{t}$ match those in the original batches.

#### Ball tree sampling for intrabatch distribution

Within a batch, the common SRS poorly preserves the data distribution at low sampling ratios^54^; we therefore propose a ball tree sampling algorithm.

Assume that the data before sampling are generated from the underlying distribution *p*(**x**). We divide the data space ${\mathcal{D}}$ into infinitesimal intervals ${\{{{\mathcal{D}}}_{i}\}}_{i = 1}^{\infty }$, each with a Lebesgue measure $\lambda ({{\mathcal{D}}}_{i})$ close to 0. Then, for any **x**, assuming it lies in the *j*(**x**)th interval, *p*(**x**) can be approximated as

7 $$\begin{array}{r}p({\mathbf{x}})\approx \frac{P({\mathbf{x}}\in {{\mathcal{D}}}_{j({\mathbf{x}})})}{\lambda ({{\mathcal{D}}}_{j({\mathbf{x}})})}\approx \frac{{N}_{j({\mathbf{x}})}}{N\lambda ({{\mathcal{D}}}_{j({\mathbf{x}})})}\end{array}$$

where *N* is the total number of cells to be sampled, *N*_*j*(**x**)_ is the number of cells in ${{\mathcal{D}}}_{j({\mathbf{x}})}$ and *P*(·) denotes probability. Similarly, after sampling with ratio *γ*, the underlying distribution $\tilde{p}({\mathbf{x}})$ is approximated as

8 $$\tilde{p}({\mathbf{x}})\approx \frac{\tilde{P}({\mathbf{x}}\in {{\mathcal{D}}}_{j({\mathbf{x}})})}{\lambda ({{\mathcal{D}}}_{j({\mathbf{x}})})}\approx \frac{{\tilde{N}}_{j({\mathbf{x}})}}{\gamma N\lambda ({{\mathcal{D}}}_{j({\mathbf{x}})})}$$

where *γN* is the total number of cells after sampling, and ${\tilde{N}}_{j({\mathbf{x}})}$ is the number of cells in ${{\mathcal{D}}}_{j({\mathbf{x}})}$ after sampling. To ensure the sampled distribution matches the original, $\tilde{p}({\mathbf{x}})=p({\mathbf{x}})$ is required. Combining equations (7) and (8), we get ${\tilde{N}}_{j({\mathbf{x}})}\approx \gamma {N}_{j({\mathbf{x}})}$, which means that, for any interval ${{\mathcal{D}}}_{i}$, we should sample at a ratio of *γ*.

Partitioning ${\mathcal{D}}$ by equal intervals (equal $\lambda ({{\mathcal{D}}}_{i})$) handles nonuniform data poorly—lacking detail in dense regions and missing sparse ones—and the number of intervals grows exponentially with dimensionality (the ‘curse of dimensionality’). We instead use equal-frequency partitioning, with the same number of cells *N*_*i*_ per interval, which adapts interval size to local density and grows only linearly with the total sample size *N*.

For fine-grained preservation, we set *N*_*i*_ = 1/*γ* (for example, *N*_*i*_ = 3 when *γ* = 1/3) and draw one sample per interval. Because 1/*γ* is not always an integer, we partition at multiple resolutions and select samples across resolutions to achieve any ratio. For the partitioning, we use the ball tree^55^, which handles nonuniform, high-dimensional data better than the *k*-dimensional tree. We term this intrabatch algorithm BTS (see Supplementary Information section 3 for details).

### Mosaic data handling

A key challenge in continual integration is mosaic data, where batches contain varying modality combinations. MIRACLE addresses this as a general CL framework wrapped around a base model that processes the mosaic structure at each incremental step. We selected MIDAS as the base model for its effectiveness in static mosaic integration^56^: MIDAS is a multimodal VAE that uses self-supervised learning to align modalities into a shared embedding space and information-theoretic principles to disentangle the biological cell state from technical noise, performing dimensionality reduction, batch correction and imputation in a single, offline training pass. MIRACLE extends MIDAS to online, incremental atlas construction through two mechanisms: (1) a dynamic architecture adaptation mechanism, to incorporate new modalities or features from incoming data, and (2) a data rehearsal mechanism, based on our DPRS approach, to mitigate catastrophic forgetting in a memory-efficient manner.

Suppose that, at time *t*, the set of modalities present in the observed data ${{\mathcal{X}}}_{1:t}$ is ${\mathcal{M}}$, for example, ${\mathcal{M}}=\{\,\mathrm{ATAC},\mathrm{RNA},\mathrm{ADT}\,\}$. For the *n*th cell observation **x**_*τ*,*n*_ in batch *τ* ∈ {1, …, *t*}, we denote it as

9 $${{\mathbf{x}}}_{\tau ,n}=\left\{{\{{{\mathbf{o}}}_{\tau ,n}^{m}\}}_{m\in {{\mathcal{M}}}_{\tau }},{s}_{\tau ,n}\right\}$$

where ${{\mathcal{M}}}_{\tau }\subseteq {\mathcal{M}}$ is the set of modalities in batch *τ*, ${{\mathbf{o}}}_{\tau ,n}^{m}\in {{\mathbb{N}}}^{{D}_{\tau ,n}^{m}}$ is the observation vector of modality *m* with size ${D}_{\tau ,n}^{m}$ and *s*_*τ*,*n*_ = *τ* is the batch ID.

Using the CL framework of MIRACLE with MIDAS as the base model, we obtain the integration result **y**_*τ*,*n*_ for each cell observation **x**_*τ*,*n*_

10 $${{\mathbf{y}}}_{\tau ,n}=\left\{{\{{\hat{{\mathbf{o}}}}_{\tau ,n}^{m}\}}_{m\in {\mathcal{M}}},{{\mathbf{z}}}_{\tau ,n}\right\}$$

where ${\hat{{\mathbf{o}}}}_{\tau ,n}^{m}\in {{\mathbb{N}}}^{{D}_{t}^{m}}$, with size ${D}_{t}^{m}$, is the imputed and batch-corrected count vector for modality *m* and ${{\mathbf{z}}}_{\tau ,n}\in {{\mathbb{R}}}^{{D}^{z}}$ is the batch-corrected low-dimensional cell embedding that represents the biological state of the cell.

### MIRACLE training

MIRACLE uses MIDAS as the base model to handle single-cell multimodal data with diverse modality combinations. During the *t*th integration step, the model’s parameters **θ**_*t*_ are composed of those for the encoders (${{\mathbf{\uptheta}}}_{t}^{\,\mathrm{enc}\,}$), decoders (${{\mathbf{\uptheta}}}_{t}^{\,\mathrm{dec}\,}$) and classifiers (${{\mathbf{\uptheta}}}_{t}^{\,\mathrm{cls}\,}$). The optimization of these parameters is achieved by iteratively minimizing two loss functions inherited from the MIDAS base model: the encoder–decoder loss ${l}^{f,g}({{\mathbf{\uptheta}}}_{t}^{\,\mathrm{enc}\,},{{\mathbf{\uptheta}}}_{t}^{\,\mathrm{dec}\,};{{\mathbf{x}}}_{\tau ,n},{{\mathbf{\uptheta}}}_{t}^{\,\mathrm{cls}\,})$ and the classifier loss ${l}^{r}({{\mathbf{\uptheta}}}_{t}^{\,\mathrm{cls}\,};{{\mathbf{x}}}_{\tau ,n},{{\mathbf{\uptheta}}}_{t}^{\,\mathrm{enc}\,})$. In contrast to MIDAS, this process in MIRACLE is conducted over both new data ${{\mathcal{X}}}_{t}$ and rehearsal data ${{\mathcal{R}}}_{t-1}$, where ${{\mathbf{x}}}_{\tau ,n}\in {{\mathcal{X}}}_{t}$ for *τ* = *t* and ${{\mathbf{x}}}_{\tau ,n}\in {{\mathcal{R}}}_{t-1}^{(\tau )}$ for 1 ≤*τ* < *t*. The complete MIRACLE training process, corresponding to equation (4a), is presented in Supplementary Information section 4, with the constituent loss functions detailed in Supplementary Information section 5.

### Integration of multiple batches at once

In practical applications, new data often arrive in multiple batches, denoted as $\{{{\mathcal{X}}}_{t},{{\mathcal{X}}}_{t+1},\ldots \}$, which may also consist of mosaic data. In this case, integrating these data batches simultaneously, rather than sequentially, can enhance the efficiency of continuous integration. Leveraging MIDAS’s capability to support mosaic data, MIRACLE can be seamlessly extended to accommodate this scenario.

### Datasets

All datasets used in this study are publicly available; their per-batch protocols, accessions and filtered cell numbers are detailed in Supplementary Table 12. We group them below by their role in our experiments and, for each, downloaded count matrices for gene unique molecular identifiers (UMIs), ATAC fragments and ADTs as applicable.

#### Large single-modality datasets

Two large RNA sequencing datasets were used to benchmark distribution-preserving sampling and scalability. DHCM^23^ is an snRNA-seq dataset of human dilated and hypertrophic cardiomyopathy (523,369 cells across 42 batches; Broad Single Cell Portal SCP1303). HLCA^4^ is a single-cell RNA sequencing (scRNA-seq) dataset of human lung (2.3 million cells from 48 studies, each treated as one batch), known for strong batch effects^57^.

#### Multimodal PBMC datasets

Ten human PBMC datasets spanning diverse modality combinations were used for bimodal, mosaic and atlas-level integration: DOGMA^29^ (DOGMA-seq; ATAC+RNA+ADT; four batches; GEO GSE166188), TEA^30^ (TEA-seq; ATAC+RNA+ADT; five batches; GEO GSE158013), TEA Multiome^30^ (10x Multiome; ATAC+RNA; two batches; GEO GSE158013), 10X Multiome^58–61^ (10x Multiome; ATAC+RNA; four batches^62^), WNN^28^ (CITE-seq; RNA+ADT; eight batches collected before HIV-vaccine administration^63^), DOGMA+CITE^64^ (one CITE-seq batch with RNA+ADT and three DOGMA-seq batches with ATAC+RNA+ADT; GEO GSE200417), ISSAAC^65^ (ISSAAC-seq; ATAC+RNA; one batch; ArrayExpress E-MTAB-11264), NEAT^31^ (NEAT-seq; ATAC+RNA; two batches; GEO GSE178707), ASAP^29^ (ASAP-seq; ATAC+ADT; two batches; GEO GSE156473) and ASAP-CITE^29^ (CITE-seq; RNA+ADT; two batches; GEO GSE156473). Subsets of these datasets were combined to form the DOTEA mosaic dataset and the PBMC reference atlas (Supplementary Tables 9 and 11).

#### Cross-tissue datasets

Three human immune tissues were used to assess cross-tissue integration: tonsil^32^ (RNA; one batch; GEO GSE165860), the BMMC^29^ (ASAP-seq; ATAC+ADT; one batch; GEO GSE156477) and spleen^33^ (RNA; one batch; GEO GSE159929).

#### Respiratory infection datasets

Three PBMC datasets from respiratory infections were used for cross-disease integration. COVID-19^36^ (CITE-seq; RNA+ADT; ArrayExpress E-MTAB-10026) spans severity levels (asymptomatic, mild, moderate, severe and critical); after quality control, we kept the two largest batches per level (ten batches total). Flu A^37^ (scRNA-seq whole blood; GEO GSE243629) provided seven flu A virus-infected batches (adults, pregnant women and children), from which we retained only PBMC cell types. TB^38^ (CITE-seq; RNA+ADT; GEO GSE158769) comprises memory T cells from active or latent infection; after quality control, we kept the 10 largest batches per type (20 batches total).

### Data preprocessing

For multimodal data spanning multiple batches, we performed quality control and feature selection per batch and then unified features across batches, using the Seurat package (version 4.1.0)^28^ for the RNA and ADT count matrices.

For RNA, we filtered low-quality cells by the number of detected genes per cell, total UMI count and mitochondrial read percentage, normalized and log-transformed the counts with NormalizeData, removed low-frequency genes and selected the top 4,000 highly variable genes per batch with FindVariableFeatures; their union via SelectionIntegrationFeatures yielded 4,000 final features.

For ADT, we excluded low-quality cells by total protein tag count, applied centered log-ratio normalization with NormalizeData and retained all ADT features without further selection.

For ATAC fragments, we used the Signac package (version 1.6.0)^66^ with MACS2 peak calling^67^, performed quality control by transcription start site enrichment score and nucleosome signal, merged peaks with Signac’s reduce function and recounted fragments within the merged peaks. RNA UMI counts, ADT tag counts and binarized ATAC fragment counts served as model inputs.

Newly arrived data followed the same procedure; to prevent excessive expansion of ATAC features during continual integration, we aligned peaks with those from previous batches.

### Third-party cell-type labels

To comprehensively evaluate PBMC datasets both qualitatively and quantitatively, we used the third-party tool Seurat for cell type annotation via label transfer. The reference dataset was the CITE-seq PBMC atlas from ref. ^28^. Label transfer was performed using Seurat’s FindTransferAnchors and TransferData functions with the ‘cca’ reduction method for reference mapping. When raw RNA expression data were unavailable, we generated a gene activity matrix from ATAC data using Signac’s GeneActivity function, which was then used for label transfer. However, owing to the predominance of CD4^+^ T cells in the NEAT dataset, finding a suitable reference for accurate annotation was challenging. Therefore, after the initial annotation using Seurat’s label transfer, we applied its FindClusters function to further cluster the data and refine the results. For the flu A dataset, originally derived from whole blood, we performed clustering in Seurat, integrated Seurat’s label transfer results with SingleR^68^ annotations for manual refinement, and retained only PBMC-associated cell types.

For the tonsil dataset, we used Azimuth (https://azimuth.hubmapconsortium.org), a reference-based single-cell analysis tool, to automate cell annotation on the basis of gene expression data. We then manually refined the annotations by integrating Seurat’s clustering results. Similarly, for the BMMC dataset, we employed Azimuth’s ATAC application for ATAC-based cell annotation. For the spleen dataset, we used SingleR for unbiased scRNA-seq annotation, followed by manual refinement using Seurat clustering.

### Sampling methods for distribution preservation

To evaluate the distribution preservation capability of our BTS algorithm, we compared its performance with SRS, Sketch, and scSampler. Because DPRS samples in the embedding space, for each comparison we used MIRACLE to convert the dataset into low-dimensional embeddings, randomly selected 10,000 cells as the reference sample and repeated the experiment 50 times across different sampling ratios.

#### SRS

We used the subsample function in the Scanpy package^69^ to perform SRS. To ensure diverse sampling results, we set different random seeds for each run of the experiment.

#### Sketch

The Sketch method^24^ is designed to preserve rare populations during sampling. We utilized the SketchData function in the Seurat package (version 5.0.0) for sample selection^70^.

#### scSampler

The python package scSampler^25^ is also designed to preserve rare populations and can be downloaded from ref. ^71^. Since our input data were already low-dimensional embeddings, we skipped the default reduction process.

#### Evaluation of distribution preservation

We used the MMD metric to evaluate the consistency of data distributions before and after sampling. A smaller MMD value indicates better sampling performance. Given two sets of samples ${\mathcal{X}}=\{{{\mathbf{x}}}_{1},\ldots ,{{\mathbf{x}}}_{{N}_{1}}\}$ and ${{\mathcal{X}}}^{{\prime} }=\{{{\mathbf{x}}}_{1}^{{\prime} },\ldots ,{{\mathbf{x}}}_{{N}_{2}}^{{\prime} }\}$, MMD is defined as

11 $$\mathrm{MMD}\,({\mathcal{X}},{{\mathcal{X}}}^{{\prime} })=\frac{1}{{N}_{1}^{2}}\mathop{\sum }\limits_{i=1}^{{N}_{1}}\mathop{\sum }\limits_{j=1}^{{N}_{1}}k({{\mathbf{x}}}_{i},{{\mathbf{x}}}_{j})+\frac{1}{{N}_{2}^{2}}\mathop{\sum }\limits_{i=1}^{{N}_{2}}\mathop{\sum }\limits_{j=1}^{{N}_{2}}k({{\mathbf{x}}}_{i}^{{\prime} },{{\mathbf{x}}}_{j}^{{\prime} })-\frac{2}{{N}_{1}{N}_{2}}\mathop{\sum }\limits_{i=1}^{{N}_{1}}\mathop{\sum }\limits_{j=1}^{{N}_{2}}k({{\mathbf{x}}}_{i},{{\mathbf{x}}}_{j}^{{\prime} })$$

where *k*( ⋅ , ⋅) is the Gaussian kernel function.

### Implementation of comparative integration methods

We compared horizontal, rectangular and mosaic integration methods (Supplementary Table 13) in both offline and online scenarios. In the offline scenario, all batches were available from the start; in the online scenario, batches arrived incrementally. For methods that originally lacked online support, we added it through CL or fine-tuning-based transfer learning. Below, each method is described with its repository and key settings, with minor offline/online variants grouped under their base method.

#### Horizontal integration methods

##### Geneformer

Geneformer^27,72^ is a large-scale model pretrained on 30 million cells; we averaged its per-gene embeddings to obtain cell embeddings.

##### PCA

PCA was applied after selecting highly variable genes, retaining the top 32 principal components as embeddings.

#### Rectangular integration methods

##### SCALEX

SCALEX^13,73^ is an online scRNA-/scATAC-seq method; lacking native ADT support, we concatenated RNA and ADT. SCALEX-projection trained on the first batch and projected the remaining batches (embedding dimension 32).

##### trVAE

trVAE^74,75^ supports a single modality, so we concatenated RNA and ADT. trVAE+scArches added scArches transfer learning, training a reference on the first batch and adaptors on the remaining batches.

##### Concerto

Concerto^12,76^ integrates RNA and ADT. Concerto-transfer pretrained on the first batch and fine-tuned sequentially on the remaining batches.

##### Symphony

Symphony^14,77^ handles new-data queries; we concatenated RNA and ADT, building a reference on the first batch and querying the rest. Symphony-offline instead trained on all batches together.

##### online iNMF

Online iNMF^10,78^ iteratively updates RNA models; we concatenated RNA and ADT, integrated by batch and used the final model for all embeddings. Online iNMF-offline trained all batches together.

##### CCA+WNN and RPCA+WNN

Seurat’s CCA^6^ removes batch effects in feature space (FindIntegrationAnchors with reduction ‘cca’, then IntegrateData), followed by PCA and WNN omics fusion; RPCA+WNN is identical with reduction ‘rpca’. Their ‘-continual’ variants integrate each new batch against previously corrected counts, resembling CL with unbounded rehearsal memory and thus declining in efficiency as integrations accumulate.

##### sciPENN

sciPENN^34,79^ integrates RNA and ADT using the intersection of RNA features and the union of ADT features. sciPENN-transfer trained on the initial batch and fine-tuned on subsequent ones.

#### Mosaic integration methods

##### MIRACLE

MIRACLE employs MIDAS^19,80^ as its base model, inheriting its neural network architecture and training hyperparameters. Its rehearsal memory was set to 200K cells for all bimodal and trimodal tasks—substantially above the 20K sufficient for single-modality RNA integration on DHCM—to better preserve biological fidelity in more complex multimodal data.

##### MIRACLE-offline and MIRACLE-transfer

MIRACLE-offline, the upper benchmark, trains the same base model on all batches simultaneously, avoiding catastrophic forgetting and requiring no rehearsal memory. MIRACLE-transfer, the lower benchmark, fine-tunes the same model on only the new data at each step without rehearsal, making it prone to forgetting.

##### Multigrate

Multigrate^81,82^ integrates RNA, ADT and ATAC (KL and integ set to 0.1 and 3000). Multigrate+scArches^11,83^ added scArches transfer learning, using the first batch (or the first two for the DOTEA mosaic, to cover all modalities) as reference and adaptors for the rest.

##### scVAEIT

scVAEIT^35,84^ integrates RNA, ADT and ATAC. scVAEIT-transfer (our extension) trained on the first batch and fine-tuned on subsequent ones.

##### totalVI

totalVI^7^ (ref. ^85^, incorporated into scArches) integrates RNA and ADT. totalVI+scArches built the reference on the first batch and adaptors on the rest.

### Evaluation metrics for integration

To comprehensively evaluate the integration performance of MIRACLE and various state-of-the-art methods, we used the scIB and scMIB metrics. For horizontal and rectangular integration tasks, we used the scIB metric to assess batch correction and biological conservation performance. For mosaic integration tasks, we used the scMIB metric to evaluate batch correction, modality alignment and biological conservation performance. Modality alignment is crucial for mosaic integration as it reflects the model’s capabilities in applications such as modality imputation and cross-modal label transfer. Note that, for other mosaic integration methods compared, batch correction can only be performed in the embedding space rather than the feature space, so we excluded feature space-based metrics for batch correction and biological conservation in scMIB.

Specifically, the batch correction metrics include the graph integration local inverse Simpson’s index (iLISI) *y*^iLISI^, graph connectivity *y*^gc^ and *k*NN batch effect test (*k*BET) *y*^kBET^. The modality alignment metrics include the modality averaged silhouette width (ASW) *y*^ASW^, fraction of samples closer than the true match (FOSCTTM) *y*^FOSCTTM^, label transfer F1 *y*^ltF1^, ATAC area under the receiver operating characteristic (AUROC) *y*^AUROC^, RNA Pearson’s *ry*^RNAr^ and ADT Pearson’s *ry*^ADTr^. The biological conservation metrics include the normalized MI (NMI) *y*^NMI^, adjusted Rand index (ARI) *y*^ARI^, isolated label F1 *y*^ilF1^ and graph cell-type LISI (cLISI) *y*^cLISI^. The definitions of these metrics are detailed in Supplementary Information section 6.

We average each type of metrics to obtain its comprehensive score, including the batch correction score *y*^batch^, modality alignment score *y*^mod^ and biological conservation score *y*^bio^

12 $$\begin{array}{lll}{y}^{{\rm{batch}}}&=&({y}^{{\rm{iLISI}}}+{y}^{{\rm{gc}}}+{y}^{{\rm{kBET}}})/3\\ {y}^{{\rm{mod}}}&=&({y}^{{\rm{ASW}}}+{y}^{{\rm{FOSCTTM}}}+{y}^{{\rm{ltF1}}}+{y}^{{\rm{AUROC}}}+{y}^{{\rm{RNAr}}}+{y}^{{\rm{ADTr}}})/6\\ {y}^{{\rm{bio}}}&=&({y}^{{\rm{NMI}}}+{y}^{{\rm{ARI}}}+{y}^{{\rm{ilF1}}}+{y}^{{\rm{cLISI}}})/4\end{array}$$

Following ref. ^26^, the scIB overall score *y*^scIB^ is the sum of *y*^batch^ weighted by 0.4 and *y*^bio^ weighted by 0.6

13 $${y}^{{\rm{scIB}}}=0.4\cdot {y}^{{\rm{batch}}}+0.6\cdot {y}^{{\rm{bio}}}$$

As an extension of scIB, following ref. ^19^, the scMIB overall score *y*^scMIB^ is the sum of *y*^batch^ weighted by 0.3, *y*^mod^ weighted by 0.3, and *y*^bio^ weighted by 0.4

14 $${y}^{{\rm{scMIB}}}=0.3\cdot {y}^{{\rm{batch}}}+0.3\cdot {y}^{{\rm{mod}}}+0.4\cdot {y}^{{\rm{bio}}}.$$

### CL metrics for catastrophic forgetting

To quantitatively assess MIRACLE’s ability to mitigate catastrophic forgetting, we adopted two classic metrics from the CL literature: average accuracy (ACC) and backward transfer (BWT)^86^. These metrics are established benchmarks in the field for evaluating a model’s retention of previously learned knowledge. While originally designed for classification tasks, we have adapted them for the context of single-cell data integration.

Let *T* be the total number of integration steps (that is, the total number of batches to be integrated sequentially). We define *y*_*t*,*τ*_ as the overall integration performance score (for example, the scIB or scMIB overall score) on the cumulative data from the first *τ* batches, ${{\mathcal{X}}}_{1:\tau }=\mathop{\bigcup }\nolimits_{i = 1}^{\tau }{{\mathcal{X}}}_{i}$, evaluated using the model parameters **θ**_*t*_ obtained after integrating up to batch *t* (where 1 ≤ *τ* ≤ *t* ≤ *T*).

#### ACC

ACC measures the average performance across all cumulative tasks at the end of the entire CL process, providing a summary of how well the final model performs. It is defined as

15 $$\,\mathrm{ACC}\,=\frac{1}{T}\mathop{\sum }\limits_{\tau =1}^{T}{y}_{T,\tau }$$

In this formula, *y*_*T*,*τ*_ denotes the integration performance on the first *τ* batches as evaluated by the final model, **θ**_*T*_, which has been trained on all *T* batches. A higher ACC indicates better overall performance of the final model across all historical integration stages.

#### BWT

BWT directly quantifies the influence of learning new tasks on the performance of previously learned tasks. A negative BWT is a hallmark of catastrophic forgetting, indicating that the model’s performance on older tasks has degraded after learning new ones. BWT is calculated as

16 $$\,\mathrm{BWT}\,=\frac{1}{T-1}\mathop{\sum }\limits_{\tau =1}^{T-1}({y}_{T,\tau }-{y}_{\tau ,\tau })$$

Here, *y*_*τ*,*τ*_ represents the integration performance on the first *τ* batches evaluated immediately after the *τ*th batch was integrated (that is, using model **θ**_*τ*_). This score can be considered the ‘peak’ performance for that cumulative task. The difference, *y*_*T*,*τ*_ − *y*_*τ*,*τ*_, therefore directly measures the performance drop (or gain) on an earlier task set after subsequent learning has occurred. A BWT value close to zero signifies successful knowledge retention, while a large negative value indicates substantial catastrophic forgetting.

### Reporting summary

Further information on research design is available in the Nature Portfolio Reporting Summary linked to this article.

## Supplementary information

Supplementary Information Supplementary sections 1–6, Figs. 1–31 and Tables 1–13.

Reporting Summary

Peer Review File

## Source data

Source Data Fig. 2. Statistical source data.

Source Data Fig. 3. Statistical source data.

Source Data Fig. 4. Statistical source data.

Source Data Fig. 5. Statistical source data.

Source Data Fig. 6. Statistical source data.

Source Data Extended Data Fig. 1. Statistical source data.

## Data Availability

All datasets analyzed in this study are publicly available; their accession codes are listed in “Datasets” in Methods and in Supplementary Table 12. Briefly, DHCM is available at the Broad Single Cell Portal (SCP1303) and HLCA at CELLxGENE (collection 6f6d381a-7701-4781-935c-db10d30de293); the multimodal PBMC, cross-tissue and respiratory-infection datasets are available from the Gene Expression Omnibus (GSE166188, GSE158013, GSE200417, GSE178707, GSE156473, GSE165860, GSE156477, GSE159929, GSE158769 and GSE243629) and ArrayExpress (E-MTAB-11264 and E-MTAB-10026), while the 10x Multiome and WNN datasets are available via 10x Genomics at https://www.10xgenomics.com/resources/datasets/ and the Fred Hutch atlas via the Fred Hutch Cancer Center at https://atlas.fredhutch.org/nygc/multimodal-pbmc (ref. ^63^). The processed data used in the analyses are available via Zenodo at https://doi.org/10.5281/zenodo.20746054 (ref. ^87^). Source data are provided with this paper.

## Code availability

MIRACLE is available via GitHub at https://github.com/sc-miracle/miracle and via Zenodo at https://doi.org/10.5281/zenodo.20744271 (ref. ^88^).
