# Publisher Correction: Continual integration of single-cell multimodal data with MIRACLE

**DOI:** 10.1038/s43588-026-01053-2

**Published:** 2026-08-26

**Authors:** Jiahao Zhou, Jing Wang, Shuofeng Hu, Tongtong Kan, Chao Feng, Xiaohan Qiang, Guohua Dong, Jinhui Shi, Runyan Liu, Xiaochen Bo, Le Ou-Yang, Xiaomin Ying, Zhen He

**Affiliations:** 1 Center for Computational Biology, Beijing Institute of Basic Medical Sciences, Beijing, China; 2 College of Electronics and Information Engineering, Shenzhen University, Shenzhen, China; 3 School of Statistics and Mathematics, Central University of Finance and Economics, Beijing, China; 4 Institute of Health Service and Transfusion Medicine, Beijing, China; 5 Faculty of Engineering, Shenzhen MSU-BIT University, Shenzhen, China

**Keywords:** Machine learning, Bioinformatics, Data integration

Correction to: *Nature Computational Science* https://doi.org/10.1038/s43588-026-01030-9, published online 31 July 2026.

In the version of this article initially published, in the last sentence of the “Online strategy 3 (ours) using CL” section of Methods, there was an error where in the text now reading “Initially, ℛ_0_ = ∅…,” the empty set symbol appeared as a theta symbol. In the “Multimodal PBMC datasets” section of Methods, the reference cited for the ISAAC dataset was incorrect; it should have been to Xu, W. et al. *Nat. Methods* https://doi.org/10.1038/s41592-022-01601-4 (2022). Also, ref. 64 had an incorrect article number and ref. 70 an incorrect article title. In the Supplementary Fig. 24a legend, two monocyte populations originally labelled “CD14^+^ T” and “CD16^+^ T” have been amended to read “CD14^+^ Mono” and “CD16^+^ Mono”. The changes have been made in the HTML and PDF versions of the article.

