# Supplementary material for: Continual integration of single-cell multimodal data with MIRACLE
Source: Nat Comput Sci. 2026 Jul 31;6(9):988–1004. doi: 10.1038/s43588-026-01030-9 (PMC13593572; doi:10.1038/s43588-026-01030-9)
Supplement: Supplementary file 1 [file 43588_2026_1030_MOESM1_ESM.pdf]

nature computational science

Supplementary information

https://doi.org/10.1038/s43588-026-01030-9

Continual integration of single-cell multimodal data with MIRACLE

In the format provided by the authors and unedited

Supplementary Information

Supplementary Section 1: Detailed Flu A integration analysis

Identification of HERC1$^+$ T cells in Flu A patients

During Flu A dataset integration, MIRACLE's label transfer detected an unknown cell population (Fig. 6a), which was further characterized by high *HERC1* expression (Fig. 6e and Supplementary Fig. 25a). Comparison with the annotations from the original study [1] revealed that these cells also expressed *PRKCH*, *MAML2*, and *ARHGAP15* (Supplementary Fig. 25b), originally annotated as TCells_PRKCH (Supplementary Fig. 25c,d). However, the original study did not assign a specific function to this population or explore its role in detail.

To systematically characterize this subset, we designated it HERC1$^+$ T cells based on its marker gene profile in the integrated dataset. Additionally, to streamline downstream analyses, MAIT cells and $\gamma\delta$ T cells were merged into a broader category termed Unconventional T cells.

PD-1 pathway activation in HERC1$^+$ T Cells

To explore the functional characteristics of HERC1$^+$ T cells, we conducted gene set enrichment analysis (GSEA) on their differentially expressed genes in the Flu A dataset. This revealed significant enrichment of the PD-1 pathway ($P = 0.0297$; Fig. 6f). Additionally, immune checkpoint-related genes within this pathway were highly expressed in HERC1$^+$ T cells (Supplementary Fig. 25e).

Notably, in influenza A virus (IAV)-infected patients, HERC1$^+$ T cells exhibited high expression of *CD274* (encoding PD-L1) and *PDCD1LG2* (encoding PD-L2), while *PDCD1* (encoding PD-1) was predominantly expressed in CD8$^+$ CTLs (Fig. 6g). However, this co-expression pattern was absent in both healthy individuals (Supplementary Fig. 25f) and COVID-19 patients (Supplementary Fig. 25g). These findings suggest that during IAV infection, HERC1$^+$ T cells in peripheral blood may be associated with CD8$^+$ CTL exhaustion via the PD-1/PD-L1/PD-L2 axis, with potential implications for the antiviral cytotoxic response. However, further studies are needed to determine the precise role of this subset in immune regulation.

HERC1$^+$ T cells and CD8$^+$ CTL exhaustion

To assess CD8$^+$ CTL exhaustion, we compared CD8$^+$ CTLs across Flu A patients, COVID-19 patients, and healthy individuals. T cell exhaustion markers (*PDCD1*, *CD244*, *TIGIT*, *LAG3*) were upregulated in CD8$^+$ CTLs from Flu A patients compared to COVID-19 patients (Fig. 6h), whereas genes encoding cytotoxic effectors (IFNs, TNFs, chemokines, ILs) were downregulated in Flu A patients.

Pathway enrichment analysis further revealed that, compared to healthy individuals, CD8$^+$ CTLs in Flu A patients were significantly enriched in pathways related to apoptosis, T cell dysfunction, immune deficiency, interferon suppression, and PD-1 signaling (Fig. 6i). Similarly, relative to COVID-19 patients, CD8$^+$ CTLs in Flu A patients showed preferential enrichment in apoptosis- and PD-1-related pathways (Supplementary Fig. 25h). These results suggest that HERC1$^+$ T cells may be involved in CD8$^+$ CTL exhaustion in IAV infection via the PD-1/PD-L1/PD-L2 axis, though further experimental validation is required.

Implications and future directions

While CD8$^+$ CTL exhaustion in IAV-infected animal models has been reported [2–4], its underlying mechanisms remain unclear. By integrating single-cell PBMC transcriptomic data from IAV-infected patients, we confirmed CD8$^+$ CTL exhaustion and identified HERC1$^+$ T cells as a previously unrecognized subset that may be involved in this process via the PD-1/PD-L1/PD-L2 axis. These findings provide insights into immune dysregulation in IAV infection. However, further research is needed to determine the functional role of HERC1$^+$ T cells in modulating CD8$^+$ T cell exhaustion. In particular, future studies should explore whether targeting this subset could modulate immune responses during IAV infection.

Supplementary Section 2: External validation of identified T cell populations

To validate the robustness of the cell populations identified by MIRACLE—specifically, CD4$^+$ CTLs in COVID-19 and HERC1$^+$ T cells in Flu A—we conducted a series of external validation analyses. These validations were designed to assess the reproducibility of our findings under two distinct conditions: (1) in a completely independent patient dataset and (2) using an alternative, widely-used integration pipeline. This approach ensures that the

identified cell states represent genuine biological signals rather than artifacts of our specific dataset or the MIRACLE methodology.

Validation of CD4$^+$ CTLs in an independent COVID-19 dataset

We analyzed a large-scale COVID-19 scRNA-seq immune atlas [5], focusing on PBMCs from patients with mild/moderate disease. Using a standard Harmony-based Seurat integration pipeline (`https://satijalab.org/seurat/reference/harmonyintegration`), we identified a distinct cluster (Cluster 24) within the CD4$^+$ T cell compartment (Supplementary Fig. 28a–c). These cells co-expressed canonical CD4$^+$ T cell markers (*CD3D*, *CD4*) and key cytotoxicity genes (*GZMB*, *GNLY*), confirming their identity as CD4$^+$ CTLs (Supplementary Fig. 28d). To further validate this quantitatively, we calculated a cytotoxicity score, which was significantly higher in this population compared to other CD4$^+$ T cell subsets (Supplementary Fig. 28e). This result successfully reproduces our finding in a completely independent cohort.

Validation of CD4$^+$ CTLs using an independent preprocessing pipeline

We re-analyzed our original COVID-19 dataset using the same Harmony-based pipeline. This alternative approach robustly recapitulated the identification of CD4$^+$ CTLs (Cluster 7), which showed a highly consistent expression profile of key marker genes (*CD3D*, *CD4*, *GZMB*, *GZMK*, *GNLY*) compared to our original finding (Fig. 6c and Supplementary Fig. 29a–d). The distribution of this population across disease severities was also similar to our MIRACLE results (Supplementary Figs. 24e and 29e). A substantial overlap was observed between the cells identified by both methods, confirming the robustness of this discovery (Supplementary Fig. 29f).

Validation of HERC1$^+$ T cells in an independent Flu A dataset

The availability of public Flu A PBMC datasets suitable for validating this specific cell population is limited. In one such analyzed dataset [6], we found a weak signal for HERC1-expressing T cells in a small subset of batches. Our analysis identified a candidate cluster (Cluster 8) expressing T cell markers and the key genes *HERC1*, *BRAF*, and *PTBP2*, but the signal was not strong enough for a definitive confirmation (Supplementary Fig. 30a–e). The challenge in replicating this specific subpopulation may stem from the inherent biological heterogeneity of influenza infection (for example, viral strain, host genetics and sampling time). This observation highlights the context-dependent nature of this cell state and may underscore the unique sensitivity of MIRACLE in capturing such rare or transient populations.

Validation of HERC1$^+$ T cells using an independent preprocessing pipeline

Crucially, when we re-analyzed our original Flu A dataset with the Harmony-based pipeline, we robustly identified a HERC1$^+$ T cell population (Cluster 12). Its marker gene expression pattern was highly consistent with the population identified by MIRACLE (Supplementary Figs. 25a and 31a–e). Furthermore, the cells identified by Harmony were largely a subset of those identified by MIRACLE, demonstrating high concordance between the methods (Supplementary Fig. 31f). This result confirms that the HERC1$^+$ T cell population is a technically robust finding derived from a genuine biological signal within the data.

Summary

In summary, the existence of CD4$^+$ CTLs in COVID-19 was robustly confirmed through both an independent dataset and an alternative analysis pipeline. The HERC1$^+$ T cell population in Flu A was strongly reproduced using the alternative pipeline on our original data, confirming it as a technically robust finding. While its signal was weak in an independent cohort, this may reflect the context-dependent nature of this rare cell state. Collectively, these validation analyses provide strong support that the cell populations identified in our study are genuine biological signals, not computational artifacts, thereby strengthening the biological conclusions drawn from our continual integration approach.

Supplementary Section 3: Details of Ball Tree sampling

Due to the high dimensionality and noise in the observed single-cell data $\mathcal{X} = \{\mathbf{x}_1, \ldots, \mathbf{x}_N\}$, we first obtain their low-dimensional cell embeddings $\mathcal{Z} = \{\mathbf{z}_1, \ldots, \mathbf{z}_N\}$. We then use the Ball Tree algorithm from the scikit-learn [7]

Python package to perform a multi-resolution partitioning of $\mathcal{Z}$, resulting in an L-layer binary tree $\mathcal{T}$. The i-th tree at layer l, denoted as $\mathcal{T}_i^l$, can be represented as:

$$\mathcal{T}_i^l = \begin{cases} \{\mathcal{T}_{2i-1}^{l+1}, \mathcal{T}_{2i}^{l+1}\}, & \text{if } 1 \leq l < L \\ \mathcal{Z}_i, & \text{if } l = L \end{cases} \tag{1}$$

where at the top layer, $\mathcal{T}_1^1$ represents the entire tree, while at the bottom layer, $\mathcal{T}_i^L = \mathcal{Z}_i \subseteq \mathcal{Z}$ represents the subtree containing the smallest subset of embeddings.

Based on the Ball Tree partitioning, we can sample at each layer. For layer l, we only need to randomly select one cell from each subtree $\mathcal{T}_i^l$ to achieve distribution-preserving sampling at that layer's resolution. Therefore, the larger the l, the higher the resolution and the more subtrees there are, resulting in more cells being collected. For a given sampling ratio γ, we conduct sampling from the bottom layer to the top layer, with resolution decreasing from high to low, until the proportion of sampled data reaches γ. See Algorithm 1 for details.

Algorithm 1 The Ball Tree sampling algorithm.

Input: Original data $\mathcal{X} = \{\mathbf{x}_1, \ldots, \mathbf{x}_N\}$ and sampling ratio γ
Output: Sampled data $\tilde{\mathcal{X}} \subset \mathcal{X}$

```
Generate the embeddings 𝒵 = {z_1, ..., z_N} from 𝒳
Construct a binary tree 𝒯 of L layers via the Ball Tree partition on 𝒵
X̃ ← ∅, l ← L
while |X̃|/N < γ do
    Randomly select ⌊(N − |X̃|)/|𝒯^l|⌋ embeddings from each 𝒯_i^l without replacement, where |𝒯^l| is the number of subtrees at layer l
    Add the original data associated with these embeddings to X̃
    l ← l − 1
end while
```

Supplementary Section 4: The MIRACLE training process

The complete MIRACLE training process is outlined in Algorithm 2.

Algorithm 2 The MIRACLE training algorithm.

Input: New data $\mathcal{X}_t$, previous rehearsal memory $\mathcal{R}_{t-1} = \{\mathcal{R}_{t-1}^{(1)}, \ldots, \mathcal{R}_{t-1}^{(t-1)}\}$, and previous model parameters $\boldsymbol{\theta}_{t-1} = \{\boldsymbol{\theta}_{t-1}^{\text{enc}}, \boldsymbol{\theta}_{t-1}^{\text{dec}}, \boldsymbol{\theta}_{t-1}^{\text{cls}}\}$
Output: Updated model parameters $\boldsymbol{\theta}_t = \{\boldsymbol{\theta}_t^{\text{enc}}, \boldsymbol{\theta}_t^{\text{dec}}, \boldsymbol{\theta}_t^{\text{cls}}\}$

```
Denote 𝒳_t as ℛ_{t−1}^{(t)}, and initialize θ_t as θ̂_t = [θ_{t−1}, θ̃_t] according to Eq. (5) of the main text
for i = 1, 2, ..., I do
    for j ∈ {new, rehersal} do
        if j = new then
            τ ← t, η ← N_τ/S_t
        else if j = rehersal then
            Randomly assign τ a value from {1, ..., t − 1}, η ← (N_τ/S_t)(t − 1)
        end if
        Sample a minibatch {x_{τ,n}}_{n∈𝒩_{τ,i}} from ℛ_{t−1}^{(τ)}, where 𝒩_{τ,i} ⊂ {1, ..., N_τ}
        for k = 1, 2, ..., K do
            Freeze θ_t^enc and update θ_t^cls via SGD with loss η/|𝒩_{τ,i}| Σ_{n∈𝒩_{τ,i}} l^r(θ_t^cls; x_{τ,n}, θ_t^enc)
        end for
        Freeze θ_t^cls and update {θ_t^enc, θ_t^dec} via SGD with loss η/|𝒩_{τ,i}| Σ_{n∈𝒩_{τ,i}} l^{f,g}(θ_t^enc, θ_t^dec; x_{τ,n}, θ_t^cls)
    end for
end for
```

Supplementary Section 5: Details of loss functions inherited from the base model

MIRACLE employs MIDAS [8] as its base model for integrating single-cell multimodal mosaic data. The training objective of MIRACLE is therefore inherited from MIDAS, which is designed to disentangle biological variation

from technical noise while aligning different modalities. The overall training process involves optimizing two main loss functions in an adversarial manner: the encoder-decoder loss $l^{f,g}$ and the classifier loss l^r. We denote the parameters for the encoders, decoders, and classifiers as θ^{enc}, θ^{dec}, and θ^{cls}, respectively. For notational simplicity, here we omit the time index t from parameters (for example, θ_t^{enc} becomes θ^{enc}) and the batch index τ from data samples (for example, $\mathbf{x}_{\tau,n}$ becomes $\mathbf{x}_n$). In this context, $\mathbf{x}_n$ represents the n-th sample from all available training data $\{\mathbf{x}_n\}_{n=1}^N$ rather than a specific batch, and similar to Eq. (9) of the main text, we have $\mathbf{x}_n = \{\{\mathbf{o}_n^m\}_{m\in\mathcal{M}_n}, s_n\}$.

Encoder-decoder loss ($l^{f,g}$)

The primary objective is to minimize the encoder-decoder loss $l^{f,g}$ with respect to θ^{enc} and θ^{dec} for all observations $\{\mathbf{x}_n\}_{n=1}^N$:

$$l^{f,g}(\theta^{\text{enc}}, \theta^{\text{dec}}; \mathbf{x}_n, \theta^{\text{cls}}) = l^{\text{elbo}}(\theta^{\text{enc}}, \theta^{\text{dec}}; \mathbf{x}_n) + l^{\text{mod}}(\theta^{\text{enc}}; \mathbf{x}_n) + l^{\text{ib}}(\theta^{\text{enc}}; \mathbf{x}_n, \theta^{\text{cls}}) \tag{2}$$

This loss comprises three components:

(1) Evidence lower bound loss (l^{elbo}). This is a VAE objective, which maximizes the lower bound of the data log-likelihood. It consists of a reconstruction term and a Kullback-Leibler (KL) divergence term for regularization. For a single cell observation $\mathbf{x}_n$, the loss is:

$$\begin{aligned} l^{\text{elbo}}(\theta^{\text{enc}}, \theta^{\text{dec}}; \mathbf{x}_n) \triangleq &- \mathbb{E}_{q_{\theta^{\text{enc}}}(\mathbf{c},\mathbf{u}|\mathbf{x}_n)}\left[\psi \log p_{\theta^{\text{dec}}}(s_n|\mathbf{u}) + \sum_{m\in\mathcal{M}_n} \log p_{\theta^{\text{dec}}}(\mathbf{o}_n^m|\mathbf{c},\mathbf{u})\right] \\ &+ \text{KL}\left(q_{\theta^{\text{enc}}}(\mathbf{c},\mathbf{u}|\mathbf{x}_n) \| p(\mathbf{c},\mathbf{u})\right) \end{aligned} \tag{3}$$

where $\mathbf{c}$ and $\mathbf{u}$ are the latent variables for biological state (cell embedding) and technical noise, respectively. $q_{\theta^{\text{enc}}}$ is the variational posterior approximated by the encoders, and $p_{\theta^{\text{dec}}}$ is the likelihood defined by the decoders. The weight hyperparameter $\psi \geq 1$ encourages the model to encode batch-specific information into $\mathbf{u}$.

(2) Self-supervised modality alignment loss (l^{mod}). To ensure that different modalities are aligned in the latent space, this loss penalizes the dispersion among unimodal latent variables. Leveraging self-supervised learning, each cell's multimodal input $\mathbf{x}_n$ is used to construct unimodal inputs $\mathbf{x}_n^m = \{\mathbf{o}_n^m, s_n\}_{m\in\mathcal{M}_n}$, each of which is associated with the latent variables $\mathbf{z}^m = \{\mathbf{c}^m, \mathbf{u}^m\}$. The loss is defined as the expected sum of squared deviations of each modality's latent variable $\mathbf{z}^m$ from their mean $\bar{\mathbf{z}}$:

$$l^{\text{mod}}(\theta^{\text{enc}}; \mathbf{x}_n) \triangleq \pi\ \mathbb{E}_{q_{\theta^{\text{enc}}}(\{\mathbf{z}^m\}_{m\in\mathcal{M}_n}|\mathbf{x}_n)}\left[\sum_{m\in\mathcal{M}_n} \|\mathbf{z}^m - \bar{\mathbf{z}}\|_2^2\right] \tag{4}$$

where $\pi > 0$ is a weighting hyperparameter.

(3) Information-theoretic disentanglement loss (l^{ib}). This loss employs the information bottleneck (IB) principle to enforce the disentanglement of $\mathbf{c}$ and $\mathbf{u}$. It aims to minimize two mutual information terms: $\text{I}(s,\mathbf{c})$ between the batch ID s and the cell embedding $\mathbf{c}$, and $\text{I}(\mathbf{o},\mathbf{u})$ between the observed modalities $\mathbf{o}$ and the technical noise $\mathbf{u}$. The minimization of $\text{I}(s,\mathbf{c})$ is achieved through an adversarial scheme involving a set of classifiers. The resulting loss term for the encoder is defined for both the joint posterior and the unimodal posteriors from the self-supervised task. Let $\hat{l}^{\text{ib}}(\theta^{\text{enc}}; \mathbf{x}, \hat{\theta}^{\text{cls}})$ be the individual IB loss for a given input $\mathbf{x} = \{\mathbf{o}, s\}$ and a classifier parameter $\hat{\theta}^{\text{cls}}$:

$$\begin{aligned} \hat{l}^{\text{ib}}(\theta^{\text{enc}}; \mathbf{x}, \hat{\theta}^{\text{cls}}) \triangleq\ & \omega^s\ \mathbb{E}_{q_{\theta^{\text{enc}}}(\mathbf{c}|\mathbf{x})}\left[\log p_{\hat{\theta}^{\text{cls}}}(s|\mathbf{c})\right] + \omega^x\, \text{KL}\left(q_{\theta^{\text{enc}}}(\mathbf{u}|\mathbf{x}) \| p(\mathbf{u})\right) \\ &- \omega^x\ \mathbb{E}_{q_{\theta^{\text{enc}}}(\mathbf{u}|\mathbf{x})}\left[\log p_{\theta^{\text{dec}}}(s|\mathbf{u})\right] \end{aligned} \tag{5}$$

where $\omega^s, \omega^x > 0$ are the weight hyperparameters. Then, the total IB loss l^{ib} for a cell is the sum of the loss on the multimodal input $\mathbf{x}_n$ and the losses on each unimodal input $\mathbf{x}_n^m$:

$$l^{\text{ib}}(\theta^{\text{enc}}; \mathbf{x}_n, \theta^{\text{cls}}) \triangleq \hat{l}^{\text{ib}}(\theta^{\text{enc}}; \mathbf{x}_n, \theta_{\mathcal{M}}^{\text{cls}}) + \sum_{m\in\mathcal{M}_n} \hat{l}^{\text{ib}}(\theta^{\text{enc}}; \mathbf{x}_n^m, \theta_m^{\text{cls}}) \tag{6}$$

where $\theta^{\text{cls}} = \{\theta_{\mathcal{M}}^{\text{cls}}, \{\theta_m^{\text{cls}}\}_{m\in\mathcal{M}}\}$ is the set of all classifier parameters, $\theta_{\mathcal{M}}^{\text{cls}}$ is the parameter for the classifier acting on the joint embedding $\mathbf{c}$, and θ_m^{cls} is the parameter for the classifier on each unimodal embedding $\mathbf{c}^m$. The first term in $\hat{l}^{\text{ib}}$, involving $p_{\hat{\theta}^{\text{cls}}}(s|\mathbf{c})$, represents the adversarial component where the encoder is trained to generate cell embeddings $\mathbf{c}$ that 'fool' the classifiers, thereby maximizing the classifiers' prediction error and minimizing the information about batch s contained in $\mathbf{c}$.

Classifier loss (l^r)

The classifiers, parameterized by $\boldsymbol{\theta}^{\text{cls}}$, are trained to correctly predict the batch ID s_n from the cell embedding $\mathbf{c}$ generated by the encoder. This is achieved by minimizing the negative log-likelihood (cross-entropy loss). Let $\hat{l}^r(\hat{\boldsymbol{\theta}}^{\text{cls}}; \mathbf{x}, \boldsymbol{\theta}^{\text{enc}})$ be the individual classifier loss for a given input $\mathbf{x}$ and a classifier parameter $\hat{\boldsymbol{\theta}}^{\text{cls}}$:

$$\hat{l}^r(\hat{\boldsymbol{\theta}}^{\text{cls}}; \mathbf{x}, \boldsymbol{\theta}^{\text{enc}}) \triangleq \mathbb{E}_{q_{\boldsymbol{\theta}^{\text{enc}}}(\mathbf{c}|\mathbf{x})} \left[-\log p_{\hat{\boldsymbol{\theta}}^{\text{cls}}}(s|\mathbf{c}) \right] \tag{7}$$

The total classifier loss l^r for a cell is the sum of the loss on the multimodal input $\mathbf{x}_n$ and the losses on each unimodal input $\mathbf{x}_n^m$:

$$l^r(\boldsymbol{\theta}^{\text{cls}}; \mathbf{x}_n, \boldsymbol{\theta}^{\text{enc}}) \triangleq \hat{l}^r(\boldsymbol{\theta}^{\text{cls}}_{\mathcal{M}}; \mathbf{x}_n, \boldsymbol{\theta}^{\text{enc}}) + \sum_{m \in \mathcal{M}_n} \hat{l}^r(\boldsymbol{\theta}^{\text{cls}}_m; \mathbf{x}_n^m, \boldsymbol{\theta}^{\text{enc}}) \tag{8}$$

The adversarial training proceeds by alternating between minimizing l^r to train the classifiers and minimizing $l^{f,g}$ (which includes adversarial terms related to l^r) to train the encoders and decoders. This process forces the encoder to learn a embedding $\mathbf{c}$ that is invariant to batch effects.

Loss hyperparameters

Following MIDAS, we set the above technical noise likelihood loss weight ψ to 1000, the modality alignment loss weight π to 50, the technical IB loss weight ω^s to 30, and the biological IB loss weight ω^x to 4. In Algorithm 2, the number of updates K for the classifier parameters $\boldsymbol{\theta}^{\text{cls}}$ in each iteration is set to 3.

Supplementary Section 6: Detailed definitions of integration metrics

We used the scIB and scMIB metrics to assess the integration performance of different methods. The detailed definitions of these metrics for batch correction, modality alignment, and biological conservation performance are as follows.

Batch correction metrics

The batch correction metrics include graph iLISI y^{iLISI}, graph connectivity y^{gc}, and kBET y^{kBET}.

Graph iLISI. The graph iLISI metric, derived from iLISI [9], measures batch mixing by computing the inverse Simpson's index for diversity in kNN graphs. It estimates the effective number of batches in the neighborhood, ranging from 1 to N, where N is the number of batches. Scores close to the actual batch number indicate good mixing. Unlike the typical iLISI, which is unsuitable for graph-based outputs, scIB's graph iLISI uses a graph-based distance metric to determine the nearest neighbors, avoiding skews in graph-based integration. The graph iLISI scores range from 0 to 1, with 0 indicating strong separation and 1 indicating perfect mixing.

Graph connectivity. Graph connectivity, proposed by scIB, checks if cells with the same label are connected in the kNN graph. For each label c, it calculates the largest connected component size of c-labeled cells divided by the total number of c-labeled cells. The average of these values across all labels represents the total graph connectivity, ranging from 0 to 1. A score of 1 means all same-label cells from different batches are connected in the integrated kNN graph, indicating perfect batch mixing.

kBET. The kBET [10] assesses local batch mixing in the kNN graph by repeatedly testing random cell fractions to see if their local label distributions match the global distribution (null hypothesis). The kBET value is the rejection rate of this hypothesis across neighborhoods, with values near 0 indicating well-mixed batches. scIB modifies kBET with a diffusion-based correction for unbiased comparison of graph- and non-graph-based integration results. kBET values are computed for each label, averaged, and subtracted from 1 to obtain the final kBET score.

Modality alignment metrics

The modality alignment metrics include modality ASW, FOSCTTM, label transfer F1, ATAC AUROC, RNA Pearson's r, and ADT Pearson's r.

Modality ASW. The modality ASW measures the alignment of different modality embeddings' distributions. Originally, ASW [11] assessed cluster separation. In scIB, ASW is adapted to evaluate batch effect removal, yielding a batch ASW score from 0 to 1, where 0 indicates strong batch separation and 1 indicates perfect batch mixing. Similarly, replacing batch embeddings with modality embeddings allows us to define a modality ASW, where 0 denotes strong modality separation and 1 denotes perfect modality alignment. In our comparison methods, modality embeddings are generated by processing each modality individually through the trained model.

FOSCTTM. The FOSCTTM [12] measures the alignment between different modality embeddings. For a modality pair $\{m_1, m_2\}$, the FOSCTTM is calculated as follows:

$$y_{m_1,m_2}^{\text{FOSCTTM}} = \frac{1}{2N}\left(\sum_i \frac{N_i^{m_1}}{N} + \sum_i \frac{N_i^{m_2}}{N}\right) \tag{9}$$

where:

$$\begin{aligned} N_i^{m_1} &= \left|\left\{j \mid \|\mathbf{e}_i^{m_1} - \mathbf{e}_j^{m_2}\|_2 < \|\mathbf{e}_i^{m_1} - \mathbf{e}_i^{m_2}\|_2\right\}\right| \\ N_i^{m_2} &= \left|\left\{j \mid \|\mathbf{e}_j^{m_1} - \mathbf{e}_i^{m_2}\|_2 < \|\mathbf{e}_i^{m_1} - \mathbf{e}_i^{m_2}\|_2\right\}\right| \end{aligned} \tag{10}$$

Here, N is the number of cells, i and j are cell indices, and $\mathbf{e}_i^{m_1}$ and $\mathbf{e}_i^{m_2}$ are the embeddings of cell i in modalities m_1 and m_2, respectively. $N_i^{m_1}$ counts how many cells in modality m_2 that are closer to $\mathbf{e}_i^{m_1}$ than $\mathbf{e}_i^{m_2}$ is, and similarly for $N_i^{m_2}$. To compute the final FOSCTTM score, we first obtain the embeddings for each modality, calculate the FOSCTTM values for every modality pair and then average these values and subtract the result from 1. A higher FOSCTTM score indicates better modality alignment.

Label transfer F1. Label transfer F1 measures the alignment of cell types between different modality embeddings. It evaluates if cell-type labels can be transferred between modalities without bias. For each modality pair, we construct a kNN graph using their embeddings and transfer labels from one modality to the other based on nearest neighbors. The transferred labels are then compared to the original labels using the micro F1 score, termed the label transfer F1. The final score is the average F1 score across all modality pairs.

ATAC AUROC The ATAC AUROC measures the alignment of different modalities in the ATAC feature space, previously used to evaluate ATAC prediction quality [13]. For each method, we convert different non-ATAC modality combinations into ATAC features, calculate the AUROC using true ATAC features as the ground truth, and average these AUROCs for the final score. For example, with ATAC, RNA, and ADT data, we evaluate combinations $\{\text{RNA}\}$, $\{\text{ADT}\}$ and $\{\text{RNA}, \text{ADT}\}$. Each combination is processed by the model to generate imputed data of all modalities $\{\text{ATAC}, \text{RNA}, \text{ADT}\}$, with the generated ATAC features used for AUROC calculation.

RNA Pearson's *r* The RNA Pearson's r value measures the alignment of different modalities in the RNA feature space. For each method, we convert different non-RNA modality combinations into RNA features, calculate the Pearson's r value between each converted result and the true RNA features, and average these values for the final score.

ADT Pearson's *r* The ADT Pearson's r value measures the alignment of different modalities in the ADT feature space, calculated similarly to the RNA Pearson's r value.

Biological conservation metrics

The biological conservation metrics include NMI, ARI, isolated label F1 and graph cLISI.

NMI. The NMI measures the similarity between predefined cell-type labels and clustering results from embeddings or graphs. Optimized Louvain clustering is used as per scIB. NMI scores range from 0 to 1, with 0 indicating no correlation and 1 indicating a perfect match.

ARI. The ARI measures the overlap between two clustering results. The Rand Index (RI [14]) considers both cell pairs in the same clusters and those in different clusters in the predicted (Louvain clustering) and true (cell-type) clusters. The ARI corrects the RI for randomly correct labels, with 0 indicating random labeling and 1 representing a perfect match.

Isolated label F1. scIB proposes the isolated label F1 score to evaluate integration performance, focusing on cells with labels shared by few batches. Labels present in the fewest batches are identified as isolated labels.The isolated label F1 score measures clustering performance on these labels, ranging from 0 to 1, with 1 indicating perfect separation of isolated label cells into one cluster.

Graph cLISI. The graph cLISI is similar to the graph iLISI but focuses on cell-type labels instead of batch labels. While iLISI highlights group mixing, cLISI values group separation [9]. The graph-adjusted cLISI ranges from 0 to 1, with 0 indicating low cell-type separation and 1 indicating strong cell-type separation.

References

[1] Yin Zhang, Lu Zong, Yuanling Zheng, Yu Zhang, Nan Li, Yaoyao Li, Yangyang Jin, Liujing Chen, Jinling Ouyang, Asma Bibi, Ying Huang, and Yuanhong Xu. A single-cell atlas of the peripheral immune response in patients with influenza A virus infection. *iScience*, 26(12), December 2023. ISSN 2589-0042. doi: 10.1016/j.isci.2023.108507.

[2] John A. Rutigliano, Shalini Sharma, Melissa Y. Morris, Thomas H. Oguin, Jennifer L. McClaren, Peter C. Doherty, and Paul G. Thomas. Highly Pathological Influenza A Virus Infection Is Associated with Augmented Expression of PD-1 by Functionally Compromised Virus-Specific CD8+ T Cells. *Journal of Virology*, 88(3): 1636–1651, February 2014. doi: 10.1128/jvi.02851-13.

[3] John J. Erickson, Pengcheng Lu, Sherry Wen, Andrew K. Hastings, Pavlo Gilchuk, Sebastian Joyce, Yu Shyr, and John V. Williams. Acute Viral Respiratory Infection Rapidly Induces a CD8+ T Cell Exhaustion–like Phenotype. *The Journal of Immunology*, 195(9):4319–4330, November 2015. ISSN 0022-1767. doi: 10.4049/jimmunol.1403004.

[4] Ilseyar Akhmetzyanova, Malgorzata Drabczyk, C. Preston Neff, Kathrin Gibbert, Kirsten K. Dietze, Tanja Werner, Jia Liu, Lieping Chen, Karl S. Lang, Brent E. Palmer, Ulf Dittmer, and Gennadiy Zelinskyy. PD-L1 Expression on Retrovirus-Infected Cells Mediates Immune Escape from CD8+ T Cell Killing. *PLOS Pathogens*, 11(10):e1005224, October 2015. ISSN 1553-7374. doi: 10.1371/journal.ppat.1005224.

[5] Xianwen Ren, Wen Wen, Xiaoying Fan, Wenhong Hou, Bin Su, Pengfei Cai, Jiesheng Li, Yang Liu, Fei Tang, Fan Zhang, Yu Yang, Jiangping He, Wenji Ma, Jingjing He, Pingping Wang, Qiqi Cao, Fangjin Chen, Yuqing Chen, Xuelian Cheng, Guohong Deng, Xilong Deng, Wenyu Ding, Yingmei Feng, Rui Gan, Chuang Guo, Weiqiang Guo, Shuai He, Chen Jiang, Juanran Liang, Yi-min Li, Jun Lin, Yun Ling, Haofei Liu, Jianwei Liu, Nianping Liu, Shu-Qiang Liu, Meng Luo, Qiang Ma, Qibing Song, Wujianan Sun, GaoXiang Wang, Feng Wang, Ying Wang, Xiaofeng Wen, Qian Wu, Gang Xu, Xiaowei Xie, Xinxin Xiong, Xudong Xing, Hao Xu, Chonghai Yin, Dongdong Yu, Kezhuo Yu, Jin Yuan, Biao Zhang, Peipei Zhang, Tong Zhang, Jincun Zhao, Peidong Zhao, Jianfeng Zhou, Wei Zhou, Sujuan Zhong, Xiaosong Zhong, Shuye Zhang, Lin Zhu, Ping Zhu, Bin Zou, Jiahua Zou, Zengtao Zuo, Fan Bai, Xi Huang, Penghui Zhou, Qinghua Jiang, Zhiwei Huang, Jin-Xin Bei, Lai Wei, Xiu-Wu Bian, Xindong Liu, Tao Cheng, Xiangpan Li, Pingsen Zhao, Fu-Sheng Wang, Hongyang Wang, Bing Su, Zheng Zhang, Kun Qu, Xiaoqun Wang, Jiekai Chen, Ronghua Jin, and Zemin Zhang. COVID-19 immune features revealed by a large-scale single-cell transcriptome atlas. *Cell*, 184(7):1895–1913.e19, April 2021. ISSN 0092-8674. doi: 10.1016/j.cell.2021.01.053.

[6] Haley E. Randolph, Jessica K. Fiege, Beth K. Thielen, Clayton K. Mickelson, Mari Shiratori, João Barroso-Batista, Ryan A. Langlois, and Luis B. Barreiro. Genetic ancestry effects on the response to viral infection are pervasive but cell type specific. *Science*, November 2021. doi: 10.1126/science.abg0928.

[7] Fabian Pedregosa, Gaël Varoquaux, Alexandre Gramfort, Vincent Michel, Bertrand Thirion, Olivier Grisel, Mathieu Blondel, Peter Prettenhofer, Ron Weiss, Vincent Dubourg, Jake Vanderplas, Alexandre Passos, David Cournapeau, Matthieu Brucher, Matthieu Perrot, and Édouard Duchesnay. Scikit-learn: Machine Learning in Python. *Journal of Machine Learning Research*, 12(85):2825–2830, 2011. ISSN 1533-7928.

[8] Zhen He, Shuofeng Hu, Yaowen Chen, Sijing An, Jiahao Zhou, Runyan Liu, Junfeng Shi, Jing Wang, Guohua Dong, Jinhui Shi, Jiaxin Zhao, Le Ou-Yang, Yuan Zhu, Xiaochen Bo, and Xiaomin Ying. Mosaic integration and knowledge transfer of single-cell multimodal data with MIDAS. *Nature Biotechnology*, pages 1–12, January 2024. ISSN 1546-1696. doi: 10.1038/s41587-023-02040-y.

[9] Ilya Korsunsky, Nghia Millard, Jean Fan, Kamil Slowikowski, Fan Zhang, Kevin Wei, Yuriy Baglaenko, Michael Brenner, Po-ru Loh, and Soumya Raychaudhuri. Fast, sensitive and accurate integration of single-cell data with Harmony. *Nature Methods*, 16(12):1289–1296, December 2019. ISSN 1548-7105. doi: 10.1038/s41592-019-0619-0.

[10] Maren Büttner, Zhichao Miao, F. Alexander Wolf, Sarah A. Teichmann, and Fabian J. Theis. A test metric for assessing single-cell RNA-seq batch correction. *Nature Methods*, 16(1):43–49, January 2019. ISSN 1548-7105. doi: 10.1038/s41592-018-0254-1.

[11] Peter J. Rousseeuw. Silhouettes: A graphical aid to the interpretation and validation of cluster analysis. *Journal of Computational and Applied Mathematics*, 20:53–65, November 1987. ISSN 0377-0427. doi: 10.1016/0377-0427(87)90125-7.

[12] Ritambhara Singh, Pinar Demetci, Giancarlo Bonora, Vijay Ramani, Choli Lee, He Fang, Zhijun Duan, Xinxian Deng, Jay Shendure, Christine Disteche, and William Stafford Noble. Unsupervised manifold alignment for single-cell multi-omics data. In *Proceedings of the 11th ACM International Conference on Bioinformatics, Computational Biology and Health Informatics*, BCB '20, pages 1–10, New York, NY, USA, September 2020. Association for Computing Machinery. ISBN 978-1-4503-7964-9. doi: 10.1145/3388440.3412410.

[13] Kevin E. Wu, Kathryn E. Yost, Howard Y. Chang, and James Zou. BABEL enables cross-modality translation between multiomic profiles at single-cell resolution. *Proceedings of the National Academy of Sciences*, 118(15), April 2021. ISSN 0027-8424, 1091-6490. doi: 10.1073/pnas.2023070118.

[14] William M. Rand. Objective Criteria for the Evaluation of Clustering Methods. *Journal of the American Statistical Association*, 66(336):846–850, 1971. ISSN 0162-1459. doi: 10.2307/2284239.

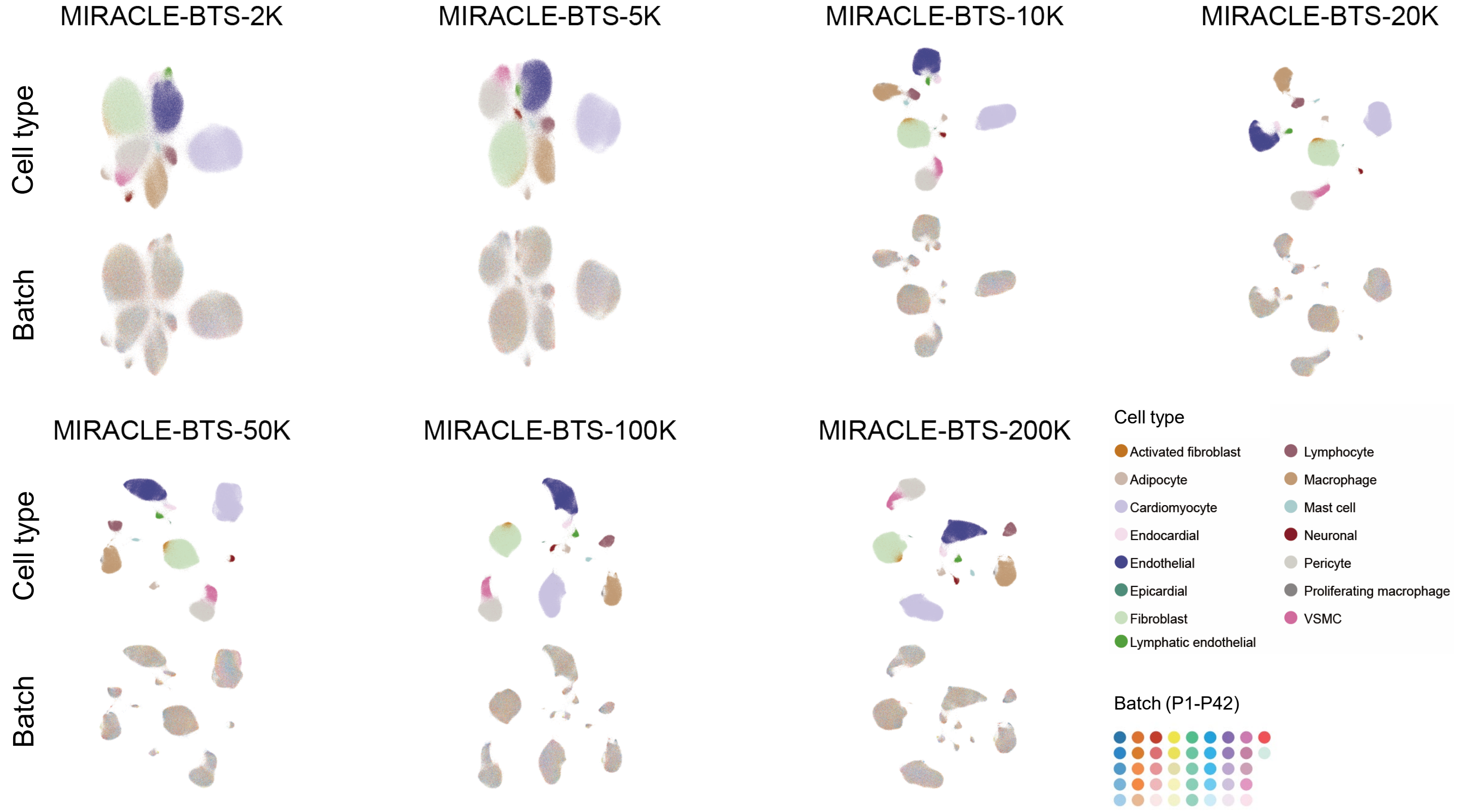

Supplementary Fig. 1: UMAP visualization of MIRACLE-BTS-inferred cell embeddings under different memory constraints.

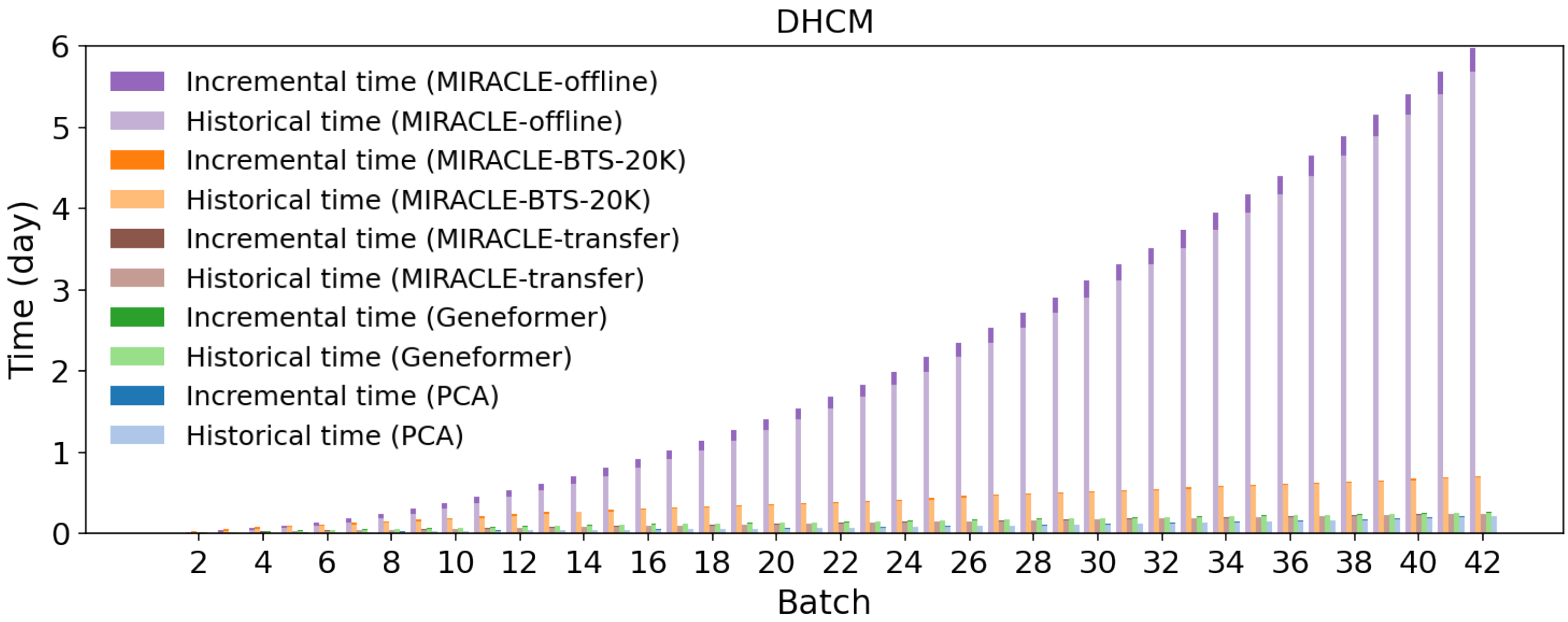

Supplementary Fig. 2: Runtime comparison of different methods on the DHCM dataset. All computational benchmarks (including the runtime and computational memory) were performed on a Linux server equipped with two Intel Xeon Platinum 8558P CPUs (192 cores total), 2 TB of RAM, and one NVIDIA A100 GPU (80 GB).

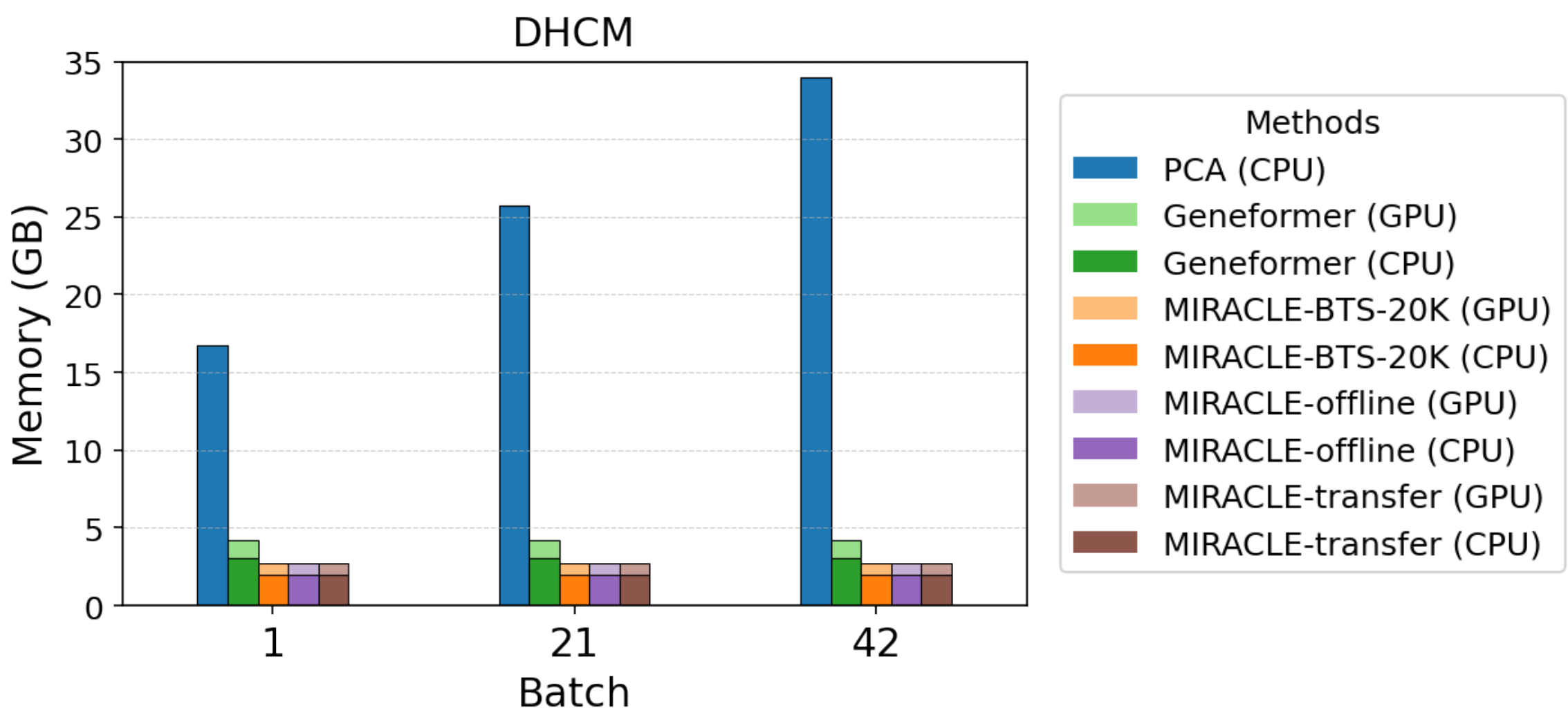

Supplementary Fig. 3: Comparative analysis of computational memory usage on the DHCM dataset. Peak computational memory (CPU RAM and GPU VRAM) consumption of MIRACLE variants, PCA and Geneformer during the integration of the full 523K-cell DHCM dataset. This analysis was performed to benchmark the scalability of different methods. The results demonstrate that all MIRACLE variants maintain a constant and low memory footprint (~2.7 GB). This efficiency is inherent to their deep learning architecture, which processes data in mini-batches, making peak memory consumption independent of the total dataset size. In stark contrast, PCA's memory requirement is substantially higher (34 GB), revealing a significant scalability bottleneck when processing large-scale data. This scalability advantage was further validated on the much larger 2.3M-cell Human Lung Cell Atlas (HLCA) dataset (Supplementary Fig. 4d). On that benchmark, MIRACLE again maintained a low, constant memory footprint. Notably, PCA's memory consumption is highly sensitive not only to cell number but also to feature dimensions. It required substantially more memory for the high-gene DHCM dataset (0.5M cells × 4078 genes; 34 GB) than for the larger but lower-gene HLCA dataset (2.3M cells × 2000 genes; ~3.2 GB, see Supplementary Fig. 4d). MIRACLE's architecture avoids this bottleneck, demonstrating superior memory efficiency and robust scalability for diverse single-cell datasets.

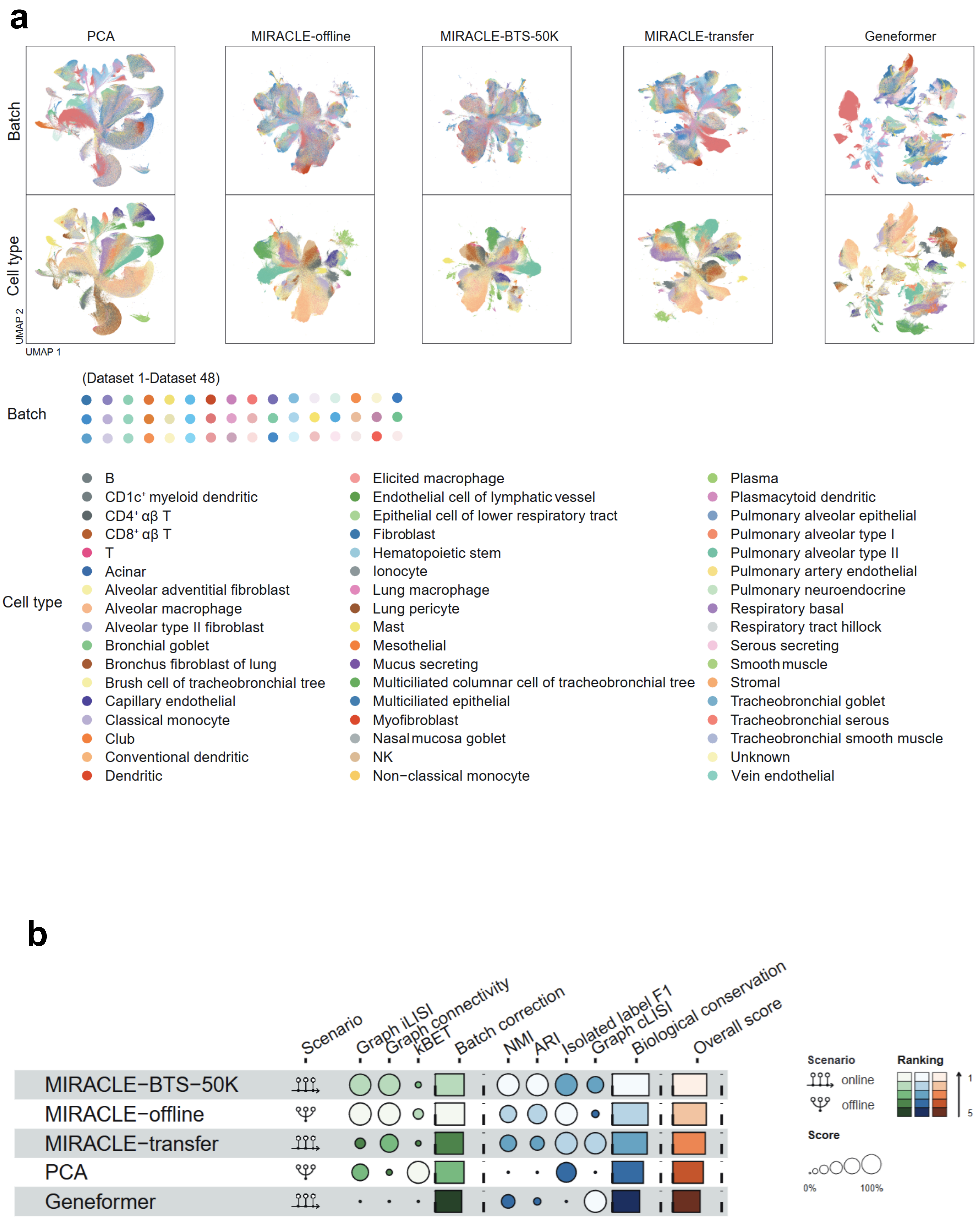

a
PCA
MIRACLE-offline
MIRACLE-BTS-50K
MIRACLE-transfer
Geneformer
Batch
Cell type
UMAP 2
UMAP 1
(Dataset 1-Dataset 48)
Batch
Cell type
B
CD1c⁺ myeloid dendritic
CD4⁺ αβ T
CD8⁺ αβ T
T
Acinar
Alveolar adventitial fibroblast
Alveolar macrophage
Alveolar type II fibroblast
Bronchial goblet
Bronchus fibroblast of lung
Brush cell of tracheobronchial tree
Capillary endothelial
Classical monocyte
Club
Conventional dendritic
Dendritic
Elicited macrophage
Endothelial cell of lymphatic vessel
Epithelial cell of lower respiratory tract
Fibroblast
Hematopoietic stem
Ionocyte
Lung macrophage
Lung pericyte
Mast
Mesothelial
Mucus secreting
Multiciliated columnar cell of tracheobronchial tree
Multiciliated epithelial
Myofibroblast
Nasal mucosa goblet
NK
Non−classical monocyte
Plasma
Plasmacytoid dendritic
Pulmonary alveolar epithelial
Pulmonary alveolar type I
Pulmonary alveolar type II
Pulmonary artery endothelial
Pulmonary neuroendocrine
Respiratory basal
Respiratory tract hillock
Serous secreting
Smooth muscle
Stromal
Tracheobronchial goblet
Tracheobronchial serous
Tracheobronchial smooth muscle
Unknown
Vein endothelial
b
Scenario
Graph iLISI
Graph connectivity
kBET
Batch correction
NMI
ARI
Isolated label F1
Graph cLISI
Biological conservation
Overall score
MIRACLE−BTS−50K
MIRACLE−offline
MIRACLE−transfer
PCA
Geneformer
Scenario
online
offline
Ranking
1
5
Score
0%
100%

c

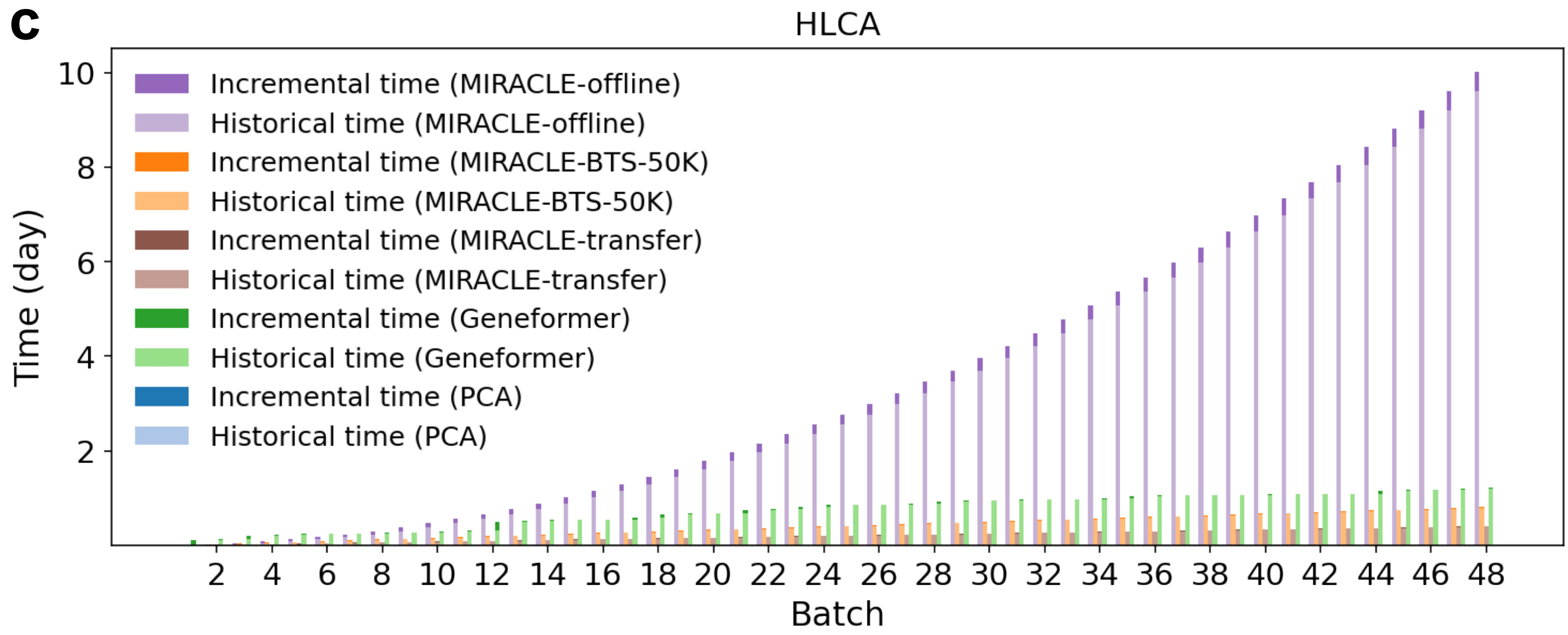

d

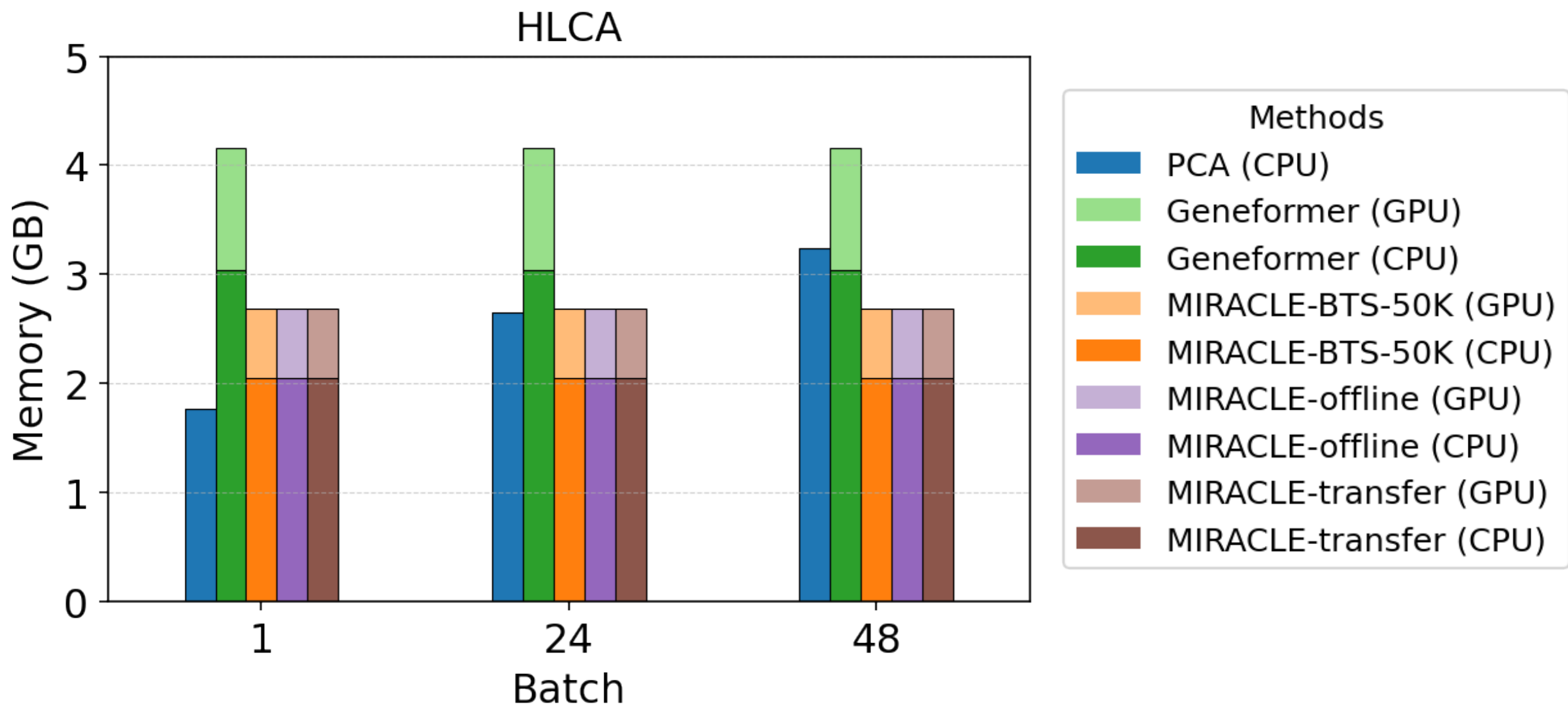

Supplementary Fig. 4: Evaluation of MIRACLE's accuracy and scalability on the large-scale HLCA dataset. The analysis was performed on the HLCA, a challenging benchmark of 2.3 million cells from 48 studies known for its strong batch effects. This experiment validates MIRACLE's performance in a memory-constrained, continual integration scenario on an ultra-large-scale dataset. **a**, UMAP visualizations of cell embeddings. Both MIRACLE-BTS-50K (online) and MIRACLE-offline successfully remove strong batch effects while preserving cell-type annotations. In contrast, PCA and Geneformer fail to adequately mix the batches, and MIRACLE-transfer shows suboptimal integration. **b**, Quantitative evaluation using scIB metrics. MIRACLE-BTS-50K achieves the highest overall score, confirming its superior integration accuracy compared to other online and offline methods. **c**, Cumulative runtime comparison. MIRACLE-BTS-50K demonstrates superior scalability, proving over an order of magnitude faster than its offline counterpart (0.8 days vs. 10 days). While Geneformer (inference-only) and PCA (non-iterative) are also fast, they compromise on integration accuracy (**a**, **b**). **d**, Computational memory usage. MIRACLE's memory footprint remains low and constant (~2.7 GB) due to its mini-batch strategy. In contrast, PCA's memory requirement scales with the increasing data size, posing a scalability bottleneck for large datasets.

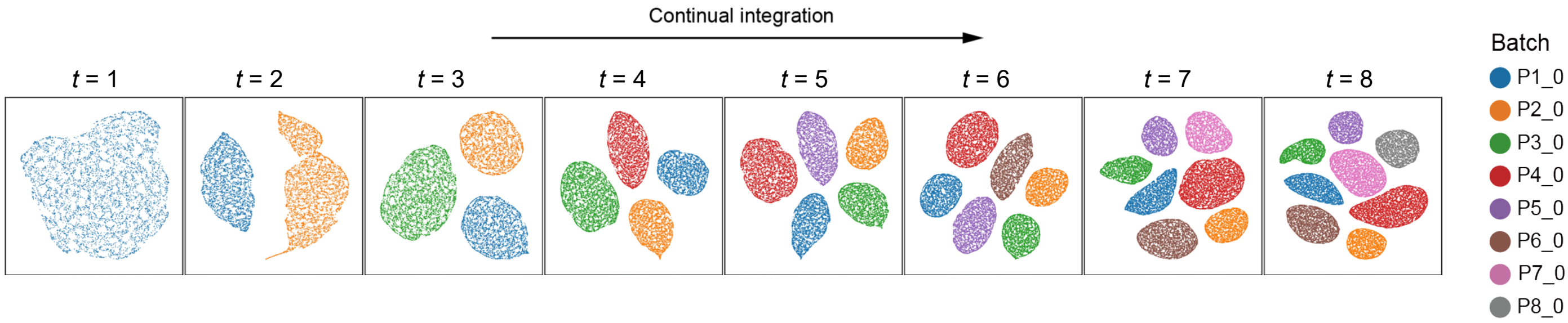

Supplementary Fig. 5: UMAP visualization of technical noise inferred by MIRACLE over time on the WNN bimodal dataset for integration across batches and cell types.

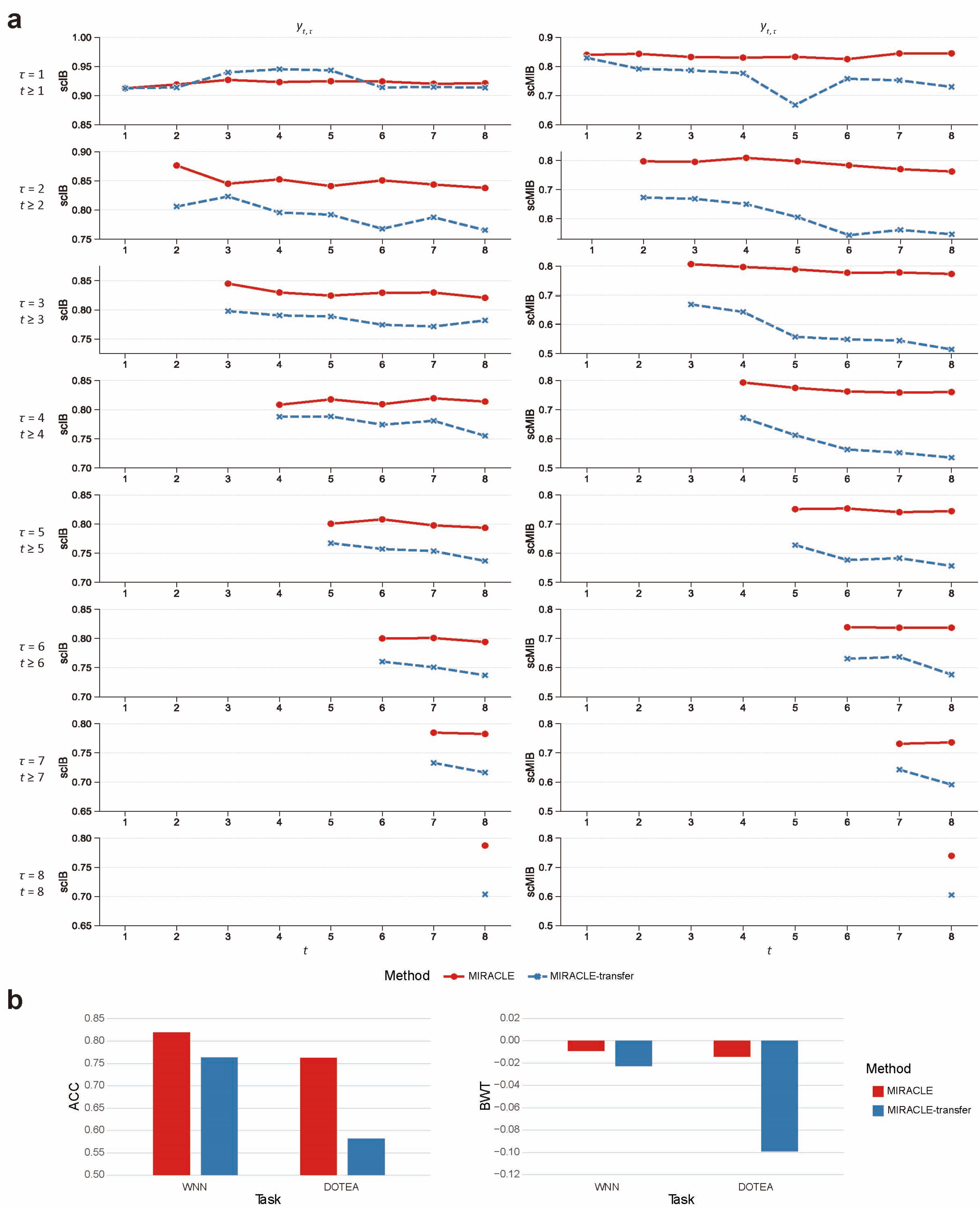
a
$y_{t,\tau}$
$y_{t,\tau}$
$\tau = 1$ $t \geq 1$
$\tau = 2$ $t \geq 2$
$\tau = 3$ $t \geq 3$
$\tau = 4$ $t \geq 4$
$\tau = 5$ $t \geq 5$
$\tau = 6$ $t \geq 6$
$\tau = 7$ $t \geq 7$
$\tau = 8$ $t = 8$
scIB
scMIB
t
t
Method
MIRACLE
MIRACLE-transfer
b
ACC
BWT
WNN
DOTEA
Task
Task
Method
MIRACLE
MIRACLE-transfer

Supplementary Fig. 6: Quantitative analysis of catastrophic forgetting in MIRACLE versus MIRACLE-transfer. This figure provides direct, quantitative evidence that MIRACLE's CL strategy mitigates the catastrophic forgetting observed in MIRACLE-transfer, which uses a conventional fine-tuning approach. The analysis was performed on both the WNN and DOTEA datasets. **a**, Evolution of integration performance over sequential training steps. Each plot visualizes the performance score, $y_{t,\tau}$, which measures the model's performance on the cumulative first τ batches (rows) after being trained up to step t (x-axis). The performance of MIRACLE (red line) remains stable as new data is integrated, indicating effective knowledge retention. In contrast, the performance of MIRACLE-transfer (blue line) exhibits a pronounced degradation, particularly on the more complex DOTEA dataset, demonstrating that it progressively "forgets" previously learned tasks. **b**, Summary of continual learning metrics. Performance is quantified using two standard CL metrics: Average Accuracy (ACC), the average performance across all historical tasks at the end of training, and Backward Transfer (BWT), which measures the influence of learning new tasks on performance on previously learned tasks. A large negative BWT is a hallmark of catastrophic forgetting. The results show MIRACLE achieves a higher ACC and a BWT value closer to zero. The strongly negative BWT for MIRACLE-transfer on the DOTEA dataset quantitatively confirms that it suffers from severe catastrophic forgetting. Collectively, this analysis substantiates the claim that MIRACLE achieves superior continual integration by effectively countering the catastrophic forgetting that plagues standard fine-tuning approaches.

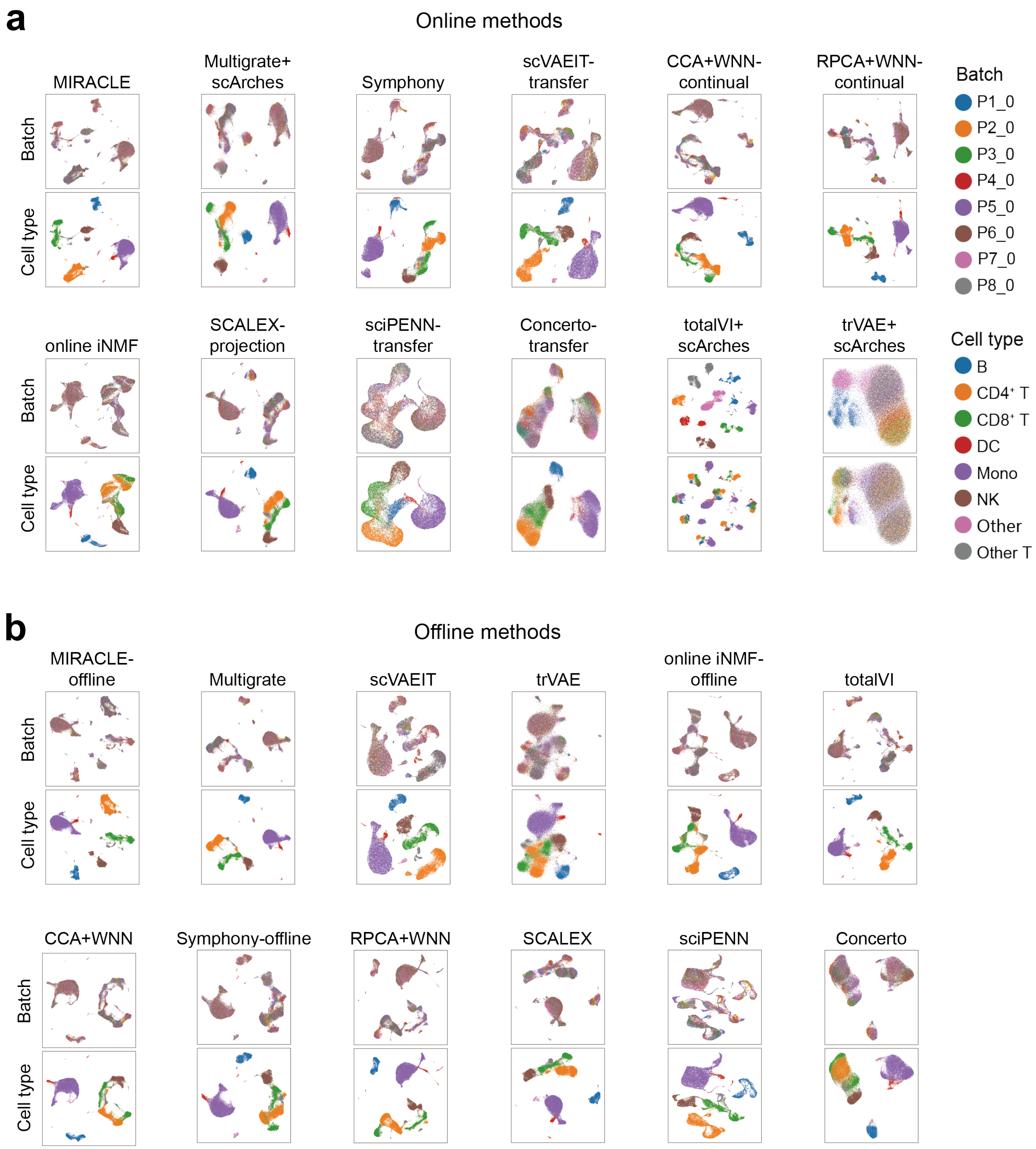

Supplementary Fig. 7: UMAP visualization comparing cell embeddings generated by MIRACLE and other state-of-the-art methods in online (a) and offline (b) scenarios on the Cell-type-missing WNN bimodal dataset for integration across batches and cell types.

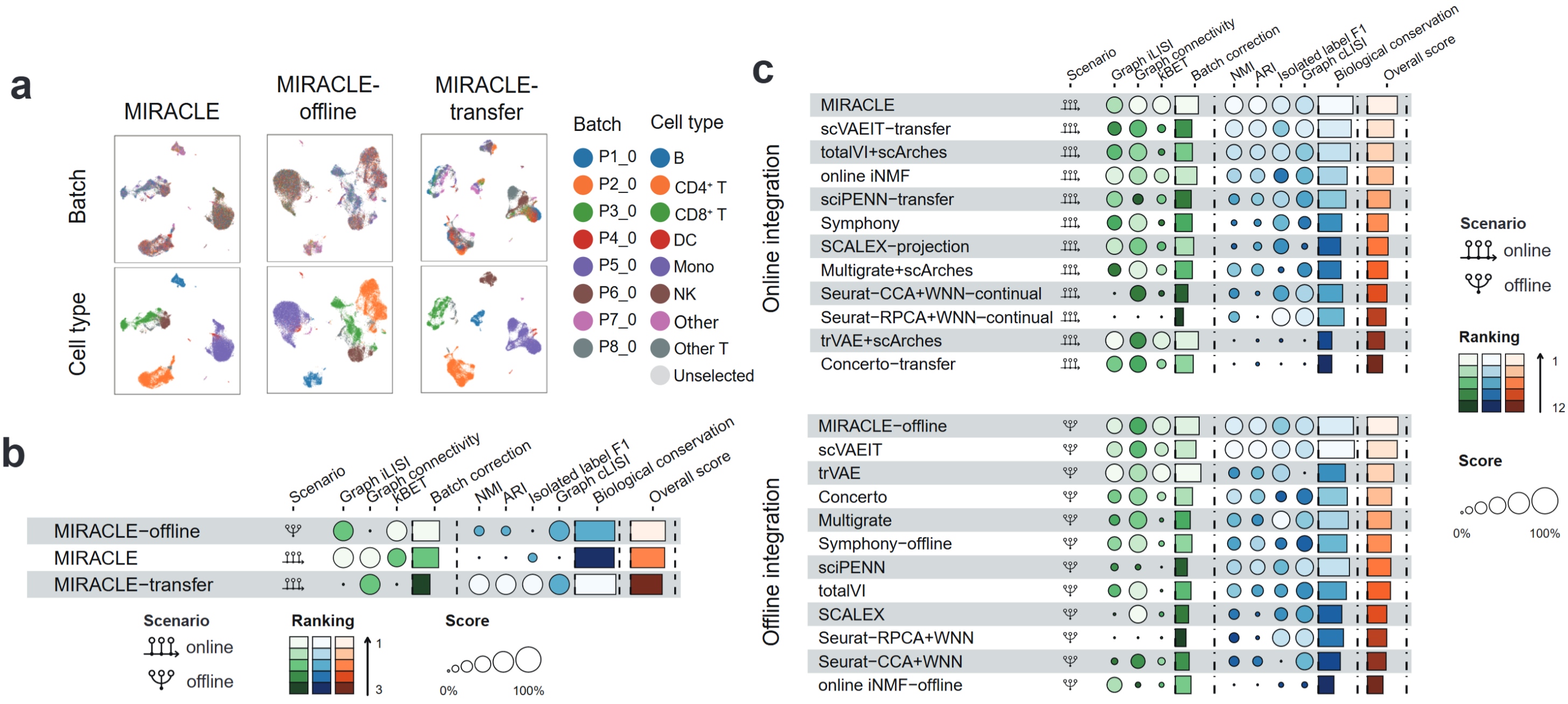

Supplementary Fig. 8: MIRACLE demonstrates superior performance on the demanding Dynamic WNN bimodal dataset. This figure presents the performance evaluation of MIRACLE on the Dynamic WNN dataset, whose challenging design is detailed in Supplementary Table 6. **a**, UMAP visualizations of cell embeddings from MIRACLE and its variants. Both MIRACLE and MIRACLE-offline successfully integrate the eight dynamic batches, maintaining clear separation between distinct cell types and correctly grouping shared cell types, indicating resilience to catastrophic forgetting. **b**, scIB benchmarking comparing MIRACLE with its variants. The results demonstrate that MIRACLE's online performance closely approaches the offline upper bound and significantly surpasses the simple transfer learning approach, validating its effective online learning strategy. **c**, Comprehensive scIB benchmarking of MIRACLE against 11 other state-of-the-art methods in both online (upper) and offline (lower) scenarios. MIRACLE achieves the highest overall score in the online setting, while MIRACLE-offline secures the top rank in the offline setting. This quantitatively establishes MIRACLE's superior robustness for continual integration, even under complex and dynamic batch compositions. The qualitative superiority of MIRACLE in mixing batches while preserving biological identity, as compared to these other methods, is further illustrated in the UMAP visualizations provided in Supplementary Fig. 9.

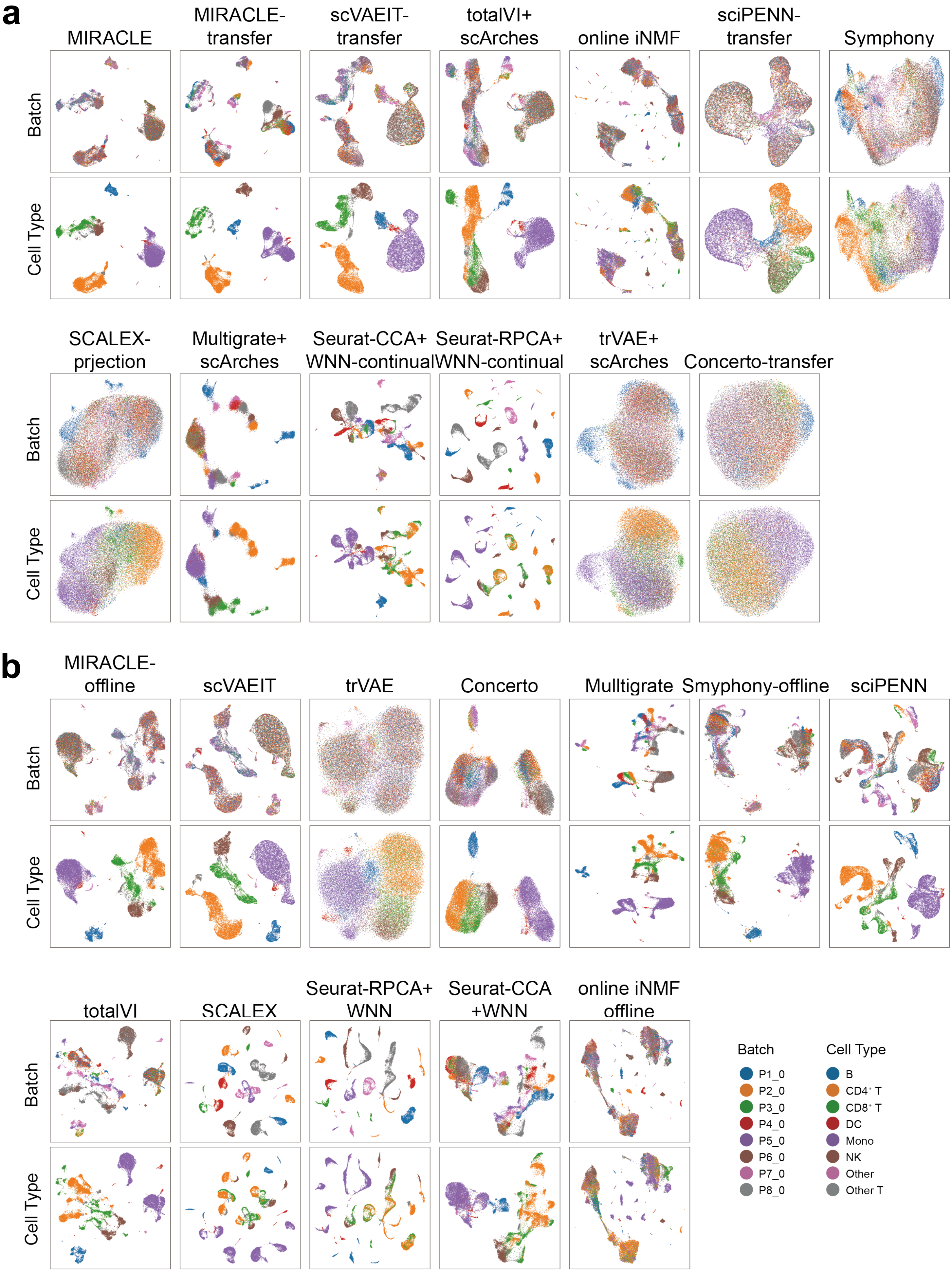

Supplementary Fig. 9: UMAP visualization comparing cell embeddings generated by MIRACLE and other state-of-the-art methods in online (a) and offline (b) scenarios on the Dynamic WNN bimodal dataset for integration across batches and cell types.

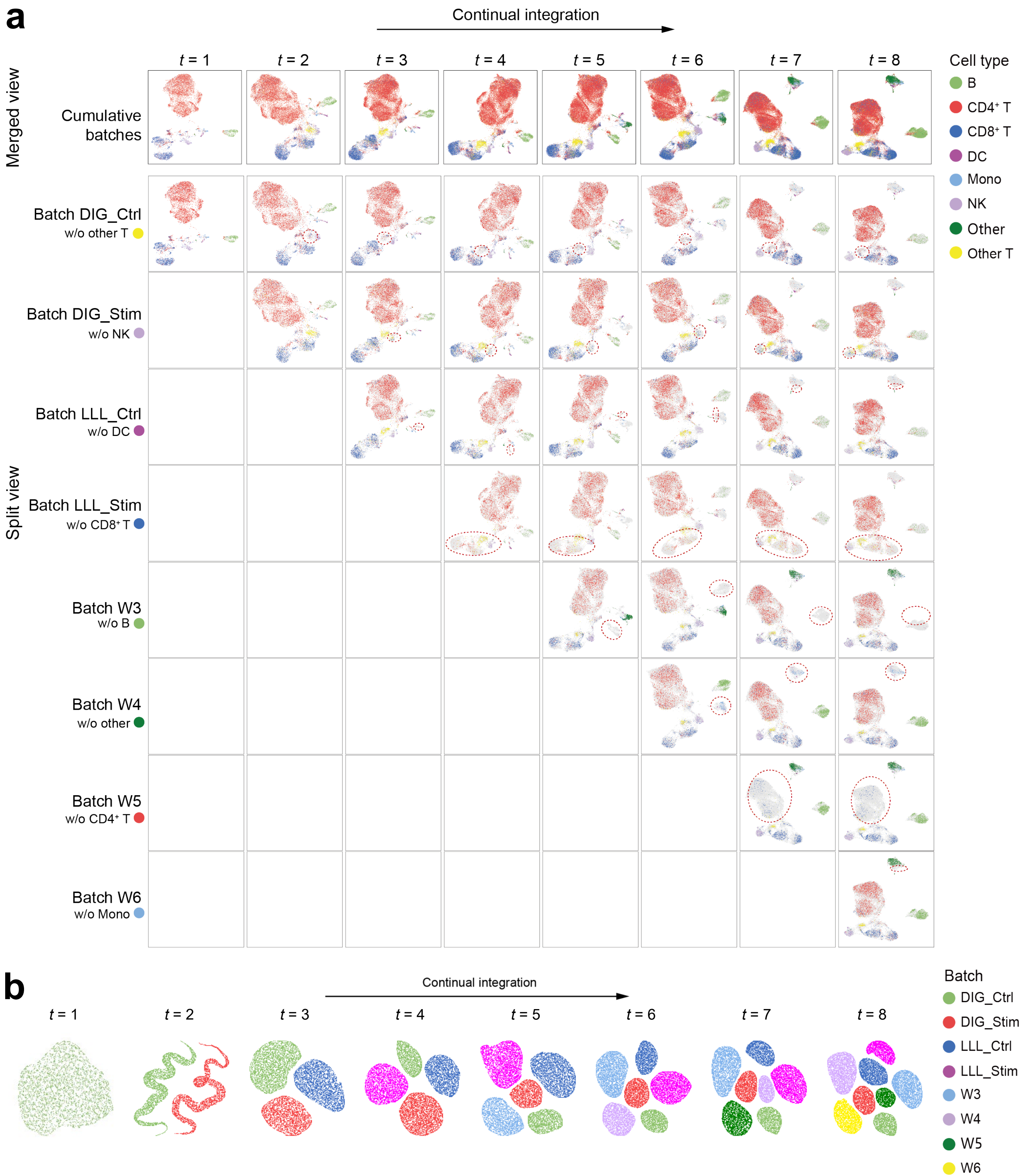

Supplementary Fig. 10: UMAP visualization of MIRACLE-inferred cell embeddings on the DOTEA trimodal dataset in continual mosaic integration. a, UMAP visualization of MIRACLE-inferred cell embeddings across time points, with each column corresponding to a specific time point. Row 1 shows the merged view of the cumulative integration of all available batches over time. Rows 2–9 show the split view, where each row displays the integration results for a single batch at different time points, with missing cell type regions highlighted (red circles); the blank lower-triangular area reflects the stepwise nature of continual integration, as data from future batches are not yet available. Cells are colored by cell type. **b**, UMAP of technical noise inferred by MIRACLE over time.

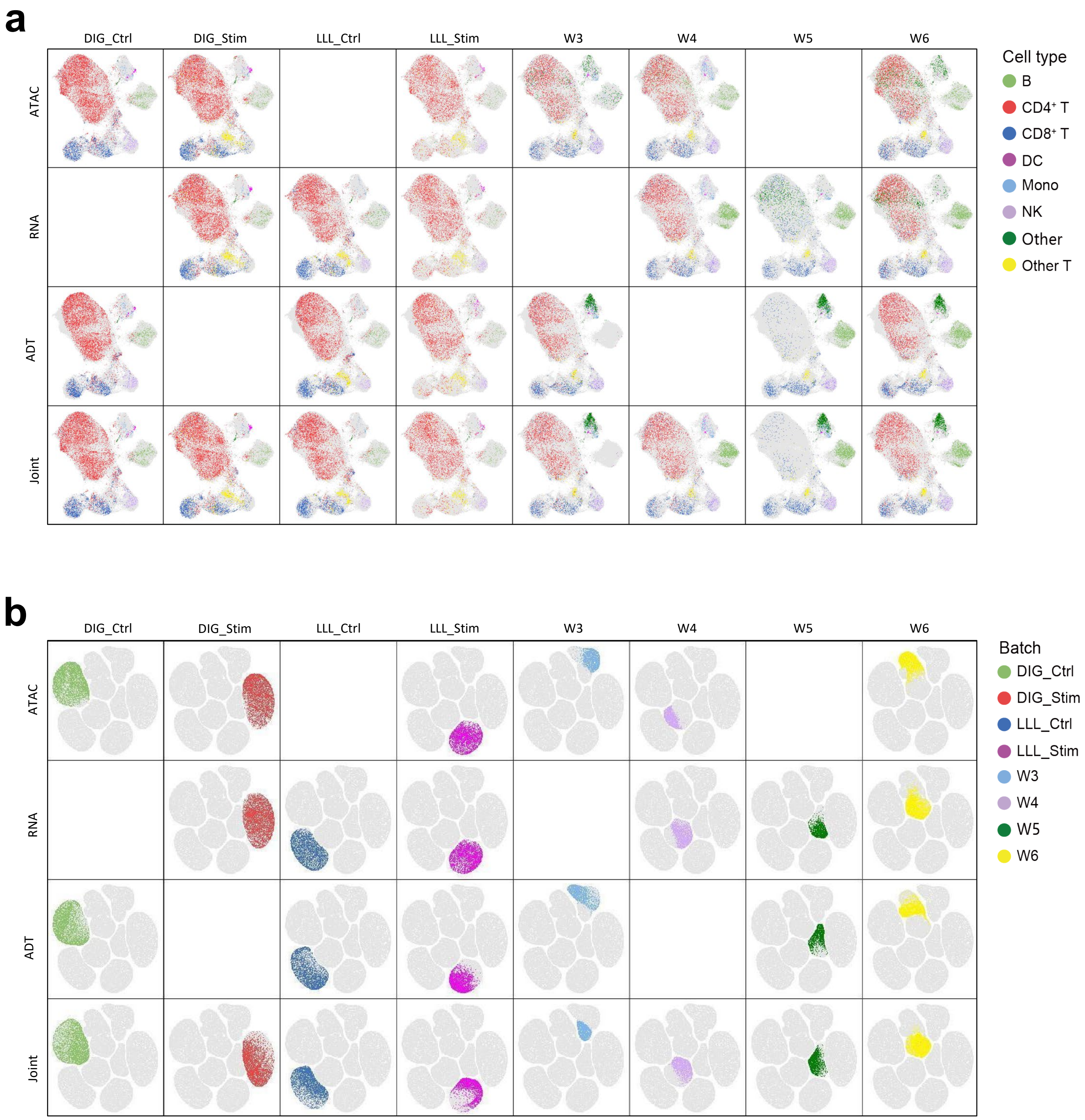

Supplementary Fig. 11: UMAP visualization of cell embeddings (a) and technical noise (b) inferred by MIRACLE on the DOTEA trimodal dataset in continual mosaic integration. UMAPs are vertically split by modality and horizontally split by batch.

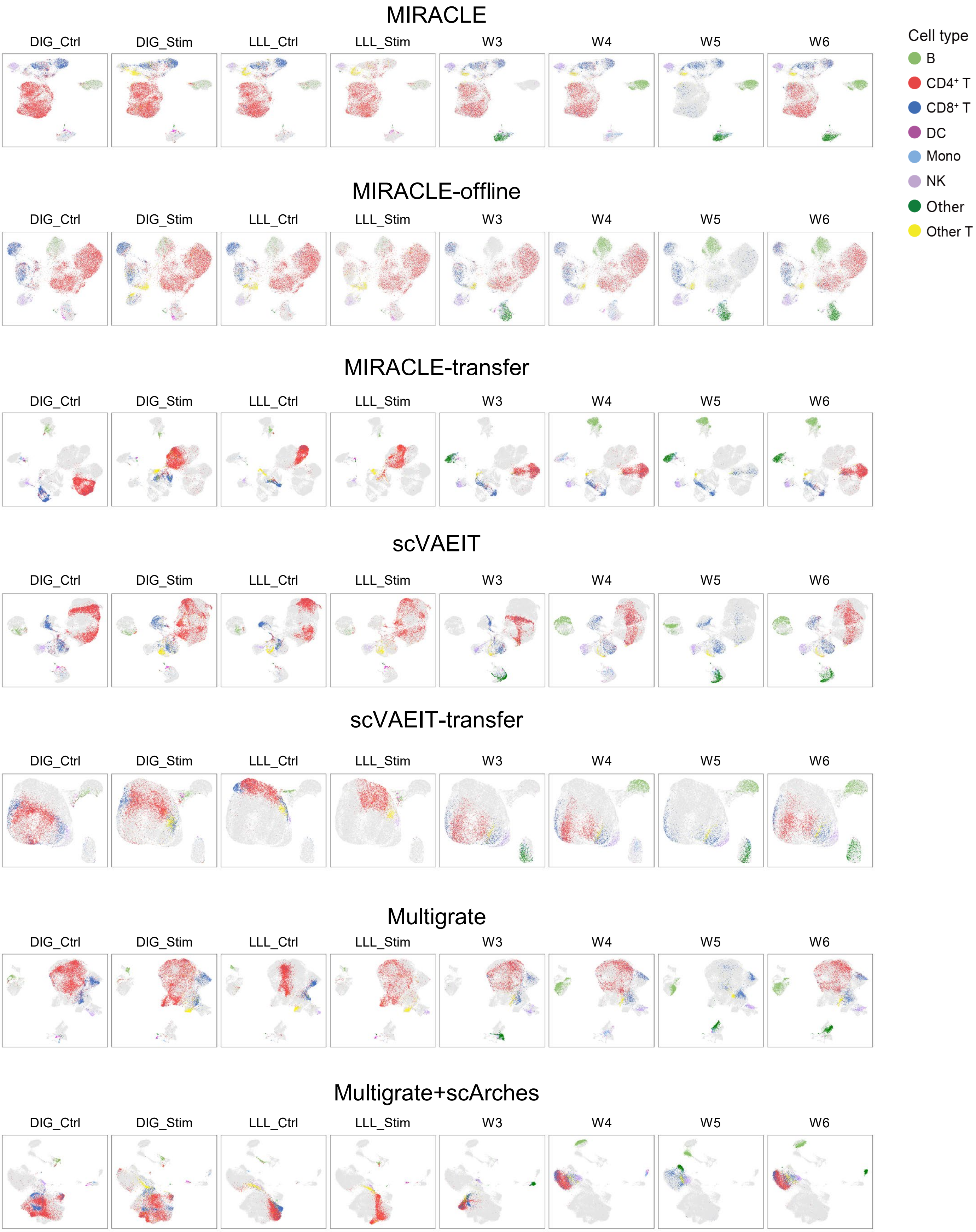

Supplementary Fig. 12: UMAP visualization of MIRACLE and other methods on the DOTEA trimodal dataset in continual mosaic integration. UMAPs are split by batch.

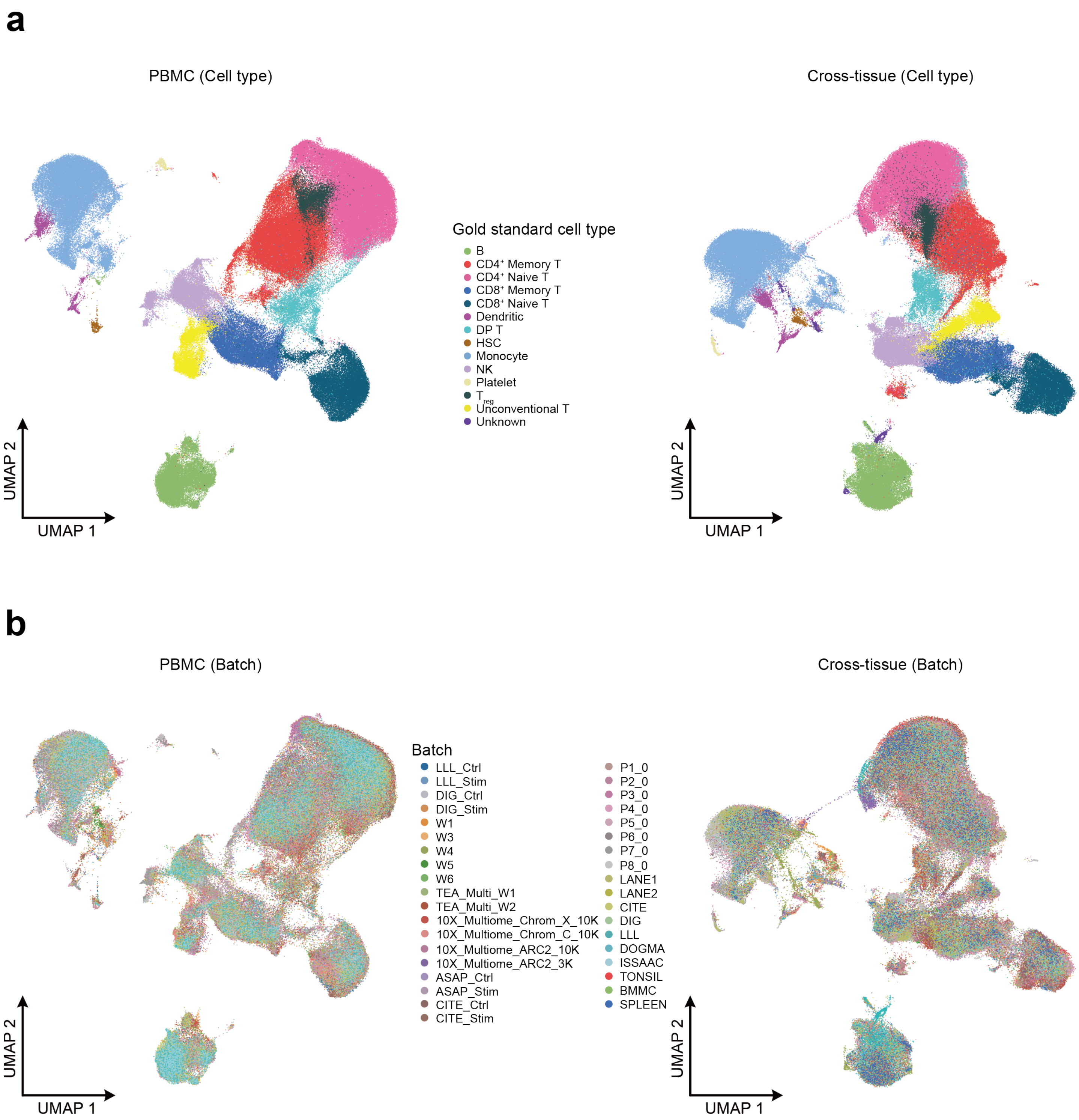

Supplementary Fig. 13: UMAP visualization (merged view) of the cell embeddings inferred by MIRACLE-offline, with the PBMC mosaic dataset containing 34 batches (left) and cross-tissue mosaic dataset containing 37 batches (right). Cells are colored by cell type (a) and batch (b), with cell types derived from MIRACLE-offline.

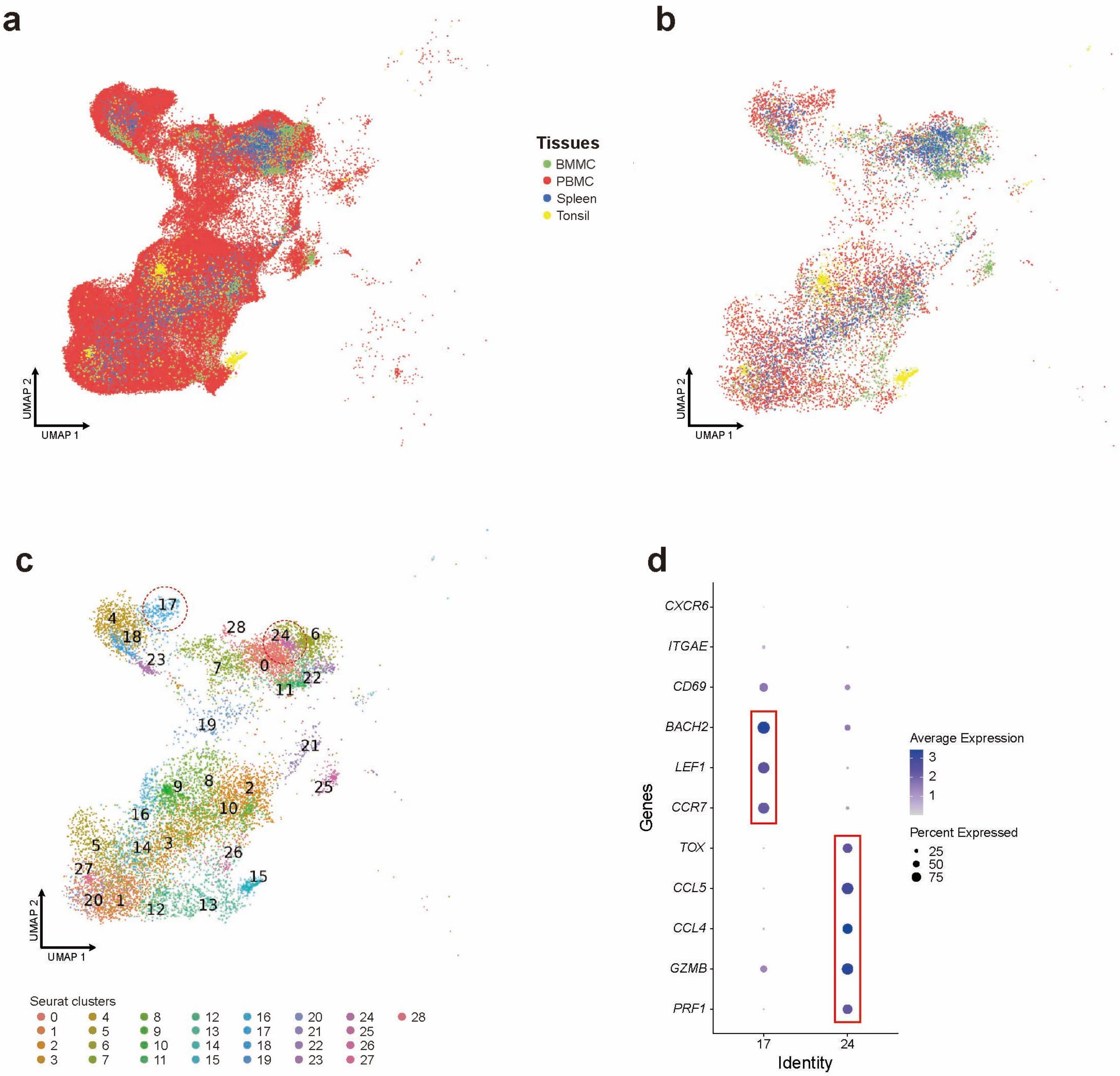

Supplementary Fig. 14: MIRACLE preserves distinct tissue-specific T cell states during cross-tissue integration. To test if MIRACLE avoids overcorrection, T cells were isolated from the integrated cross-tissue atlas (PBMC, tonsil, BMMC, and spleen). **a**, UMAP of all isolated T cells, showing the dominance of the PBMC population. **b**, To prevent clustering bias, PBMCs were downsampled to the average cell count of the other three tissues. **c**, High-resolution sub-clustering of the downsampled population revealed distinct clusters with clear tissue enrichment, demonstrating that MIRACLE did not erroneously merge them. For instance, cluster 17 is 97.3% PBMC-derived, while cluster 24 is 90.2% spleen-derived. **d**, Dot plot of differentially expressed genes confirms the opposing functional identities of these two clusters. The PBMC-enriched cluster 17 represents circulating memory T cells, characterized by the lymph node-homing receptor *CCR7* and transcription factors *LEF1* and *BACH2* that suppress effector differentiation. In stark contrast, the spleen-enriched cluster 24 represents terminally differentiated effector T cells, defined by cytotoxic genes (*PRF1*, *GZMB*), recruitment chemokines (*CCL4*, *CCL5*), and the transcription factor *TOX* driving terminal exhaustion. Notably, this cluster lacks classic tissue-residency markers (e.g., *CD69*), as its transcriptional program is likely dominated by its potent effector function. This functional dichotomy, driven by mutually exclusive transcription factor programs, provides strong evidence that MIRACLE preserves critical biological heterogeneity.

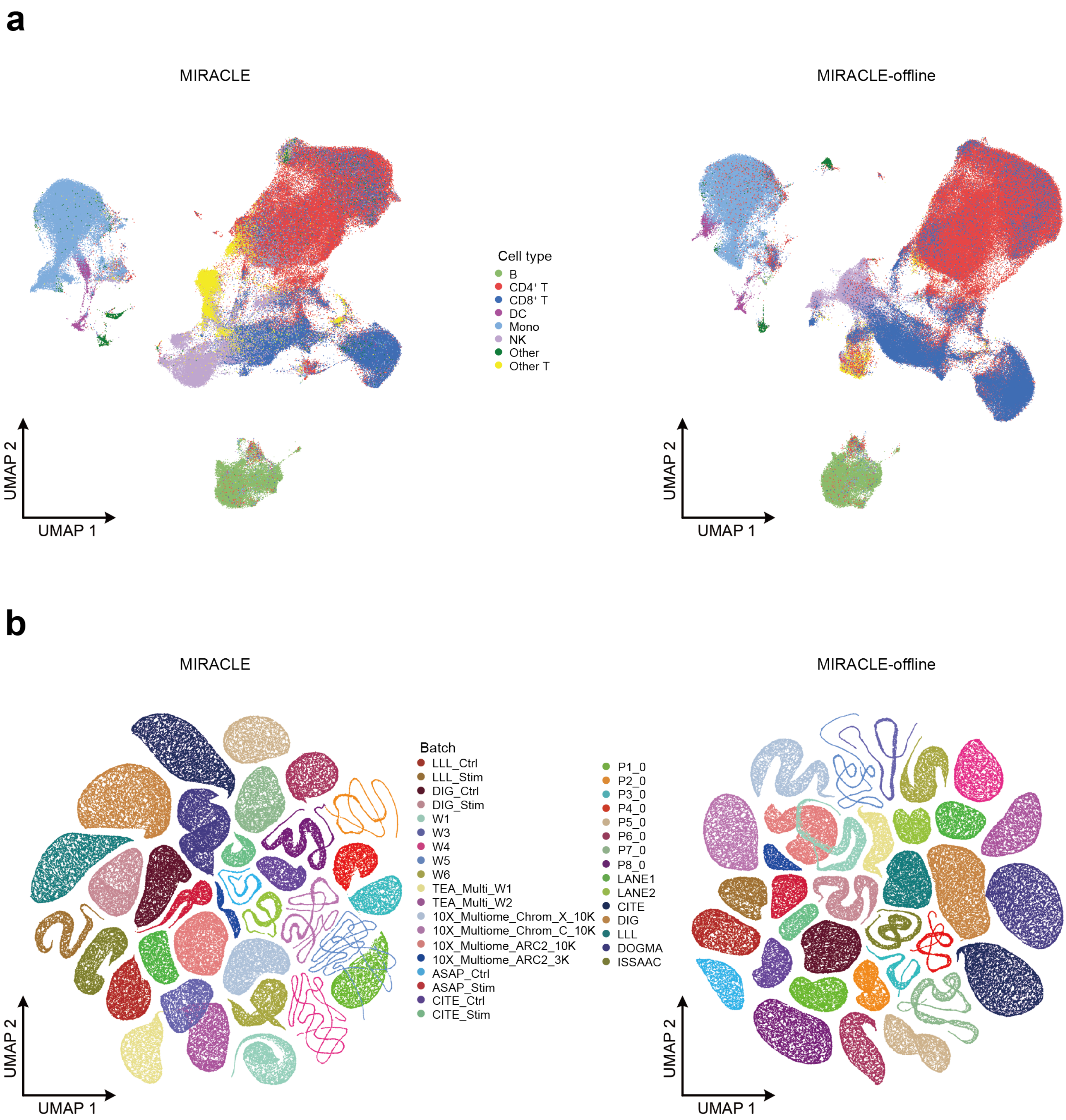

Supplementary Fig. 15: UMAP visualization (merged view) of cell embeddings (a) and technical noise (b) inferred by MIRACLE (left) and MIRACLE-offline (right) on the PBMC trimodal mosaic dataset containing 34 batches. Cells are colored by cell type (a) and batch (b), with cell types derived from Seurat labeling.

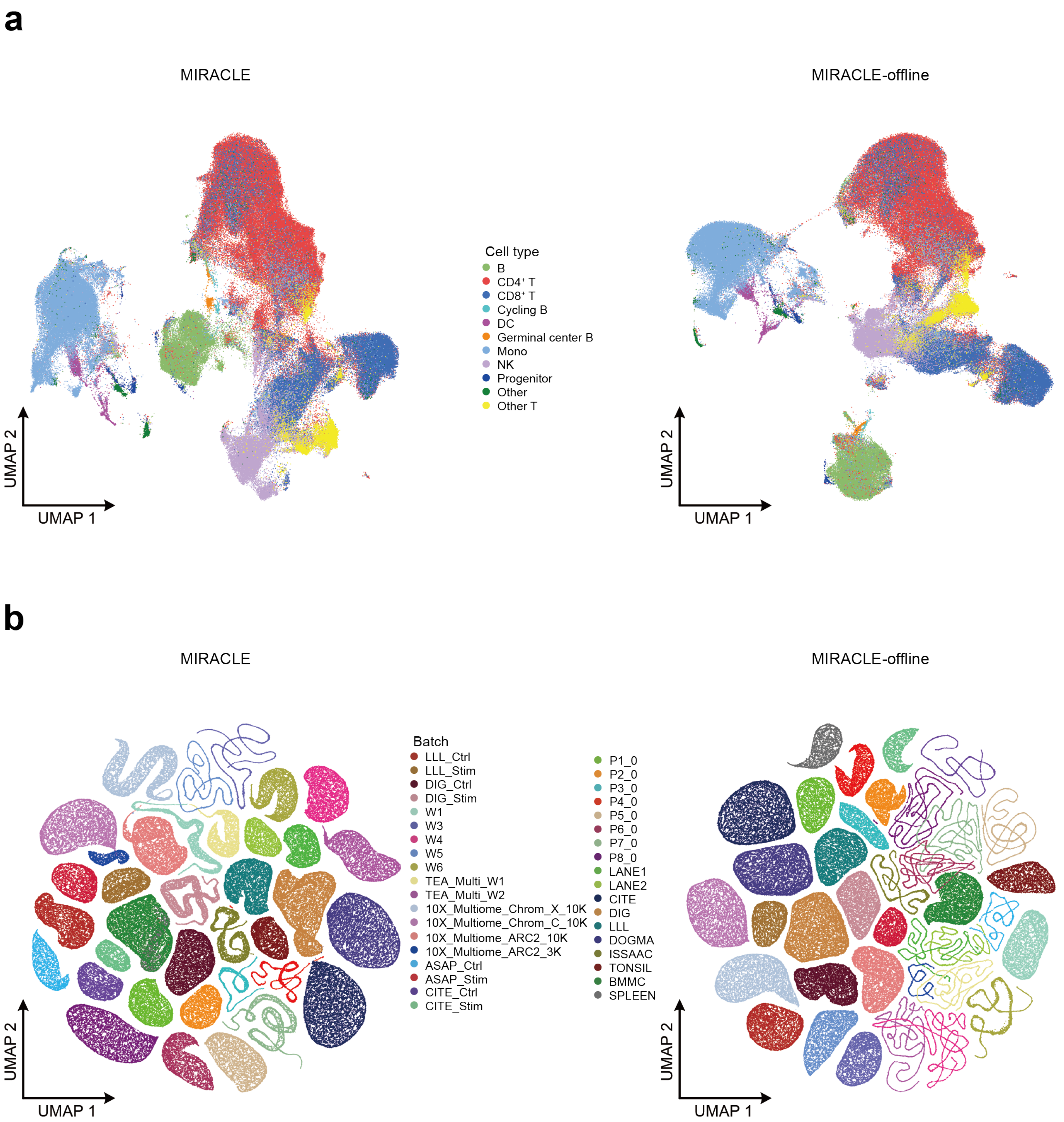

Supplementary Fig. 16: UMAP visualization (merged view) of cell embeddings (a) and technical noise (b) inferred by MIRACLE (left) and MIRACLE-offline (right) on the cross-tissue (PBMC, tonsil, BMMC, spleen) trimodal mosaic dataset containing 37 batches. Cells are colored by cell type (a) and batch (b), with cell types derived from Seurat and SingleR labeling.

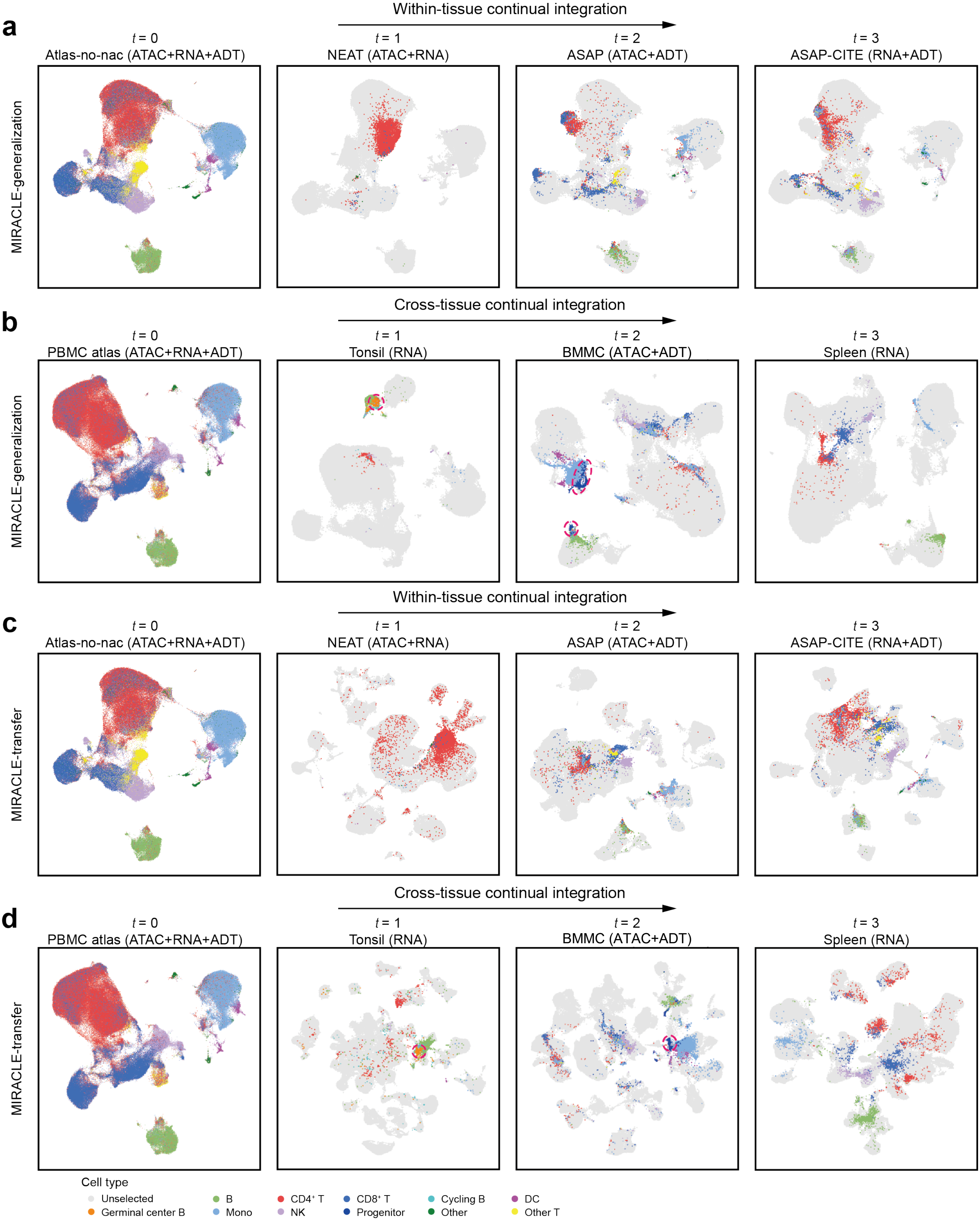

Supplementary Fig. 17: UMAP visualization of the cell embeddings inferred by MIRACLE-generalization (a,b) and MIRACLE-transfer (c,d) during within-tissue (a,c) and cross-tissue (b,d) continual atlas construction. Novel cell types are highlighted (red circles).

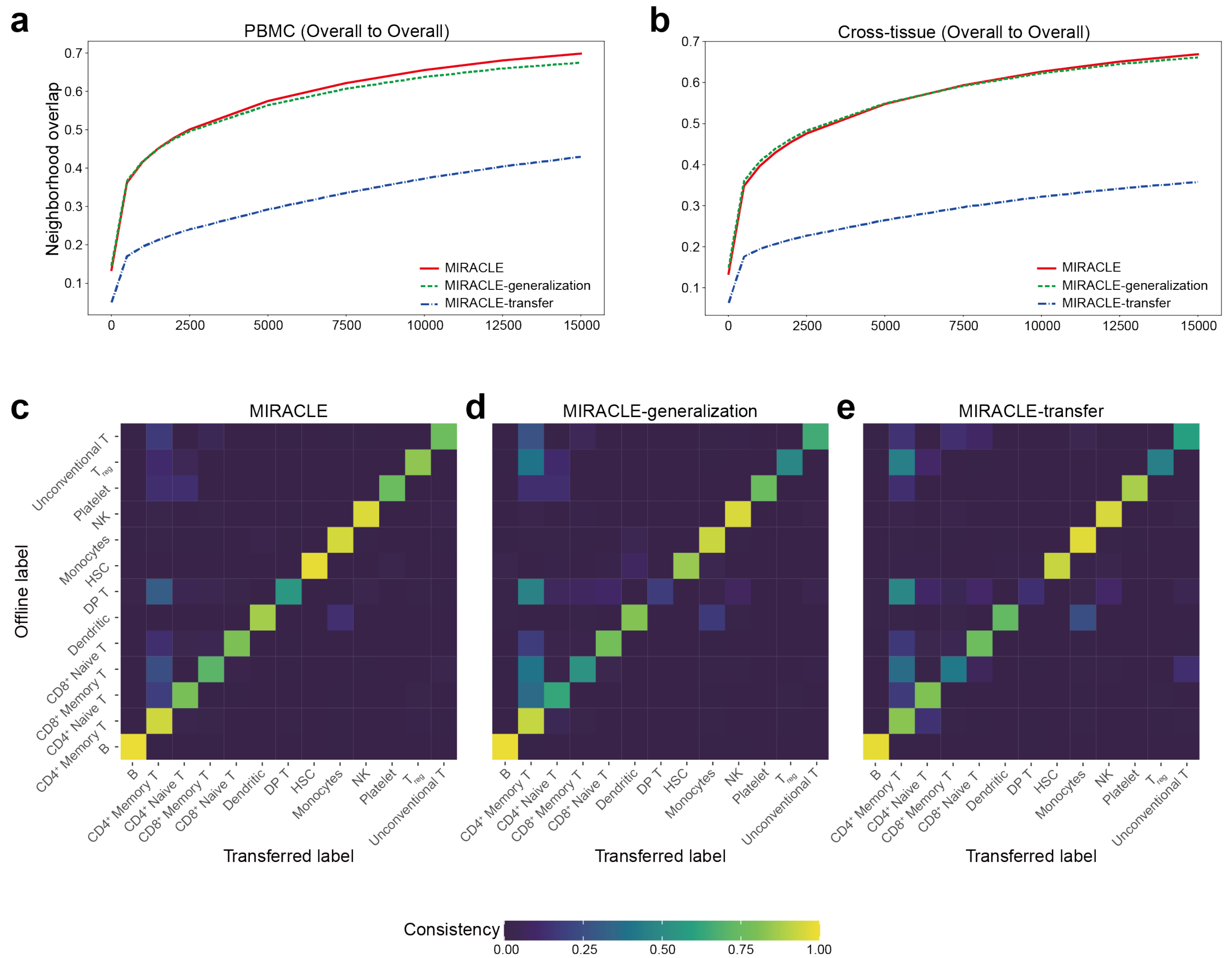

Supplementary Fig. 18: Neighborhood overlap analysis and label transfer confusion plots comparing MIRACLE's online and offline integration results. a, b, Neighborhood overlap between the dimensionality reduction results of online strategies and MIRACLE-offline across different neighborhood sizes in within-tissue (a) and cross-tissue (b) continual integration, measured by the identification overlap of overall cells' nearest neighbors. **c–e**, Label confusion plots comparing transfer consistency for MIRACLE (c), MIRACLE-generalization (d), and MIRACLE-transfer (e) in within-tissue continual integration.

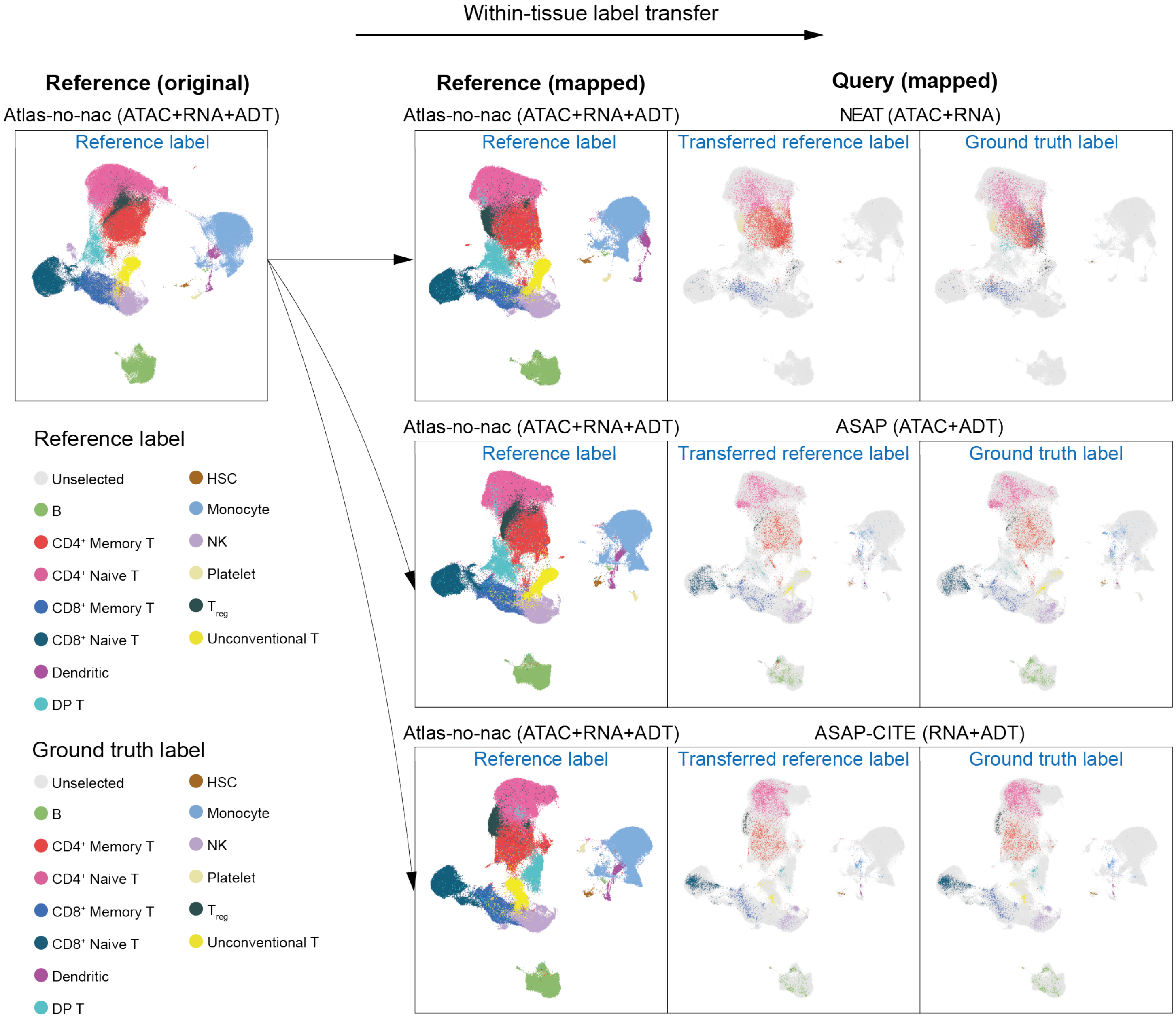

Supplementary Fig. 19: UMAP visualization of MIRACLE-inferred cell embeddings for within-tissue label transfer. Column 1: original PBMC reference colored by reference labels. Column 2: mapped PBMC references after separately incorporating different PBMC queries, colored by reference labels. Columns 3 and 4: the three mapped PBMC queries, colored by transferred reference labels (Column 3) and manually-annotated ground truth labels (Column 4).

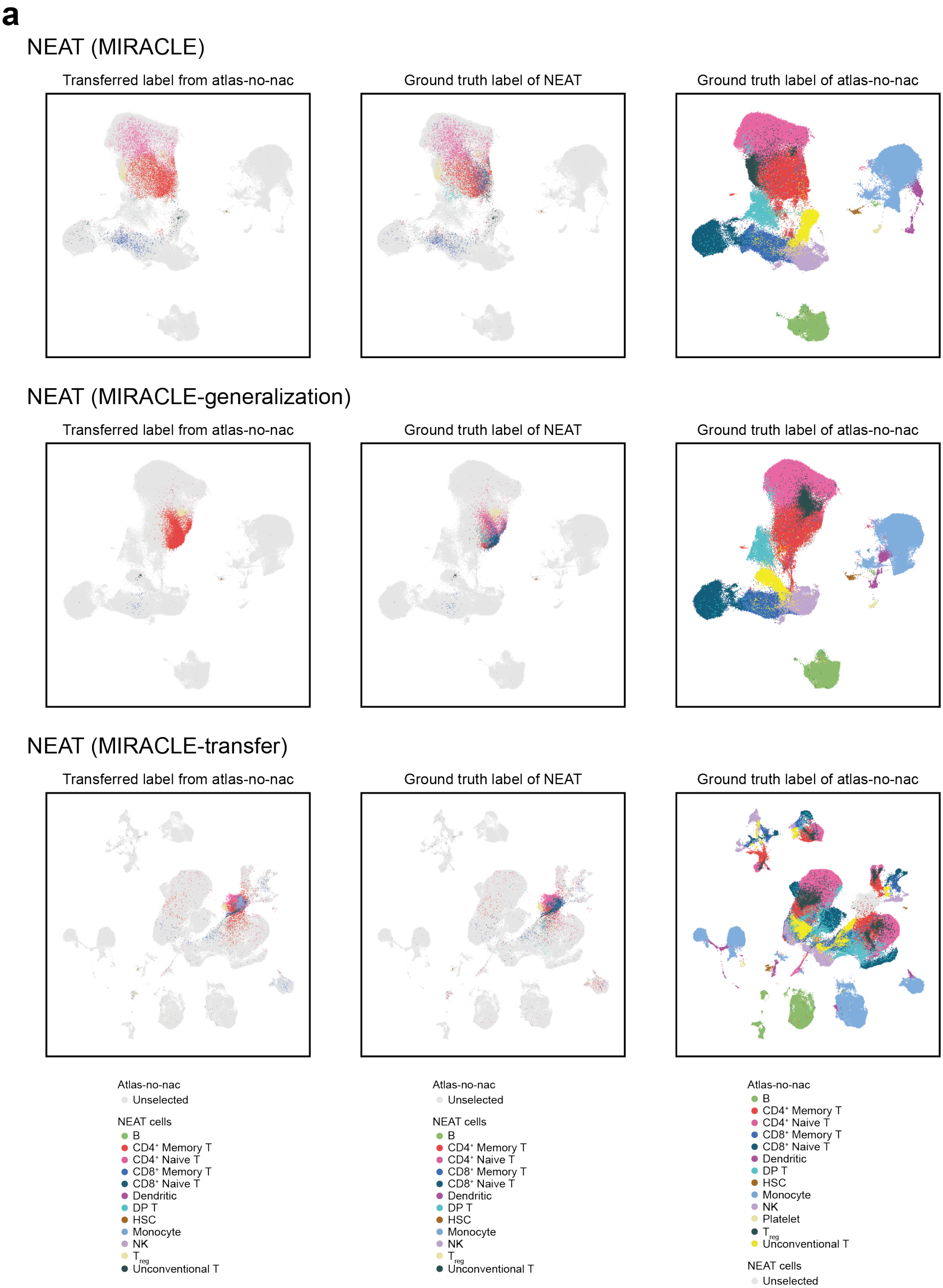
a
NEAT (MIRACLE)
Transferred label from atlas-no-nac
Ground truth label of NEAT
Ground truth label of atlas-no-nac
NEAT (MIRACLE-generalization)
Transferred label from atlas-no-nac
Ground truth label of NEAT
Ground truth label of atlas-no-nac
NEAT (MIRACLE-transfer)
Transferred label from atlas-no-nac
Ground truth label of NEAT
Ground truth label of atlas-no-nac
Atlas-no-nac
Unselected
NEAT cells
B
CD4+ Memory T
CD4+ Naive T
CD8+ Memory T
CD8+ Naive T
Dendritic
DP T
HSC
Monocyte
NK
Treg
Unconventional T
Atlas-no-nac
Unselected
NEAT cells
B
CD4+ Memory T
CD4+ Naive T
CD8+ Memory T
CD8+ Naive T
Dendritic
DP T
HSC
Monocyte
NK
Treg
Unconventional T
Atlas-no-nac
B
CD4+ Memory T
CD4+ Naive T
CD8+ Memory T
CD8+ Naive T
Dendritic
DP T
HSC
Monocyte
NK
Platelet
Treg
Unconventional T
NEAT cells
Unselected

b

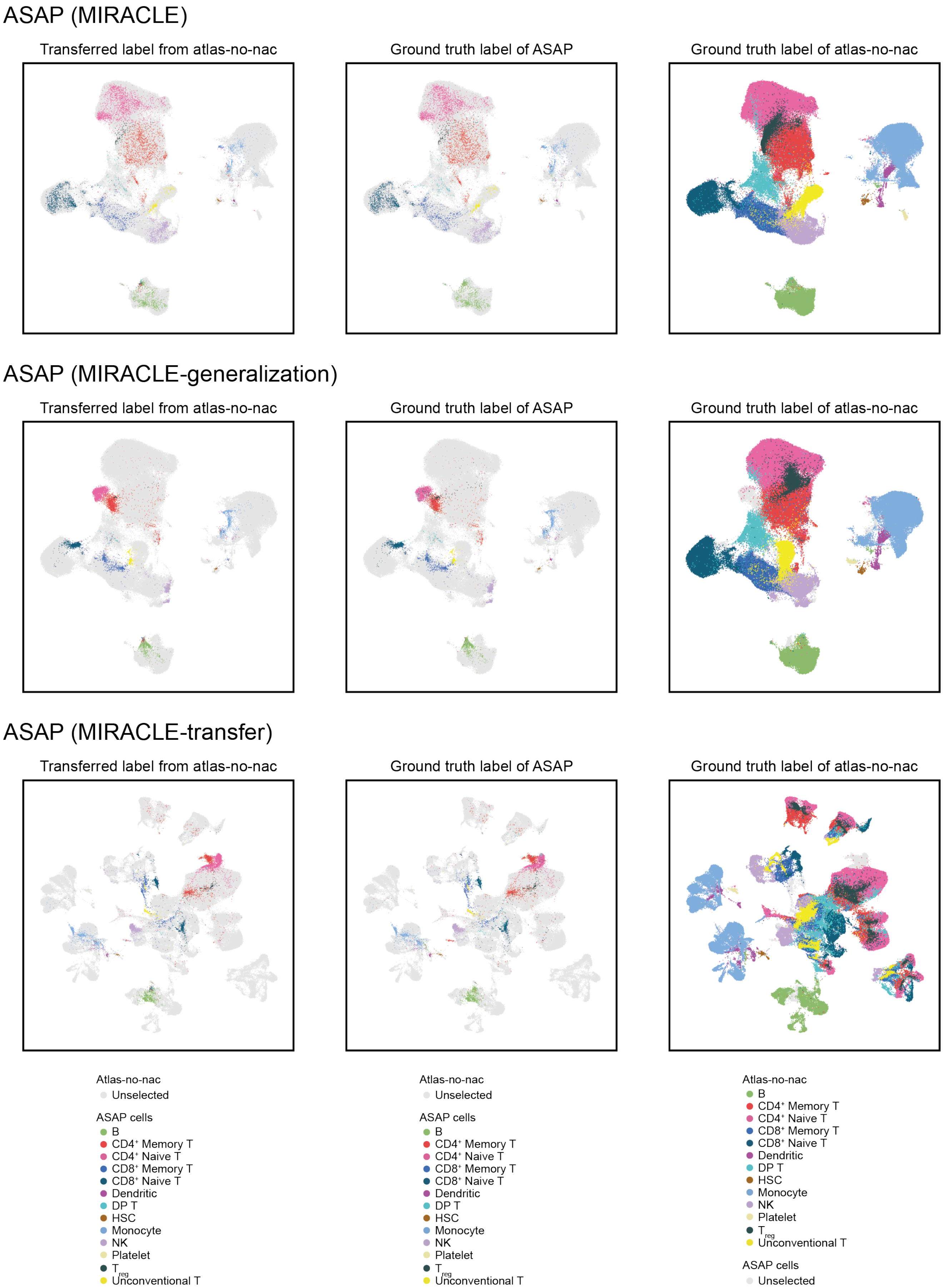

ASAP (MIRACLE)
Transferred label from atlas-no-nac
Ground truth label of ASAP
Ground truth label of atlas-no-nac
ASAP (MIRACLE-generalization)
Transferred label from atlas-no-nac
Ground truth label of ASAP
Ground truth label of atlas-no-nac
ASAP (MIRACLE-transfer)
Transferred label from atlas-no-nac
Ground truth label of ASAP
Ground truth label of atlas-no-nac
Atlas-no-nac
Unselected
ASAP cells
B
CD4+ Memory T
CD4+ Naive T
CD8+ Memory T
CD8+ Naive T
Dendritic
DP T
HSC
Monocyte
NK
Platelet
Treg
Unconventional T
Atlas-no-nac
Unselected
ASAP cells
B
CD4+ Memory T
CD4+ Naive T
CD8+ Memory T
CD8+ Naive T
Dendritic
DP T
HSC
Monocyte
NK
Platelet
Treg
Unconventional T
Atlas-no-nac
B
CD4+ Memory T
CD4+ Naive T
CD8+ Memory T
CD8+ Naive T
Dendritic
DP T
HSC
Monocyte
NK
Platelet
Treg
Unconventional T
ASAP cells
Unselected

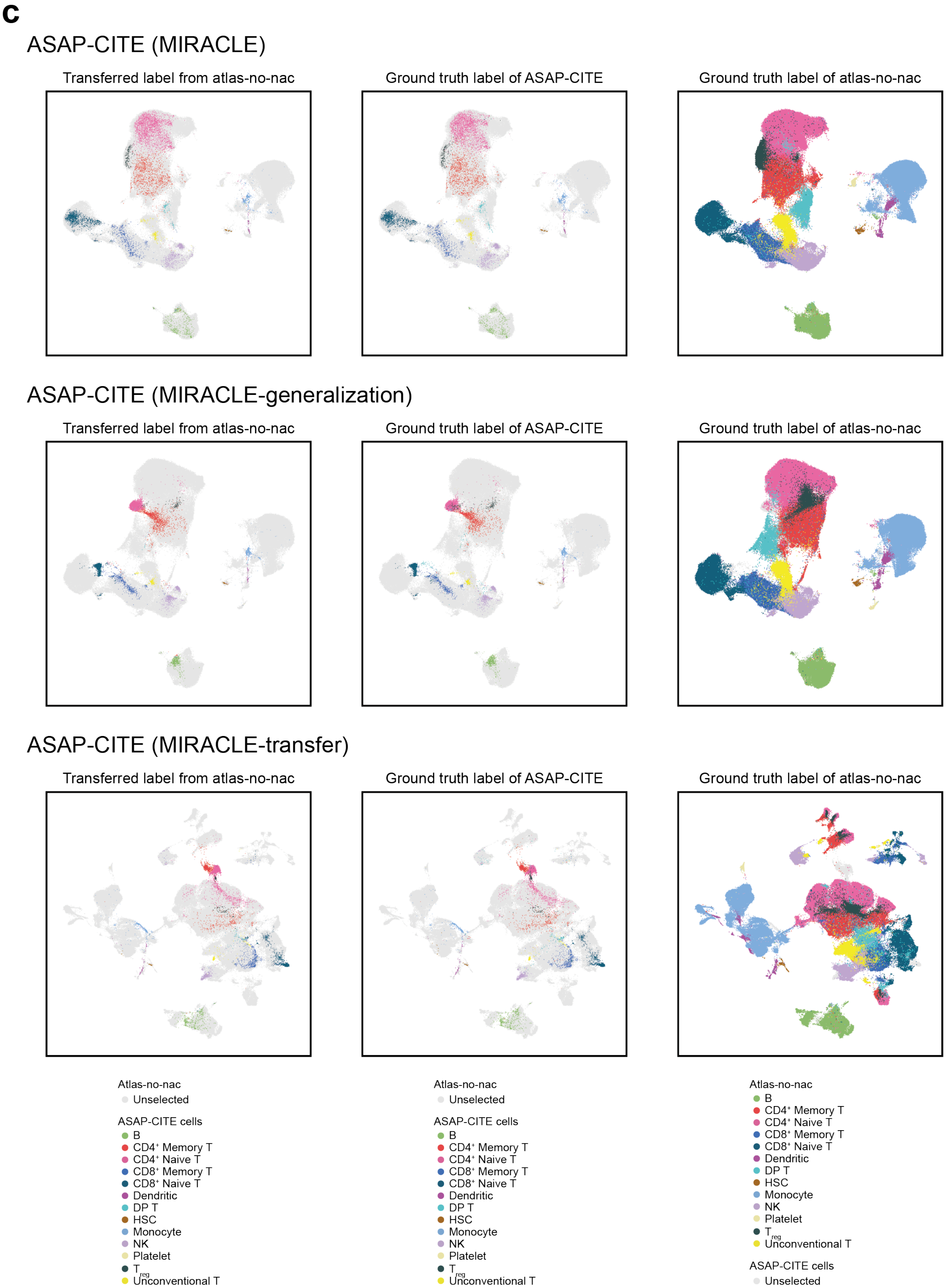

Supplementary Fig. 20: UMAP visualization of cell embeddings inferred by different MIRACLE online strategies for within-tissue label transfer. Columns 1 and 2: mapped PBMC queries, colored by transferred reference labels (Column 1) and manually-annotated ground truth labels (Column 2). Column 3: mapped PBMC references after incorporating the PBMC queries, colored by reference labels.

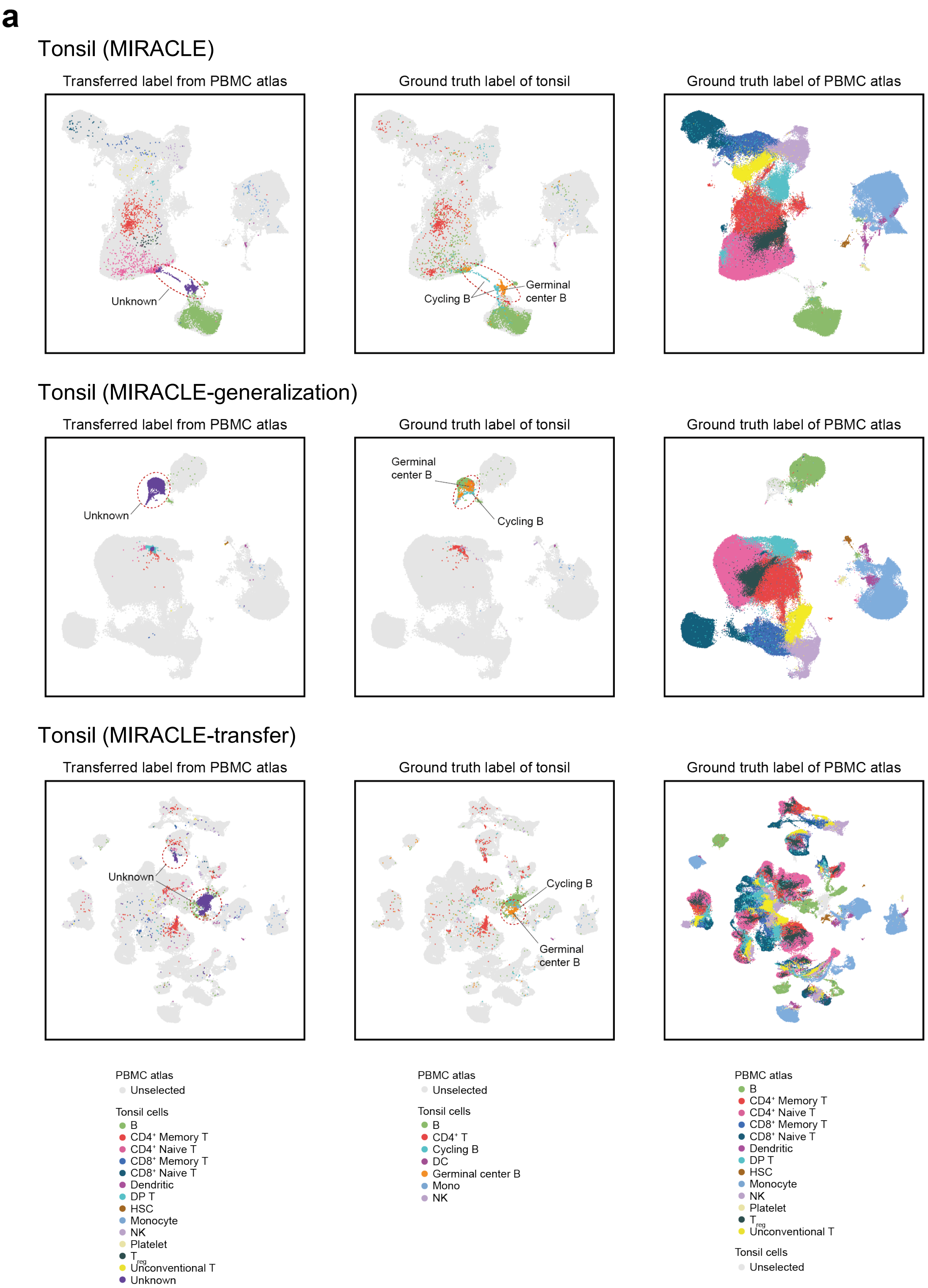
a
Tonsil (MIRACLE)
Transferred label from PBMC atlas
Unknown
Ground truth label of tonsil
Cycling B
Germinal center B
Ground truth label of PBMC atlas
Tonsil (MIRACLE-generalization)
Transferred label from PBMC atlas
Unknown
Ground truth label of tonsil
Germinal center B
Cycling B
Ground truth label of PBMC atlas
Tonsil (MIRACLE-transfer)
Transferred label from PBMC atlas
Unknown
Ground truth label of tonsil
Cycling B
Germinal center B
Ground truth label of PBMC atlas
PBMC atlas
Unselected
Tonsil cells
B
$CD4^{+}$ Memory T
$CD4^{+}$ Naive T
$CD8^{+}$ Memory T
$CD8^{+}$ Naive T
Dendritic
DP T
HSC
Monocyte
NK
Platelet
T_{reg}
Unconventional T
Unknown
PBMC atlas
Unselected
Tonsil cells
B
$CD4^{+}$ T
Cycling B
DC
Germinal center B
Mono
NK
PBMC atlas
B
$CD4^{+}$ Memory T
$CD4^{+}$ Naive T
$CD8^{+}$ Memory T
$CD8^{+}$ Naive T
Dendritic
DP T
HSC
Monocyte
NK
Platelet
T_{reg}
Unconventional T
Tonsil cells
Unselected

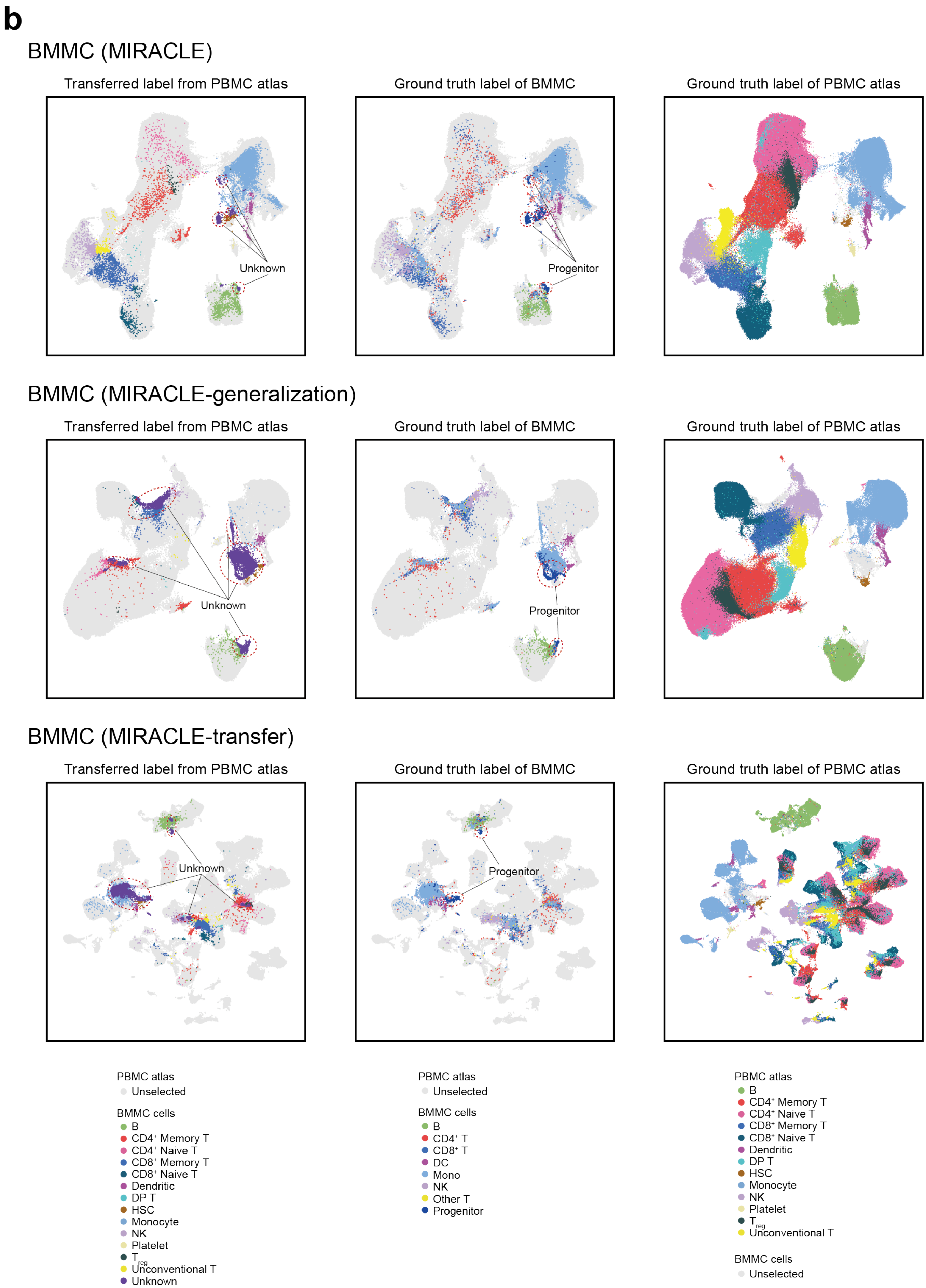
b
BMMC (MIRACLE)
Transferred label from PBMC atlas
Unknown
Ground truth label of BMMC
Progenitor
Ground truth label of PBMC atlas
BMMC (MIRACLE-generalization)
Transferred label from PBMC atlas
Unknown
Ground truth label of BMMC
Progenitor
Ground truth label of PBMC atlas
BMMC (MIRACLE-transfer)
Transferred label from PBMC atlas
Unknown
Ground truth label of BMMC
Progenitor
Ground truth label of PBMC atlas
PBMC atlas
Unselected
BMMC cells
B
CD4+ Memory T
CD4+ Naive T
CD8+ Memory T
CD8+ Naive T
Dendritic
DP T
HSC
Monocyte
NK
Platelet
T_{reg}
Unconventional T
Unknown
PBMC atlas
Unselected
BMMC cells
B
CD4+ T
CD8+ T
DC
Mono
NK
Other T
Progenitor
PBMC atlas
B
CD4+ Memory T
CD4+ Naive T
CD8+ Memory T
CD8+ Naive T
Dendritic
DP T
HSC
Monocyte
NK
Platelet
T_{reg}
Unconventional T
BMMC cells
Unselected

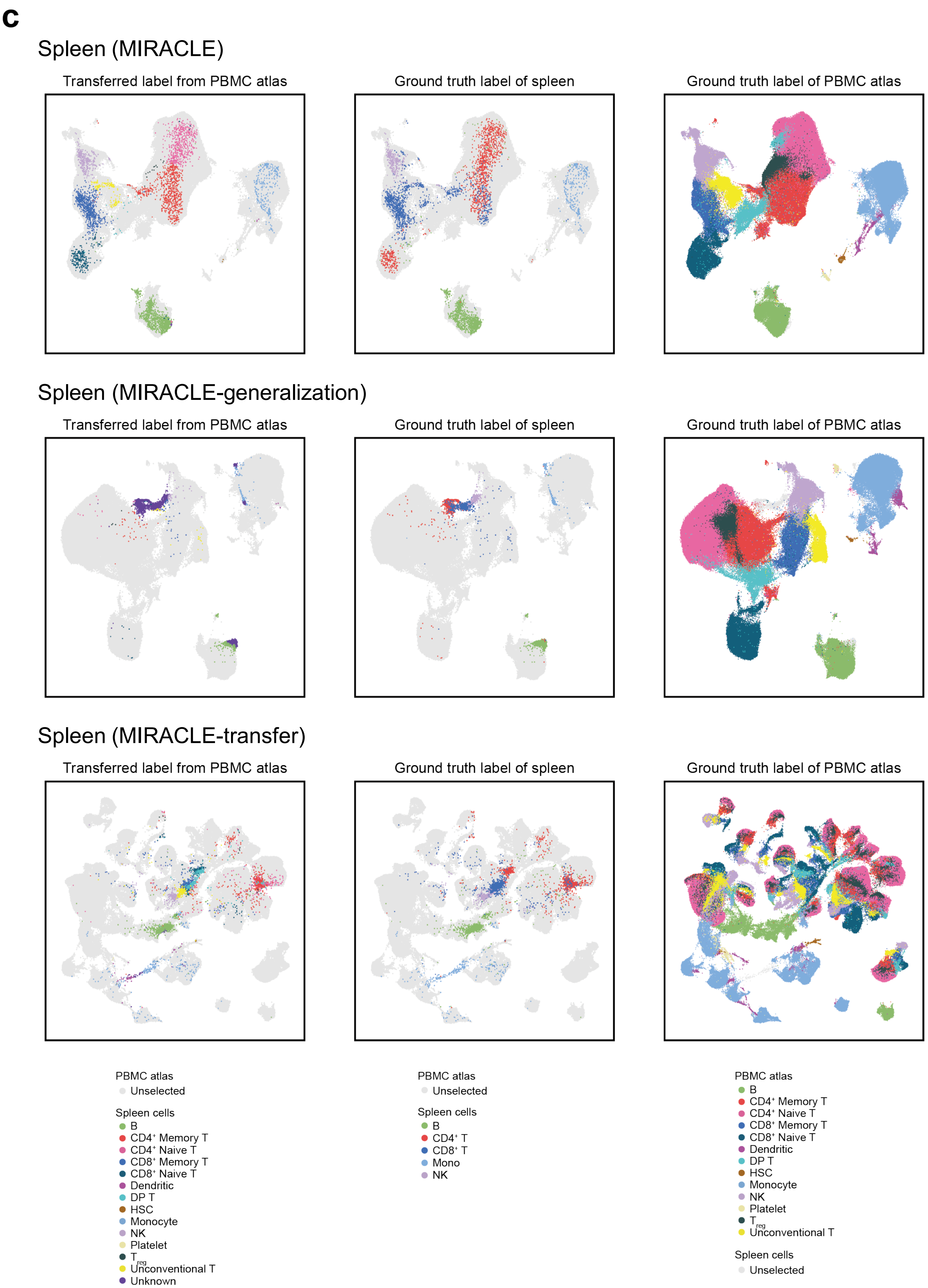

Supplementary Fig. 21: UMAP visualization of cell embeddings inferred by different MIRACLE online strategies for cross-tissue label transfer. Columns 1 and 2: mapped cross-tissue queries, colored by transferred reference labels (Column 1) and third-party ground truth labels (Column 2). Column 3: mapped PBMC references after incorporating the cross-tissue queries, colored by reference labels.

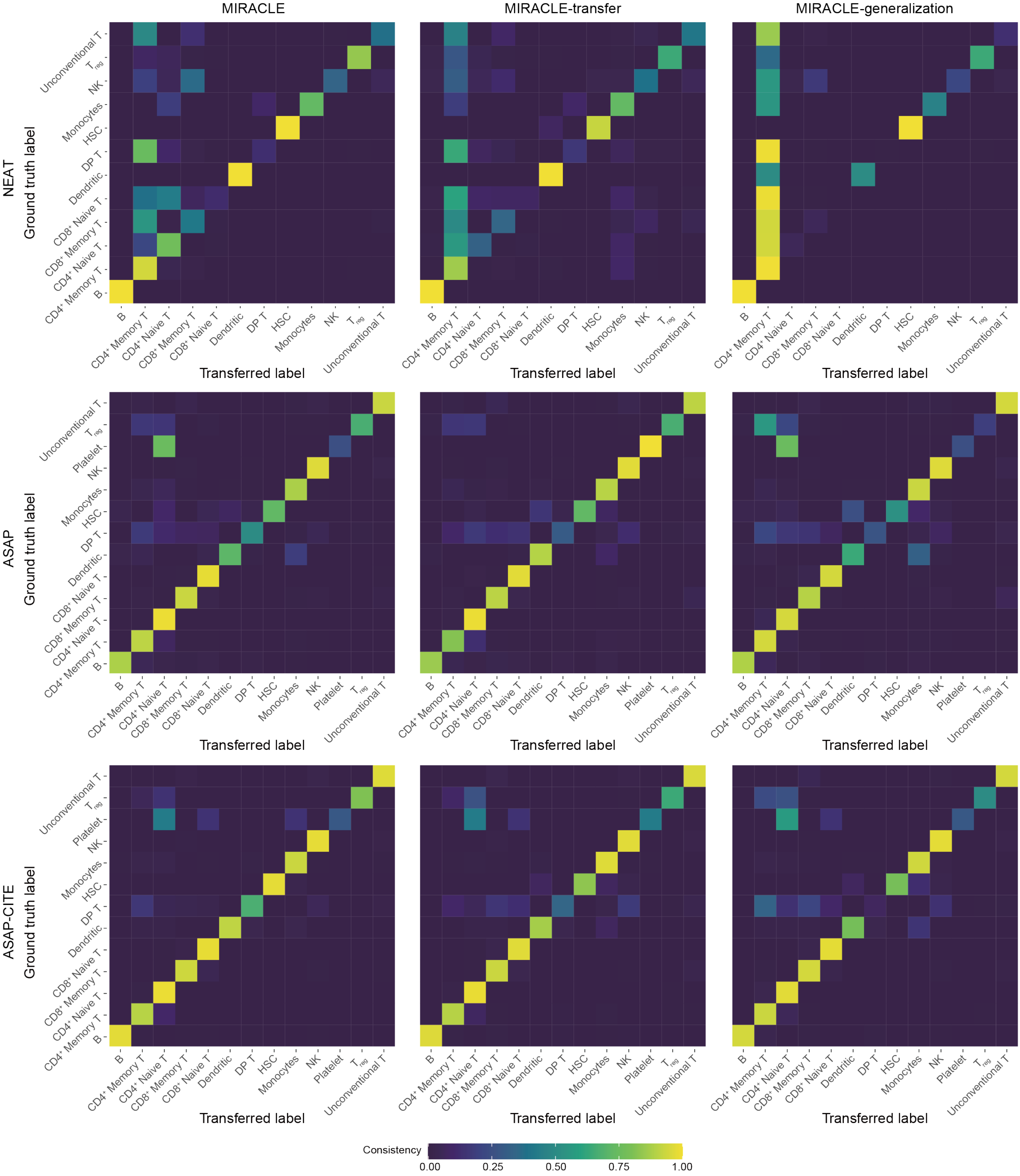

Supplementary Fig. 22: Confusion plots corresponding to the label transfer micro F1-scores for MIRACLE and two other strategies in within-tissue label transfer tasks.

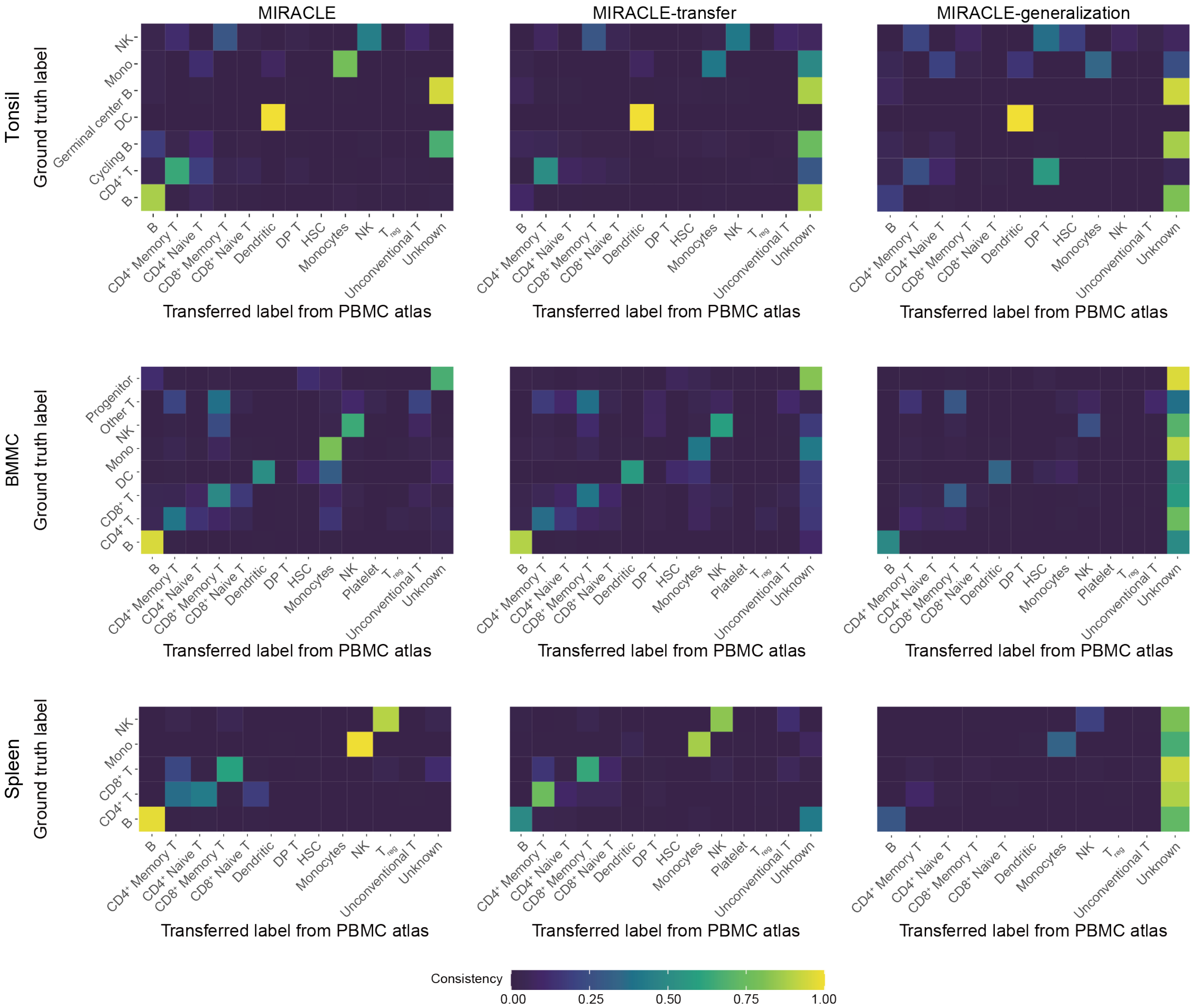

Supplementary Fig. 23: Confusion plots corresponding to the label transfer micro F1-scores for MIRACLE and two other strategies in cross-tissue label transfer tasks.

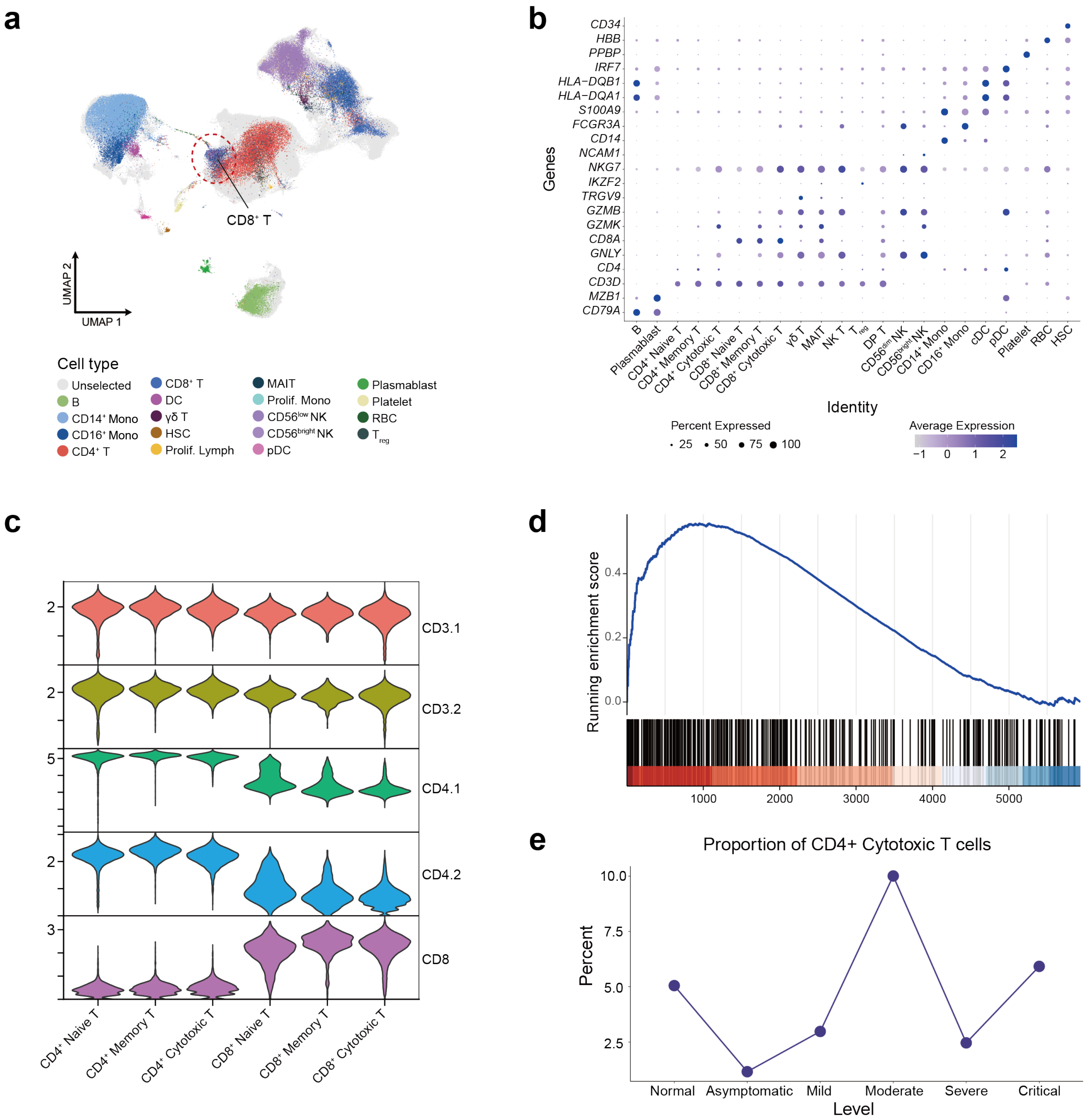

Supplementary Fig. 24: Multimodal characterization of CD4$^+$ cytotoxic T lymphocytes in COVID-19. a, UMAP visualization of MIRACLE-inferred cell embeddings of the integrated COVID-19 dataset, colored by labels from the original study. The region before major update to cell type is highlighted (red circle). **b**, Dot plot of marker gene expression across refined cell types in the integrated healthy and COVID-19 datasets. **c**, Violin plot of protein expression in COVID-19 T cells. **d**, Gene set enrichment analysis of cytotoxicity-related signature genes in CD4$^+$ CTLs. **e**, CD4$^+$ CTL proportions across severity statuses.

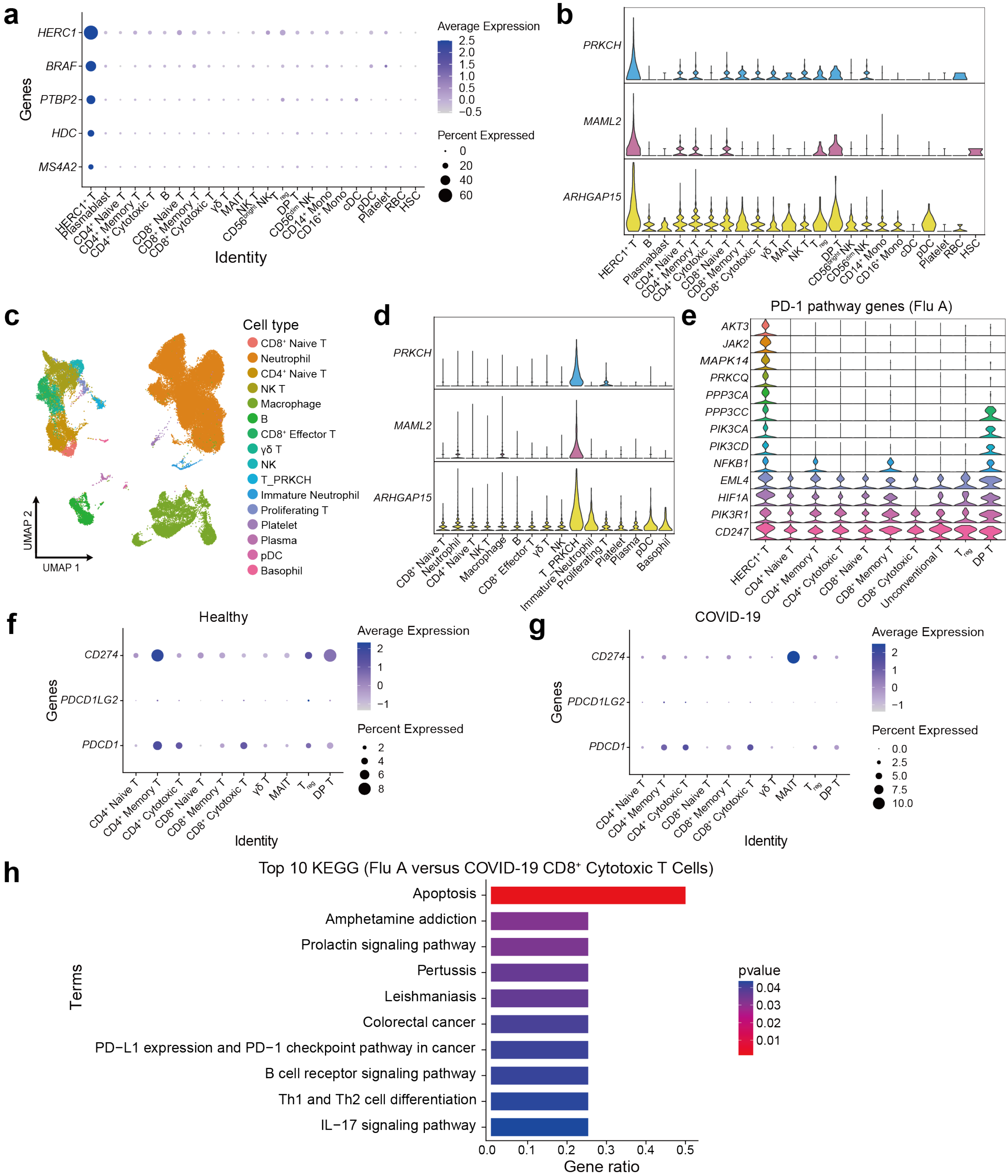

Supplementary Fig. 25: Identification of HERC1⁺ T cells and CD8⁺ T cell exhaustion in Flu A. a, Dot plot of top 5 marker genes in Flu A HERC1⁺ T cells. **b**, Violin plot of TCell_PRKCH subset (annotated from the original study) marker genes in the integrated Flu A dataset. **c**, UMAP reproduction of 7 Flu A samples according to the original study. **d**, Violin plot of TCell_PRKCH subset marker genes in the original Flu A dataset. **e**, Violin plot of PD-L1 expression and PD-1 checkpoint pathway related genes in Flu A T cells. **f**,**g**, Dot plots of *PDCD1* (PD-1), *CD274* (PD-L1), and *PDCD1LG2* (PD-L2) gene expression in healthy (**f**) and COVID-19 (**g**) T cells. **h**, Enrichment of T-cell exhaustion-related terms in Flu A versus COVID-19 CD8⁺ cytotoxic T cells.

a

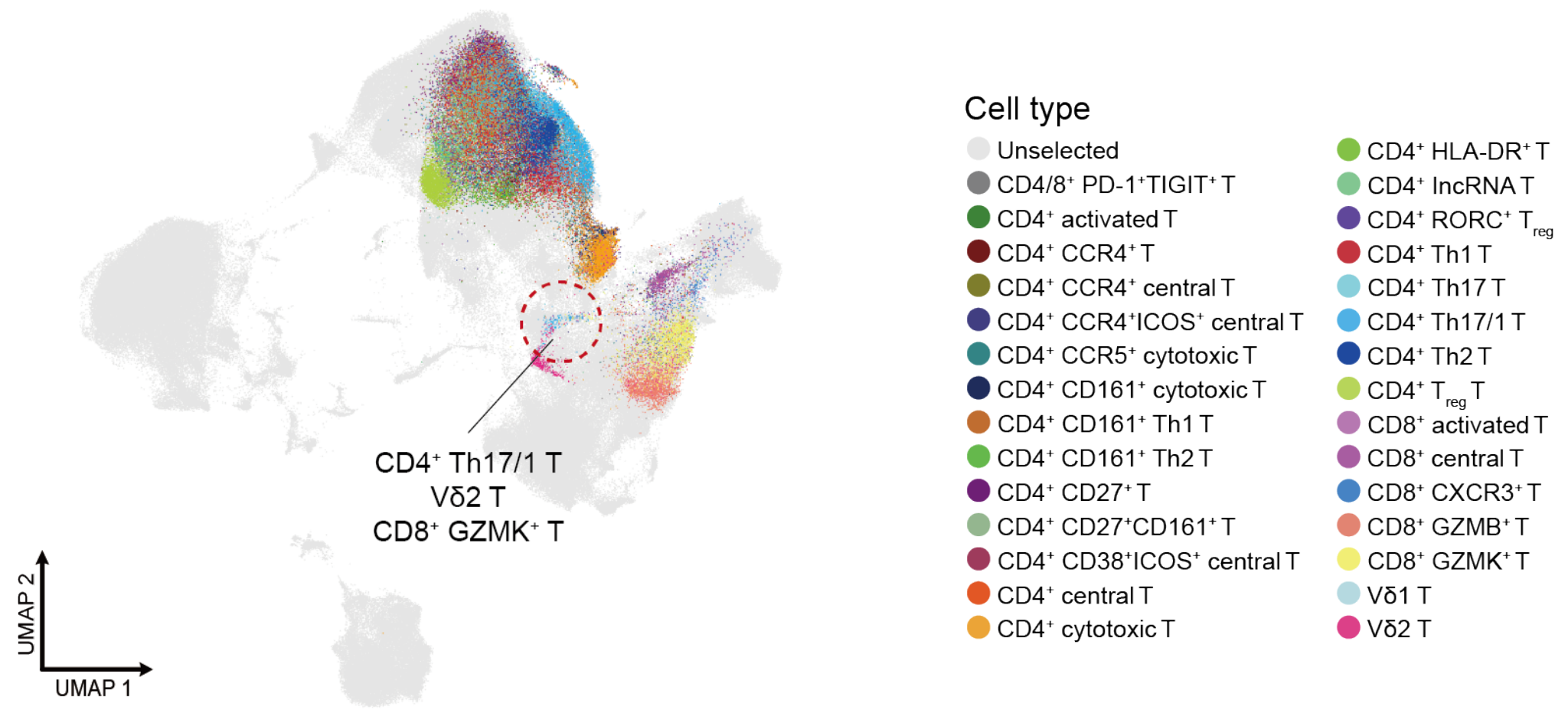
Cell type
Unselected
CD4/8⁺ PD-1⁺TIGIT⁺ T
CD4⁺ activated T
CD4⁺ CCR4⁺ T
CD4⁺ CCR4⁺ central T
CD4⁺ CCR4⁺ICOS⁺ central T
CD4⁺ CCR5⁺ cytotoxic T
CD4⁺ CD161⁺ cytotoxic T
CD4⁺ CD161⁺ Th1 T
CD4⁺ CD161⁺ Th2 T
CD4⁺ CD27⁺ T
CD4⁺ CD27⁺CD161⁺ T
CD4⁺ CD38⁺ICOS⁺ central T
CD4⁺ central T
CD4⁺ cytotoxic T
CD4⁺ HLA-DR⁺ T
CD4⁺ lncRNA T
CD4⁺ RORC⁺ Treg
CD4⁺ Th1 T
CD4⁺ Th17 T
CD4⁺ Th17/1 T
CD4⁺ Th2 T
CD4⁺ Treg T
CD8⁺ activated T
CD8⁺ central T
CD8⁺ CXCR3⁺ T
CD8⁺ GZMB⁺ T
CD8⁺ GZMK⁺ T
Vδ1 T
Vδ2 T
CD4⁺ Th17/1 T
Vδ2 T
CD8⁺ GZMK⁺ T
UMAP 2
UMAP 1

b

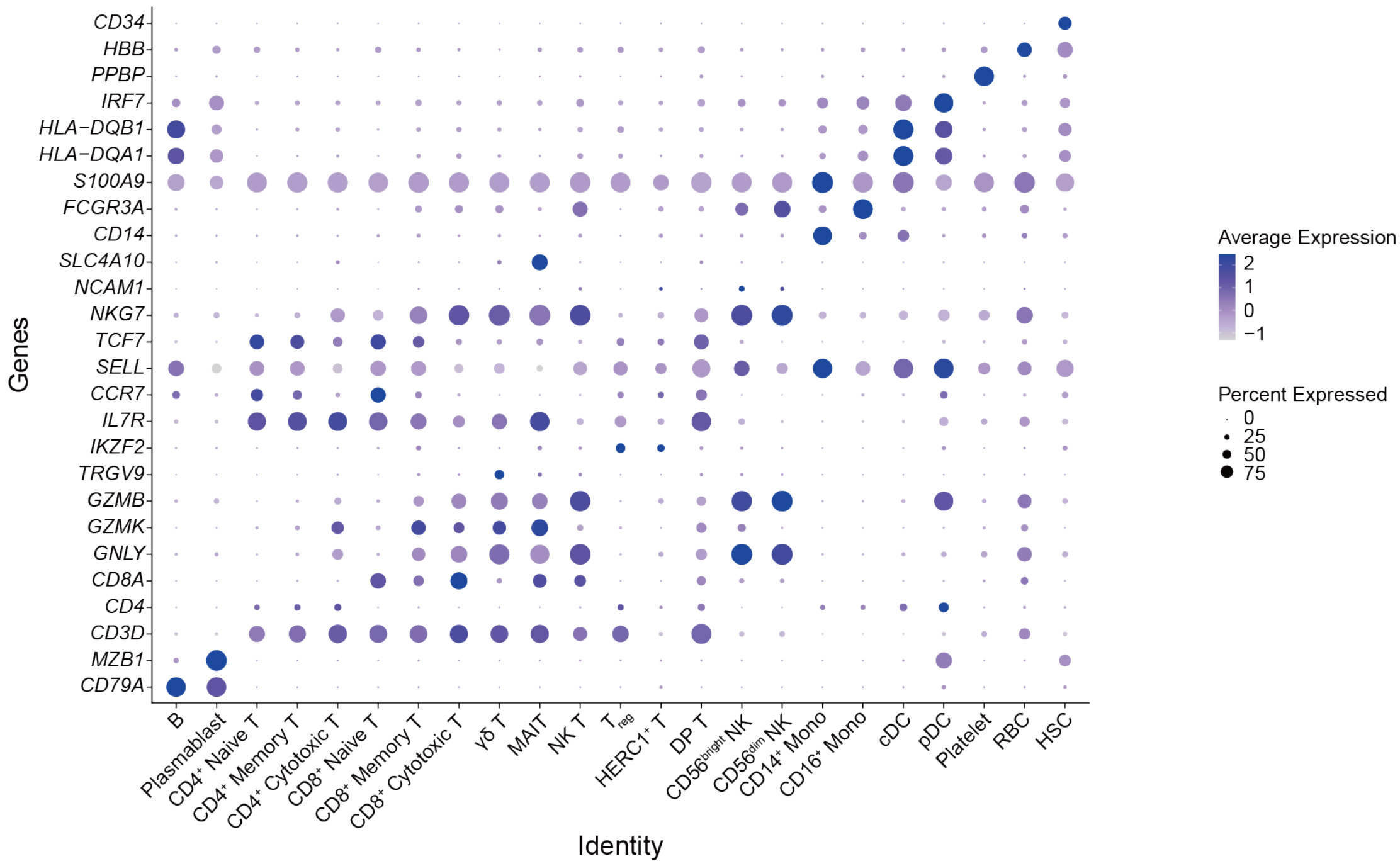
Genes
CD34
HBB
PPBP
IRF7
HLA−DQB1
HLA−DQA1
S100A9
FCGR3A
CD14
SLC4A10
NCAM1
NKG7
TCF7
SELL
CCR7
IL7R
IKZF2
TRGV9
GZMB
GZMK
GNLY
CD8A
CD4
CD3D
MZB1
CD79A
B
Plasmablast
CD4⁺ Naive T
CD4⁺ Memory T
CD4⁺ Cytotoxic T
CD8⁺ Naive T
CD8⁺ Memory T
CD8⁺ Cytotoxic T
γδ T
MAIT
NK T
Treg
HERC1⁺ T
DP T
CD56bright NK
CD56dim NK
CD14⁺ Mono
CD16⁺ Mono
cDC
pDC
Platelet
RBC
HSC
Identity
Average Expression
2
1
0
−1
Percent Expressed
0
25
50
75

c

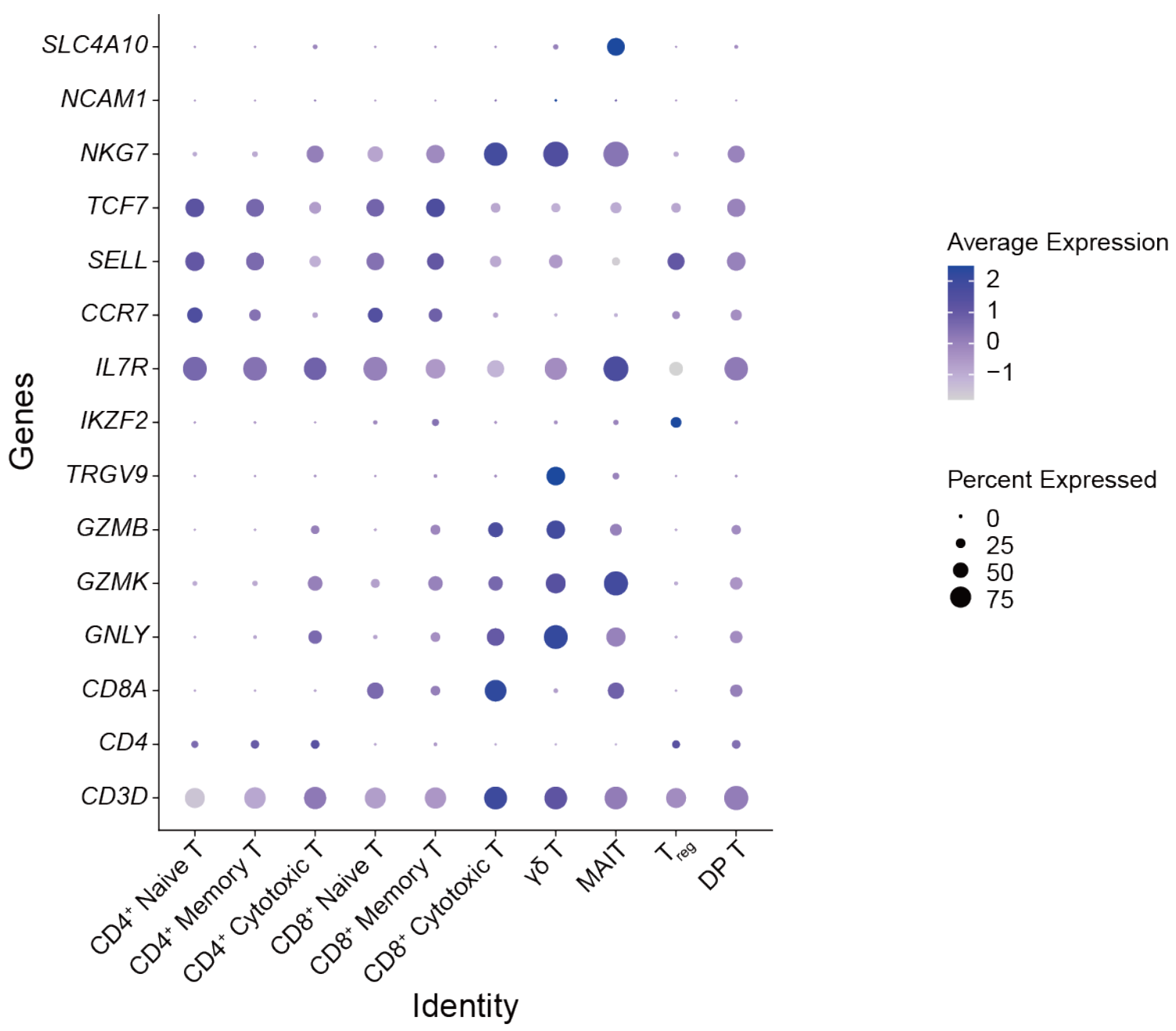
Genes
SLC4A10
NCAM1
NKG7
TCF7
SELL
CCR7
IL7R
IKZF2
TRGV9
GZMB
GZMK
GNLY
CD8A
CD4
CD3D
CD4⁺ Naive T
CD4⁺ Memory T
CD4⁺ Cytotoxic T
CD8⁺ Naive T
CD8⁺ Memory T
CD8⁺ Cytotoxic T
γδ T
MAIT
Treg
DP T
Identity
Average Expression
2
1
0
−1
Percent Expressed
0
25
50
75

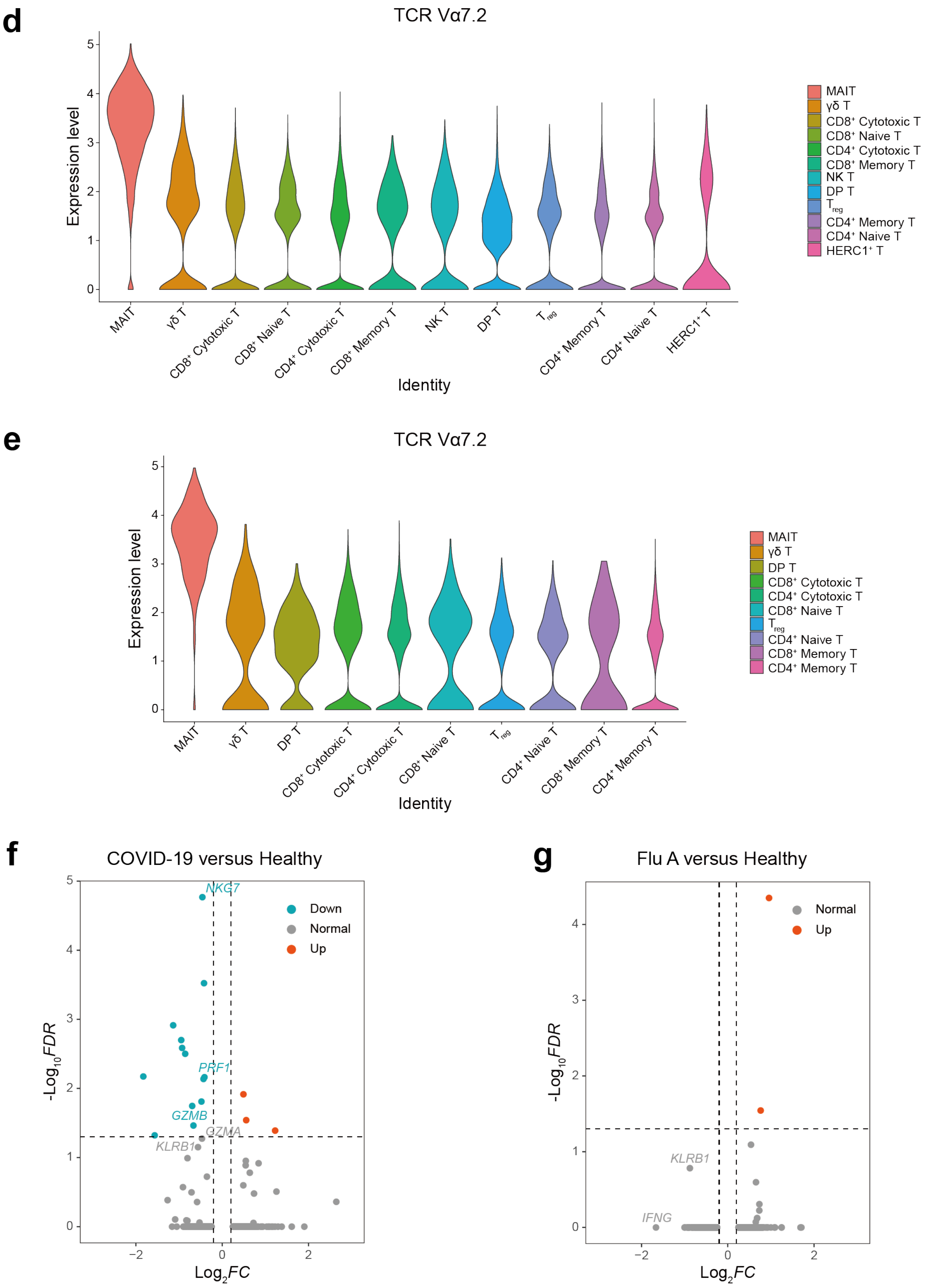

Supplementary Fig. 26: Refinement of T cell annotations and MAIT cell characterization across infections. a, UMAP visualization of MIRACLE-inferred cell embeddings of the integrated TB dataset, colored by labels from the original study. The region before major update to cell type is highlighted (red circle). **b**, Dot plot of marker gene expression across refined cell types in the integrated healthy, COVID-19, Flu A, and TB datasets. **c**, Dot plot of marker gene expression in TB T cells. **d**, Violin plot of TCR Vα7.2 protein expression of T cells in the integrated healthy, COVID-19, Flu A, and TB datasets. **e**, Violin plot of TCR Vα7.2 protein expression in TB T cells. **f**,**g**, Volcano plots of differentially expressed genes in MAIT cells, comparing COVID-19 versus healthy (**f**) and Flu A versus healthy (**g**) individuals.

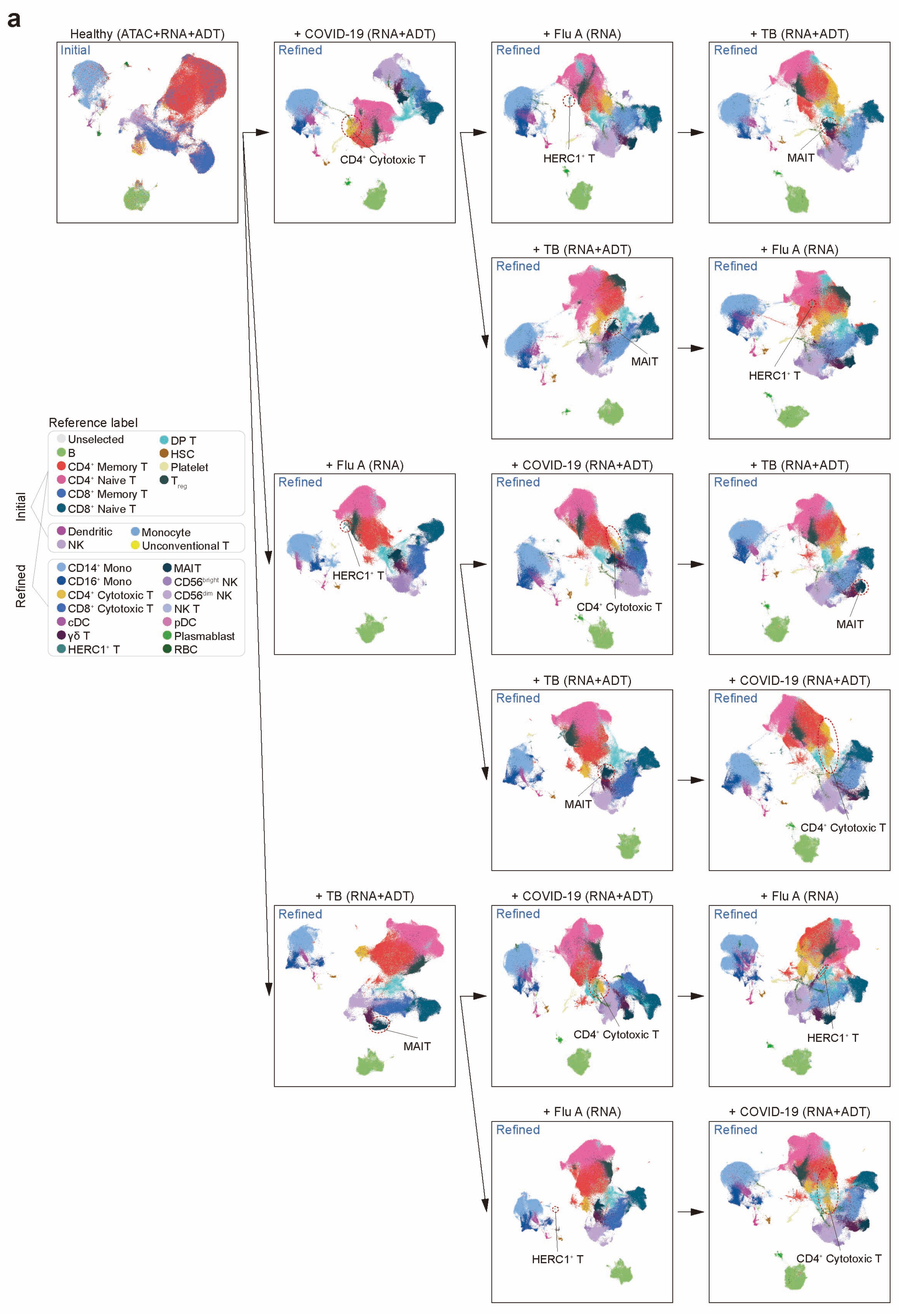

a
Healthy (ATAC+RNA+ADT)
Initial
+ COVID-19 (RNA+ADT)
Refined
CD4⁺ Cytotoxic T
+ Flu A (RNA)
HERC1⁺ T
+ TB (RNA+ADT)
MAIT
Reference label
Unselected
B
CD4⁺ Memory T
CD4⁺ Naive T
CD8⁺ Memory T
CD8⁺ Naive T
DP T
HSC
Platelet
Treg
Dendritic
NK
Monocyte
Unconventional T
CD14⁺ Mono
CD16⁺ Mono
CD4⁺ Cytotoxic T
CD8⁺ Cytotoxic T
cDC
γδ T
HERC1⁺ T
MAIT
CD56bright NK
CD56dim NK
NK T
pDC
Plasmablast
RBC
Initial
Refined

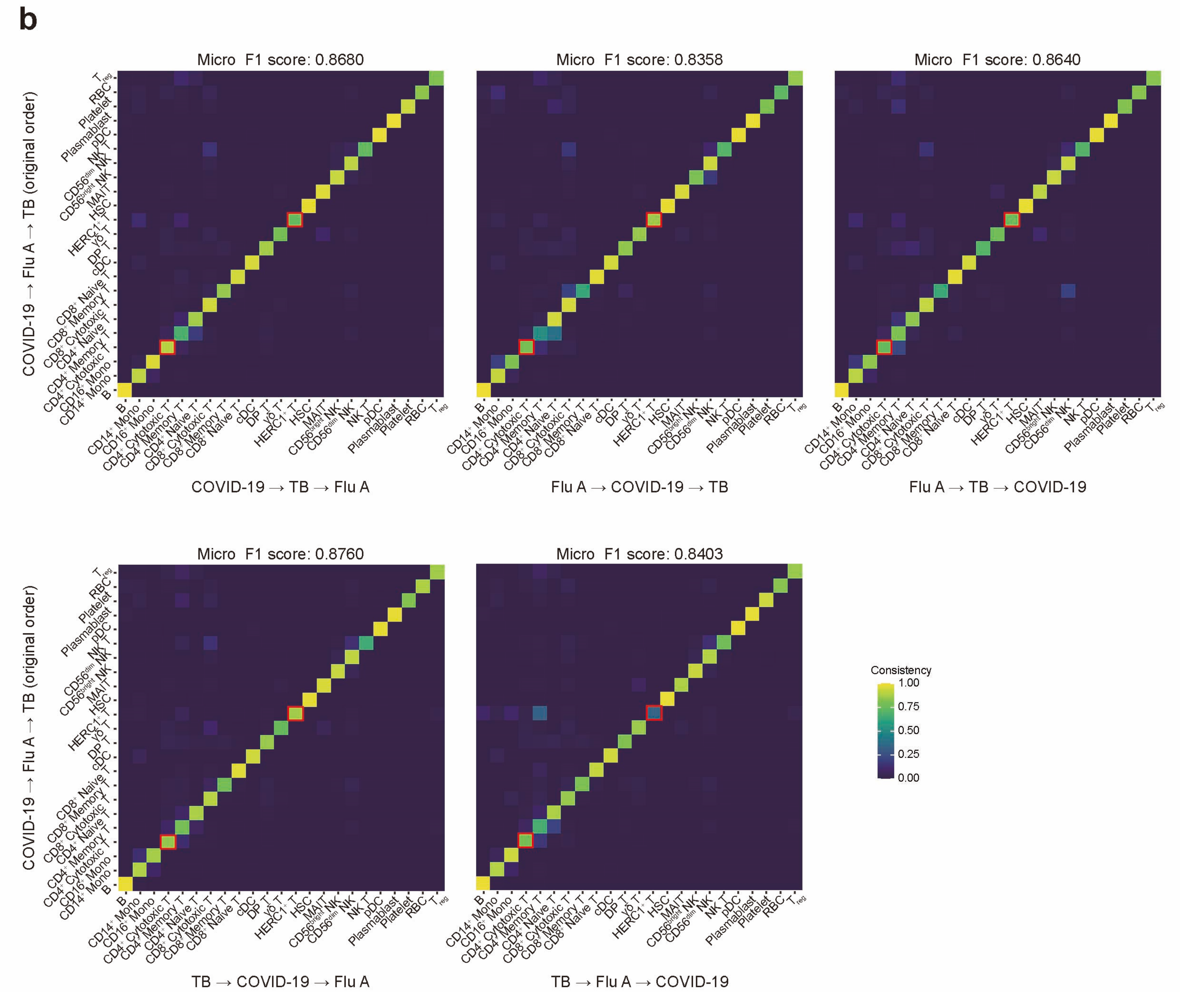

Supplementary Fig. 27: MIRACLE demonstrates robustness to the order of data integration. To evaluate MIRACLE's sensitivity to the sequence of data integration, we tested all six possible permutations of the disease integration order (COVID-19, Flu A, and TB) for the cross-disease atlas construction. **a**, UMAP visualizations of the final integrated atlases for each of the six integration orders. The original order used in the main text is presented in the first row, followed by the five permuted orders. The resulting cellular topologies are highly consistent across all permutations. Critically, key biological discoveries, including the expanded $CD4^+$ CTLs in COVID-19 and the $HERC1^+$ T cells in Flu A, were robustly identified in every sequence, confirming they are genuine biological signals rather than sequence-dependent artifacts. **b**, Confusion matrix comparing the final, manually-refined cell-type labels generated from each permuted integration order against the labels from the original order. The comparison shows high concordance, achieving a micro F1 score of 0.86 ± 0.02 (mean ± s.d. across the five permutations). This quantitative result confirms the stability of cell identity assignments regardless of the integration sequence. This overall robustness is attributed to MIRACLE's data rehearsal strategy, which mitigates dependency on the most recent task by continually revisiting a memory of previously integrated datasets.

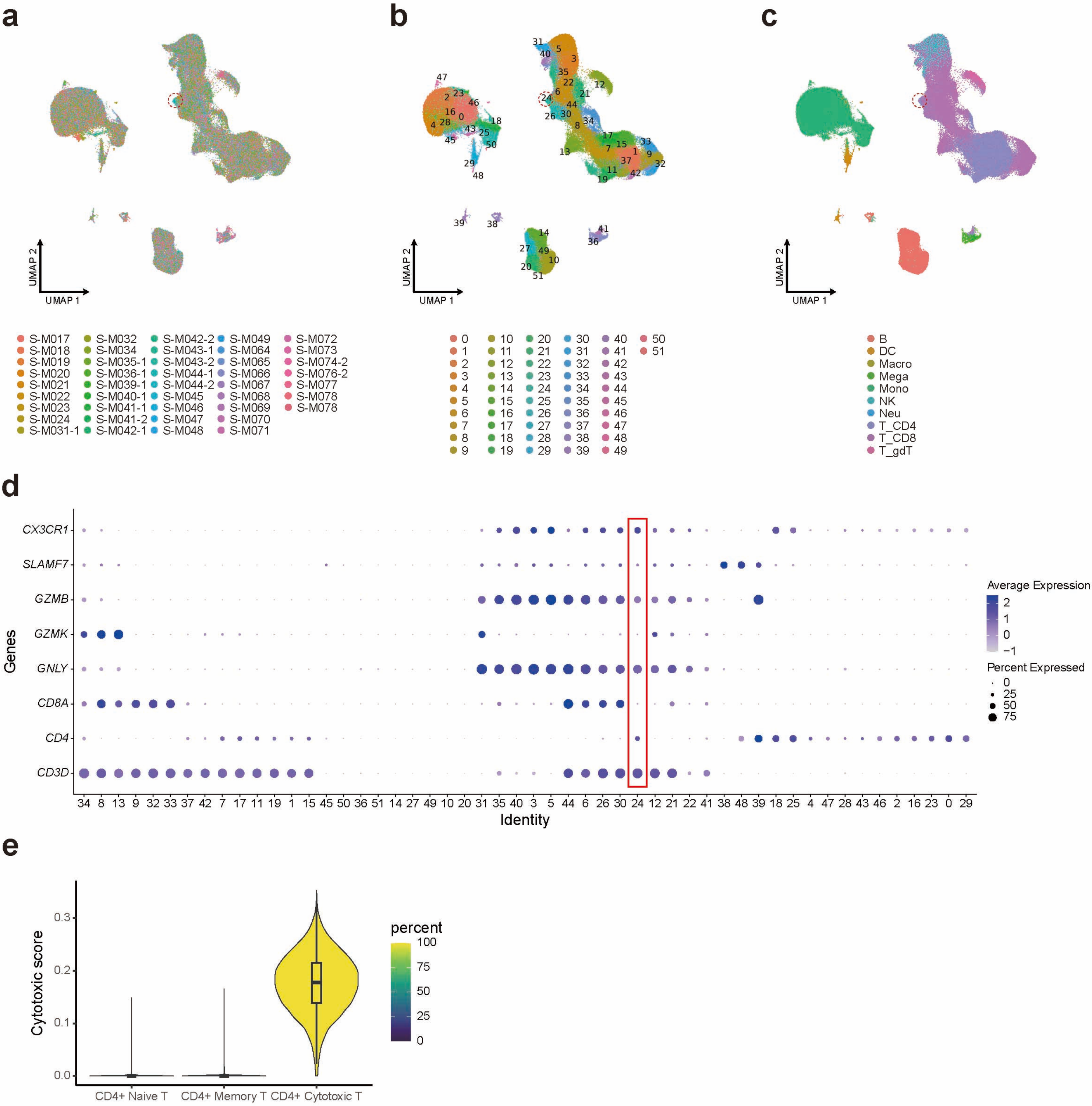

Supplementary Fig. 28: Validation of CD4⁺ CTLs in an independent COVID-19 dataset. **a–c**, UMAP plots of an independent COVID-19 dataset integrated by Harmony, colored by (a) batches, (b) clusters, and (c) original cell labels. **d**, Dot plot confirming co-expression of CD4⁺ T cell (*CD3D*, *CD4*) and cytotoxic (*GZMB*, *GNLY*) markers in Cluster 24. **e**, Box plot showing significantly higher cytotoxicity scores in CD4⁺ CTLs compared to other CD4⁺ T cell subsets.

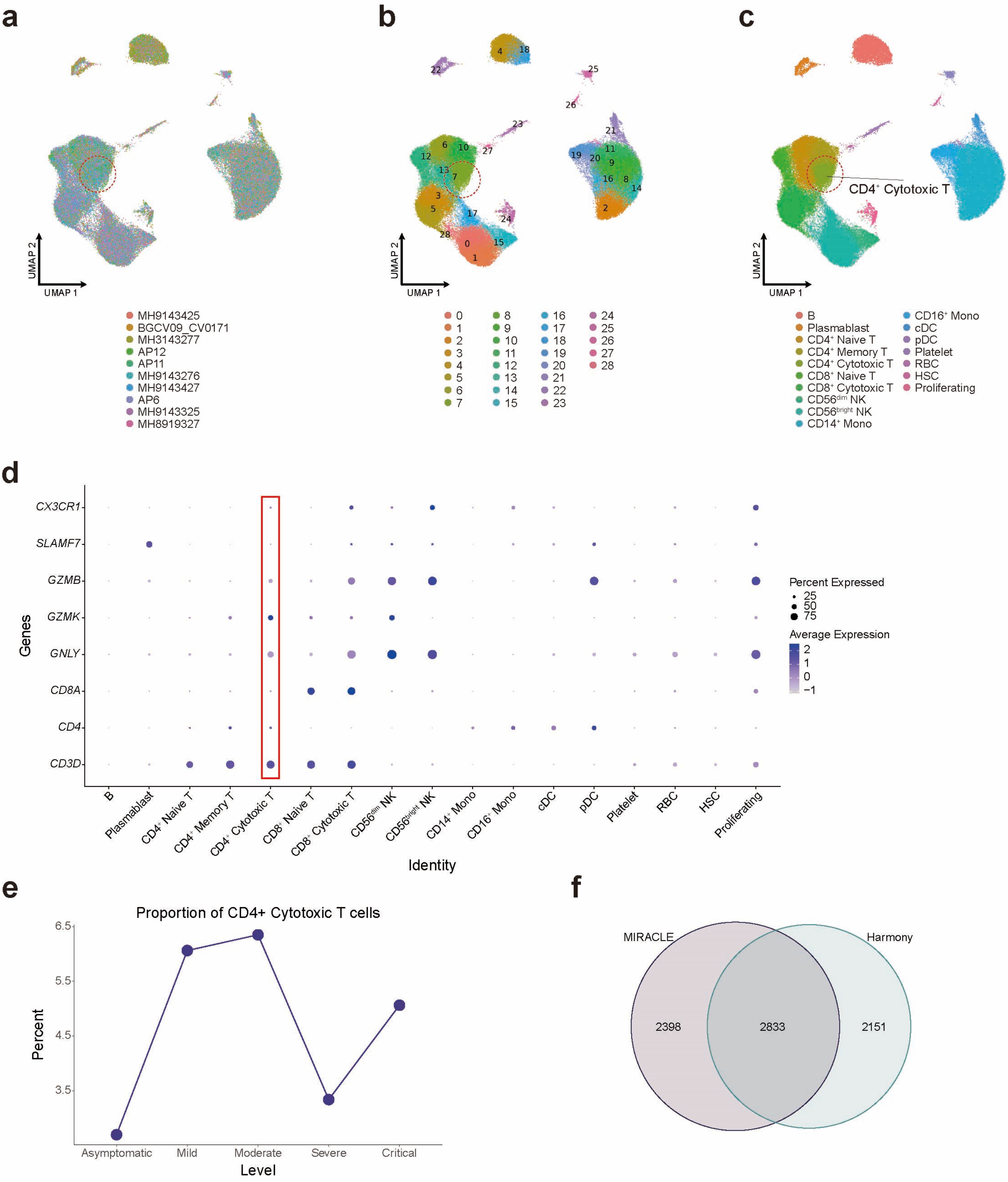

Supplementary Fig. 29: Validation of CD4⁺ CTLs using an independent pipeline on the original dataset. a–c, UMAP plots from a Harmony-based pipeline on the original data, identifying (a–c) the CD4⁺ CTL cluster (Cluster 7). **d**, Dot plot showing consistent CD4⁺ CTL marker expression in Cluster 7. **e**, Proportions of CD4⁺ CTLs across disease severities, recapitulating the trend found by MIRACLE. **f**, Venn diagram showing the overlap of CD4⁺ CTLs identified by MIRACLE and Harmony.

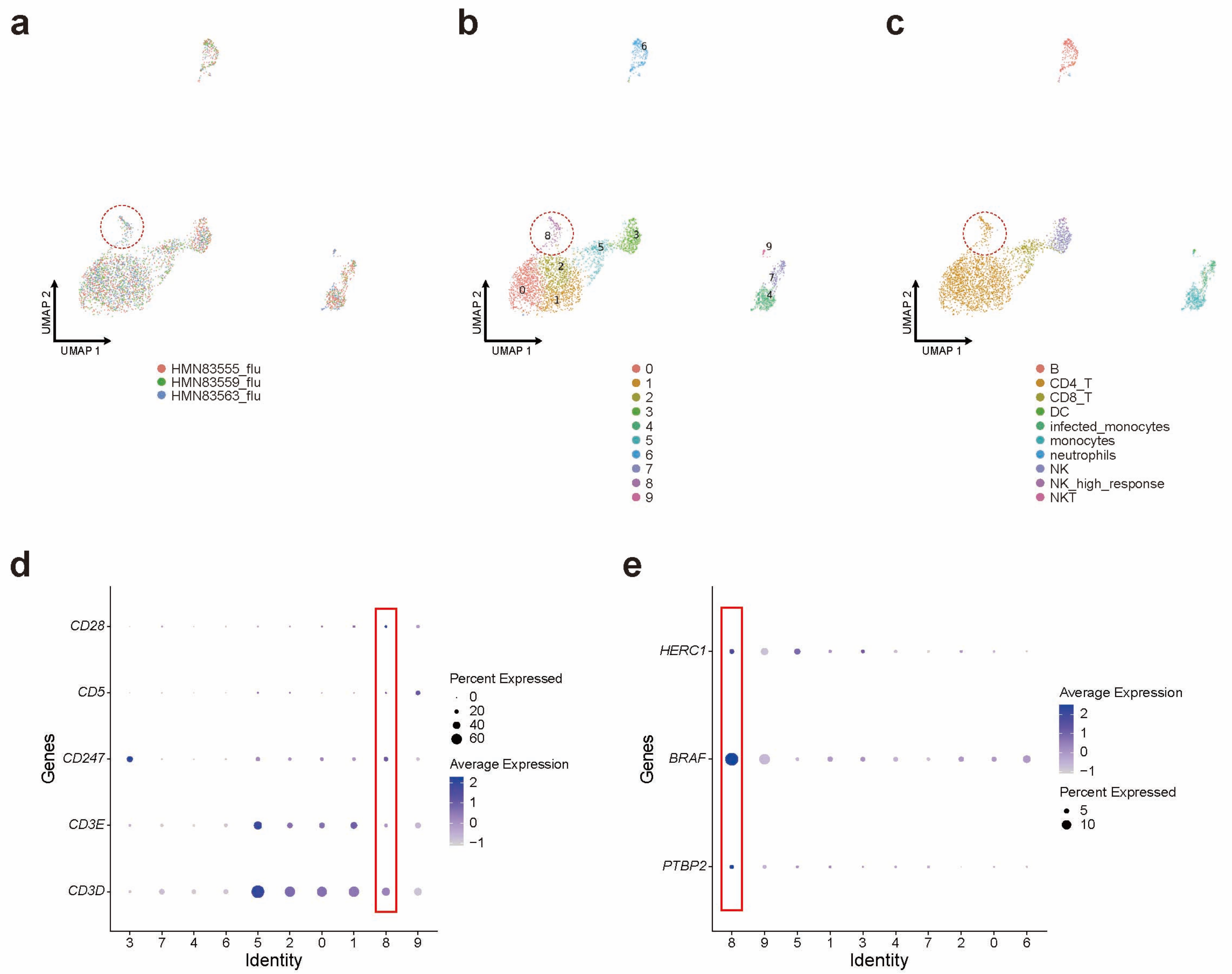

Supplementary Fig. 30: Attempted validation of HERC1⁺ T cells in an independent Flu A dataset. a–c, UMAP plots of an independent Flu A dataset, showing (a–c) the candidate HERC1⁺ T cell cluster (Cluster 8). **d**, Dot plot showing T cell markers in Cluster 8. **e**, Dot plot showing weak expression of HERC1⁺ T-associated genes (*HERC1*, *BRAF*, *PTBP2*) in Cluster 8.

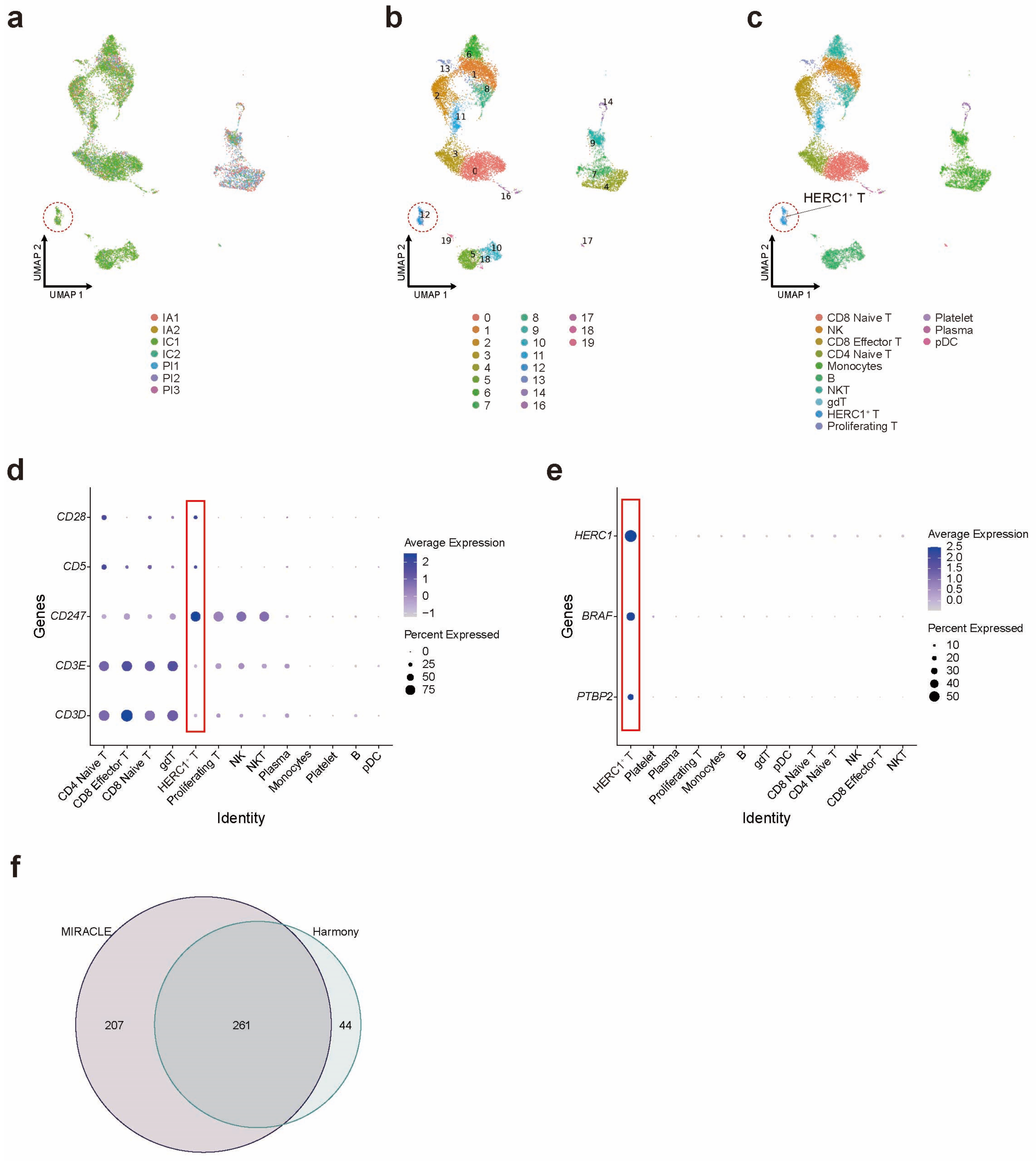

Supplementary Fig. 31: Validation of HERC1$^+$ T cells using an independent pipeline on the original dataset. a–c, UMAP plots from a Harmony-based pipeline on the original Flu A data, identifying (a–c) the HERC1$^+$ T cell cluster (Cluster 12). **d**, **e**, Dot plots confirming expression of (d) T cell markers and (e) key HERC1$^+$ T-associated genes (*HERC1*, *BRAF*, *PTBP2*) in Cluster 12. **f**, Venn diagram showing substantial overlap of HERC1$^+$ T cells identified by MIRACLE and Harmony.

Supplementary Table 1. scIB benchmarking on the DHCM dataset.

Method	Graph iLISI	Graph connectivity	kBET	Batch correction	NMI	ARI	Isolated label F1	Graph cLISI	Biological conservation	Overall score
**MIRACLE-offline**	0.3156	0.9718	0.4546	0.5807	0.9506	0.9677	0.8966	1.0000	0.9537	0.8045
**MIRACLE-BTS-20K**	0.2744	0.9559	0.4376	0.5560	0.9419	0.9628	0.8263	0.9998	0.9327	0.7820
**PCA**	0.1581	0.9703	0.2914	0.4733	0.9232	0.9109	0.8417	1.0000	0.9189	0.7407
**MIRACLE-SRS-20K**	0.2763	0.9591	0.4384	0.5579	0.9349	0.9604	0.0795	0.9997	0.7436	0.6693
**MIRACLE-transfer**	0.2330	0.9396	0.3860	0.5195	0.9295	0.9567	0.0414	0.9996	0.7318	0.6469
**Geneformer**	0.2348	0.9564	0.3690	0.5200	0.8938	0.8990	0.0786	0.9998	0.7178	0.6387
**MIRACLE-520K**	0.2723	0.9772	0.3160	0.5218	0.9516	0.9686	0.9191	1.0000	0.9598	0.7846
**MIRACLE-BTS-200K**	0.2918	0.9746	0.3673	0.5445	0.9537	0.9686	0.9132	1.0000	0.9589	0.7931
**MIRACLE-BTS-100K**	0.2852	0.9780	0.3754	0.5462	0.9529	0.9680	0.9098	1.0000	0.9577	0.7931
**MIRACLE-BTS-50K**	0.2669	0.9681	0.3732	0.5361	0.9449	0.9645	0.8931	1.0000	0.9506	0.7848
**MIRACLE-BTS-20K**	0.2744	0.9559	0.4376	0.5560	0.9419	0.9628	0.8263	0.9998	0.9327	0.7820
**MIRACLE-BTS-10K**	0.2812	0.9343	0.4915	0.5690	0.8952	0.9271	0.0518	0.9988	0.7182	0.6585
**MIRACLE-BTS-5K**	0.3000	0.8979	0.5958	0.5979	0.7849	0.8473	0.0430	0.9897	0.6662	0.6389
**MIRACLE-BTS-2K**	0.3007	0.8628	0.5257	0.5631	0.7518	0.8152	0.0159	0.9878	0.6427	0.6108

Supplementary Table 2. scIB benchmarking on the HLCA dataset.

Method	Graph iLISI	Graph connectivity	kBET	Batch correction	NMI	ARI	Isolated label F1	Graph cLISI	Biological conservation	Overall score
**MIRACLE-BTS-50K**	0.0725	0.9395	0.7765	0.5962	0.7294	0.6319	0.6725	0.9936	0.7569	0.6926
**MIRACLE-offline**	0.0744	0.9416	0.7963	0.6041	0.6958	0.5886	0.6769	0.9914	0.7382	0.6846
**MIRACLE-transfer**	0.0384	0.9239	0.7739	0.5788	0.6941	0.5138	0.6740	0.9948	0.7192	0.6630
**PCA**	0.0565	0.8712	0.8530	0.5936	0.6062	0.3607	0.6071	0.9901	0.6410	0.6220
**Geneformer**	0.0201	0.8549	0.7588	0.5446	0.6758	0.4226	0.1709	0.9950	0.5661	0.5575

Supplementary Table 3. Construction of the Cell-type-missing WNN dataset.

Data batch		Cell type							
		B	$CD4^+$ T	$CD8^+$ T	DC	Mono	NK	Other	Other T
Original data	P1_0	236	2198	1154	128	1890	494	184	94
	P2_0	455	1921	1140	93	1284	554	75	377
	P3_0	363	1422	525	72	1159	890	40	157
	P4_0	410	1524	913	113	1740	119	46	420
	P5_0	543	896	1157	216	2747	863	190	340
	P6_0	729	1062	356	95	2755	795	151	117
	P7_0	987	1258	1223	383	3748	997	84	174
	P8_0	394	2052	1646	123	3188	1075	100	330
Constructed data	P1_0	**0**	2198	1154	128	1890	494	184	94
	P2_0	455	**0**	1140	93	1284	554	75	377
	P3_0	363	1422	**0**	72	1159	890	40	157
	P4_0	410	1524	913	**0**	1740	119	46	420
	P5_0	543	896	1157	216	**0**	863	190	340
	P6_0	729	1062	356	95	2755	**0**	151	117
	P7_0	987	1258	1223	383	3748	997	**0**	174
	P8_0	394	2052	1646	123	3188	1075	100	**0**

*Cell numbers of each batch belonging different cell types are presented.

Supplementary Table 4. scIB benchmarking of online methods on the Cell-type-missing WNN dataset.

Method	Graph iLISI	Graph connectivity	kBET	Batch correction	NMI	ARI	Isolated label F1	Graph cLISI	Biological conservation	Overall score
**MIRACLE**	0.4485	0.9569	0.3138	0.5731	0.9079	0.9167	0.8958	1.0000	0.9301	0.7873
**Multigrate+scArches**	0.4248	0.9498	0.2776	0.5507	0.8248	0.8174	0.8118	0.9983	0.8631	0.7381
**Symphony**	0.4452	0.9525	0.2021	0.5333	0.8219	0.8108	0.8221	0.9996	0.8636	0.7315
**scVAEIT-transfer**	0.2887	0.9806	0.0493	0.4396	0.8676	0.8350	0.8957	1.0000	0.8996	0.7156
**RPCA+WNN-continual**	0.3109	0.9389	0.1105	0.4534	0.8393	0.8255	0.8349	1.0000	0.8749	0.7063
**CCA+WNN-continual**	0.5291	0.9483	0.1725	0.5500	0.7566	0.7074	0.7728	1.0000	0.8092	0.7055
**MIRACLE-transfer**	0.1543	0.9568	0.0182	0.3764	0.9039	0.9026	0.8803	1.0000	0.9217	0.7036
**SCALEX-projection**	0.3736	0.9513	0.0673	0.4641	0.8100	0.7839	0.8521	0.9975	0.8609	0.7022
**online iNMF**	0.4983	0.9549	0.3790	0.6107	0.7017	0.6276	0.7066	0.9933	0.7573	0.6987
**sciPENN-transfer**	0.3736	0.9155	0.0808	0.4566	0.7189	0.7621	0.7319	0.9975	0.8026	0.6642
**Concerto-transfer**	0.2980	0.9534	0.0694	0.4403	0.7246	0.6681	0.7755	0.9873	0.7889	0.6494
**totalVI+scArches**	0.0000	0.6722	0.0022	0.2248	0.4821	0.1710	0.3402	0.9918	0.4963	0.3877
**trVAE+scArches**	0.2949	0.7845	0.0576	0.3790	0.0939	0.0276	0.2643	0.6744	0.2651	0.3106

Supplementary Table 5. scIB benchmarking of offline methods on the Cell-type-missing WNN dataset.

Method	Graph iLISI	Graph connectivity	kBET	Batch correction	NMI	ARI	Isolated label F1	Graph cLISI	Biological conservation	Overall score
**MIRACLE-offline**	0.4622	0.9570	0.3536	0.5909	0.8966	0.8856	0.9017	1.0000	0.9210	0.7890
**Multigrate**	0.4543	0.9518	0.3045	0.5702	0.8841	0.8803	0.8855	0.9995	0.9124	0.7755
**trVAE**	0.5226	0.9489	0.5681	0.6799	0.7666	0.7442	0.8141	0.9413	0.8166	0.7619
**totalVI**	0.3850	0.9570	0.1131	0.4850	0.9089	0.9190	0.9201	1.0000	0.9370	0.7562
**online iNMF-offline**	0.5073	0.9508	0.5131	0.6571	0.7538	0.7230	0.7759	0.9978	0.8126	0.7504
**scVAEIT**	0.3907	0.9551	0.1523	0.4994	0.8757	0.8272	0.8498	1.0000	0.8882	0.7327
**CCA+WNN**	0.5511	0.9515	0.2264	0.5763	0.7563	0.7093	0.7767	1.0000	0.8106	0.7169
**RPCA+WNN**	0.5136	0.9408	0.1688	0.5411	0.7812	0.7340	0.7920	1.0000	0.8268	0.7125
**Symphony-offline**	0.4377	0.9496	0.1796	0.5223	0.7771	0.7617	0.8165	0.9982	0.8384	0.7119
**SCALEX**	0.2749	0.9438	0.0309	0.4165	0.8384	0.8150	0.8874	0.9996	0.8851	0.6977
**Concerto**	0.3113	0.9753	0.1019	0.4628	0.7725	0.6772	0.7894	0.9945	0.8084	0.6702
**sciPENN**	0.1732	0.9755	0.0129	0.3872	0.8091	0.7182	0.8316	1.0000	0.8397	0.6587

Supplementary Table 6. Construction of the Dynamic WNN dataset.

Data batch		Cell type							
		**B**	**CD4$^+$ T**	**CD8$^+$ T**	**DC**	**Mono**	**NK**	**Other**	**Other T**
**Original data**	**P1_0**	236	2198	1154	128	1890	494	184	94
	**P2_0**	455	1921	1140	93	1284	554	75	377
	**P3_0**	363	1422	525	72	1159	890	40	157
	**P4_0**	410	1524	913	113	1740	119	46	420
	**P5_0**	543	896	1157	216	2747	863	190	340
	**P6_0**	729	1062	356	95	2755	795	151	117
	**P7_0**	987	1258	1223	383	3748	997	84	174
	**P8_0**	394	2052	1646	123	3188	1075	100	330
**Constructed data**	**P1_0**	**0**	2198	1154	**0**	1890	**0**	**0**	**0**
	**P2_0**	455	1921	**0**	**0**	1284	554	**0**	**0**
	**P3_0**	363	**0**	525	72	1159	**0**	**0**	**0**
	**P4_0**	**0**	1524	**0**	113	1740	**0**	**0**	420
	**P5_0**	543	**0**	1157	**0**	**0**	863	**0**	340
	**P6_0**	**0**	1062	**0**	**0**	2755	795	151	**0**
	**P7_0**	987	1258	1223	383	**0**	**0**	84	**0**
	**P8_0**	**0**	2052	1646	**0**	3188	1075	**0**	330

*Cell numbers of each batch belonging different cell types are presented.

*This benchmark dataset was designed to more stringently evaluate the model's ability to handle complex and dynamic data streams. It was specifically constructed to incorporate several demanding challenges, including: (1) the continual introduction of novel cell types across batches to test adaptability; (2) the alternating presence and absence of key cell types (e.g., CD8$^+$ T cells) to explicitly test for catastrophic forgetting; and (3) complex compositional shifts at each integration step, involving the simultaneous addition and removal of multiple cell types. This design creates a demanding benchmark scenario to validate the robustness and superiority of online integration methods in handling dynamic, real-world data streams.

Supplementary Table 7. scIB benchmarking of online methods on the Dynamic WNN dataset.

Method	Graph iLISI	Graph connectivity	kBET	Batch correction	NMI	ARI	Isolated label F1	Graph cLISI	Biological conservation	Overall score
**MIRACLE**	0.9932	0.4259	0.3469	0.5887	0.8547	0.8446	0.8269	0.9996	0.8815	0.7643
**MIRACLE-transfer**	0.9932	0.0274	0.1673	0.3959	0.9120	0.9237	0.8365	1.0000	0.9181	0.7092
**scVAEIT-transfer**	0.9700	0.0689	0.2297	0.4229	0.8345	0.8134	0.7054	1.0000	0.8383	0.6721
**totalVI+scArches**	0.9836	0.0380	0.2889	0.4369	0.7411	0.7123	0.8188	0.9937	0.8165	0.6646
**online iNMF**	0.9847	0.2702	0.3993	0.5514	0.6945	0.6747	0.5824	0.9852	0.7342	0.6611
**sciPENN-transfer**	0.8089	0.0912	0.3158	0.0912	0.5512	0.5662	0.7127	0.9787	0.7022	0.4578
**Symphony**	0.9901	0.0313	0.2711	0.4308	0.3844	0.3775	0.7027	0.9267	0.5978	0.5310
**SCALEX-projection**	0.9747	0.0801	0.3529	0.4692	0.3751	0.4172	0.6351	0.8407	0.5670	0.5279
**Multigrate+scArches**	0.9905	0.1166	0.1998	0.4356	0.6597	0.5645	0.1318	0.9503	0.5766	0.5202
**Seurat-CCA+WNN-continual**	0.9400	0.0268	0.0004	0.3224	0.5256	0.2895	0.6629	0.9958	0.6185	0.5000
**Seurat-RPCA+WNN-continual**	0.6215	0.0000	0.0000	0.2072	0.5536	0.2157	0.8517	1.0000	0.6553	0.4760
**trVAE+scArches**	0.9474	0.3911	0.4261	0.5882	0.2717	0.2469	0.0673	0.7872	0.3433	0.4412
**Concerto-transfer**	0.9615	0.0919	0.3197	0.4577	0.2746	0.2708	0.0317	0.7904	0.3419	0.3882

Supplementary Table 8. scIB benchmarking of offline methods on the Dynamic WNN dataset.

Method	Graph iLISI	Graph connectivity	kBET	Batch correction	NMI	ARI	Isolated label F1	Graph cLISI	Biological conservation	Overall score
**MIRACLE-offline**	0.9837	0.4997	0.3424	0.6086	0.8794	0.8762	0.8210	1.0000	0.8942	0.7799
**scVAEIT**	0.9837	0.2722	0.3317	0.5292	0.9027	0.9190	0.8475	1.0000	0.9173	0.7620
**trVAE**	0.9923	0.5046	0.4624	0.6531	0.5716	0.5634	0.8313	0.7878	0.6885	0.6744
**Concerto**	0.9922	0.0911	0.2338	0.4390	0.7365	0.6886	0.5520	0.9723	0.7373	0.6180
**Multigrate**	0.9914	0.0121	0.1415	0.3816	0.6911	0.5103	0.8571	0.9919	0.7626	0.6102
**Symphony-offline**	0.9945	0.0296	0.2531	0.4257	0.6719	0.6935	0.5771	0.9714	0.7285	0.6074
**sciPENN**	0.8570	0.0032	0.0569	0.0032	0.7271	0.7213	0.7202	1.0000	0.7921	0.4766
**totalVI**	0.9956	0.0018	0.2045	0.4006	0.7014	0.5311	0.6416	0.9835	0.7144	0.5889
**SCALEX**	0.9957	0.0180	0.0022	0.3386	0.5531	0.2113	0.6400	0.9879	0.5981	0.4943
**Seurat-RPCA+WNN**	0.8197	0.0008	0.0000	0.2735	0.5338	0.1914	0.8345	1.0000	0.6399	0.4934
**Seurat-CCA+WNN**	0.9539	0.0645	0.0450	0.3545	0.5428	0.4378	0.2638	0.9913	0.5589	0.4771
**online iNMF-offline**	0.8589	0.0224	0.3218	0.4010	0.2552	0.1324	0.3566	0.8401	0.3961	0.3981

Supplementary Table 9. Construction of the Cell-type-missing mosaic DOTEA dataset.

**Data batch**		**Cell type**								**Modality**		
		**B**	**CD4⁺ T**	**CD8⁺ T**	**DC**	**Mono**	**NK**	**other**	**other T**	**ATAC**	**RNA**	**ADT**
**Original data**	**DIG Ctrl**	463	6913	1713	57	112	298	23	611	√	√	√
	**DIG Stim**	332	6377	1608	62	64	431	33	620	√	√	√
	**LLL Ctrl**	296	4983	1435	43	53	166	21	364	√	√	√
	**LLL Stim**	159	3974	1080	45	11	213	27	388	√	√	√
	**W3**	1245	2859	1066	78	216	309	652	162	√	√	√
	**W4**	1390	2984	1088	39	206	312	716	162	√	√	√
	**W5**	1280	2747	1283	64	275	343	711	207	√	√	√
	**W6**	1326	3128	1119	13	277	328	742	204	√	√	√
**Constructed data**	**DIG Ctrl**	463	6913	1713	57	112	298	23	**0**	√		√
	**DIG Stim**	332	6377	1608	62	64	**0**	33	620	√	√	
	**LLL Ctrl**	296	4983	1435	**0**	53	166	21	364		√	√
	**LLL Stim**	159	3974	**0**	45	11	213	27	388	√	√	√
	**W3**	**0**	2859	1066	78	216	309	652	162	√		√
	**W4**	1390	2984	1088	39	206	312	**0**	162	√	√	
	**W5**	1280	**0**	1283	64	275	343	711	207		√	√
	**W6**	1326	3128	1119	13	**0**	328	742	204	√	√	√

*Cell numbers across different cell types and modalities of each batch are presented.

*To address the large difference in the number of various cell types in the DOTEA dataset, we adopted the strategy that minimizing the variance of the number of deleted cells.

Supplementary Table 10. scMIB benchmarking of online and offline methods on the DOTEA dataset.

Method	Graph iLISI	Graph connectivity	kBET	Batch correction	Modality ASW	FOSCTTM	Label Transfer F1	ATAC AUROC	RNA Pearson's R	ADT Pearson's R	Modality alignment	NMI	ARI	Isolated label F1	Graph cLISI	Biological conservation	Overall score
**MIRACLE**	0.4819	0.9389	0.3649	0.5952	0.9040	0.9030	0.8413	0.8171	0.7209	0.8539	0.8400	0.6433	0.7028	0.7591	0.9834	0.7721	0.7394
**MIRACLE -offline**	0.4929	0.9390	0.3962	0.6093	0.9291	0.9371	0.8722	0.8178	0.8201	0.8928	0.8782	0.6031	0.6668	0.6675	0.9788	0.7291	0.7379
**scVAEIT**	0.1345	0.9114	0.0163	0.3541	0.8052	0.9094	0.8959	0.8195	0.8523	0.8661	0.8581	0.6581	0.7202	0.7135	0.9900	0.7705	0.6718
**MIRACLE -transfer**	0.1258	0.9381	0.0464	0.3701	0.8333	0.8347	0.8126	0.8070	0.5865	0.5838	0.7430	0.5972	0.4305	0.6937	0.9886	0.6775	0.6049
**Multigrate**	0.2226	0.9253	0.0301	0.3927	0.9920	0.9873	0.9264	0.6303	0.6687	0.7860	0.8318	0.5290	0.3830	0.6458	0.9849	0.6357	0.6216
**Multigrate+ scArches**	0.1243	0.9135	0.0050	0.3476	0.9916	0.9706	0.9165	0.5263	0.7684	0.8349	0.8347	0.3986	0.2332	0.5810	0.9665	0.5448	0.5726
**scVAEIT- transfer**	0.1237	0.8938	0.0486	0.3554	0.5021	0.6727	0.6837	0.8032	0.7355	0.4310	0.6380	0.4058	0.1862	0.5638	0.9619	0.5294	0.5098

Supplementary Table 11. Datasets used in continual atlas construction and label transfer.

Dataset	Tissue	Disease condition	Batch number	Modality			Cell number	Within-tissue		Cross-tissue		Cross-disease	
				ATAC	RNA	ADT		Reference (atlas-no-nac)	New (nac)	Reference (PBMC atlas)	New (non-PBMC)	Reference (PBMC atlas)	New (infected-PBMC)
DOGMA	Blood	Healthy	4	√	√	√	32,975	√		√		√	
TEA			5	√	√	√	34,856	√		√		√	
TEA Multiome			2	√	√		13,380	√		√		√	
10X Multiome			4	√	√		33,132	√		√		√	
WNN			8		√	√	52,964	√		√		√	
DOGMA+CITE			1		√	√	11,967	√		√		√	
			3	√	√	√	37,727	√		√		√	
ISSAAC			1	√	√		7,053	√		√		√	
NEAT			2	√	√		10,524		√	√		√	
ASAP			2	√		√	9,496		√	√		√	
ASAP-CITE			2		√	√	8,715		√	√		√	
Tonsil	Lymphoid		1		√		5,503				√		
BMMC	Hematopoietic		1	√		√	10,671				√		
Spleen	Lymphoid		1		√		4,287				√		
COVID-19	Blood	COVID-19	10		√	√	97,938						√
Flu A		Flu A	7		√		15,693						√
TB		TB	20		√	√	55,628						√
Total cell number				189,814	464,135	181,160	442,509	224,054	28,735	252,789	20,461	252,789	169,259

Supplementary Table 12. Public datasets used in this study.

Dataset	Batch	Protocol	Accession ID/URL	Filtered cell number	ATAC	RNA	ADT
DHCM	P1–P42	10x v3 Sequencing	SCP1303	523,369		√	
HLCA	Dataset 1–Dataset 48	10x 3' v1<br>10x 3' v2<br>10x 3' v3<br>10x 5' transcription profiling<br>10x 5' v1<br>10x 5' v2<br>Drop-seq<br>Seq-Well	https://cellxgene.cziscience.com/collections/6f6d381a-7701-4781-935c-db10d30de293	2,282,447		√	
DOGMA	LLL Ctrl	DOGMA-seq	GSE166188 (GSM5065524, GSM5065525, GSM5065526)	7,361	√	√	√
	LLL Stim	DOGMA-seq	GSE166188 (GSM5065527, GSM5065528, GSM5065529)	5,897	√	√	√
	DIG Ctrl	DOGMA-seq	GSE166188 (GSM5065530, GSM5065531, GSM5065532)	10,190	√	√	√
	DIG Stim	DOGMA-seq	GSE166188 (GSM5065533, GSM5065534, GSM5065535)	9,527	√	√	√
TEA	W1	TEA-seq	GSE158013 (GSM4949911)	7,325	√	√	√
	W3	TEA-seq	GSE158013 (GSM5123951)	6,587	√	√	√
	W4	TEA-seq	GSE158013 (GSM5123952)	6,897	√	√	√
	W5	TEA-seq	GSE158013 (GSM5123953)	6,910	√	√	√
	W6	TEA-seq	GSE158013 (GSM5123954)	7,137	√	√	√
TEA Multiome	W1	10x Chromium Single Cell Multiome ATAC + Gene Expression	GSE158013 (GSM5123949)	6,096	√	√	
	W2	10x Chromium Single Cell Multiome ATAC + Gene Expression	GSE158013 (GSM5123950)	7,284	√	√	
10X Multiome	ChrX	10x Chromium Single Cell Multiome ATAC + Gene Expression	https://www.10xgenomics.com/resources/datasets/10-k-human-pbm-cs-multiome-v-1-0-chromium-x-1-standard-2-0-0	9,868	√	√	
	ChrC	10x Chromium Single Cell Multiome ATAC + Gene Expression	https://www.10xgenomics.com/resources/datasets/10-k-human-pbm-cs-multiome-v-1-0-chromium-controller-1-standard-2-0-0	9,582	√	√	
	ARC2 10K	10x Chromium Single Cell Multiome ATAC + Gene Expression	https://www.10xgenomics.com/resources/datasets/pbmc-from-a-healthy-donor-no-cell-sorting-10-k-1-standard-2-0-0	11,116	√	√	
	ARC2 3K	10x Chromium Single Cell Multiome ATAC + Gene Expression	https://www.10xgenomics.com/resources/datasets/pbmc-from-a-healthy-donor-no-cell-sorting-3-k-1-standard-2-0-0	2,566	√	√	
	P1_0	CITE-seq	https://atlas.fredhutch.org/nygc/multimodal-pbmc/	6,378		√	√

WNN	P2_0	CITE-seq	https://atlas.fredhutch.org/nygc/multimodal-pbmc/	5,899		√	√
	P3_0	CITE-seq	https://atlas.fredhutch.org/nygc/multimodal-pbmc/	4,628		√	√
	P4_0	CITE-seq	https://atlas.fredhutch.org/nygc/multimodal-pbmc/	5,285		√	√
	P5_0	CITE-seq	https://atlas.fredhutch.org/nygc/multimodal-pbmc/	6,952		√	√
	P6_0	CITE-seq	https://atlas.fredhutch.org/nygc/multimodal-pbmc/	6,060		√	√
	P7_0	CITE-seq	https://atlas.fredhutch.org/nygc/multimodal-pbmc/	8,854		√	√
	P8_0	CITE-seq	https://atlas.fredhutch.org/nygc/multimodal-pbmc/	8,908		√	√
DOGMA+ CITE	CITE	CITE-seq	GSE200417 (GSM6032898, GSM6032900)	11,967		√	√
	LLL	DOGMA-seq	GSE200417 (GSM6032890, GSM6032891, GSM6032893)	9,794	√	√	√
	DIG	DOGMA-seq	GSE200417 (GSM6032886, GSM6032887, GSM6032889)	14,838	√	√	√
	DOGMA	DOGMA-seq	GSE200417 (GSM6032894, GSM6032895, GSM6032897)	13,095	√	√	√
ISSAAC	ISSAAC	ISSAAC-seq	E-MTAB-11264	7,053	√	√	
NEAT	lane1	NEAT-seq	GSE178707 (GSM5396332, GSM5396333)	5,568	√	√	
	lane2	NEAT-seq	GSE178707 (GSM5396336, GSM5396337)	4,956	√	√	
ASAP	Ctrl	ASAP-seq	GSE156473 (GSM4732109, GSM4732110)	4,255	√		√
	Stim	ASAP-seq	GSE156473 (GSM4732111, GSM4732112)	5,241	√		√
ASAP-CITE	Ctrl	CITE-seq	GSE156473 (GSM4732113, GSM4732114)	5,086		√	√
	Stim	CITE-seq	GSE156473 (GSM4732115, GSM4732116)	3,629		√	√
Tonsil	Tonsil	10x Genomics Chromium Single Cell 3' kit	GSE165860 (GSM5051493)	5,503		√	
BMMC	BMMC	ASAP-seq	GSE156477 (GSM4732140, GSM4732141)	10,671	√		√
Spleen	Spleen	10x Genomics Chromium Single Cell V(D)J kit	GSE159929 (GSM4850589)	4,287		√	
COVID-19	MH9143425	CITE-seq	E-MTAB-10026	7,011		√	√
	BGCV09_CV0171	CITE-seq	E-MTAB-10026	3,274		√	√
	MH9143277	CITE-seq	E-MTAB-10026	11,710		√	√
	AP12	CITE-seq	E-MTAB-10026	8,574		√	√
	AP11	CITE-seq	E-MTAB-10026	10,921		√	√
	MH9143276	CITE-seq	E-MTAB-10026	10,207		√	√
	MH9143427	CITE-seq	E-MTAB-10026	14,317		√	√
	AP6	CITE-seq	E-MTAB-10026	14,086		√	√

	MH9143325	CITE-seq	E-MTAB-10026	9,019		√	√
	MH8919327	CITE-seq	E-MTAB-10026	8,819		√	√
Flu A	IA1	GEXSCOPE Single-Cell RNA Library Kit	GSE243629 (GSM7792039)	2,239		√	
	IA2	GEXSCOPE Single-Cell RNA Library Kit	GSE243629 (GSM7792040)	361		√	
	IC1	GEXSCOPE Single-Cell RNA Library Kit	GSE243629 (GSM7792037)	9,570		√	
	IC2	GEXSCOPE Single-Cell RNA Library Kit	GSE243629 (GSM7792038)	535		√	
	PI1	GEXSCOPE Single-Cell RNA Library Kit	GSE243629 (GSM7792041)	1,527		√	
	PI2	GEXSCOPE Single-Cell RNA Library Kit	GSE243629 (GSM7792042)	1,105		√	
	PI3	GEXSCOPE Single-Cell RNA Library Kit	GSE243629 (GSM7792043)	356		√	
TB	TB6361266_39	CITE-seq	GSE158769	3,350		√	√
	TB8165290_27	CITE-seq	GSE158769	3,001		√	√
	TB7697020_35	CITE-seq	GSE158769	2,809		√	√
	TB1792755_39	CITE-seq	GSE158769	2,863		√	√
	TB1611911_36	CITE-seq	GSE158769	2,788		√	√
	TB3329322_4	CITE-seq	GSE158769	2,571		√	√
	TB7392866_14	CITE-seq	GSE158769	2,717		√	√
	TB9100553_14	CITE-seq	GSE158769	2,609		√	√
	TB2374859_27	CITE-seq	GSE158769	2,588		√	√
	TB7300167_2	CITE-seq	GSE158769	2,520		√	√
	TB2531658_39	CITE-seq	GSE158769	2,942		√	√
	TB0533278_40	CITE-seq	GSE158769	2,932		√	√
	TB6692076_39	CITE-seq	GSE158769	3,119		√	√
	TB8866882_36	CITE-seq	GSE158769	2,716		√	√
	TB2247275_40	CITE-seq	GSE158769	2,736		√	√
	TB8896313_18	CITE-seq	GSE158769	2,775		√	√

	TB6209826_44	CITE-seq	GSE158769	2,680		√	√
	TB5556538_37	CITE-seq	GSE158769	2,561		√	√
	TB4598647_35	CITE-seq	GSE158769	2,913		√	√
	TB6270533_36	CITE-seq	GSE158769	2,438		√	√

Supplementary Table 13. Overview of compared methods for single-cell multimodal integration.

Integration type	Method	Scenario				Task		
		offline	online			Unimodal task (RNA, horizontal)	Bimodal task (RNA+ADT, rectangular)	Trimodal task (ATAC+RNA+ADT, mosaic)
			generalization	fine-tuning	continual			
Mosaic	MIRACLE-offline	√				√	√	√
	MIRACLE				√	√	√	√
	MIRACLE-transfer			√		√	√	√
	Multigrate	√					√	√
	Multigrate+scArches		√				√	√
	scVAEIT	√					√	√
	scVAEIT-transfer*			√			√	√
	totalVI	√					√	
	totalVI+scArches		√				√	
Rectangular	SCALEX	√					√	
	SCALEX-projection		√				√	
	trVAE	√					√	
	trVAE+scArches		√				√	
	Concerto	√					√	
	Concerto-transfer			√			√	
	Symphony-offline	√					√	
	Symphony		√				√	
	online iNMF-offline	√					√	
	online iNMF			√			√	
	CCA+WNN	√					√	
	CCA+WNN-continual				√		√	
	RPCA+WNN	√					√	
	RPCA+WNN-continual				√		√	
	sciPENN	√					√	
	sciPENN-transfer*			√			√	
Horizontal	Geneformer		√			√		
	PCA	√				√		

The asterisk (*) indicates that this method does not support online scenario, thus we adopted fine-tuning-based transfer learning strategies.
