# Supplementary material for: Continual integration of single-cell multimodal data with MIRACLE
Source: Nat Comput Sci. 2026 Jul 31;6(9):988–1004. doi: 10.1038/s43588-026-01030-9 (PMC13593572; doi:10.1038/s43588-026-01030-9)
Supplement: Supplementary file 3 [file 43588_2026_1030_MOESM3_ESM.pdf]

nature portfolio

Peer Review File

Continual integration of single-cell multimodal data with MIRACLE

Corresponding Author: Professor Zhen He

This file contains all editorial decision letters in order by version, followed by all author rebuttals in order by version.

Version 0:

Decision Letter:

** Please ensure you delete the link to your author homepage in this e-mail if you wish to forward it to your co-authors. **

Dear Professor Ying,

Your manuscript "Continual integration of single-cell multimodal data with MIRACLE" has now been seen by 2 referees, whose comments are appended below. You will see that while they find your work of interest, they have raised points that need to be addressed before we can make a decision on publication.

The referees' reports seem to be quite clear. Naturally, we will need you to address *all* of the points raised.

Please use the following link to submit your revised manuscript and a point-by-point response to the referees' comments (which should be in a separate document to any cover letter):

Link Redacted

** This url links to your confidential homepage and associated information about manuscripts you may have submitted or be reviewing for us. If you wish to forward this e-mail to co-authors, please delete this link to your homepage first. **

To aid in the review process, we would appreciate it if you could also provide a copy of your manuscript files that indicates your revisions by making use of Track Changes or similar mark-up tools. Please also ensure that all correspondence is marked with your Nature Computational Science reference number in the subject line.

In addition, please make sure to upload a Word Document or LaTeX version of your text, to assist us in the editorial stage.

If you have any issues when updating your Code Ocean capsule during the revision process, please email the Code Ocean support team Cc'ing me.

To improve transparency in authorship, we request that all authors identified as 'corresponding author' on published papers create and link their Open Researcher and Contributor Identifier (ORCID) with their account on the Manuscript Tracking System (MTS), prior to acceptance. ORCID helps the scientific community achieve unambiguous attribution of all scholarly contributions. You can create and link your ORCID from the home page of the MTS by clicking on 'Modify my Springer Nature account'. For more information please visit please visit www.springernature.com/orcid.

We hope to receive your revised paper within three weeks. If you cannot send it within this time, please let us know.

We look forward to hearing from you soon.

Best regards,

Michelle Badri, PhD
Associate Editor, Research Cross-Journal Editorial Team for Nature Computational Science

Reviewers comments:

Reviewer #2 (Remarks to the Author):

This manuscript introduces MIRACLE, a continual learning framework for online integration of single-cell multimodal mosaic data. It addresses a critical challenge by enabling efficient atlas updates without catastrophic forgetting through dynamic architecture adaptation and data rehearsal. The study is well-structured, and benchmarking demonstrates MIRACLE's competitive performance against existing methods. The respiratory infection case study provides biologically interesting findings with translational potential. Below are suggestions to enhance the manuscript's clarity and impact.

Major Comments

1. Terms like "online integration," "continual integration," and "dynamic integration" require clear definitions early in the Introduction to avoid ambiguity. Consistent terminology should be maintained throughout.
2. While MIRACLE's efficiency is highlighted, its validation on the 523K-cell DHCM dataset—relatively modest for current large-scale studies—limits this claim. Benchmarking runtime/GPU usage on substantially larger datasets, with direct comparisons to baselines, would strengthen this argument.
3. Since MIRACLE builds on MIDAS, it is required to briefly describe MIDAS's core methodology and explicitly detail how MIRACLE extends or improves upon it.
4. Figure 2's evaluation uses a single-nucleus RNA-seq dataset with apparently mild batch effects (per PCA). It is necessary to assess the integration performance on datasets with pronounced technical artifacts for a more rigorous test.
5. The attribution of transfer learning's poor performance to catastrophic forgetting lacks direct evidence. Please provide explicit analysis (e.g., forgetting metrics or ablation studies) to establish this causal link.
6. How does MIRACLE handle sparse ATAC peaks, noisy ADT signals, or missing modalities? These are very common issues in practice.
7. The cross-tissue integration results are encouraging, but the biological implications and potential issues should be discussed in more depth. For instance, is there a risk of mixing tissue-resident and circulating cell states (e.g., T cells from PBMCs vs. spleen)? It would be helpful to address how MIRACLE mitigates or interprets such ambiguity.
8. Continual learning methods can be sensitive to the order in which data are introduced. It is very important to test MIRACLE with varied input sequences (e.g., TB before/after Flu A) and to report performance stability or mitigation strategies.
9. The identification of HERC1+ T cells in Flu A and CD4+ CTLs in COVID-19 is interesting, but external validations are required to support these findings. Are these populations reproducible in independent datasets or preprocessing pipelines?
10. The manuscript highlights the importance of data sharing and collaboration. How MIRACLE's pipeline addresses privacy compliance?
11. It may be worth briefly discussing MIRACLE's extensibility to spatial omics data, where missing modalities and spatial artifacts pose distinct challenges. This would broaden MIRACLE's relevance to a rapidly evolving field.
12. In Figure 3a, add a brief caption note explaining that the blank lower triangle intentionally reflects stepwise integration, avoiding misinterpretation of incompleteness.

Reviewer #3 (Remarks to the Author):

This manuscript introduces an online learning framework, MIRACLE, for multimodal single-cell data integration. The proposed approach aims to incrementally incorporate new data while retaining previously learned information, which could enhance cell label transfer accuracy and facilitate novel cell type discovery. These capabilities have the potential to significantly improve understanding of complex biological systems and disease mechanisms. While the methodology appears promising, I have several concerns and questions that I would like the authors to address before I can recommend the manuscript for publication:
1. The authors state that "Systematic evaluation demonstrates that MIRACLE efficiently integrates multimodal data across batches, modalities, and cell types with minimal memory usage, achieving accuracy comparable to offline methods while significantly reducing computational costs." However, the manuscript does not provide sufficient evidence to support the claim of "minimal memory usage". Section 2.2, titled "MIRACLE enables continual integration using limited memory," does not clearly define what aspect of memory is being measured—is this during training, inference, or across data accumulation? Additionally, it would be helpful to provide a comparative analysis of memory usage across other methods. Please clarify this aspect and explain why MIRACLE is expected to be memory-efficient.
2. Could the authors provide the loss functions, particularly the classifier loss? Furthermore, the justification for the selected hyperparameters is unclear. Is there a possibility that performance could be improved further through hyperparameter tuning? If these details are already provided, I apologize for overlooking them.
3. Figure 2a is difficult to interpret. Could the authors clarify what is represented in the third row and the second column, for example? Additionally, the cell type combinations described in Supplementary Table 2 appear insufficient to support the conclusion that MIRACLE is superior in continually integrating distinct cell types. For instance, in the case where t=2, only one new cell type is introduced compared to t=1. In addition, when t=3, there is no novel cell types.
4. Does the offline model utilize the full dataset or only a sampled subset? How does the offline model perform using full and

sampled data, respectively?
5. The manuscript claims that MIRACLE outperforms MIRACLE-offline in constructing a cross-tissue multimodal atlas. Could the authors elaborate on the reasons behind this improvement? What aspects of the online learning strategy contribute to better performance in this context?
6. The authors state that "Our results demonstrate that MIRACLE matches the integration quality of its offline counterpart, the performance upper limit." This is a strong claim—is there a theoretical proof ? As I understand, offline model could be tuned further to generate a better result for a certain dataset.

Reviewer #3 (Remarks on code availability):

A tutorial is preferred, but not the code for the result.

Version 1:

Decision Letter:

Our ref: NATCOMPUTSCI-25-1024A

9th April 2026

Dear Dr. He,

Thank you for submitting your revised manuscript "Continual integration of single-cell multimodal data with MIRACLE" (NATCOMPUTSCI-25-1024A). In our last email communication I mentioned difficulties with the review process and I am happy to update that this has been resolved. We have discussed your responses to all referees with one of the original referees and the remaining comments are below. The reviewers find that the paper has improved in revision, and therefore we'll be happy in principle to publish it in Nature Computational Science, pending minor revisions to satisfy the referees' final requests and to comply with our editorial and formatting guidelines.

We are now performing detailed checks on your paper and will send you a checklist detailing our editorial and formatting requirements in the next few weeks. Please do not upload the final materials and make any revisions until you receive this additional information from us.

TRANSPARENT PEER REVIEW
Nature Computational Science offers a transparent peer review option for original research manuscripts. We encourage increased transparency in peer review by publishing the reviewer comments, author rebuttal letters and editorial decision letters if the authors agree. Such peer review material is made available as a supplementary peer review file. **Please remember to choose, using the manuscript system, whether or not you want to participate in transparent peer review.**
Please note: we allow redactions to authors' rebuttal and reviewer comments in the interest of confidentiality. If you are concerned about the release of confidential data, please let us know specifically what information you would like to have removed. Please note that we cannot incorporate redactions for any other reasons. Reviewer names will be published in the peer review files if the reviewer signed the comments to authors, or if reviewers explicitly agree to release their name. For more information, please refer to our FAQ page.

Thank you again for your interest in Nature Computational Science. Please do not hesitate to contact me if you have any questions.

Sincerely,

Michelle Badri, PhD
Associate Editor, Research Cross-Journal Editorial Team for Nature Computational Science
Nature Portfolio

ORCID
IMPORTANT: Non-corresponding authors do not have to link their ORCIDs but are encouraged to do so. Please note that it will not be possible to add/modify ORCIDs at proof. Thus, please let your co-authors know that if they wish to have their ORCID added to the paper they must follow the procedure described in the following link prior to acceptance:
https://www.springernature.com/gp/researchers/orcid/orcid-for-nature-research

Author names using non-Roman characters
Nature Portfolio journals can support presentation of author names using non-Roman characters in the HTML version of the article. If you wish to, please include author names in parentheses after the Roman-character spelling; see example online here. Currently supported scripts are:

Arabic, Chinese, Cyrillic, Devanagari, Greek, Hebrew, Hangul, Japanese and Persian. You will be asked to verify the rendering is correct at proof stage.

Reviewer #3 (Remarks to the Author):

The authors’ responses have adequately addressed my concerns.
The remaining issue is that the documentation link provided in the response (https://miracle-docs.readthedocs.io/en/latest/) appears to be inaccessible (returns a “Project not found” error). I guass the latest one is https://miracle-v002.readthedocs.io/en/latest/. I encourage the authors to verify and correct this URL to ensure reproducibility and user accessibility.

Version 2:

Decision Letter:

Dear Professor He,

We are pleased to inform you that your Article "Continual integration of single-cell multimodal data with MIRACLE" has now been accepted for publication in Nature Computational Science.

Once your manuscript is typeset, you will receive an email with a link to choose the appropriate publishing options for your paper and our Author Services team will be in touch regarding any additional information that may be required.

Authors may need to take specific actions to achieve compliance with funder and institutional open access mandates. If your research is supported by a funder that requires immediate open access (e.g. according to Plan S principles or the NIH public access policy) then you should select the gold OA route, and we will direct you to the compliant route where possible. Because authors warrant under our subscription licensing terms that they haven’t committed to licensing any version of their article under a licence inconsistent with the terms of our agreement – including the applicable embargo period – publication under the subscription model isn’t suitable for authors whose funders require no embargo.

If you have any questions about our publishing options, costs, Open Access requirements, or our legal forms, please contact ASJournals@springernature.com

Acceptance of your manuscript is conditional on all authors' agreement with our publication policies (see https://www.nature.com/natcomputsci/for-authors). In particular your manuscript must not be published elsewhere and there must be no announcement of the work to any media outlet until the publication date (the day on which it is uploaded onto our web site).

Before your manuscript is typeset, we will edit the text to ensure it is intelligible to our wide readership and conforms to house style. We look particularly carefully at the titles of all papers to ensure that they are relatively brief and understandable.

Once your manuscript is typeset, you will receive a link to your electronic proof via email with a request to make any corrections within 48 hours. If, when you receive your proof, you cannot meet this deadline, please inform us at rjsproduction@springernature.com immediately.

If you have queries at any point during the production process then please contact the production team at rjsproduction@springernature.com.

You may wish to make your media relations office aware of your accepted publication, in case they consider it appropriate to organize some internal or external publicity. Once your paper has been scheduled you will receive an email confirming the publication details. This is normally 3-4 working days in advance of publication. If you need additional notice of the date and time of publication, please let the production team know when you receive the proof of your article to ensure there is sufficient time to coordinate. Further information on our embargo policies can be found here: https://www.nature.com/authors/policies/embargo.html

An online order form for reprints of your paper is available at https://www.nature.com/reprints/author-reprints.html. All co-authors, authors' institutions and authors' funding agencies can order reprints using the form appropriate to their geographical region.

We welcome the submission of potential cover material (including a short caption of around 40 words) related to your manuscript; suggestions should be sent to Nature Computational Science as electronic files (the image should be 300 dpi at 210 x 297 mm in either TIFF or JPEG format). We also welcome suggestions for the Hero Image, which appears at the top of our home page; these should be 72 dpi at 1400 x 400 pixels in JPEG format. Please note that such pictures should be selected more for their aesthetic appeal than for their scientific content, and that colour images work better than black and white or grayscale images. Please do not try to design a cover with the Nature Computational Science logo etc., and please do not submit composites of images related to your work. I am sure you will

understand that we cannot make any promise as to whether any of your suggestions might be selected for the cover of the journal.

You can now use a single sign-on for all your accounts, view the status of all your manuscript submissions and reviews, access usage statistics for your published articles and download a record of your refereeing activity for the Nature journals.

To assist our authors in disseminating their research to the broader community, our SharedIt initiative provides you with a unique shareable link that will allow anyone (with or without a subscription) to read the published article. Recipients of the link with a subscription will also be able to download and print the PDF.

As soon as your article is published, you will receive an automated email with your shareable link.

We look forward to publishing your paper.

Best regards,

Michelle Badri, PhD
Associate Editor, Research Cross-Journal Editorial Team for Nature Computational Science
Nature Portfolio

P.S. Click on the following link if you would like to recommend Nature Computational Science to your librarian: https://www.springernature.com/gp/librarians/recommend-to-your-library

** Visit the Springer Nature Editorial and Publishing website at www.springernature.com/editorial-and-publishing-jobs for more information about our career opportunities. If you have any questions please click here.**

Open Access This Peer Review File is licensed under a Creative Commons Attribution 4.0 International License, which permits use, sharing, adaptation, distribution and reproduction in any medium or format, as long as you give appropriate credit to the original author(s) and the source, provide a link to the Creative Commons license, and indicate if changes were made.
In cases where reviewers are anonymous, credit should be given to 'Anonymous Referee' and the source.
The images or other third party material in this Peer Review File are included in the article's Creative Commons license, unless indicated otherwise in a credit line to the material. If material is not included in the article's Creative Commons license and your intended use is not permitted by statutory regulation or exceeds the permitted use, you will need to obtain permission directly from the copyright holder.
To view a copy of this license, visit https://creativecommons.org/licenses/by/4.0/

Point-to-point Responses to Reviewers' Comments

Reviewers' Comments:

Reviewer #2 (Remarks to the Author):

This manuscript introduces MIRACLE, a continual learning framework for online integration of single-cell multimodal mosaic data. It addresses a critical challenge by enabling efficient atlas updates without catastrophic forgetting through dynamic architecture adaptation and data rehearsal. The study is well-structured, and benchmarking demonstrates MIRACLE's competitive performance against existing methods. The respiratory infection case study provides biologically interesting findings with translational potential. Below are suggestions to enhance the manuscript's clarity and impact.

Response:

We thank the reviewer for their positive assessment and constructive suggestions. In response to the points raised, we have performed substantial new experiments and external validations, which we believe have significantly strengthened the manuscript.

Major Comments:

1. Terms like "online integration," "continual integration," and "dynamic integration" require clear definitions early in the Introduction to avoid ambiguity. Consistent terminology should be maintained throughout.

Response:

We thank the reviewer for this insightful comment. We agree that a clear and early definition of key terminologies is crucial for readability, and we have revised the manuscript accordingly.

First, to clarify our intended definitions:

- "Continual integration" is the overarching scenario where new data arrives over time, requiring continuous updates to an atlas. This challenge can be addressed by either offline or online integration strategies.
- "Online integration" is a category of methods that update a model using only new data, as opposed to offline methods that reprocess the entire dataset.
- "Dynamic integration" was used as an informal, adjectival phrase to emphasize the evolving nature of the process we now formally define as continual integration.

While we provided formal mathematical definitions for continual integration and online integration in the Methods section ("The scenario of single-cell continual integration" and "Online strategy 1/2/3" in Sec. 4.1), we acknowledge the reviewer's point that these terms should be introduced and distinguished much earlier.

To address this, we have made the following key revisions:

(1) We now explicitly define continual integration as a "scenario" in the Introduction to establish the context of our work clearly.

(2) To eliminate ambiguity, we have replaced "dynamic integration" with "continual integration" to prevent it from being misconstrued as a technical term, retaining "dynamic" only as a general adjective (e.g., "dynamic, shared, and collaborative single-cell analysis").

We have also performed a full-text review to ensure these terms are used consistently throughout the manuscript. We believe these changes significantly improve the paper's clarity.

2. While MIRACLE's efficiency is highlighted, its validation on the 523K-cell DHCM dataset—relatively modest for current large-scale studies—limits this claim. Benchmarking runtime/GPU usage on substantially larger datasets, with direct comparisons to baselines, would strengthen this argument.

Response:

Thank you for your constructive feedback. We agree that benchmarking on a substantially larger dataset is crucial. Accordingly, we have performed a comprehensive benchmark on the Human Lung Cell Atlas (HLCA) [1], a challenging scRNA-seq dataset of 2.3 million cells from 48 studies known for its strong batch effects and biological heterogeneity. We compared MIRACLE-BTS-50K (online), MIRACLE-offline, MIRACLE-transfer, PCA, and Geneformer, with the following results:

(1) Integration accuracy

UMAP plots (Response Fig. 1.1) show that MIRACLE-BTS-50K and MIRACLE-offline effectively remove strong batch effects while preserving cell-type structures, unlike PCA, Geneformer, and MIRACLE-transfer. Quantitative scIB metrics (Response Fig. 1.2) confirm this, with MIRACLE-BTS-50K ranking first with the highest overall score.

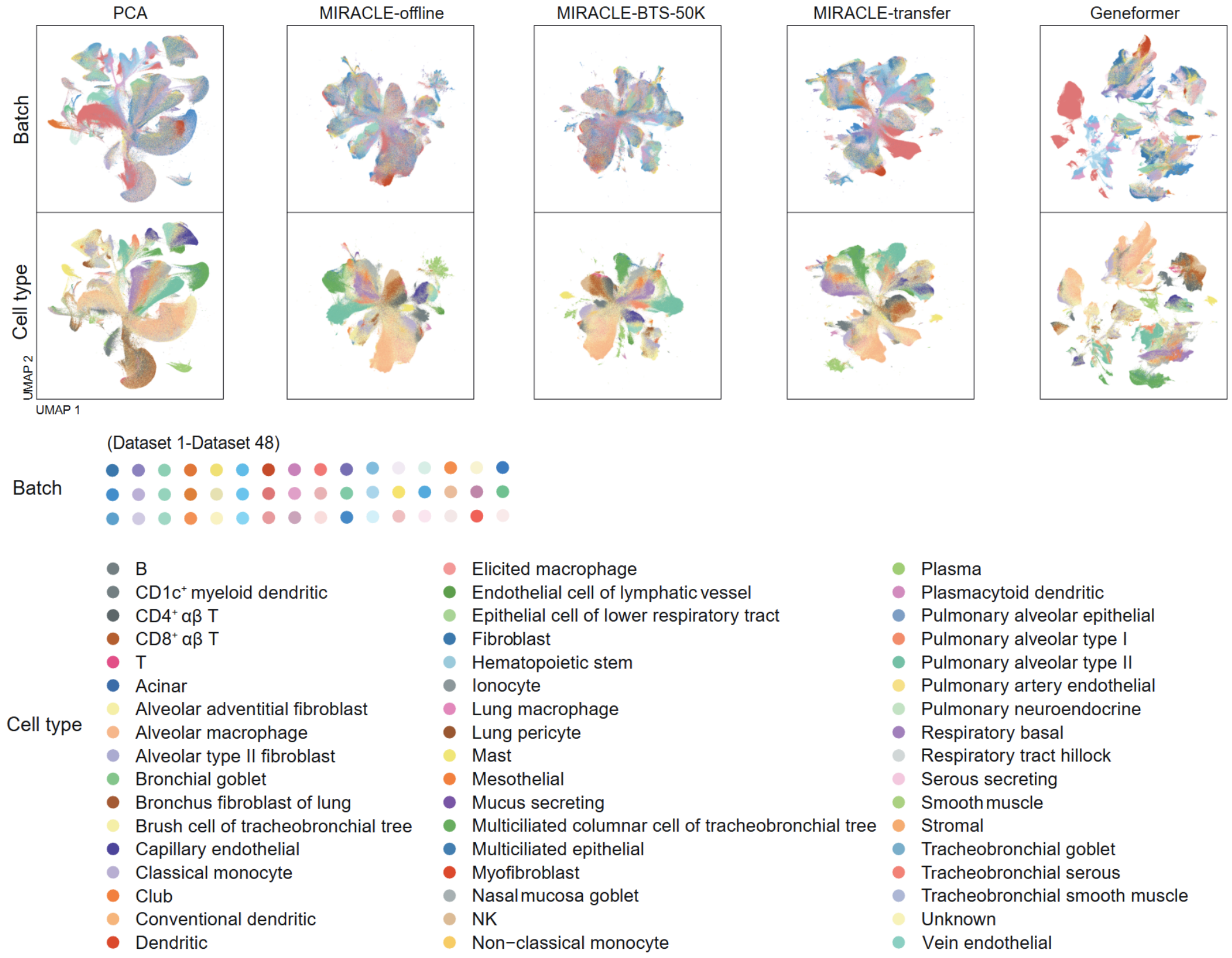

Response Fig. 1.1: UMAP visualization of cell embeddings inferred by different methods on the HLCA scRNA-seq dataset.

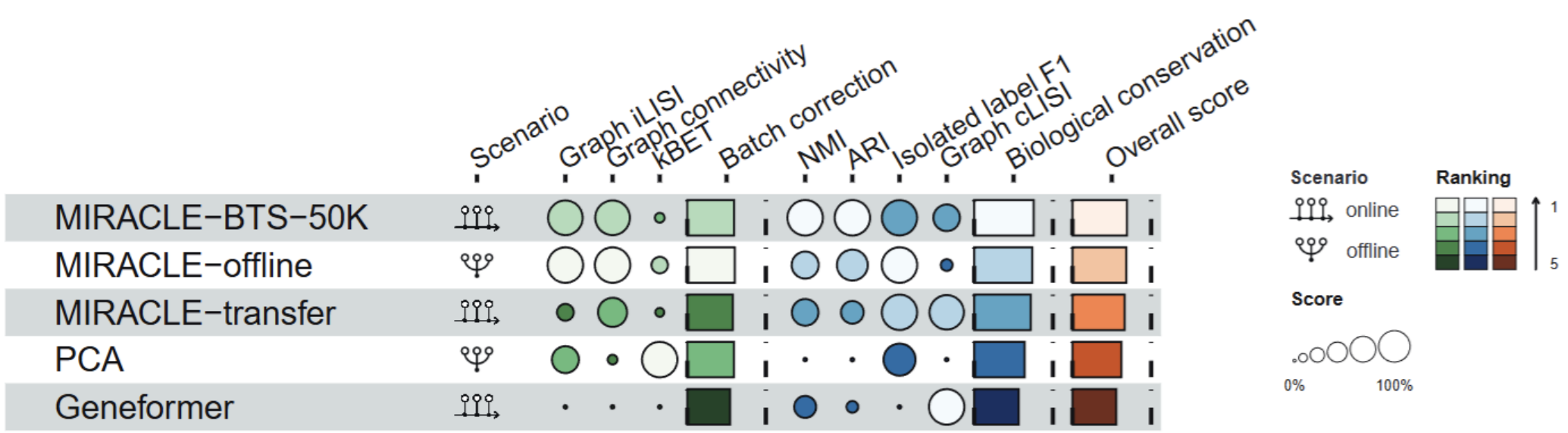

Response Fig. 1.2: Quantitative evaluation using scIB metrics on the HLCA dataset.

(2) Efficiency and scalability

For runtime (Response Fig. 1.3), MIRACLE-BTS-50K was significantly faster than MIRACLE-offline (0.8 day vs. 10 days in cumulative time), demonstrating superior scalability. The fast speeds of Geneformer and PCA are expected, as the former is a pre-trained, inference-only model and the latter is a non-iterative, analytical method.

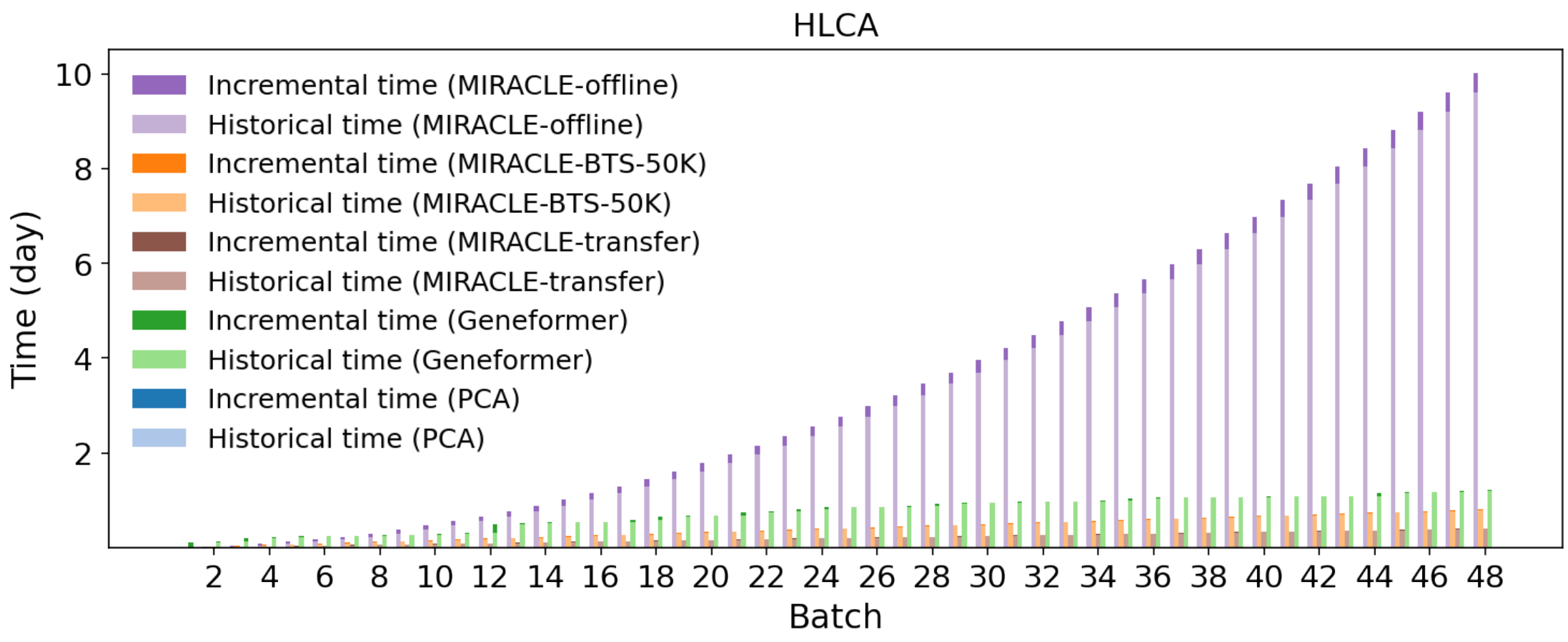

Response Fig. 1.3: Runtime comparison on the HLCA dataset.

Regarding computational memory (Response Fig. 1.4), all MIRACLE methods maintained a constant usage (~2.7 GB) due to their mini-batch training strategy, ultimately consuming less memory than other methods, whereas PCA's memory requirement grows with the data, posing a scalability bottleneck. Further analysis can be found in our response to Reviewer #3's Comment 1.

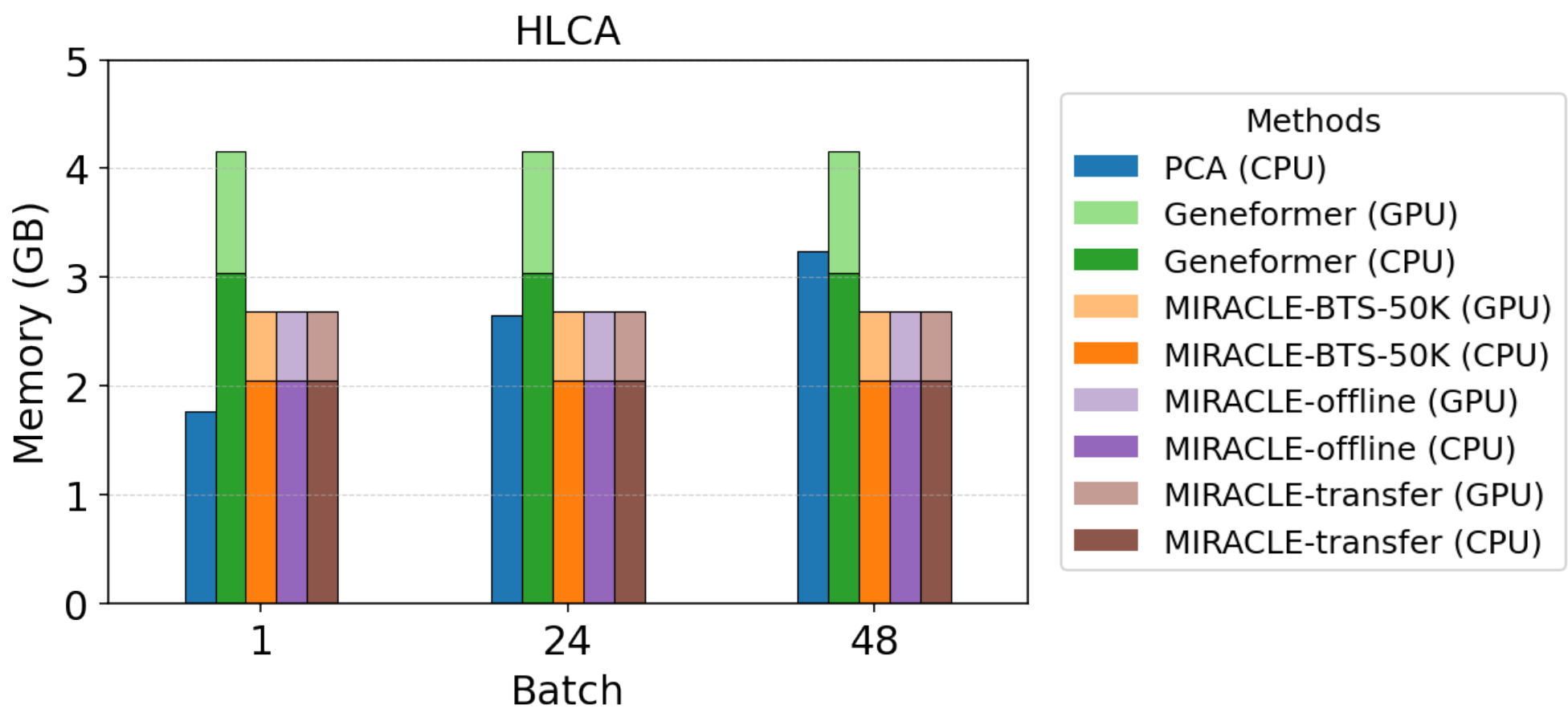

Response Fig. 1.4: Computational memory usage on the HLCA dataset.

In summary, these results demonstrate MIRACLE's superior accuracy and scalability on an ultra-large-scale dataset. We have incorporated these findings into the Results Sec. 2.2, Supplementary Fig. 4 and Supplementary Table 2 to strongly validate our claims.

References

[1] Sikkema, Lisa, et al. "An integrated cell atlas of the lung in health and disease." *Nature medicine* 29.6 (2023): 1563-1577.

3. Since MIRACLE builds on MIDAS, it is required to briefly describe MIDAS's core methodology and explicitly detail how MIRACLE extends or improves upon it.

Response:

We thank the reviewer for this constructive feedback. As suggested, we have revised the Methods section (Sec. 4.4) to explicitly define the core methodology of MIDAS and clarify MIRACLE's relationship to it.

Specifically, MIDAS is a deep probabilistic framework designed for static, offline single-cell data integration. Its primary strength is handling "mosaic data", where batches have varying modality combinations. It uses a multimodal VAE architecture with self-supervised learning to align modalities and information-theoretic methods to disentangle biological states from technical noise, enabling dimensionality reduction, batch correction, and imputation in a single, one-off integration task.

MIRACLE, in contrast, is a general-purpose online learning framework designed for the continual, incremental integration of single-cell atlases. To handle complex mosaic data within this dynamic context, we chose MIDAS as its base model. However, MIDAS's offline design means it cannot inherently address catastrophic forgetting or adapt to new modalities arriving sequentially. MIRACLE overcomes these limitations by introducing a suite of continual learning strategies. Its core innovations include (1) a dynamic architecture adaptation mechanism to incorporate new modalities and (2) a data rehearsal memory mechanism, featuring our novel Ball Tree Sampling, to efficiently counteract knowledge forgetting. These capabilities are not part of MIDAS's original design.

Thus, MIRACLE's novelty lies in establishing a new framework that addresses the distinct scientific challenge of online, dynamic atlas construction. It achieves this by leveraging and extending MIDAS as a foundational component, equipping it with the necessary continual learning capabilities.

We believe this clarification, now integrated into Sec. 4.4, effectively delineates the scope of both models and better highlights MIRACLE's unique contributions. We have also added Supplementary Note 4, detailing the loss functions that MIRACLE inherits from MIDAS.

4. Figure 2's evaluation uses a single-nucleus RNA-seq dataset with apparently mild batch effects (per PCA). It is necessary to assess the integration performance on datasets with pronounced technical artifacts for a more rigorous test.

Response:

Thank you for your insightful comment, which has significantly enhanced the rigor of our work. We completely agree that testing on a dataset with pronounced batch effects is a crucial validation step.

In fact, in response to your Comment 2, we performed a comprehensive benchmark on precisely such a dataset: the HLCA. Comprising 2.3 million cells from 48 distinct studies, this atlas is characterized by strong technical artifacts arising from its diverse origins (e.g., different labs, technologies, and sample processing methods), making it an ideal test case.

Our new results, detailed in our response to your Comment 2, clearly demonstrate the following:

- The initial PCA plot (Response Fig. 1.5) confirms these strong artifacts, with cells clustering distinctly by their study of origin.
- After integration with MIRACLE-BTS-50K, these batch effects are eliminated, while biological cell types remain accurately preserved.
- Quantitative scIB metrics (Response Fig. 1.6) further substantiate this, ranking MIRACLE-BTS-50K first overall for its superior performance in both batch correction and biological conservation.

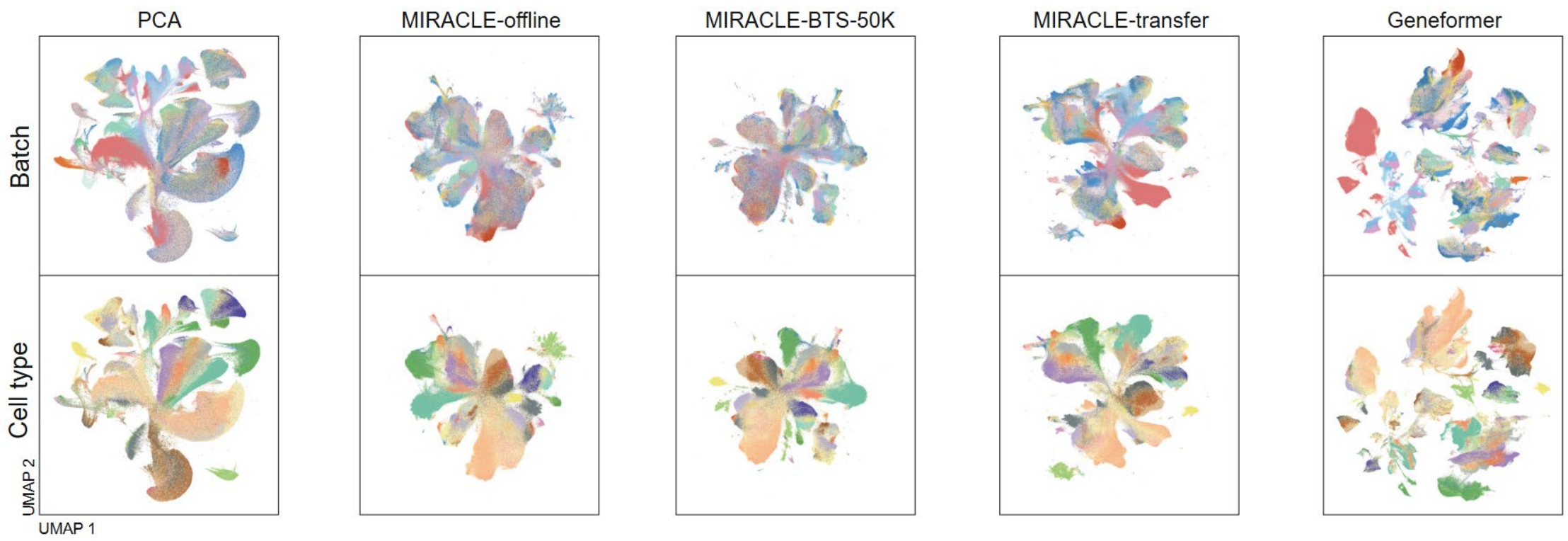

Response Fig. 1.5: UMAP visualization of cell embeddings inferred by different methods on the HLCA dataset.

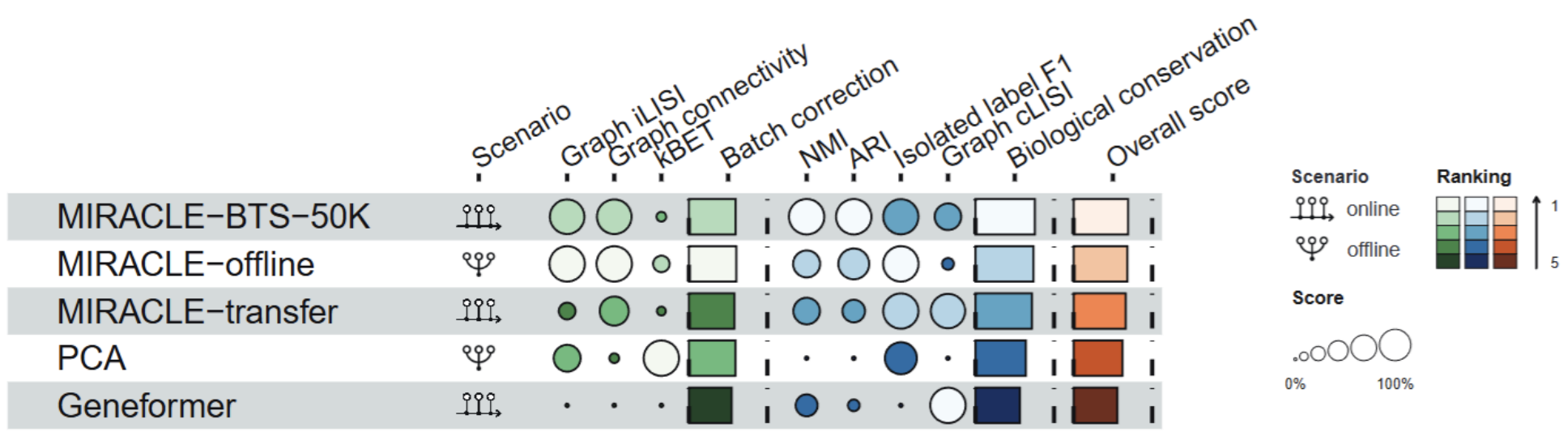

Response Fig. 1.6: Quantitative evaluation using scIB metrics on the HLCA dataset.

We acknowledge that our original analysis in Results Sec. 2 focused primarily on computational efficiency. Your suggestion rightly highlighted the need for a more challenging test case. Our new experiment on the HLCA dataset now simultaneously validates both MIRACLE's scalability and its robustness against strong batch effects. Accordingly, we have incorporated these findings into Results Sec. 2.2, Supplementary Fig. 4, and Supplementary Table 2.

5. The attribution of transfer learning's poor performance to catastrophic forgetting lacks direct evidence. Please provide explicit analysis (e.g., forgetting metrics or ablation studies) to establish this causal link.

Response:

We thank the reviewer for this insightful suggestion. We agree that our claim regarding catastrophic forgetting in MIRACLE-transfer requires more direct, quantitative evidence. To

address this, we have conducted a new analysis specifically designed to measure and compare the knowledge retention of MIRACLE (uses continual learning), against MIRACLE-transfer (uses transfer learning). We believe these additions have significantly strengthened our study and are grateful for the feedback.

Our analysis employs two established metrics (Methods 4.13) from the Continual Learning field: (1) Average Accuracy (ACC), which evaluates the model's average performance across all historical tasks at the end of training, and (2) Backward Transfer (BWT), which directly quantifies the impact of learning new tasks on performance on previously learned ones, where a significant negative value is a hallmark of catastrophic forgetting.

We began by calculating the integration performance score $y_{t,\tau}$—that is, the performance on the cumulative first τ batches after the model had been trained up to step t (for all $1 \leq \tau \leq t \leq T$, where T is the final step). The evolution of these scores, now visualized in a new series of plots (Response Fig. 1.7), reveals a stark contrast between the two models:

- The performance curve for MIRACLE (red line) remains remarkably stable throughout the process as t increases, indicating that it effectively retains knowledge about past data while learning from new data.
- Conversely, the curve for MIRACLE-transfer (blue line) exhibits a pronounced downward trend, particularly on the more complex DOTEA dataset. This demonstrates that as the model is continually updated, it progressively "forgets" what it learned from earlier tasks.

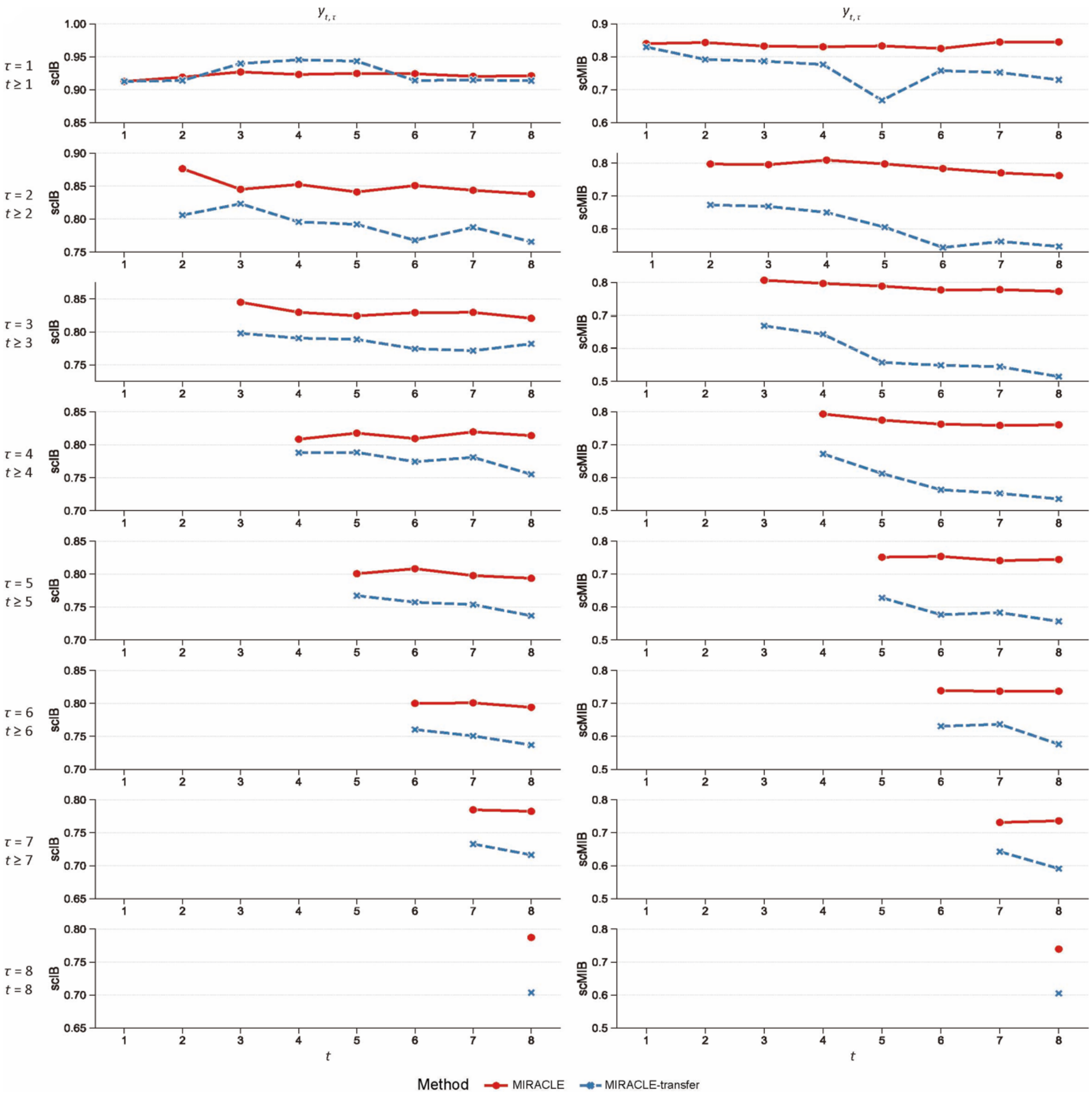

Response Fig. 1.7: Evolution of integration performance for MIRACLE and MIRACLE-transfer over sequential training on the WNN (left) and DOTEA (right) datasets. Each plot shows the performance score ($y_{t,\tau}$) on the first τ cumulative batches (rows) after training up to step t (x-axis).

These performance scores ($y_{t,\tau}$) further enabled us to compute the ACC and BWT summary metrics for a final quantitative comparison. The results (Response Fig. 1.8) show that:

- On both the WNN and DOTEA datasets, MIRACLE achieves clearly better metrics (higher ACC and a BWT value closer to zero).
- Notably, on the DOTEA dataset, the BWT of MIRACLE-transfer is far more negative than that of MIRACLE. This quantitatively confirms that MIRACLE-transfer suffers from severe catastrophic forgetting, a problem that MIRACLE's continual learning strategy successfully mitigates.

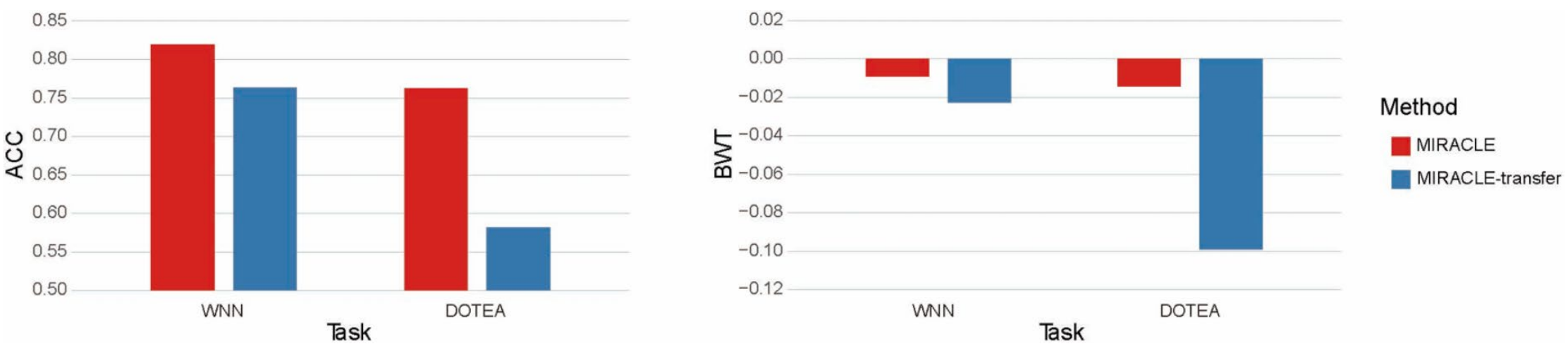

Response Fig. 1.8: Using ACC and BWT metrics to evaluate catastrophic forgetting for MIRACLE and MIRACLE-transfer on the WNN and DOTEA datasets.

This analysis provides strong, direct evidence for our central claim that MIRACLE achieves superior continual integration performance by effectively mitigating catastrophic forgetting. We have incorporated these findings into Results Sections 2.3 and 2.4 and Supplementary Fig. 6, and have detailed the definition of ACC and BWT metrics in a new Methods Sec. 4.13.

6. How does MIRACLE handle sparse ATAC peaks, noisy ADT signals, or missing modalities? These are very common issues in practice.

Response:

We thank the reviewer for this insightful question. MIRACLE addresses these real-world challenges within a continual learning framework by extending the prior work, MIDAS—a powerful but static generative model. Its foundational mechanisms, inherited from MIDAS, provide inherent robustness:

(1) For sparse ATAC peaks, the model uses strong regularization, including the VAE's intrinsic low-dimensional bottleneck, KL divergence, and Dropout, to prevent overfitting on sparse, high-dimensional features. This is enhanced by cross-modal regularization, where denser modalities (e.g., transcriptome) inform the representation of sparser ones. Critically, Layer Normalization counteracts vanishing gradients from sparse data, ensuring stable and effective training.

(2) For noisy ADT signals, the model similarly leverages the VAE's inherent denoising properties (bottleneck, KL divergence, and Dropout) to learn a smooth, generalizable representation rather than memorizing noise. This is further strengthened by cross-modal regularization, e.g., the richer transcriptome signal guides the model to learn true protein expression patterns over noise.

(3) For missing modalities, MIRACLE's key innovation is to transform a static solution into a dynamic one. The MIDAS foundation already provides an efficient Product of Experts (PoE) framework with a modular design (M encoders instead of 2^M - 1) to handle any given static combination of missing data. MIRACLE extends this crucially by making the architecture dynamically extensible, allowing new modalities to be added over time without full retraining (Fig. 1a). The framework also enables functional modality imputation; by aligning modalities in the latent space, it generates a complete data view vital for downstream analyses like cell typing and trajectory inference. These capabilities provide a flexible solution for the evolving nature of real-world data analysis.

7. The cross-tissue integration results are encouraging, but the biological implications and potential issues should be discussed in more depth. For instance, is there a risk of mixing tissue-resident and circulating cell states (e.g., T cells from PBMCs vs. spleen)? It would be helpful to address how MIRACLE mitigates or interprets such ambiguity.

Response:

We thank the reviewer for this insightful point. Conflating distinct cell populations, like tissue-resident versus circulating T cells, is a known risk of overcorrection in data integration. To address this, our new analyses demonstrate that MIRACLE's design inherently mitigates this risk.

While existing methods struggle with the trade-off between removing batch effects and preserving biological variation, MIRACLE addresses it via the intrinsic properties of its deep generative model. As a Variational Autoencoder (VAE), its primary goal of accurate data reconstruction incentivizes preserving strong biological signals. This creates an implicit regularization from the model's finite capacity: the model lacks the flexibility to first erase complex biological variations by mapping them to a homogenized latent space (i.e., overcorrect) and then perfectly reconstruct their original, distinct profiles. To minimize reconstruction loss, it is therefore forced to preserve these biological differences in its low-dimensional representation. This effect strengthens during continual integration as more data better defines the biological manifold, making overcorrection increasingly costly.

To test this, we analyzed T cells from our cross-tissue atlas (Fig. 5b). We isolated these T cells, downsampled the dominant PBMC population to prevent bias, and performed high-resolution sub-clustering (Response Fig. 1.9a–c). This revealed distinct, tissue-specific populations that MIRACLE kept separate, including a PBMC-enriched cluster (cluster 17, 97.3% PBMC) and a spleen-enriched cluster (cluster 24, 90.2% spleen).

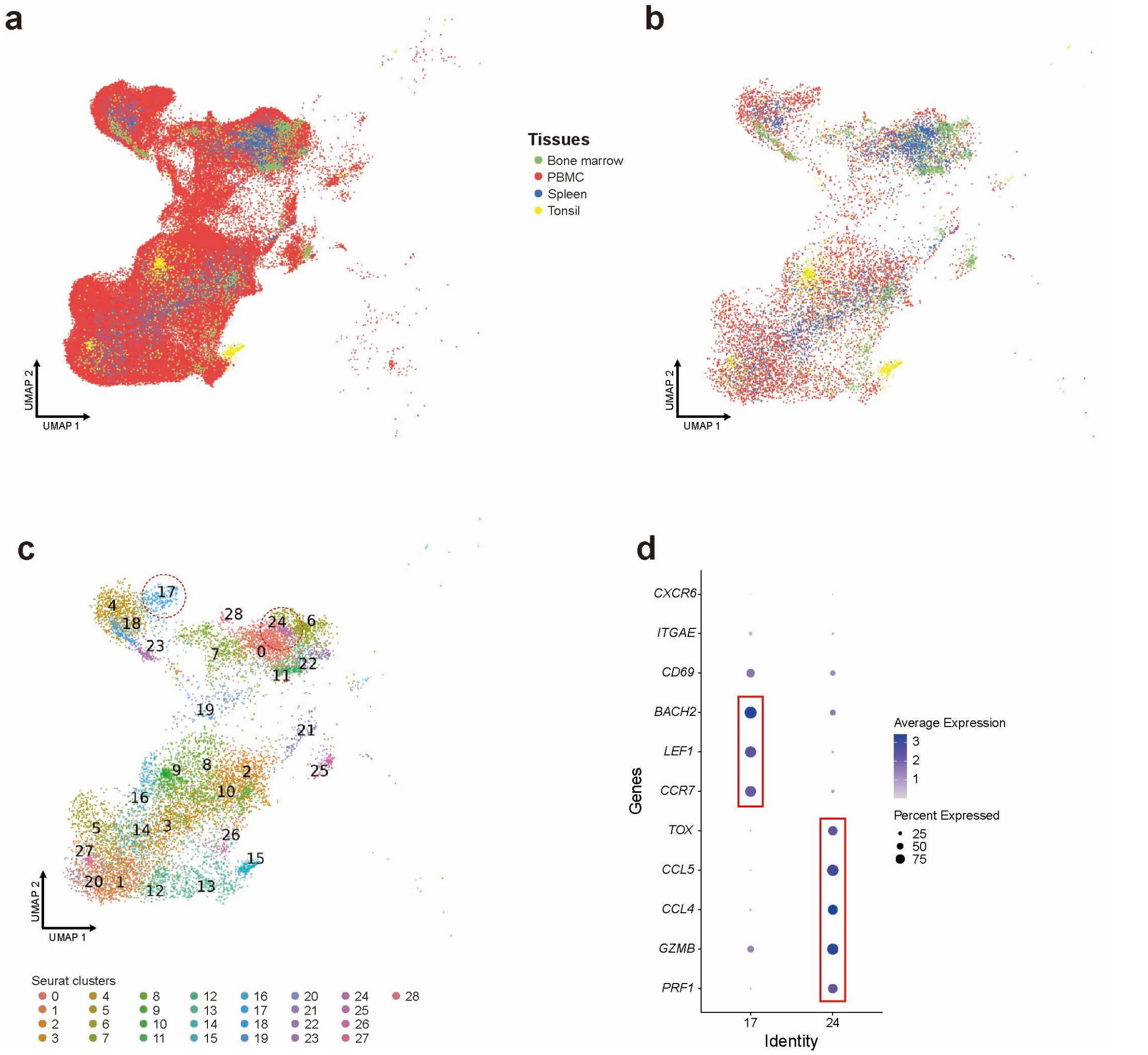

Response Fig. 1.9: MIRACLE preserves distinct tissue-specific T cell states. **a–c**, UMAP plots of T cells from the cross-tissue atlas, showing (**a**) all isolated cells, (**b**) the population after downsampling PBMCs, and (**c**) the resulting high-resolution sub-clusters. **d**, Dot plot of differentially expressed genes between the PBMC-enriched cluster 17 and the spleen-enriched cluster 24.

Differential expression analysis (Response Fig. 1.9d) confirmed their opposing functional identities:

- Cluster 17 (PBMC) represents early-differentiated, circulating memory cells, defined by the homing receptor *CCR7* and transcription factors *LEF1* and *BACH2* that suppress the effector program.
- Cluster 24 (spleen), in contrast, represents terminally differentiated effector cells, expressing cytotoxic (*PRF1*, *GZMB*), recruitment (*CCL4*, *CCL5*), and terminal differentiation (*TOX*) genes. Notably, this cluster lacks classic tissue-residency markers (*CD69*, *ITGAE*, *CXCR6*), likely because its transcriptional program is dominated by its effector function, a signature of its role as a potent, on-site effector cell.

This functional dichotomy, driven by opposing transcription factors (*TOX* vs. *LEF1*/*BACH2*), provides strong evidence against cell state conflation. It shows that MIRACLE resolves the gene regulatory networks driving cell identity, thus preserving biological insights in cross-tissue atlases. We have incorporated these analyses into Results Sec. 2.5 and Supplementary

Fig. 14.

Nevertheless, we acknowledge that MIRACLE is not immune to overcorrection—a risk that becomes pronounced with small datasets or severe compositional differences (e.g., T cells in one batch, B cells in another). Future work will explore enhancing MIRACLE's robustness by introducing tissue-specific latent variables, leveraging semi-supervised information like cell-type labels when available, and adding novel regularization terms through self-supervised or multi-task learning.

8. Continual learning methods can be sensitive to the order in which data are introduced. It is very important to test MIRACLE with varied input sequences (e.g., TB before/after Flu A) and to report performance stability or mitigation strategies.

Response:

We thank the reviewer for this insightful question, which has significantly strengthened our study. We agree that sensitivity to data integration order is a critical aspect of continual learning that demands rigorous evaluation. This order-dependency is a known phenomenon and is intentionally leveraged by strategies like curriculum learning to optimize training. This underscores the importance of assessing our model's robustness.

Following your suggestion, we systematically evaluated MIRACLE's robustness by testing the five other possible permutations of the disease integration sequence from Results Sec. 2.7. For each new sequence, we performed continual integration and subsequent manual cell-type refinement, then compared the outcomes to our original results. The model's stability was confirmed across all orders:

- The UMAP plots of final integrated atlases showed highly consistent cellular topologies (Response Fig. 1.10), and key findings, such as the $CD4^+$ CTLs and $HERC1^+$ T cells, were robustly identified, confirming they are not sequence-dependent artifacts.

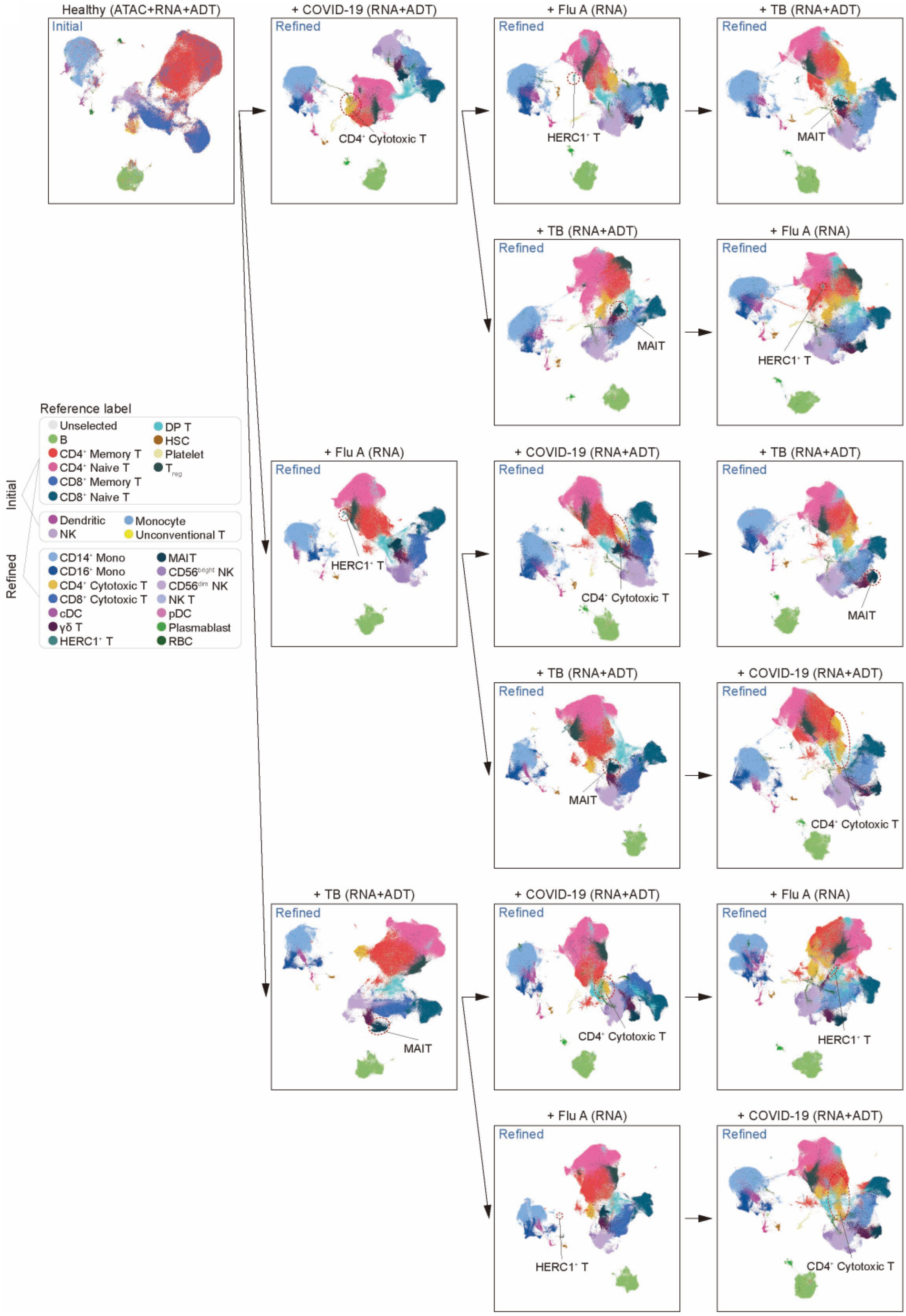

Response Figure 1.10: UMAP visualization of MIRACLE-inferred cell embeddings for cross-disease atlas construction under different integration orders. Row 1: the original order. Rows 2 to 6: the permuted order. Column 1: healthy PBMC atlas colored by initial reference labels. Columns 2 to 4: updated PBMC atlases colored by manually-refined reference labels. Regions with major updates to cell types are highlighted (red circles).

- Quantitative comparison of the refined cell-type labels (permuted vs. original order) revealed high concordance (micro F1 score: 0.86 ± 0.02; Response Fig. 1.11), demonstrating stable cell identity assignment.

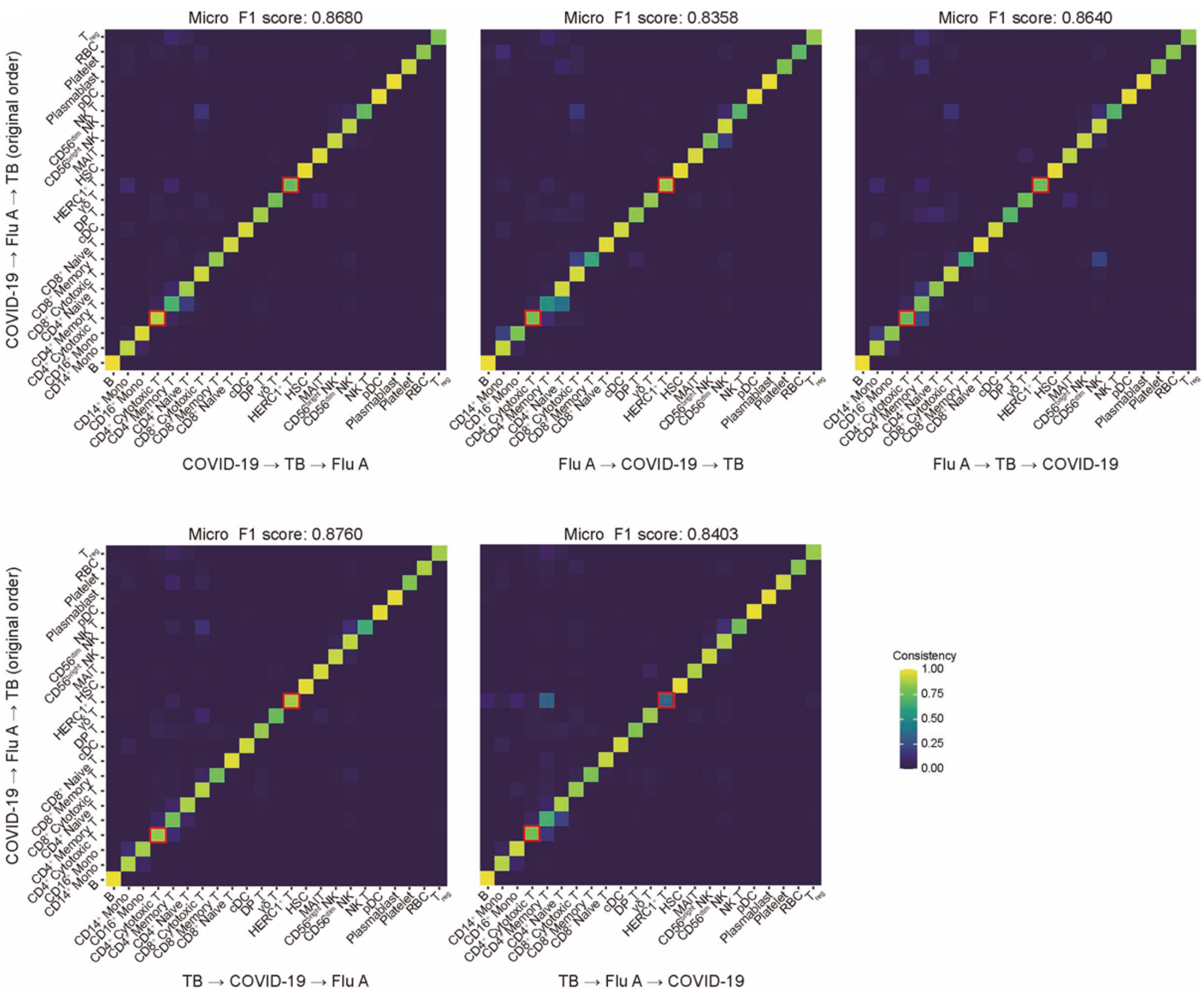

Response Figure 1.11: Confusion plot comparing cell-type labels generated from the permuted and original integration orders in cross-disease atlas construction.

These results are now incorporated into the manuscript (Sec. 2.7 and Supplementary Fig. 27).

MIRACLE's robustness stems from its data rehearsal strategy. By training on both new data and a "rehearsal memory" sampled from all prior data, the model constantly reviews its learning history. This approach mitigates dependency on the most recent task and significantly reduces sensitivity to the overall sequence.

Finally, to your point about further mitigation strategies, we have added a forward-looking statement to the Discussion. While our data rehearsal method proves effective, we see great potential in exploring more advanced order-robust continual learning techniques [1,2]. This could further enhance model stability, particularly for longer and more complex integration sequences in future applications.

References

[1] Yoon, Jaehong, et al. "Scalable and order-robust continual learning with additive parameter decomposition." In *International Conference on Learning Representations* https://openreview.net/forum?id=r1gdj2EKPB (2020).

[2] Li, Jiaqi, et al. "Hessian aware low-rank perturbation for order-robust continual learning." *IEEE Transactions on Knowledge and Data Engineering* 36.11 (2024): 6385-6396.

9. The identification of HERC1^{+} T cells in Flu A and CD4^{+} CTLs in COVID-19 is interesting, but external validations are required to support these findings. Are these populations reproducible in independent datasets or preprocessing pipelines?

Response:

We thank the reviewer for this excellent and critical suggestion. We agree that external validation is crucial for novel biological findings. As recommended, we performed a systematic validation for both CD4^{+} CTLs and HERC1^{+} T cells from two perspectives: reproducibility in an independent dataset and reproducibility using an independent analysis pipeline.

(1) Validation of CD4^{+} CTLs in an independent COVID-19 dataset

We analyzed a large-scale COVID-19 scRNA-seq immune atlas [1], focusing on PBMCs from patients with mild/moderate disease. Using a standard Harmony-based Seurat integration pipeline (https://satijalab.org/seurat/reference/harmonyintegration), we identified a distinct cluster (Cluster 24) within the CD4^{+} T cell compartment (Response Fig. 1.12a–c). These cells co-expressed canonical CD4^{+} T cell markers (*CD3D*, *CD4*) and key cytotoxicity genes (*GZMB*, *GNLY*), confirming their identity as CD4^{+} CTLs (Response Fig. 1.12d). To further validate this quantitatively, we calculated a cytotoxicity score, which was significantly higher in this population compared to other CD4^{+} T cell subsets (Response Fig. 1.12e). This result successfully reproduces our finding in a completely independent cohort.

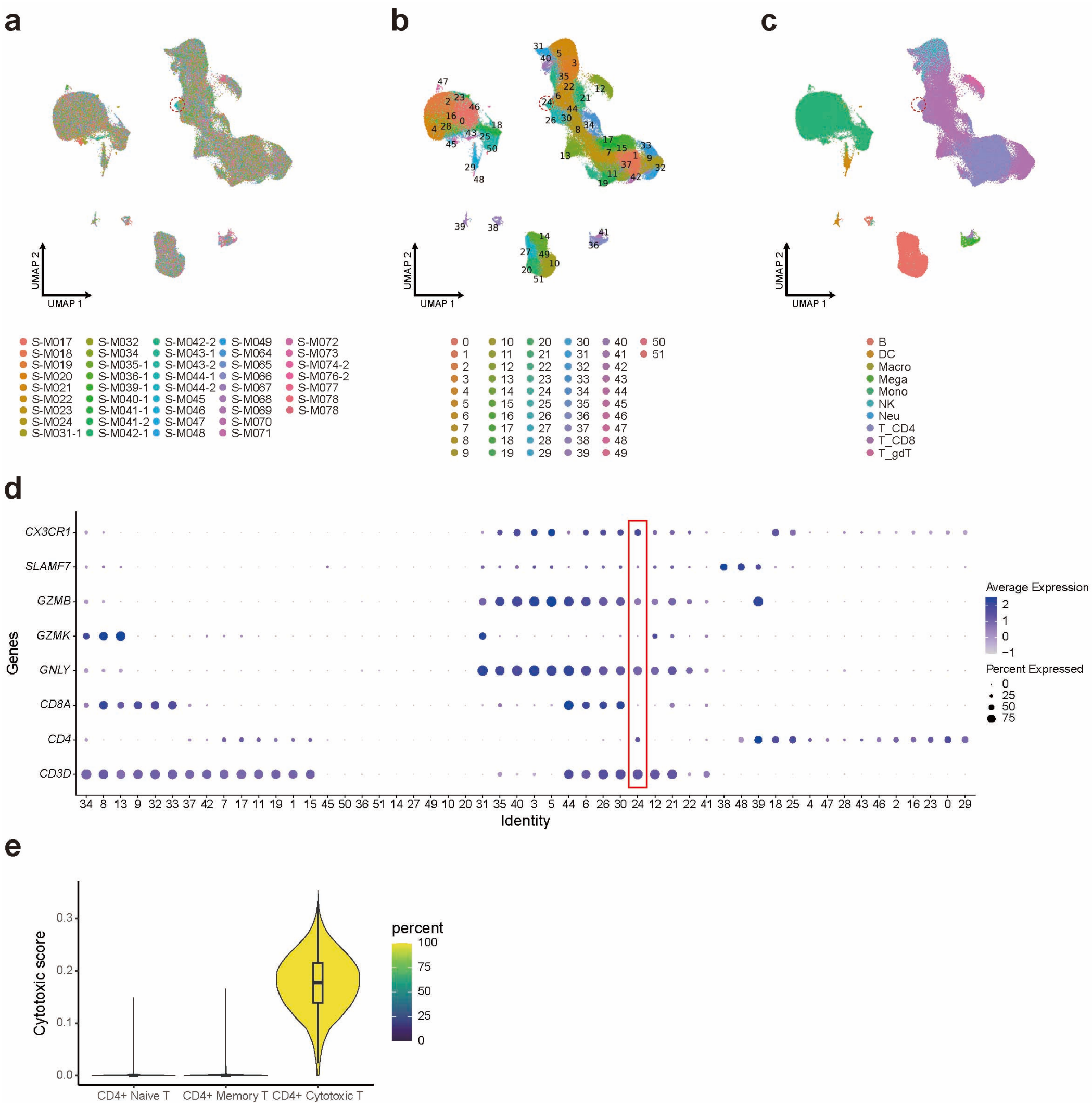

Response Fig. 1.12: Validation of CD4$^+$ CTLs in an independent COVID-19 dataset. **a–c**, UMAP plots of an independent COVID-19 dataset integrated by Harmony, colored by (**a**) batches, (**b**) clusters, and (**c**) original cell labels. **d**, Dot plot confirming co-expression of CD4$^+$ T cell (*CD3D*, *CD4*) and cytotoxic (*GZMB*, *GNLY*) markers in Cluster 24. **e**, Box plot showing significantly higher cytotoxicity scores in CD4$^+$ CTLs compared to other CD4$^+$ T cell subsets.

(2) Validation of CD4$^+$ CTLs using an independent preprocessing pipeline

We re-analyzed our original COVID-19 dataset using the same Harmony-based pipeline. This alternative approach robustly recapitulated the identification of CD4$^+$ CTLs (Cluster 7), which showed a highly consistent expression profile of key marker genes (*CD3D*, *CD4*, *GZMB*, *GZMK*, *GNLY*) compared to our original finding (Response Fig. 1.13a–d, cf. Fig. 7c). The distribution of this population across disease severities was also similar to our MIRACLE results (Response Fig. 1.13e, cf. Supplementary Fig. 24e). A substantial overlap was observed between the cells identified by both methods, confirming the robustness of this discovery (Response Fig. 1.13f).

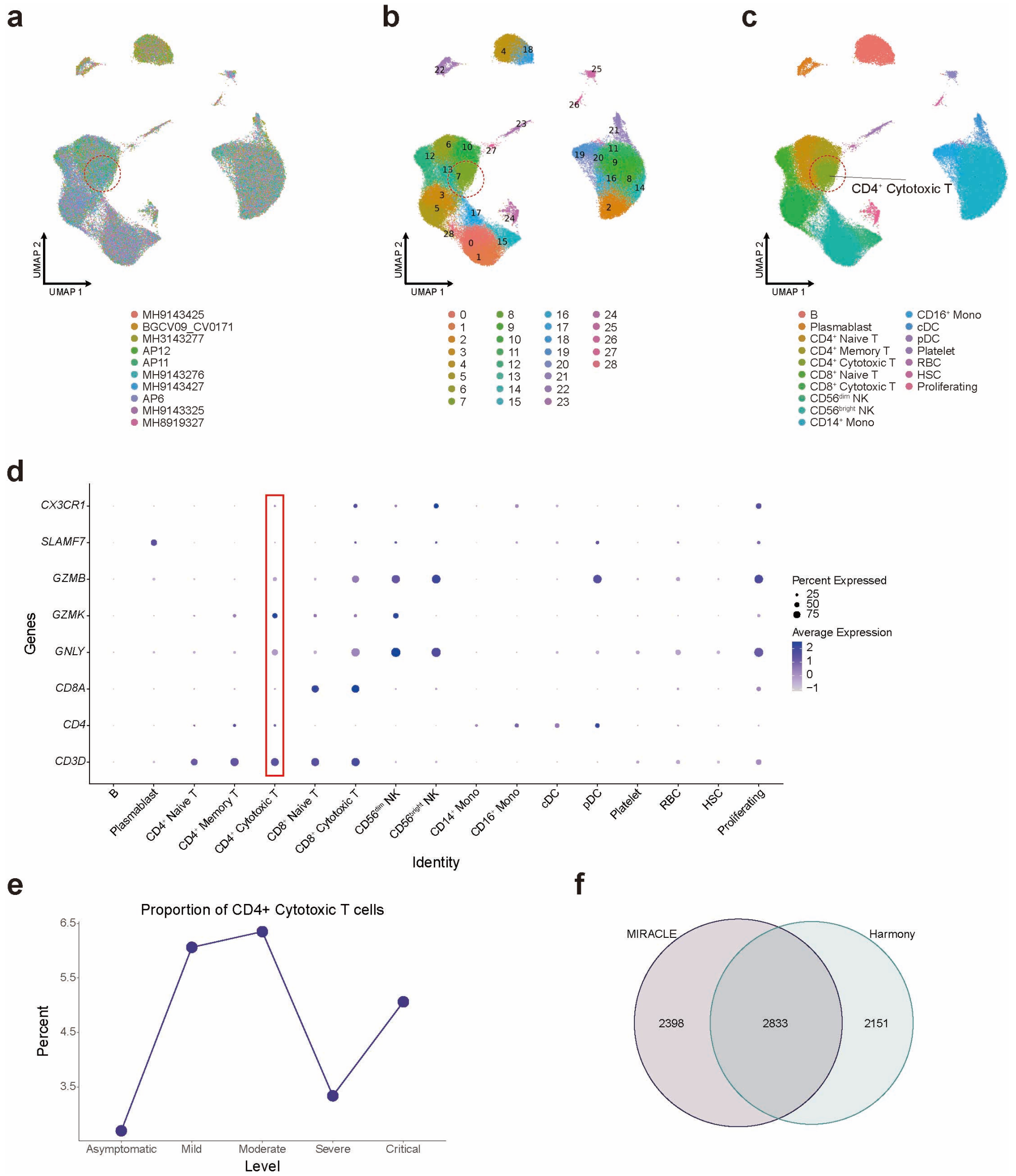

Response Fig. 1.13: Validation of CD4$^+$ CTLs using an independent pipeline on the original dataset. **a–c**, UMAP plots from a Harmony-based pipeline on the original data, identifying (**a–c**) the CD4$^+$ CTL cluster (Cluster 7). **d**, Dot plot showing consistent CD4$^+$ CTL marker expression in Cluster 7. **e**, Proportions of CD4$^+$ CTLs across disease severities, recapitulating the trend found by MIRACLE. **f**, Venn diagram showing the overlap of CD4$^+$ CTLs identified by MIRACLE and Harmony.

(3) Validation of HERC1$^+$ T cells in an independent Flu A dataset

The availability of public Flu A PBMC datasets suitable for validating this specific cell population is limited. In one such analyzed dataset [2], we found a weak signal for HERC1-expressing T cells in a small subset of batches. Our analysis identified a candidate cluster (Cluster 8) expressing T cell markers and the key genes *HERC1*, *BRAF*, and *PTBP2*, but the signal was not strong enough for a definitive confirmation (Response Fig. 1.14). We infer that the challenge in replicating this specific subpopulation may stem from the inherent biological heterogeneity of influenza infection (e.g., viral strain, host genetics, sampling time). This observation, rather than invalidating the finding, may highlight the context-dependent nature of

this cell state and underscores the unique sensitivity of MIRACLE in capturing such rare or transient populations.

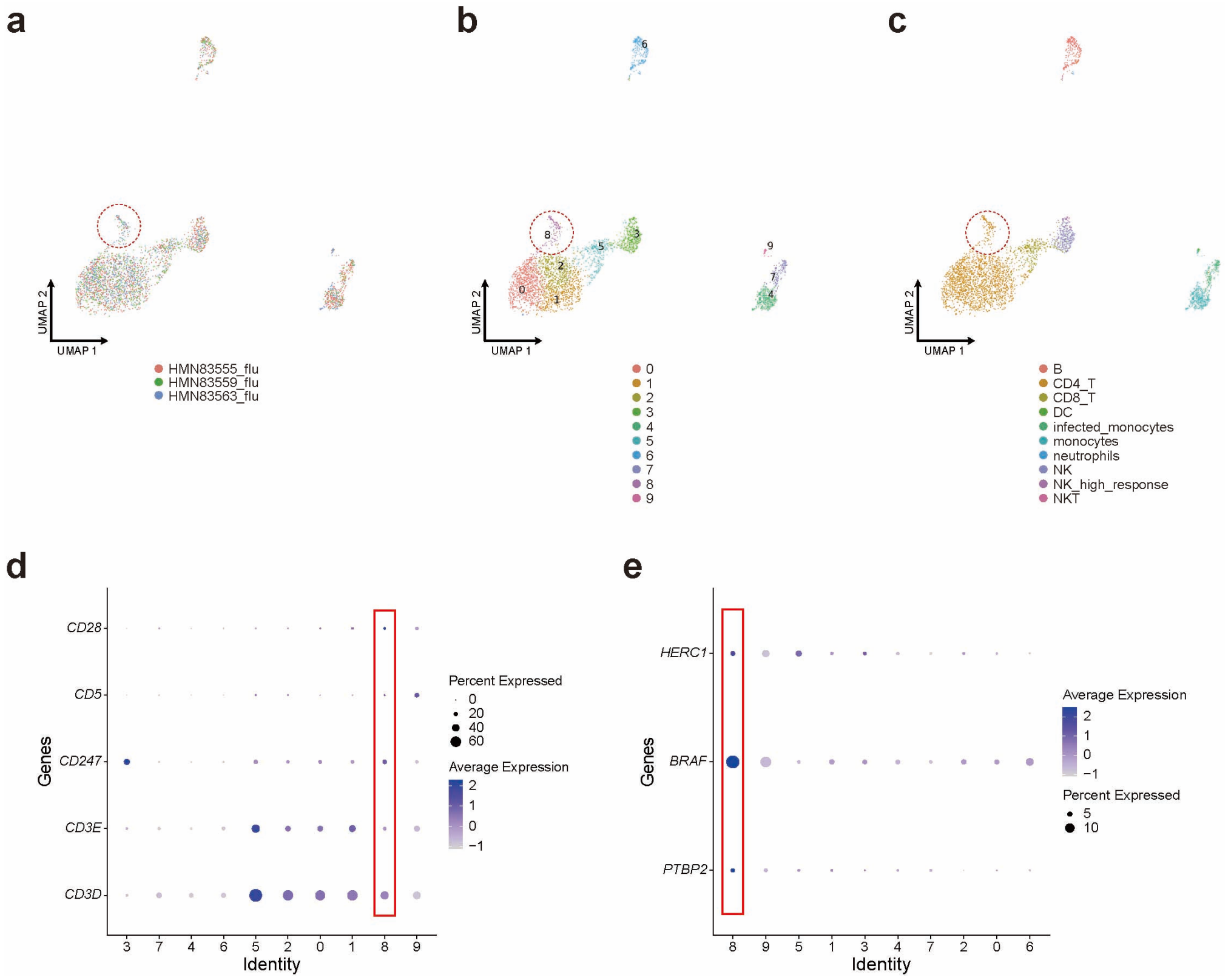

Response Fig. 1.14: Attempted validation of HERC1^{+} T cells in an independent Flu A dataset. **a–c**, UMAP plots of an independent Flu A dataset, showing (**a–c**) the candidate HERC1^{+} T cell cluster (Cluster 8). **d**, Dot plot showing T cell markers in Cluster 8. **e**, Dot plot showing weak expression of HERC1^{+} T-associated genes (*HERC1*, *BRAF*, *PTBP2*) in Cluster 8.

(4) Validation of HERC1^{+} T cells using an independent preprocessing pipeline

Crucially, when we re-analyzed our original Flu A dataset with the Harmony-based pipeline, we robustly identified a HERC1^{+} T cell population (Cluster 12). Its marker gene expression pattern was highly consistent with the population identified by MIRACLE (Response Fig. 1.15a–e, cf. Supplementary Fig. 25a). Furthermore, the cells identified by Harmony were largely a subset of those identified by MIRACLE, demonstrating high concordance between the methods (Response Fig. 1.15f). This result confirms that the HERC1^{+} T cell population is a technically robust finding derived from a genuine biological signal within the data.

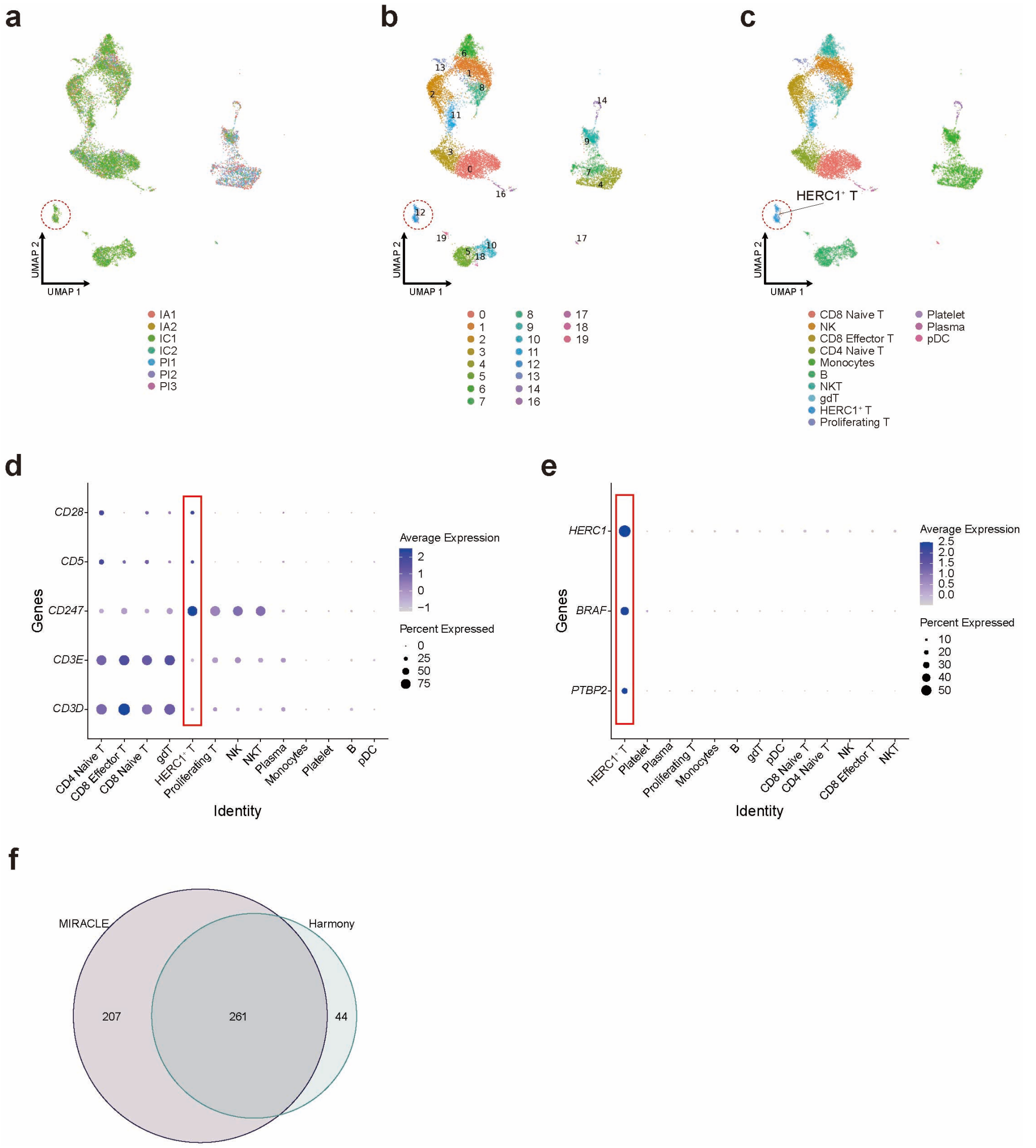

Response Fig. 1.15: Validation of HERC1^{+} T cells using an independent pipeline on the original dataset. **a–c**, UMAP plots from a Harmony-based pipeline on the original Flu A data, identifying (**a–c**) the HERC1^{+} T cell cluster (Cluster 12). **d**, **e**, Dot plots confirming expression of (**d**) T cell markers and (**e**) key HERC1^{+} T-associated genes (*HERC1*, *BRAF*, *PTBP2*) in Cluster 12. **f**, Venn diagram showing substantial overlap of HERC1^{+} T cells identified by MIRACLE and Harmony.

In summary, the existence of CD4^{+} CTLs in COVID-19 was successfully validated by both an independent dataset and an alternative pipeline. The HERC1^{+} T cell population in Flu A was robustly reproduced by an alternative pipeline, and its rarity in other datasets highlights the sensitivity of MIRACLE. These comprehensive validations provide strong evidence that our findings represent genuine and robust biological signals, not computational artifacts. This reinforces our confidence in the biological relevance of these novel cell states and establishes MIRACLE as a powerful and reliable engine for dynamic scientific discovery. We have incorporated these findings into Results Sec. 2.7 and a new Supplementary Note 2.

References

[1] Ren, Xianwen, et al. "COVID-19 immune features revealed by a large-scale single-cell transcriptome atlas." *Cell* 184.7 (2021): 1895-1913.

[2] Randolph, Haley E., et al. "Genetic ancestry effects on the response to viral infection are pervasive but cell type specific." *Science* 374.6571 (2021): 1127-1133.

10. The manuscript highlights the importance of data sharing and collaboration. How MIRACLE's pipeline addresses privacy compliance?

Response:

Thank you for this critical question. We fully agree that protecting participant privacy is a paramount ethical and legal requirement when handling human single-cell data, and we have given this careful consideration in designing MIRACLE's collaborative workflow.

It's helpful to first distinguish between the two collaboration modes MIRACLE supports: (1) In the simplest case, a *unidirectional atlas application*, a user downloads the reference atlas to use locally for their own data integration or label transfer. Since their private data never leaves their local environment, this mode is inherently privacy-preserving. (2) The privacy challenge arises in the more complex mode: *multi-party collaborative construction*. Here, for multiple users to build an atlas together, they would need to share not just the updated model but also the rehearsal memory. We certainly acknowledge that sharing this memory, which contains a small amount of real cell data, presents a hurdle for collaborations under strict privacy frameworks (e.g., clinical).

Nevertheless, this design choice was intentional, born from a pragmatic trade-off between model accuracy and privacy. We had initially explored a "generative replay" approach that uses no real data for memory rehearsal, but we found that the complexity of single-cell data led to an accumulation of minor deviations in the generated samples, ultimately causing biased results. This led us to the current design as a robust and accurate solution for academic collaborations where privacy constraints may be less stringent.

Looking forward, we believe the path to enabling strictly private collaborations involves integrating advanced privacy-preserving technologies, which is a key direction for our future work (as noted in the Discussion). We are actively exploring two main technical paths:

- The first is to build a *Federated Continual Learning Framework*. This would move beyond simple federated aggregation (like FedAvg), which cannot address catastrophic forgetting, and instead adopt sophisticated strategies. For example, we could combine knowledge distillation (e.g., FedGen) to share abstract insights rather than data, and then enable decentralized model evolution across a peer-to-peer network for effective collaboration.
- The second path involves *Direct Privacy Enhancements*. For scenarios that still require a shared memory, techniques like Differential Privacy could provide mathematical guarantees by adding controlled noise. Furthermore, Homomorphic Encryption could be leveraged to perform computations directly on encrypted data.

By integrating these technologies, we are confident that MIRACLE can evolve into a secure, compliant, and decentralized ecosystem for building dynamic biological atlases, fully achieving our vision of collaborative discovery.

11. It may be worth briefly discussing MIRACLE's extensibility to spatial omics data, where missing modalities and spatial artifacts pose distinct challenges. This would broaden MIRACLE's relevance to a rapidly evolving field.

Response:

We thank the reviewer for this insightful and forward-thinking suggestion. We agree that extending MIRACLE to spatial omics would significantly broaden the relevance and impact of our work, and this comment has prompted us to consider this direction more deeply.

The "missing modalities" challenge in spatial multi-omics, which the reviewer highlighted, is a scenario where our framework's mosaic integration capability is directly applicable. To address the specific challenges of incorporating spatial information and correcting for potential artifacts, we see several exciting avenues:

- A direct approach is to treat spatial coordinates as an additional numerical input or a new "spatial modality", allowing the model to implicitly learn spatial patterns.
- A more sophisticated method involves using graph neural networks or a spatial regularization term to explicitly model tissue architecture. This would also naturally help correct for spatial artifacts by enforcing local consistency.
- A particularly promising strategy is to leverage MIRACLE as a universal continual learning wrapper for state-of-the-art offline spatial integration models (e.g., MISO, SIMO, SpatialGlue).

Based on these considerations, we have revised the Discussion section of our manuscript to briefly outline the extensibility of MIRACLE to spatial omics.

12. In Figure 3a, add a brief caption note explaining that the blank lower triangle intentionally reflects stepwise integration, avoiding misinterpretation of incompleteness.

Response:

We thank the reviewer for this excellent suggestion to improve the clarity of Figure 3a.

To prevent any misinterpretation, we have revised the figure's internal labels and its corresponding caption:

- We have relabeled the first row to "Cumulative Batches", and added vertical labels on the left to distinguish the "Merged View" (row 1) from the "Split View" (rows 2-9).
- The caption has also been updated to explicitly state that the blank lower triangle is a design choice, reflecting the stepwise process of continual integration where data from future batches are not yet available.

We believe these changes make the figure's design much more intuitive.

Reviewer #3 (Remarks to the Author):

This manuscript introduces an online learning framework, MIRACLE, for multimodal single-cell data integration. The proposed approach aims to incrementally incorporate new data while retaining previously learned information, which could enhance cell label transfer accuracy and facilitate novel cell type discovery. These capabilities have the potential to significantly improve understanding of complex biological systems and disease mechanisms. While the methodology appears promising, I have several concerns and questions that I would like the authors to address before I can recommend the manuscript for publication.

Response:

We thank the reviewer for their insightful feedback, which has helped us significantly improve the manuscript's rigor. We have sought to address these concerns by conducting new, more challenging experiments and providing comprehensive clarifications on our methodology and claims.

1. The authors state that "Systematic evaluation demonstrates that MIRACLE efficiently integrates multimodal data across batches, modalities, and cell types with minimal memory usage, achieving accuracy comparable to offline methods while significantly reducing computational costs." However, the manuscript does not provide sufficient evidence to support the claim of "minimal memory usage". Section 2.2, titled "MIRACLE enables continual integration using limited memory," does not clearly define what aspect of memory is being measured—is this during training, inference, or across data accumulation? Additionally, it would be helpful to provide a comparative analysis of memory usage across other methods. Please clarify this aspect and explain why MIRACLE is expected to be memory-efficient.

Response:

We thank you for your insightful comments, which have helped us strengthen the manuscript's claims about memory efficiency. We address the four key aspects you raised as follows:

(1) Clarification of "minimal memory usage"

The term "minimal memory usage" was intended to highlight that MIRACLE maintains high accuracy using only a small rehearsal memory, not that it achieves a theoretical minimum. For instance, on the 523K-cell DHCM dataset, MIRACLE required a rehearsal memory of just 20K cells (~4% of the total data) to match the performance of its offline version (the upper benchmark) trained on the entire dataset.

To avoid ambiguity, we have revised this phrase to "constrained memory usage" to more accurately reflect the use of a small, controlled amount of rehearsal data, rather than implying a theoretical minimum.

(2) The specific aspect of memory being measured

You rightly pointed out the ambiguity of "memory". We now explicitly differentiate between two types:

- Rehearsal memory: The subset of historical data (denoted as $\mathcal{R}_t$ in Eq. 4b) stored to prevent catastrophic forgetting. Its capacity, an algorithmic hyperparameter, is measured by the number of cells. Our analysis in Sec. 2.2 focuses on how this capacity impacts

integration accuracy.

- Computational memory: The hardware resources (peak CPU RAM or GPU VRAM) consumed during execution. It is a key metric for scalability and is usually measured in gigabytes (GB).

In the revised manuscript, “memory” by default refers to “rehearsal memory”, while “computational memory” is used explicitly when discussing hardware consumption.

(3) Comparative analysis of computational memory

Following your suggestion, we benchmarked the computational memory usage of MIRACLE against other methods on both the original DHCM (523K cells) and a new, much larger Human Lung Cell Atlas (HLCA; 2.3M cells) [1] datasets. The results (Response Fig. 2.1) demonstrate:

- All MIRACLE methods maintain a constant and low memory footprint (~2.7 GB) on both datasets, irrespective of data size, due to their mini-batch training strategy.
- In contrast, PCA’s memory requirement grows linearly with data size, revealing a major scalability bottleneck (e.g., peaking at 34 GB on DHCM).
- Furthermore, PCA's memory usage is highly sensitive to feature dimensions. It consumed far more memory on the high-gene DHCM dataset (0.5M cells × 4078 genes; 34 GB) than on the larger but lower-gene HLCA dataset (2.3M cells × 2000 genes; ~3.2 GB), highlighting a bottleneck that MIRACLE’s architecture avoids.

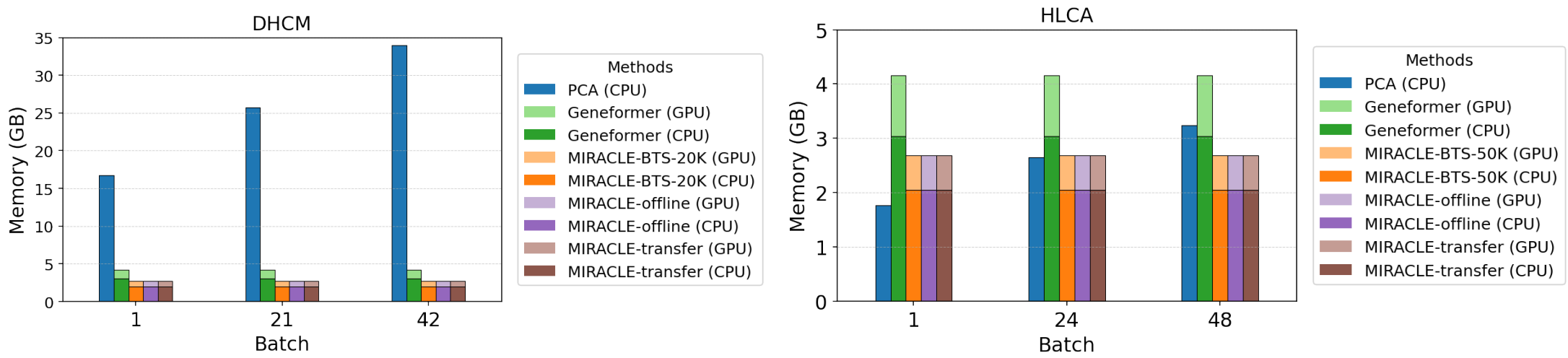

Response Fig. 2.1: Comparing computational memory of different methods on the 523K-cell DHCM (left) and 2.3M-cell HLCA (right) datasets.

These findings are now included in Results Sec. 2.2 and Supplementary Figs. 3 and 4d to quantitatively support our claims.

(4) Why MIRACLE is memory-efficient

MIRACLE's memory efficiency is achieved through three core design principles:

- Incremental updates with rehearsal memory: Unlike offline methods that must retrain on all accumulated data, MIRACLE processes only the new batch and a small, fixed-size rehearsal set. This design decouples computational cost from historical data size, addressing a key scalability bottleneck.
- Efficient rehearsal memory management: Our DPRS sampling strategy effectively preserves the data's underlying distribution within a limited budget. This allows us to use a very small rehearsal memory (e.g., 20K cells) without compromising integration

accuracy, ensuring both efficiency and facilitating model sharing.

- Mini-batch training: As a deep learning model, MIRACLE processes data in mini-batches. This strategy caps peak computational memory (RAM/VRAM) at a low, constant level, as the per-iteration data load is independent of the total dataset size. This explains why its memory usage profile remains flat.

References

[1] Sikkema, Lisa, et al. "An integrated cell atlas of the lung in health and disease." *Nature medicine* 29.6 (2023): 1563-1577.

2. Could the authors provide the loss functions, particularly the classifier loss? Furthermore, the justification for the selected hyperparameters is unclear. Is there a possibility that performance could be improved further through hyperparameter tuning? If these details are already provided, I apologize for overlooking them.

Response:

Thank you for your insightful and constructive feedback, which has significantly helped us improve the manuscript. We have revised the text accordingly.

Regarding the loss functions, we agree that a clear definition is essential. To address your point, we have added a new Supplementary Note 4 that provides a complete mathematical description of the model's loss functions. The main loss function, the encoder-decoder loss ($l^{f,g}$), is composed of three components: the evidence lower bound loss (l^{elbo}), a modality alignment loss (l^{mod}), and an information bottleneck loss (l^{ib}). The classifier loss (l^{r}), which you highlighted, is optimized adversarially against $l^{f,g}$ to achieve the disentanglement of batch information.

As for the hyperparameter justification, we have now clarified in the manuscript that: (1) The core hyperparameters are inherited from the base model, MIDAS, whose robustness was validated in its original study. (2) The key new parameter specific to MIRACLE—the rehearsal memory size—was empirically and conservatively set to better preserve biological fidelity in complex multimodal datasets. Please check the first paragraph in Sec. 4.11.3 for details.

Finally, on the point of performance improvement via tuning, we agree completely that performance could be further boosted by fine-tuning hyperparameters for specific tasks. Taking this idea a step further, we believe that in a dynamic continual learning setting, the optimal hyperparameters are likely not static and would need to adapt as data and tasks evolve. This perspective motivates our future work, as outlined in the Discussion, where we propose incorporating techniques like AutoML or meta-learning into the continual learning process. This would enable MIRACLE to automatically adapt its architecture or training strategies based on the intrinsic properties of incoming data.

3. Figure 2a is difficult to interpret. Could the authors clarify what is represented in the third row and the second column, for example? Additionally, the cell type combinations described in Supplementary Table 2 appear insufficient to support the conclusion that MIRACLE is superior in continually integrating distinct cell types. For instance, in the case where t=2, only

one new cell type is introduced compared to t=1. In addition, when t=3, there is no novel cell types.

Response:

We thank the reviewer for these insightful comments, which have helped us significantly improve the manuscript. In response, we have clarified Figure 3a and, most importantly, conducted a new, more rigorous experiment to validate our claims, as you suggested.

(1) Regarding Figure Interpretability

Thank you for highlighting the need for clarification. We believe you are referring to Figure 3a. The subplot in the third row and second column shows the UMAP distribution of cells exclusively from batch P2_0 at time point $t = 2$. This provides an isolated view to assess how well that specific new batch has been integrated.

Accordingly, we have revised the figure's labeling and caption in the manuscript. This includes adding clearer axis labels ("Cumulative Batches" and "Merged/Split View") and updating the caption to explain that the blank lower-left area is intentional, representing future, unobserved batches.

(2) Regarding the Experimental Design

We agree that while our original intent was to vary cell type compositions across batches, the resulting experimental design could indeed be more challenging, and we appreciate this highly constructive point.

Following your suggestion, we designed and conducted a new, more demanding experiment to stress-test MIRACLE's continual integration capabilities. The new “Dynamic WNN” dataset (Response Table 2.1) introduces more complex dynamics, such as the continual introduction of novel cell types, the alternating presence of key cell types (e.g., $CD8^{+}$ T cells) to test for catastrophic forgetting, and the simultaneous addition and removal of multiple cell types at each integration step.

Response Table 2.1: Construction of the Dynamic WNN dataset.

**Data batch**		**Cell type**							
		**B**	**$CD4^{+}$ T**	**$CD8^{+}$ T**	**DC**	**Mono**	**NK**	**Other**	**Other T**
**Original data**	**P1_0**	236	2198	1154	128	1890	494	184	94
	**P2_0**	455	1921	1140	93	1284	554	75	377
	**P3_0**	363	1422	525	72	1159	890	40	157
	**P4_0**	410	1524	913	113	1740	119	46	420
	**P5_0**	543	896	1157	216	2747	863	190	340
	**P6_0**	729	1062	356	95	2755	795	151	117
	**P7_0**	987	1258	1223	383	3748	997	84	174
	**P8_0**	394	2052	1646	123	3188	1075	100	330
**Constructed data**	**P1_0**	**0**	2198	1154	**0**	1890	**0**	**0**	**0**
	**P2_0**	455	1921	**0**	**0**	1284	554	**0**	**0**
	**P3_0**	363	**0**	525	72	1159	**0**	**0**	**0**
	**P4_0**	**0**	1524	**0**	113	1740	**0**	**0**	420
	**P5_0**	543	**0**	1157	**0**	**0**	863	**0**	340
	**P6_0**	**0**	1062	**0**	**0**	2755	795	151	**0**
	**P7_0**	987	1258	1223	383	**0**	**0**	84	**0**
	**P8_0**	**0**	2052	1646	**0**	3188	1075	**0**	330

The results on this new dataset are compelling (Response Fig. 2.2). Quantitative scIB metrics show that MIRACLE's online performance approaches the MIRACLE-offline upper benchmark, clearly surpasses the transfer baseline, and significantly outperforms other online methods. MIRACLE-offline demonstrates similar superiority in the offline setting. These results provide stronger evidence of MIRACLE's superiority for continual integration across dynamic batches and cell compositions.

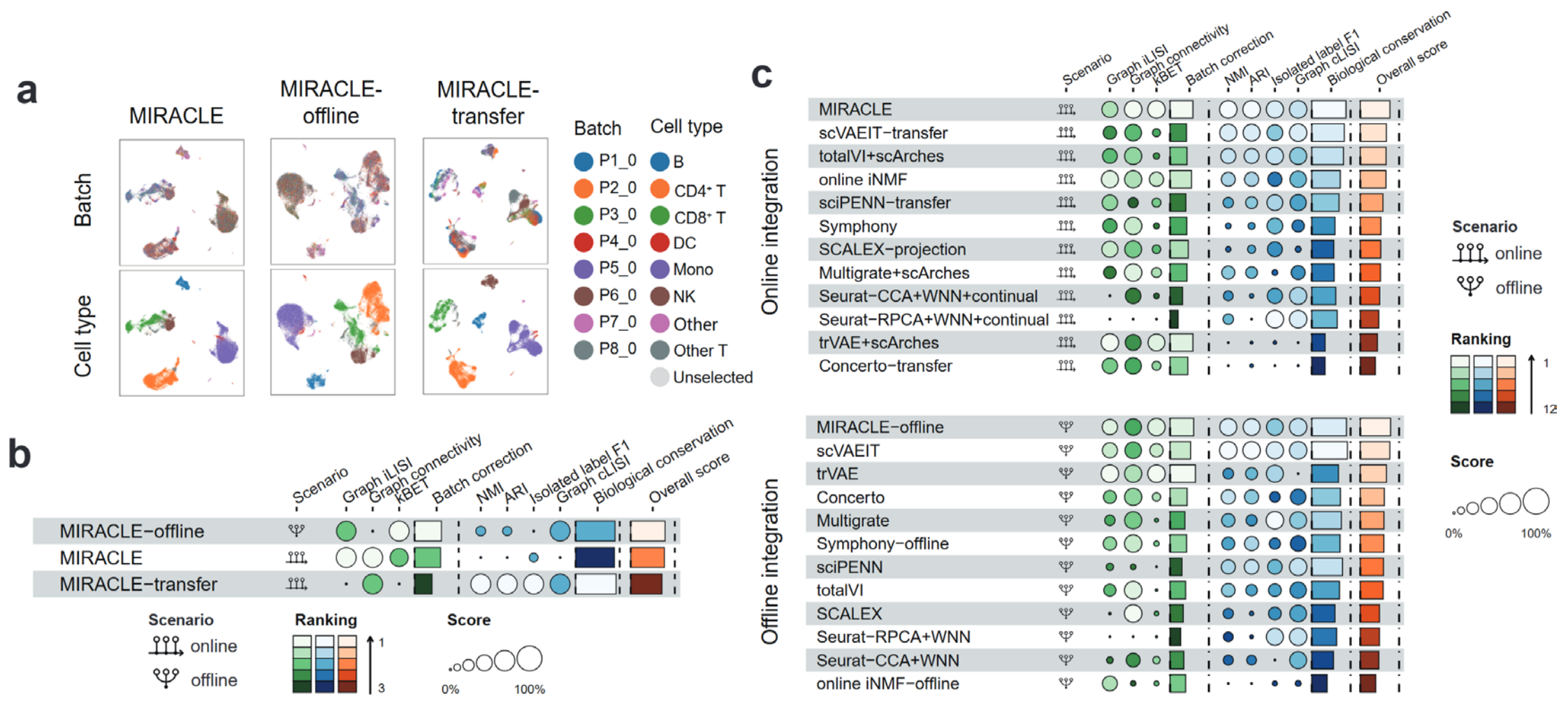

Response Fig. 2.2: Evaluation of MIRACLE on the Dynamic WNN bimodal dataset for continual integration across batches and cell types. **a**, UMAP visualization of biological states inferred by MIRACLE and its variants. **b**, scIB benchmarking comparing MIRACLE with its variants. **c**, scIB benchmarking comparing MIRACLE to 11 state-of-the-art single-cell integration methods in online (upper) and offline (lower) scenarios.

These new results and the corresponding UMAP visualizations are now incorporated into Sec. 2.3, Supplementary Figs. 8 and 9, and Supplementary Tables 6–8, supplementing our original findings to provide a more rigorous evaluation of MIRACLE's robustness.

4. Does the offline model utilize the full dataset or only a sampled subset? How does the offline model perform using full and sampled data, respectively?

Response:

Thank you for your insightful questions. Your suggestions prompted us to conduct a supplementary analysis that has strengthened our study.

(1) On the dataset for the offline model (MIRACLE-offline)

Yes, MIRACLE-offline is always trained on the complete dataset. As described in Methods (4.11.3), we designed it as an "upper benchmark" to represent the ideal integration performance achievable with unlimited memory and access to all data at once. This comparison directly assesses how well our memory-efficient online method performs relative to this ideal, unconstrained scenario.

(2) On the offline model's performance with full versus sampled data

To address your question about data scale, we conducted a supplementary experiment on the 523K DHCM dataset. We trained MIRACLE-offline on subsets of varying sizes (from 2K to 523K cells) sampled via BTS. The results (Response Fig. 2.3) showed that the model's performance is strongly dependent on data size. The scIB score improves with more data, begins to saturate around 100K cells, and peaks with the full dataset (scIB ≈ 0.79).

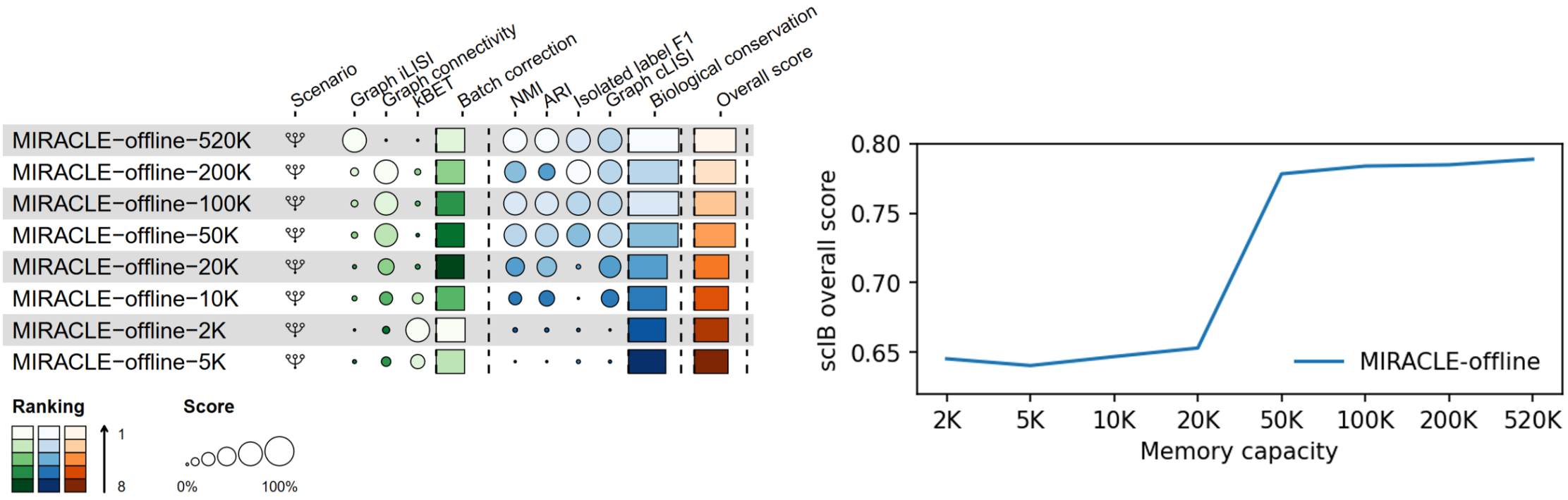

Response Fig. 2.3: Evaluation of MIRACLE-offline's performance with full versus sampled data on the DHCM dataset. Left: Detailed scIB scores. Right: scIB overall score.

Notably, on a 20K-cell sample, the score drops to just ~0.65. This large performance gap (0.79 vs. 0.65) quantitatively demonstrates the critical importance of data scale. It shows that a small, static data sample is insufficient for optimal integration, even for an offline model. This highlights the value of an online method (like MIRACLE) capable of learning from the entire dataset.

5. The manuscript claims that MIRACLE outperforms MIRACLE-offline in constructing a cross-tissue multimodal atlas. Could the authors elaborate on the reasons behind this improvement? What aspects of the online learning strategy contribute to better performance in this context?

Response:

We thank the reviewer for this question regarding the performance comparison between MIRACLE and MIRACLE-offline.

To clarify the point raised by the reviewer, the observation that MIRACLE can outperform MIRACLE-offline pertains to the mosaic integration benchmark on the DOTEA dataset (Fig. 4b and new Supplementary Table 10). In this specific test, the online MIRACLE (Overall Score: 0.7394) exhibited a slight performance advantage over the offline version (0.7379). We agree this result warrants an explanation.

We hypothesize that this advantage may be attributable to a "curriculum learning" effect, which is inherent to the sequential nature of online integration. The mechanisms are as follows:

- In an *offline learning* setting, the model is presented with the full complexity of the entire dataset simultaneously. This creates a highly complex optimization landscape, which may

increase the risk of the optimizer converging to a suboptimal local minimum.

- With *online learning*, in contrast, the model learns sequentially. It can first establish a robust representation on simpler or more complete data batches before adapting to more complex ones. This "easy-to-hard" progression may guide the optimizer toward a more generalizable solution.

Therefore, in response to the reviewer's question about which aspects of the online strategy contribute to this, we propose it is the stepwise learning paradigm itself. This effect is particularly relevant when the data sequence forms a natural curriculum, such as starting with a homogeneous reference before integrating heterogeneous queries.

We acknowledge that the observed performance margin is small and that a formal proof of this hypothesis is beyond the scope of the current study. However, we believe it is an important point for discussion. Based on the reviewer's feedback, we have revised the Discussion section to briefly incorporate our interpretation of this phenomenon.

6. The authors state that “Our results demonstrate that MIRACLE matches the integration quality of its offline counterpart, the performance upper limit.” This is a strong claim—is there a theoretical proof? As I understand, offline model could be tuned further to generate a better result for a certain dataset.

Response:

We thank the reviewer for this insightful point. We agree that the term "performance upper limit" is too strong and lacks sufficient scientific rigor.

As the reviewer rightly notes, the performance of an offline model is not a fixed theoretical maximum, as it can be further improved with hyperparameter tuning. Moreover, as you also highlighted in Comment 5, our own results demonstrate that the online strategy can occasionally outperform its offline counterpart.

Our initial rationale stemmed from a common practice in the continual learning field, where the performance of offline learning (with full data access) is often treated as a practical upper bound, while naive fine-tuning serves as a lower bound. This is because the former avoids catastrophic forgetting, whereas the latter is fully susceptible to it.

However, to enhance the rigor of our manuscript, we have adopted your excellent suggestion. We have systematically replaced all absolute terms such as "performance upper/lower limit" with the more appropriate phrase "upper/lower benchmark" throughout the revised text.

We believe this revision more accurately reflects the empirical nature of our work. Thank you again for helping us improve the scientific rigor of our manuscript.

Reviewer #3 (Remarks on code availability):

A tutorial is preferred, but not the code for the result.

Response:

Thank you for your constructive suggestion. We agree that a user-friendly tutorial is crucial for

our tool's adoption.

Following your advice, we have restructured our code to clearly separate the user tutorial from the manuscript reproducibility scripts. The abstract in the revised manuscript now links to our main project repository (https://github.com/sc-miracle/miracle), which provides direct access to both resources:

- Documentation and tutorials: Step-by-step guides to help users get started with MIRACLE, available at https://miracle-docs.readthedocs.io/en/latest/.
- Manuscript reproducibility: A dedicated repository containing the code to replicate all results from the manuscript, available at https://github.com/sc-miracle/miracle-reproducibility.

We are committed to continually improving the MIRACLE package and its documentation to best support the research community.

Point-to-point Responses to Reviewers' Comments

We sincerely thank the editor and the reviewers for their time and constructive evaluation. Reviewer #2 had no further comments, and Reviewer #3 confirmed that our previous responses adequately addressed their concerns, raising only one remaining point, which we address below.

Reviewers' Comments:

Reviewer #3 (Remarks to the Author):

The authors' responses have adequately addressed my concerns.

The remaining issue is that the documentation link provided in the response (https://miracle-docs.readthedocs.io/en/latest/) appears to be inaccessible (returns a "Project not found" error). I guess the latest one is https://miracle-v002.readthedocs.io/en/latest/. I encourage the authors to verify and correct this URL to ensure reproducibility and user accessibility.

Response:

We thank the reviewer for the careful check. The documentation is hosted at https://miracle-docs.readthedocs.io/en/latest/. At the time of the review, the documentation link had been temporarily changed, which produced the "Project not found" message; we have since restored it and verified that the page is now fully accessible. The same documentation is also linked from the project's GitHub repository (https://github.com/sc-miracle/miracle), ensuring reproducibility and user accessibility.
